# Synthesis and Biological Evaluation of a New Family of STAT3 Inhibitors by a Diversity Oriented Approach Based on the Ugi-Multicomponent Reaction

**DOI:** 10.1021/acsomega.6c07733

**Published:** 2026-09-09

**Authors:** Chiara Lambruschini, Manuel Anselmo, Luca Banfi, Nadia Bertola, Grazia Carbotti, Maria Maddalena Cavalluzzi, Giovanni Lentini, Valeria Marisa Rocca, Camillo Rosano, Maurizio Viale, Renata Riva

**Affiliations:** † Dipartimento di Chimica e Chimica Industriale, Università di Genova, Via Dodecaneso 31, 16146 Genova, Italy; ‡ AOM-IRCCS Ospedale Policlinico San Martino, U.O.C. Bioterapie, Largo R. Benzi 10, 16132 Genova, Italy; § Dipartimento di Farmacia−Scienze del Farmaco, Università degli Studi di Bari Aldo Moro, Via Orabona 4, 70125 Bari, Italy; ∥ AOM-IRCCS Ospedale Policlinico San Martino, U.O.S. Proteomica e Spettrometria di Massa, Largo R. Benzi 10, 16132 Genova, Italy

## Abstract

STAT3 (Signal Transducer and Activator of Transcription 3) is a protein frequently hyperactivated in a wide range of tumors, and as such, it has emerged as a promising and relatively recent therapeutic target for the development of novel anticancer agents. In this framework, we have designed and synthesized a new family of STAT3 inhibitors using a diversity-oriented strategy centered on the Ugi multicomponent reaction, enabling rapid access to structurally varied molecular scaffolds. We subsequently evaluated their antiproliferative activity across eight different tumor cell lines, revealing that four of the 23 newly obtained compounds exhibit pharmacologically meaningful growth-inhibitory effects within the micromolar range of concentrations and displayed promising drug-like profiles. Taken together, these findings highlight the potential of our approach to generate effective STAT3-modulating chemotypes and underscore the value of multicomponent reactions in accelerating early-stage anticancer drug discovery.

## Graphical abstract

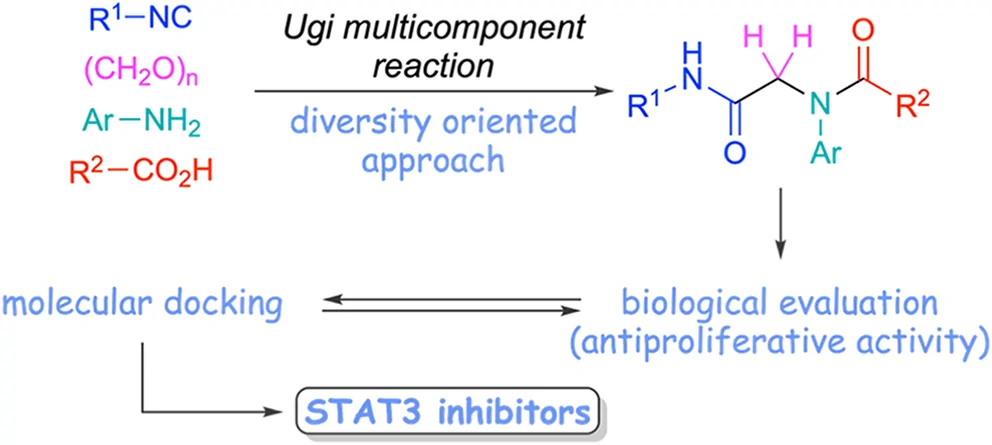

## Introduction

For oncologists, the demand for antitumor drugs with innovative or still-underdeveloped mechanisms remains strong. This need is driven by the limitations of current therapies, the resistance they may induce, and their toxicities, sometimes unexpected, even in the case of newer biotechnological treatments. The problem is further compounded by the rising incidence of cancers linked to modern industrial lifestyles and daily exposure to carcinogens.^1^ These factors motivate researchers to identify new or relatively novel molecules that could improve cancer control, which continues to be the second leading cause of death worldwide.^1^ Any effort to expand the repertoire of antitumor agents is therefore important, as it may lead to more effective and less toxic therapeutic strategies.

Although several treatments are effective against many tumor types, cancers such as breast, ovarian, prostate, lung, lymphoma, and leukemia often develop resistance. This highlights the urgency of identifying molecules or drugs that target cellular pathways not yet exploited by approved anticancer therapies. Among these potential targets, the STAT3 protein, frequently hyperactivated in many of the cancers mentioned, remains particularly promising.^2,3^

For these reasons, we identified the pharmacological and efficient inhibition of the STAT3 protein as the primary goal of our study. As is well-known, once STAT3 is activated in cells through phosphorylation and subsequent dimerization mediated by its SH2 domain, it promotes the transcription of antiapoptotic genes that play a crucial role in tumor cell survival and proliferation. This makes STAT3 a highly promising and attractive molecular target for anticancer therapy.^2,3^

Importantly, as said, STAT3 is often activated in various human malignancies, including leukemia, lymphoma, breast, prostate, lung, and ovarian cancers. Moreover, constitutive activation of STAT3 alone has been shown to induce cellular transformation in vitro and can convert nonmalignant cells into ones displaying a malignant-like phenotype.^4,5^ Persistent STAT3 activation is therefore implicated not only in cancer cell survival but also in tumor initiation and progression.

Consequently, inhibiting STAT3 dimerization could represent an innovative strategy to block the activation of downstream genes responsible for the accumulation of antiapoptotic proteins in tumor cells, thereby compromising their survival.^6^

Although several classes of small molecules with anti-STAT3 activity have been developed over the past two decades,^6,7^ none have yet reached standard clinical application. Thus, despite the intense research on STAT3 as a therapeutic target that has generated numerous studies and patents describing compounds of diverse chemical origins with varying degrees of STAT3 inhibitory activity and therapeutic potential, there is still a need for new compounds able to cover, if possible, the lack of anticancer drugs with this mechanism of action.

As is known, direct STAT3 inhibitors include those aimed at specific regions of the protein, such as the SH2, CCD, N-terminal, and DNA-binding domains.

Among them, many new chemical categories with good antiproliferative potential have recently emerged, including quinone derivatives, triarylpiperazine derivatives, 4-benzyl-1-amidopiperazine derivatives, 2,4-dihydroxybenzophenone hydrazones, tricyclic fused-ring derivatives, benzimidazole-1,3,4-thiadiazole derivatives, *N*-(thiazol-2-yl)­benzamide derivatives, as well as bioactive natural compounds such as celastrol, euonyquinone A, pretrichodermamide B, isoquercitrin, luteolin, and others, as well described in the review by Gao et al.^7^

Nevertheless, only a small fraction of these numerous compounds has been patented, and even fewer have reached clinical evaluation with interesting results. Among these, we mention TTI-101, analyzed in a phase I study on patients with advanced tumors, OPB-111077, studied in a Phase II study also in patients with advanced tumors, and, finally, Napabucasin (BBI608), which, as the only case, reached two phase III studies in combination with paclitaxel and FOLFIRI in patients with metastatic colon cancer.^8–11^ Often, these phase studies were stopped due to the low antitumor activity observed in humans and the emergence of characteristic toxic effects, such as metabolic impairment^12^ with resulting peripheral neurotoxicity^12,13^ other than cardiotoxicity, or hepatotoxicity.^13^

Here, we describe the synthesis and preliminary evaluation of several new compounds of first- and second-generation (derived from active or inactive compounds of the first generation) recently patented by us.^14^ These molecules act as direct inhibitors of STAT3, specifically targeting its SH2 domain. Our work has enabled us to select four molecules with pharmacologically significant antiproliferative activity, warranting further investigation of both their activity and their specific mechanisms of action.

## Results and Discussion

### Synthesis of the New STAT3 Inhibitors

For the synthesis of the new molecules, we focused on a diversity-oriented synthesis (DOS) strategy, according with the pioneering review of Schreiber and Burke,^15^ because this would have enabled us to rapidly optimize the structures of the molecules based on the results of the initial biological tests. For this reason we planned to use a highly convergent strategy based on the Ugi multicomponent reaction (U-MCR), an isocyanide-based reaction widely used in the field of drug discovery and medicinal chemistry,^16–21^ including molecules with anticancer activity.^22^ To the best of our knowledge, only one approach to STAT3 inhibitors through a MCR has been reported,^23^ but the Ugi reaction has never been used before. In the Ugi reaction four reagents are involved at the same time, specifically an isocyanide, a carbonyl compound (most frequently an aldehyde), a primary amine and a carboxylic acid, which allows a fast access to peptidomimetic structures of general structure **1** (Scheme 1), with the undeniable advantage of being able to easily vary the decorations characterizing the four building blocks and achieving in most cases a significant atom economy, one of the 12 principles of green chemistry.^24^ Furthermore, the same Ugi reaction has been described as a green alternative toward, for example, active pharmaceutical ingredients (APIs).^25^

**Scheme 1:**
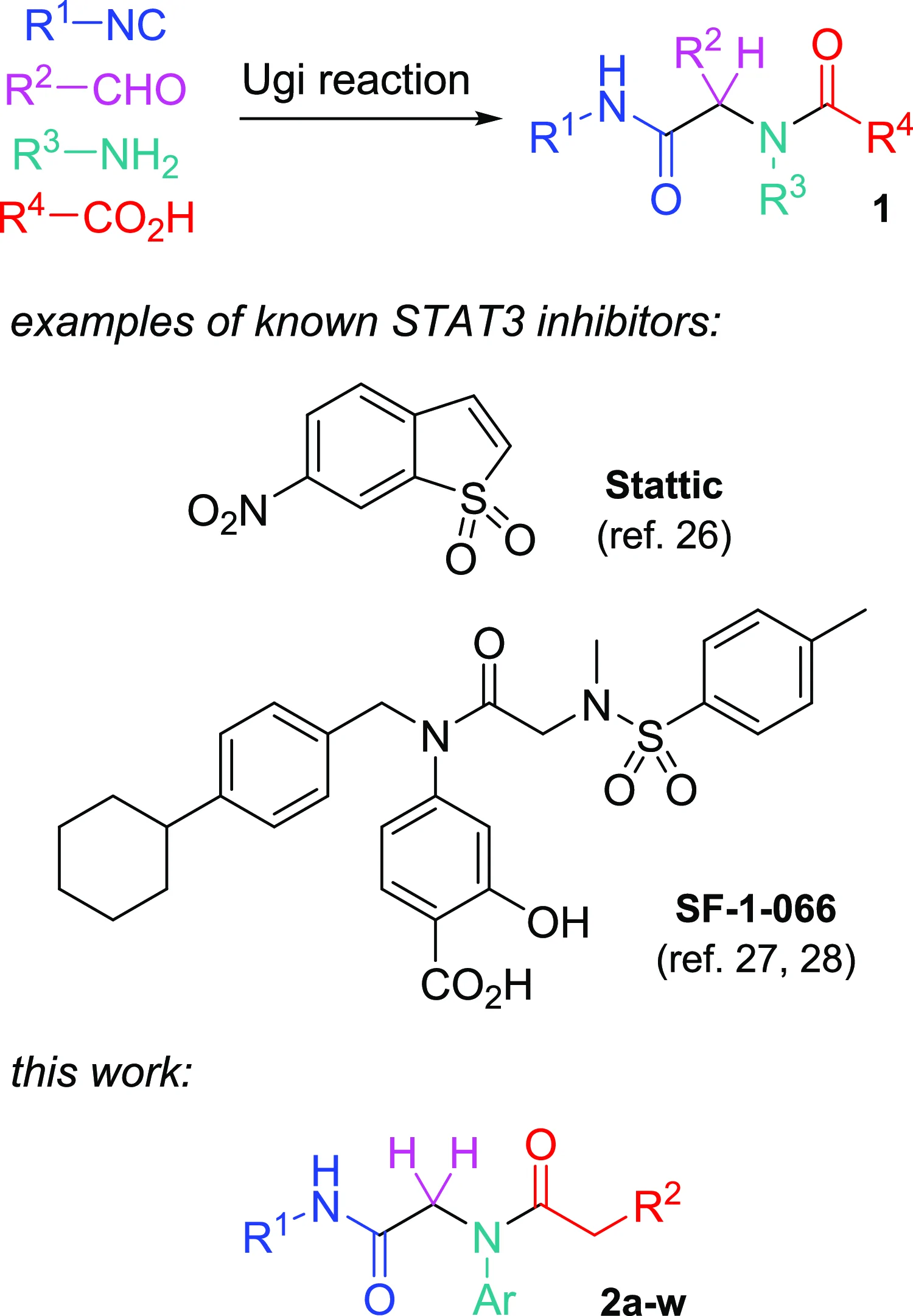
Synthetic Strategy

To decide how to explore the diversity introduced by the decorations in the Ugi reagents, we were in part inspired by the structure of some known STAT3 inhibitors, which were, however, synthesized in a completely different manner through a multistep approach. In particular, the heterocyclic structure of **Stattic** ^26^ suggested a possible fragment to be incorporated in the acid component, as well as the sulfonamide moiety of **SF-1–066** (Scheme 1).^27,28^

Based on the literature^27,28^ we hypothesized that an aromatic isocyanide would play an important role in the biological activity of these molecules. Nevertheless, we included also an aliphatic isocyanide in our study. As it did not result in improved activity (see below), we chose not to investigate further aliphatic isocyanides. Therefore, we explored the diversity resulting from the presence of substituents in all positions of the aromatic ring, including the 4-cyclohexylphenyl moiety present in **SF-1–066**. The amines we used were inspired by **SF-1–066**, but were not necessarily salicylic derivatives, and we mainly investigated the diversity of substituents in the *meta* position with respect to the amino group, placing especially electron-withdrawing groups, including the nitro group present in **Stattic**. On the contrary, the carbonyl component used has always been formaldehyde so as not to generate a stereogenic center in the Ugi products with the formation of racemic mixtures. Therefore, our molecules can be summarized with the generic structure **2** (Scheme 1). Finally, in some cases, as explained later, protected Ugi partners were necessary, and the protecting groups were removed after the MCR, obtaining structures **2**.

The reagents to be used for the synthesis of the new library of 23 compounds (**2a**–**w**) have been chosen among commercially available ones (**4**, **5**, **12**, **13**, **16**) or have been synthesized by known [**8**, chosen because of the presence of an electron-withdrawing group,^29^ and **11** and **17** ^27^ (for the last two, see also the Supporting Information)] or by original procedures (**3**, **6**, **7**, **9**, **10**, **14**, **15**, **18**) (Scheme 2).

**Scheme 2:**
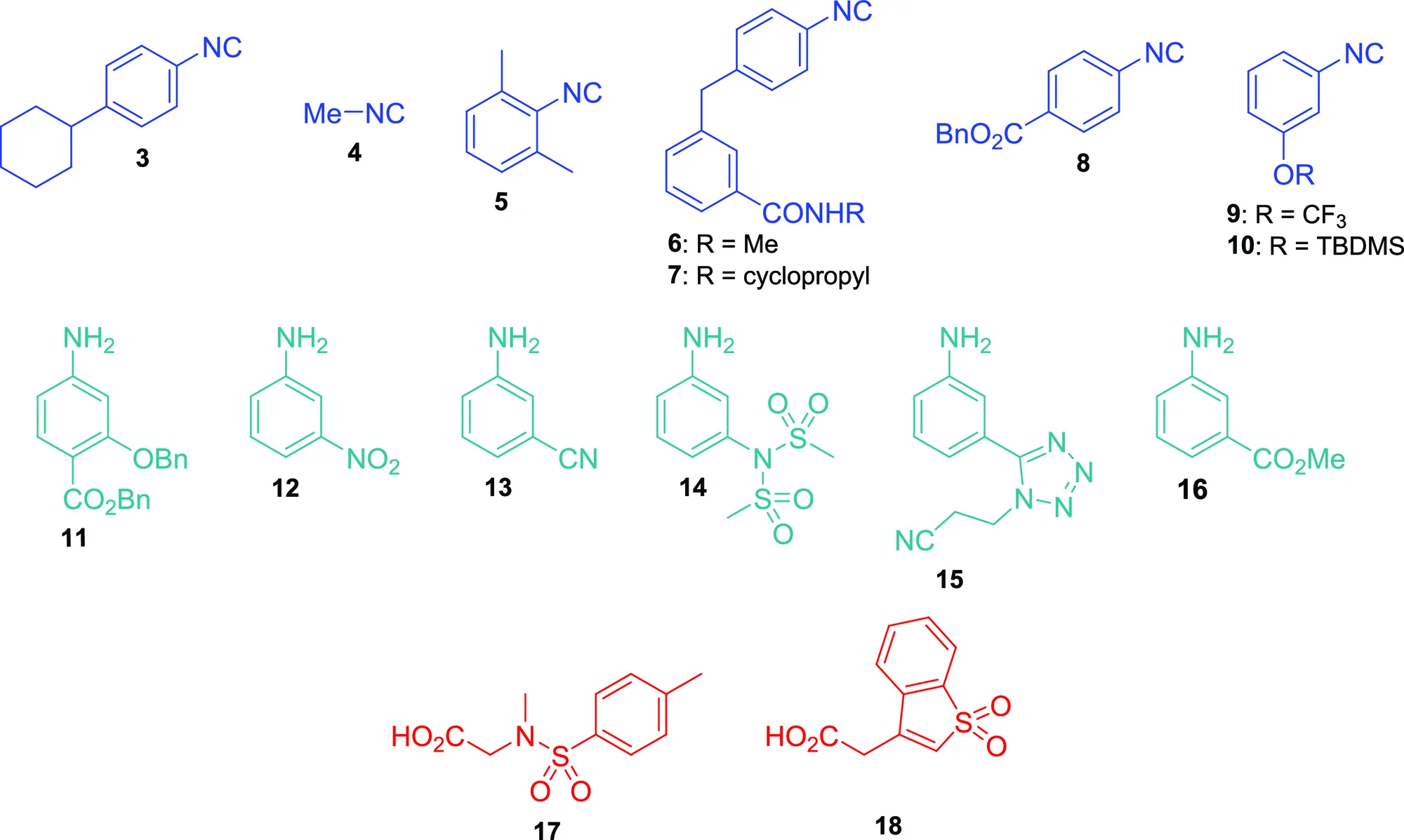
Building Blocks Used for the Ugi Reaction

The original syntheses of isocyanides **3**, **6**, **7**, **9**, **10** are summarized in Schemes 3–5. They all share the two final steps, namely the transformation of the primary amines (obtained by Cbz removal on **20**, **28**, **29** or directly starting from **32** and **35**) into the corresponding formamides **21**, **30**, **31**, **33**, **36**, followed by the dehydration leading to the isocyanides. As dehydrating agents we used either diphosgene (ClCO_2_CCl_3_) or POCl_3_, and the reaction conditions for both steps have been optimized for every single compound. Both reagents cannot be considered as green as the Ugi reaction. However, since the primary focus of this project was to generate as fast as possible a library of compounds for biological evaluation, our efforts were directed toward obtaining the target molecules using the most efficient synthetic strategy at the laboratory scale. Obviously, when there will be the need to prepare these isocyanides on a larger scale, greener methods for dehydration will be explored, taking advantage of some recent advancements in this field.^30–32^ Furthermore, owing to the relatively low stability of the aromatic isocyanides, they have been prepared and immediately used in the MCR, just after a very fast filtration over silica gel.

**Scheme 3:**
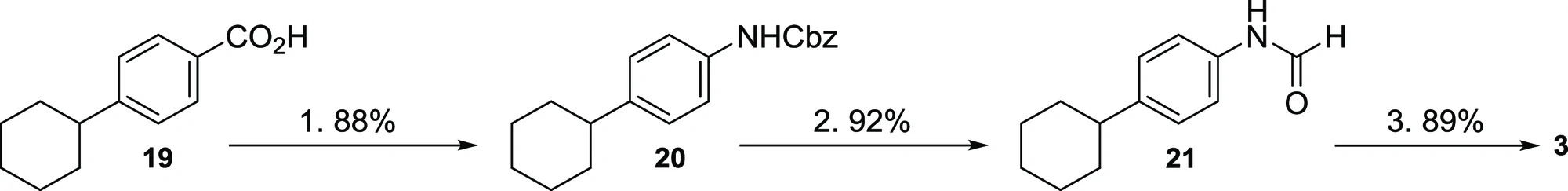
Synthesis of Isocyanide **3** ^a^

a Conditions: 1. (a) DPPA, Et_3_N, toluene, 80 °C; (b) BnOH, reflux; 2. (a) H_2_, Pd/C 10%, *i*-PrOH, rt; (b) HCO_2_H, DCC, 4-DMAP, CH_2_Cl_2_, rt; 3. diphosgene, Et_3_N, CH_2_Cl_2_, 0 °C.

The key step to isocyanide **3** was the Curtius rearrangement of **19**, affording **20** after trapping the intermediate isocyanate with benzyl alcohol (Scheme 3).

Isocyanides **6** and **7** bear an additional secondary amide group, which can engage in additional hydrogen bonds, potentially enhancing binding affinity. Moreover, they present a benzylic carbon in the position para to the isocyanide connecting two aromatic rings, and we wanted to assess if this could enhance the positive effect arising from the presence of two aromatic rings, or if the higher steric requirements, or the combination of them, would influence the activity.

The synthesis of **6** and **7** (Scheme 4) is based on the common intermediate **27**, which can be prepared through the Suzuki coupling between **22** and **23** affording **24**, followed by the Pinnick oxidation to acid **25**, directly submitted to the Curtius rearrangement and formation of the benzyloxycarbonyl derivative **26**, finally hydrolyzed to **27**. The coupling affording the amides **28** and **29**, performed using 1,1′-carbonyl diimidazole, required DBU as base for **28**, because triethylamine was not efficient enough for converting the methylamine hydrochloride into the corresponding amine, while for **29** triethylamine was suited.

**Scheme 4:**
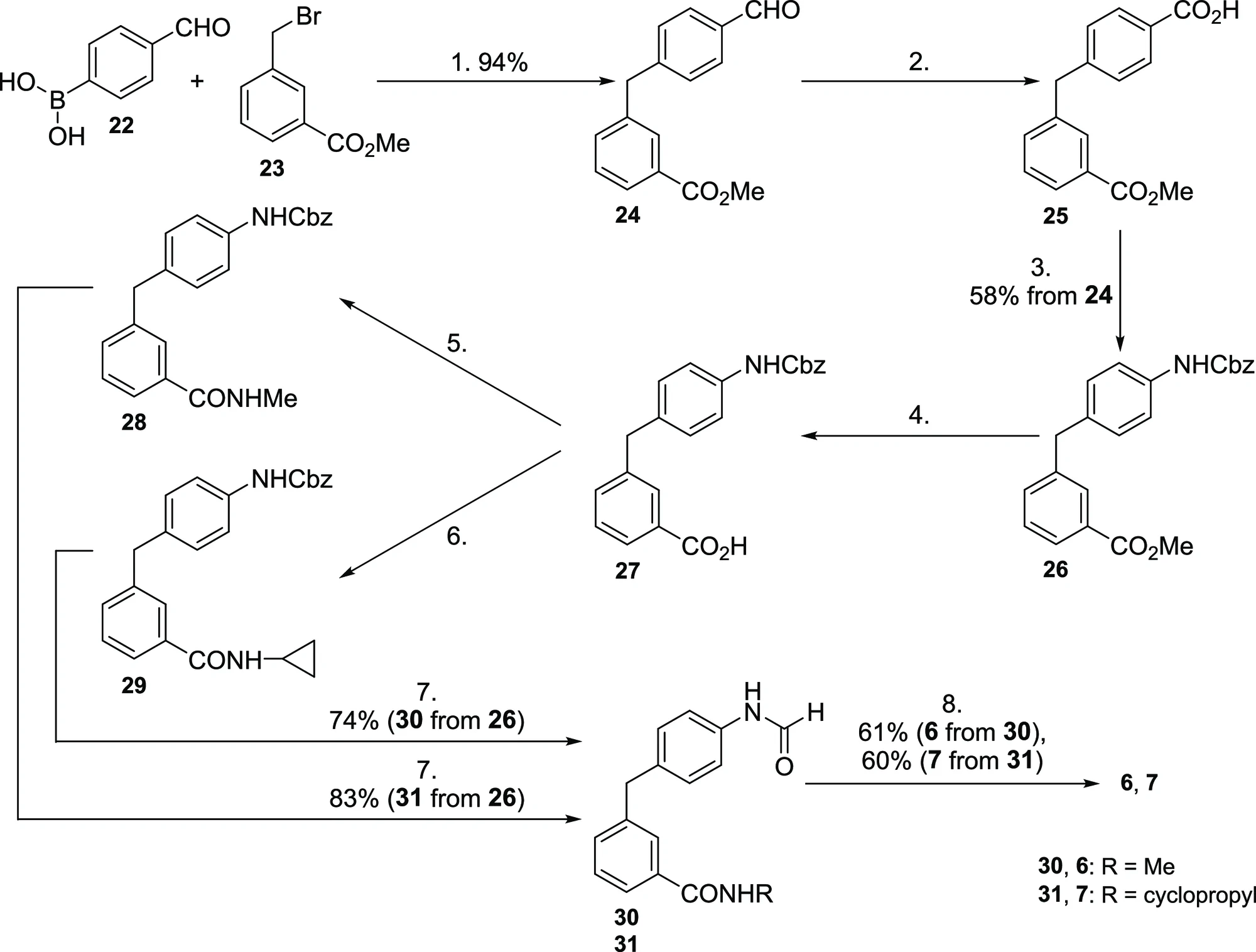
Synthesis of Isocyanides **6** and **7** ^a^

a Conditions: 1. K_3_PO_4_, PdCl_2_(PPh_3_)_2_, 1,4-dioxane/H_2_O 10:2, 80 °C; 2. 2-methyl-2-butene, NaClO_2_, NaH_2_PO_4_, THF/H_2_O, rt; 3. (a) DPPA, Et_3_N, toluene, 80 °C; (b) BnOH, reflux; 4. LiOH (1 M in H_2_O), THF/H_2_O, rt; 5. CDI, MeNH_2_·HCl, DBU, THF, rt; 6. CDI, cyclopropylamine, Et_3_N, THF, rt; 7. (a) H_2_, Pd/C 10%, MeOH, rt; (b) HCO_2_Et, reflux; 8. POCl_3_, Et_3_N, CH_2_Cl_2_, 0 °C.

Finally, the hydrogenolysis of **28** and **29**, followed by formylation, afforded **30** and **31** with an excellent overall yield in 4 steps from **26** without any purification of the intermediates, which were dehydrated to **6** and **7** respectively with POCl_3_, the only conditions giving a clean reaction. It should be noted that the more convergent approach, namely the transformation of **26** into the corresponding formamide, followed by installation of the two different amide groups after ester hydrolysis, was not feasible, as the formamide also underwent hydrolysis.

Isocyanide **9**, bearing an OCF_3_ group (Scheme 5), was synthesized because its presence is known to generally improve the activity of drugs, although in a poorly explored and unpredictable manner.^33^ This group is a good acceptor of hydrogen bond and is currently considered a privileged substituent also because it enhances the lipophilicity of the molecule, which might have some beneficial effect on the activity.

**Scheme 5:**
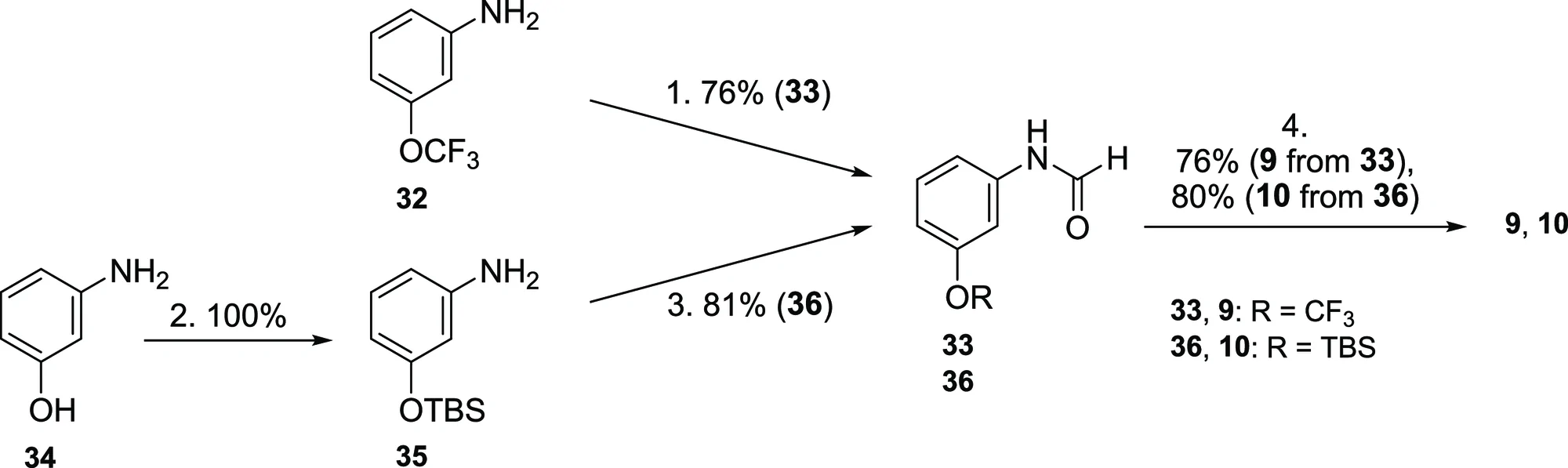
Synthesis of Isocyanides **9**, **10** ^a^

a Conditions: 1. HCO_2_H, CDI, Et_3_N, CH_2_Cl_2_, rt; 2. TBS-Cl, imidazole, CH_2_Cl_2_, rt; 3. HCO_2_H, DCC, 4-DMAP, CH_2_Cl_2_, rt; 4. diphosgene, Et_3_N, CH_2_Cl_2_, −15 to 0 °C.

We were also interested in the evaluation of a phenol group in the same position. However, phenols are usually incompatible with the Ugi reaction, owing to the potential occurrence of the competing Ugi–Smiles reaction,^34^ where the phenol may substitute the carboxylic acid as nucleophile after the addition of the isocyanide to the imine. To avoid that, protected phenols have been used, both starting from amine **34**, which was silylated to **35**, or from 4-aminosalicylic acid, the precursor of **11** (see Supporting Information), which was doubly benzylated. Of course the protecting groups have been removed after the MCR. The synthesis of isocyanides **9** and **10** was accomplished by transforming amines **32** and **35** into the corresponding formamides **33** and **36** respectively, and then dehydrating them with diphosgene (Scheme 5).

All used amines (**11–16**) are characterized by the presence of a group with different electronic properties in the *meta* position. The *N*-sulfonyl moiety is a well-known and promising fragment for cancer therapy^35^ and therefore we planned to introduce it in one of our amines. The monosulfonylation of **37** was unsuccessful even using a substoichiometric amount of mesyl chloride, with **38** being consistently by far the major product. Therefore, we focused on the synthesis of amine **14**, obtained by catalytic hydrogenation of the corresponding nitro derivative **38** (Scheme 6).

**Scheme 6:**
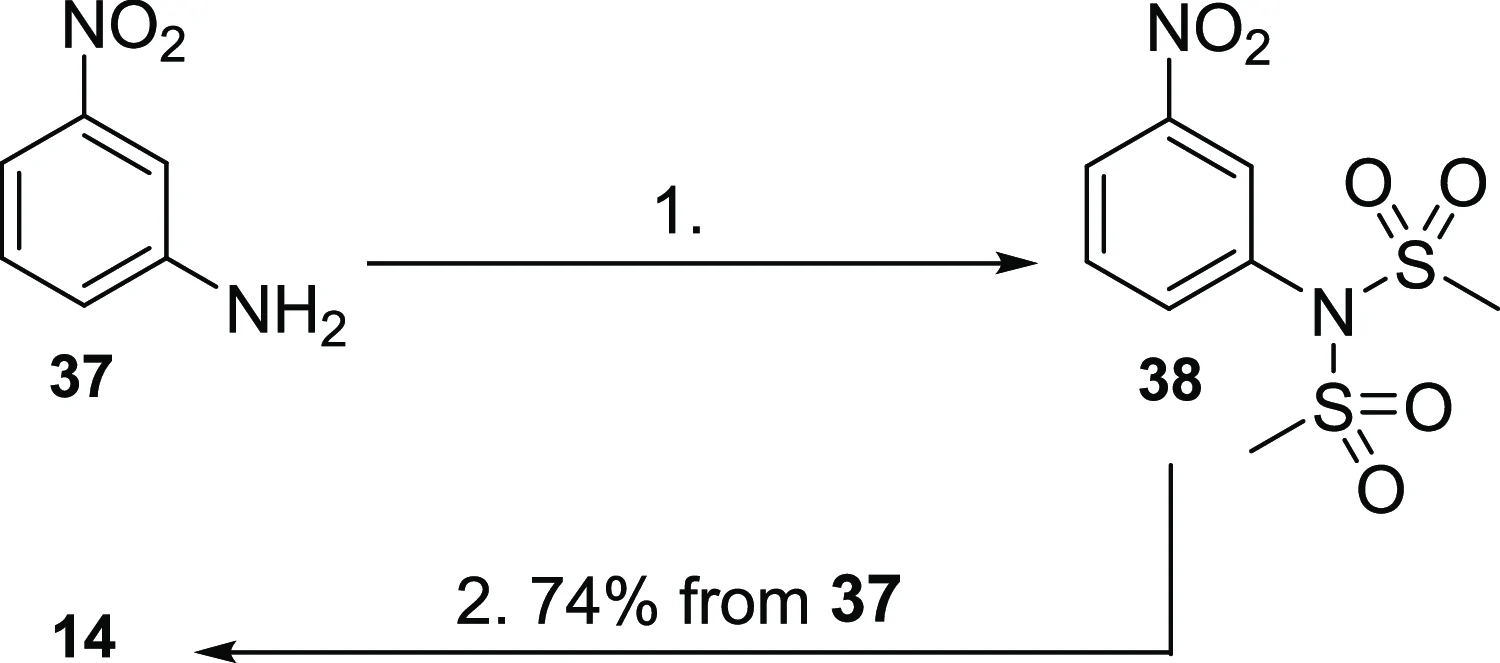
Synthesis of Amine **14** ^a^

a Conditions: 1. MeSO_2_Cl, Et_3_N, CH_2_Cl_2_, rt; 2. H_2_, Pd/C 10%, MeOH, rt.

In Scheme 7, we report the synthesis of **15**, chosen because tetrazole is often used as a metabolism-resistant bioisostere to carboxylic acid.^36^ For the synthesis of the heterocycle, we exploited the known one-pot transformation of a secondary amide into a tetrazole moiety.^37^ As starting material, we used acid **39**, and the sequence of the individual steps leading to product **45**, the precursor of the tetrazole, was carefully optimized to ensure a robust and reliable synthetic pathway.

**Scheme 7:**
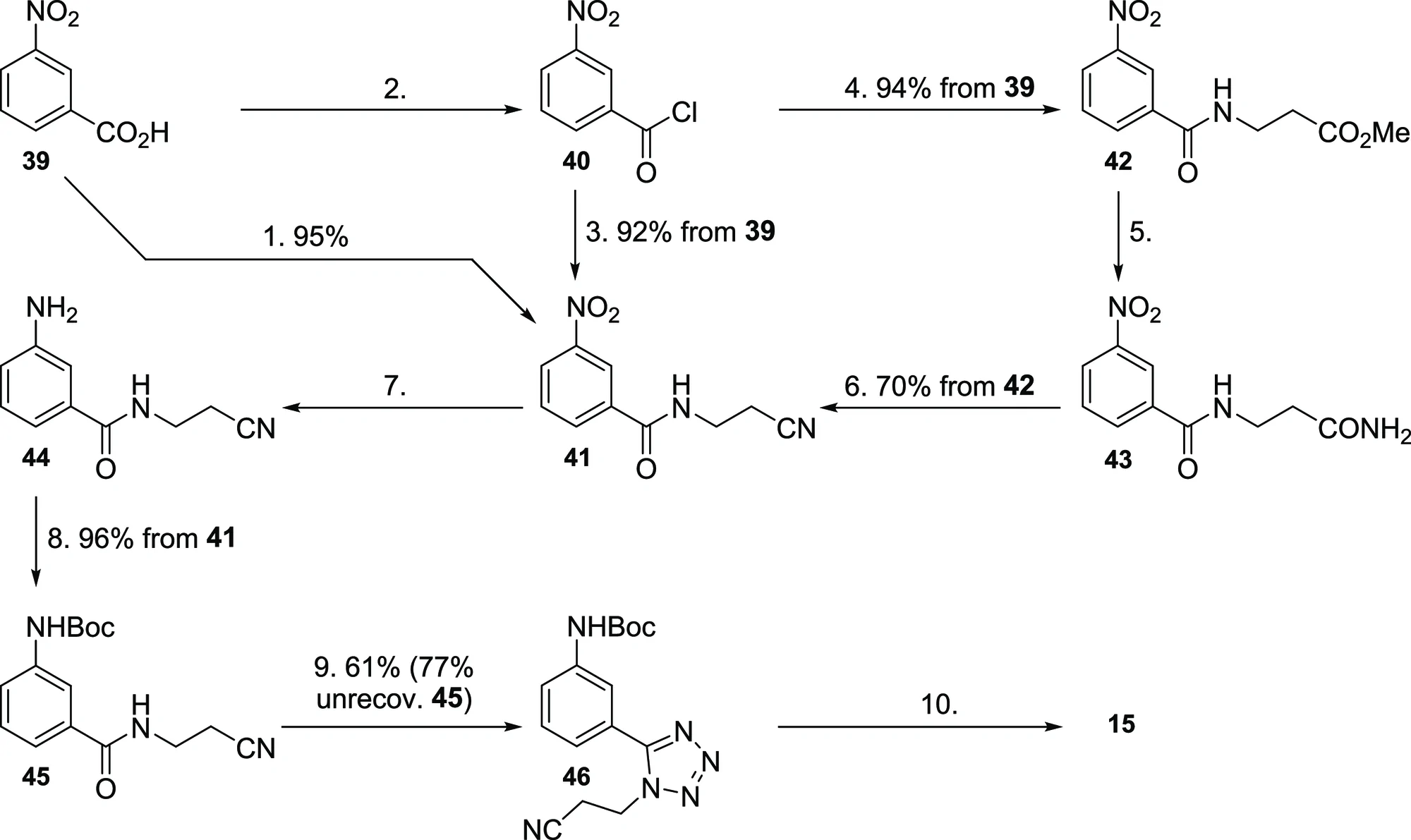
Synthesis of Amine **15** ^a^

a Conditions: 1. NH_2_CH_2_CH_2_CN, EDC·HCl, HOBT, DMF, rt; 2. (COCl)_2_, cat. DMF, 0 °C to rt; 3. NH_2_CH_2_CH_2_CN, Et_3_N, CH_2_Cl_2_, 0 °C to rt; 4. NH_2_CH_2_CH_2_CO_2_Me, Et_3_N, CH_2_Cl_2_, 0 °C to rt; 5. NH_3_ (7 M in MeOH), 80 °C; 6. POCl_3_, pyridine, −15 °C; 7. iron powder, aq NH_4_Cl, EtOH, 70 °C; 8. Boc_2_O, 1,4-dioxane, 70 °C; 9. PPh_3_, DIAD, TMSN_3_, THF, 0 °C to rt; 10. TFA/CH_2_Cl_2_ 1:2, 0 °C, then basic extraction.

The crucial precursor turned out to be **41**, which can be prepared in a similar yield by a direct condensation of **39** with 3-aminopropanenitrile (EDC·HCl, HOBT) or through the acyl chloride **40**.

The CH_2_CH_2_CN moiety was chosen because it can be easily removed under basic conditions after the formation of the tetrazole.^37^ However, since 3-aminopropanenitrile is quite harmful, and it may undergo polymerization upon prolonged storage, even at low temperature, to give a potentially explosive yellow solid,^38^ we preferred to develop a different, safer and more cost-effective synthesis of **41**, although slightly less efficient. To this end, we treated **40** with β-alanine obtaining **42**, which was submitted to ammonolysis with a 7 M solution of ammonia in MeOH heated at 80 °C in a closed vessel. Finally, the primary amide was dehydrated by means of POCl_3_, affording **41**. An efficient nitro group reduction with iron powder and Boc protection of amine **44** afforded **45**, which was converted into **46** by a Mitsunobu-type reaction by treatment with TMSN_3_ in the presence of triphenylphosphine and DIAD, whose hydrazine dicarboxylate can be more easily separated, compared to the one derived from more widely used DEAD. Boc removal afforded the amine without needing any purification.

Finally, the chosen carboxylic acids are aryl (**17**) or heteroarylacetic (**18**) derivatives. Unknown **18** was obtained in one-step by oxidation of the benzothiophene sulfur atom to sulfone of commercially available acid **47** (Scheme 8).^39^

**Scheme 8:**
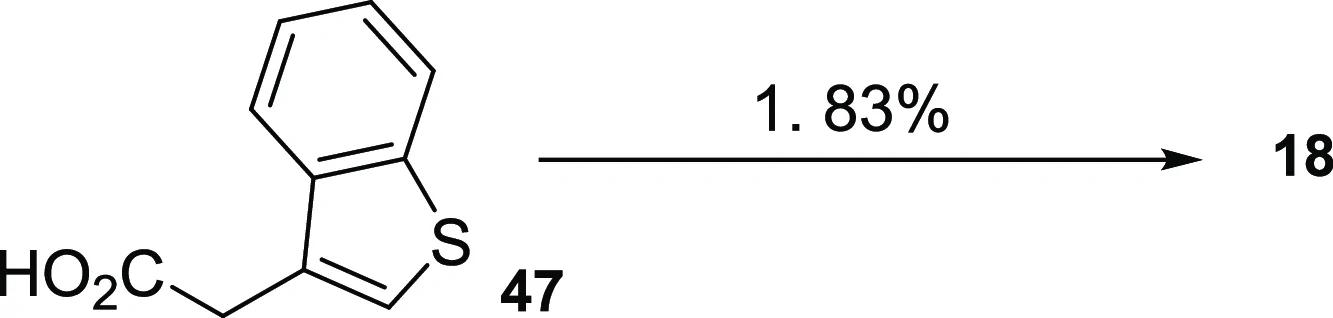
Synthesis of Acid **18** ^a^

a Conditions: 1. H_2_O_2_ 30%, AcOH, 65 °C.

The Ugi reaction was performed as summarized in the scheme of Table 1. This time, the imines of paraformaldehyde **48**, which slowly depolymerizes *in situ*, were preformed at 70 °C in the presence of powdered 3 Å molecular sieves, because of the lower reactivity of the aromatic amines. Then the appropriate isocyanide and acid were added, and the Ugi reaction was performed at 50 °C. This allowed us to isolate 18 Ugi products, in most cases with good to excellent yield. The only drawback of these compounds is their poor solubility in organic solvents, which sometimes made purification by flash chromatography and/or trituration challenging.

**Table 1:**
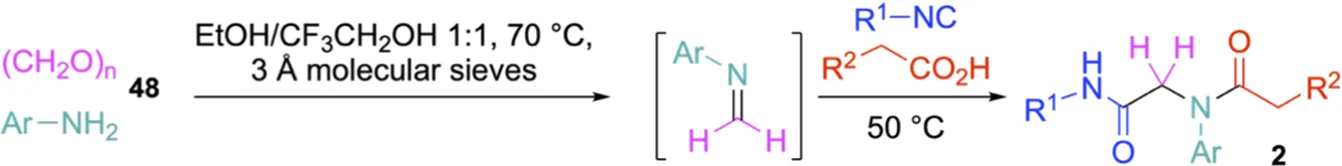

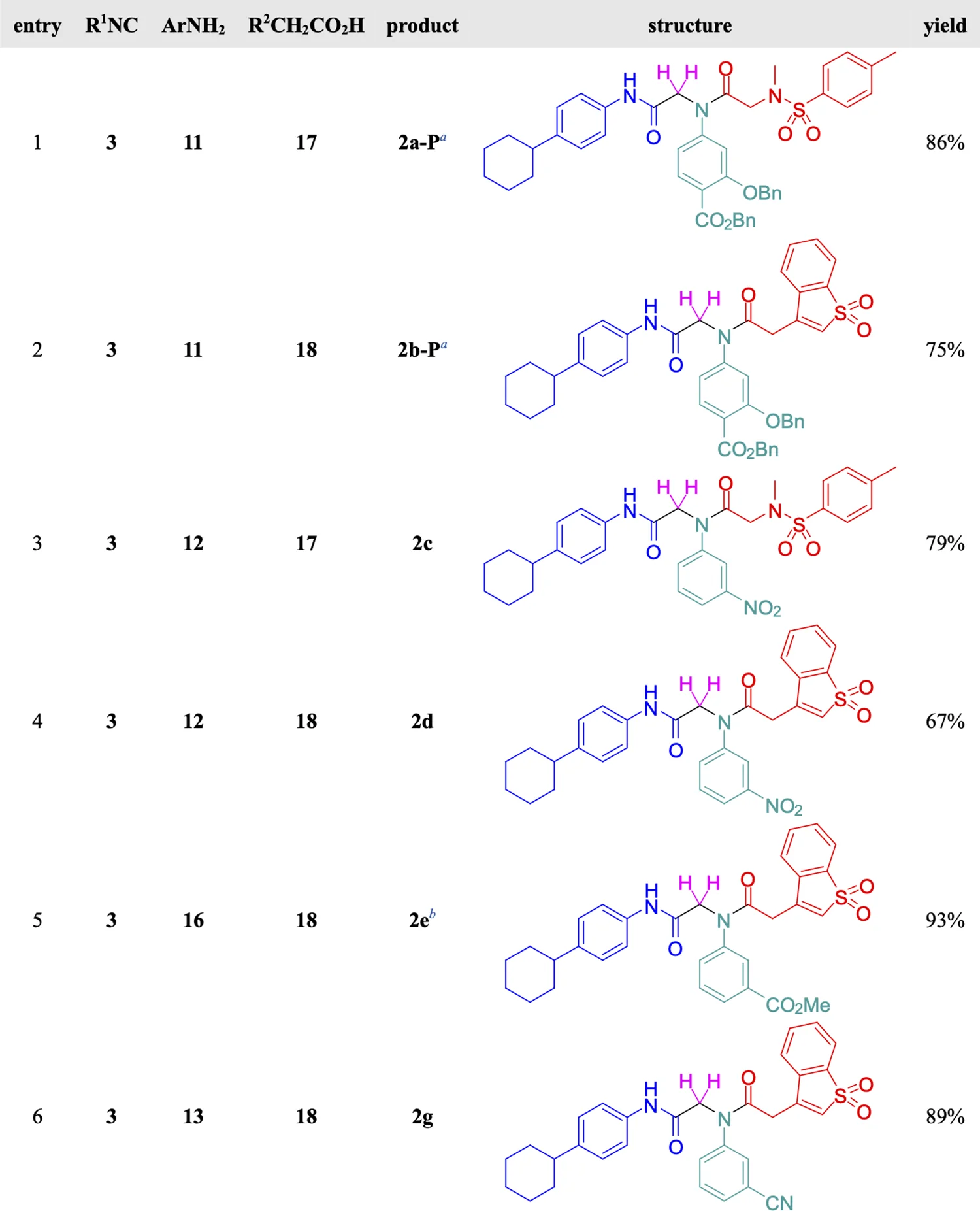

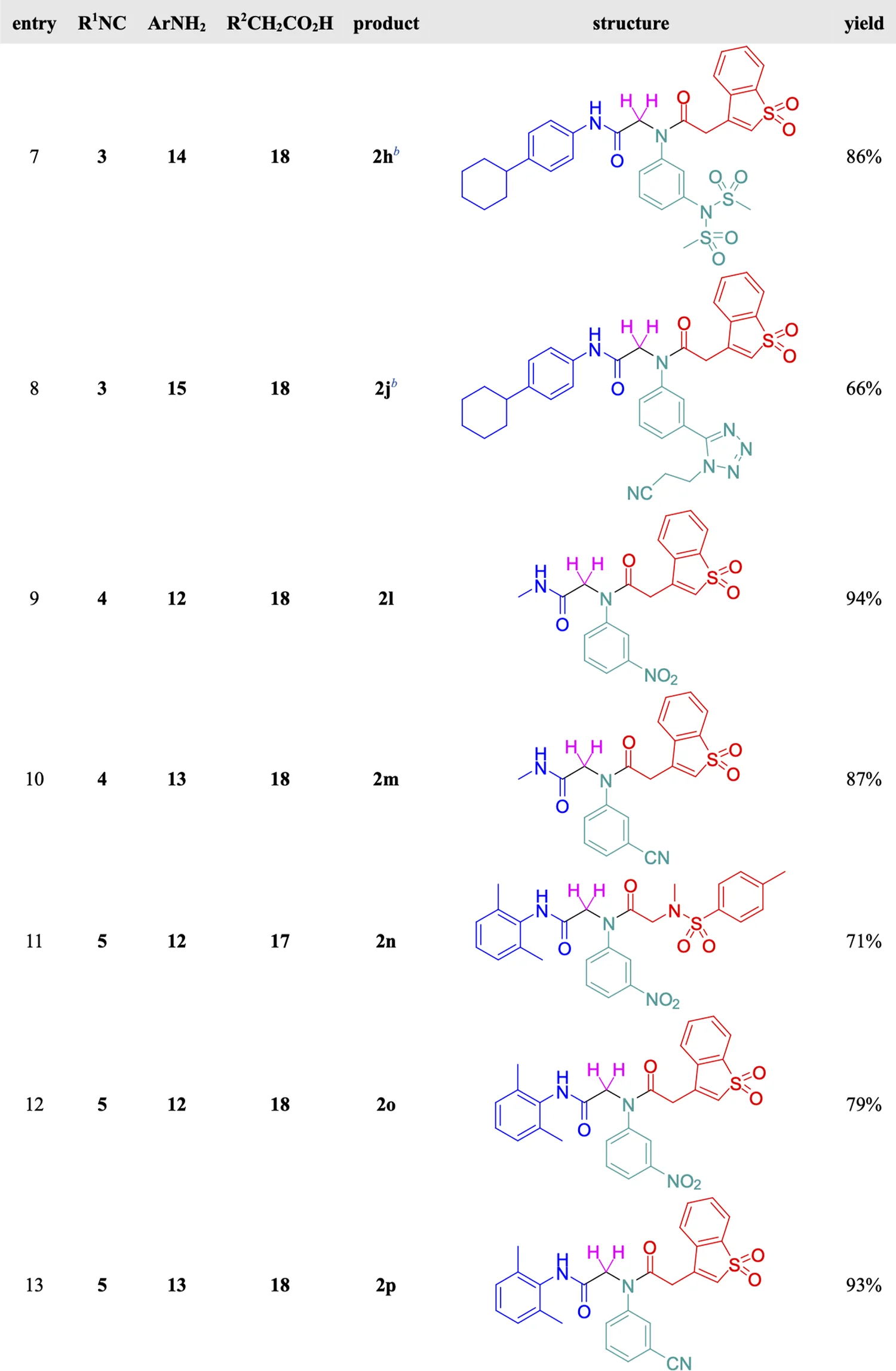

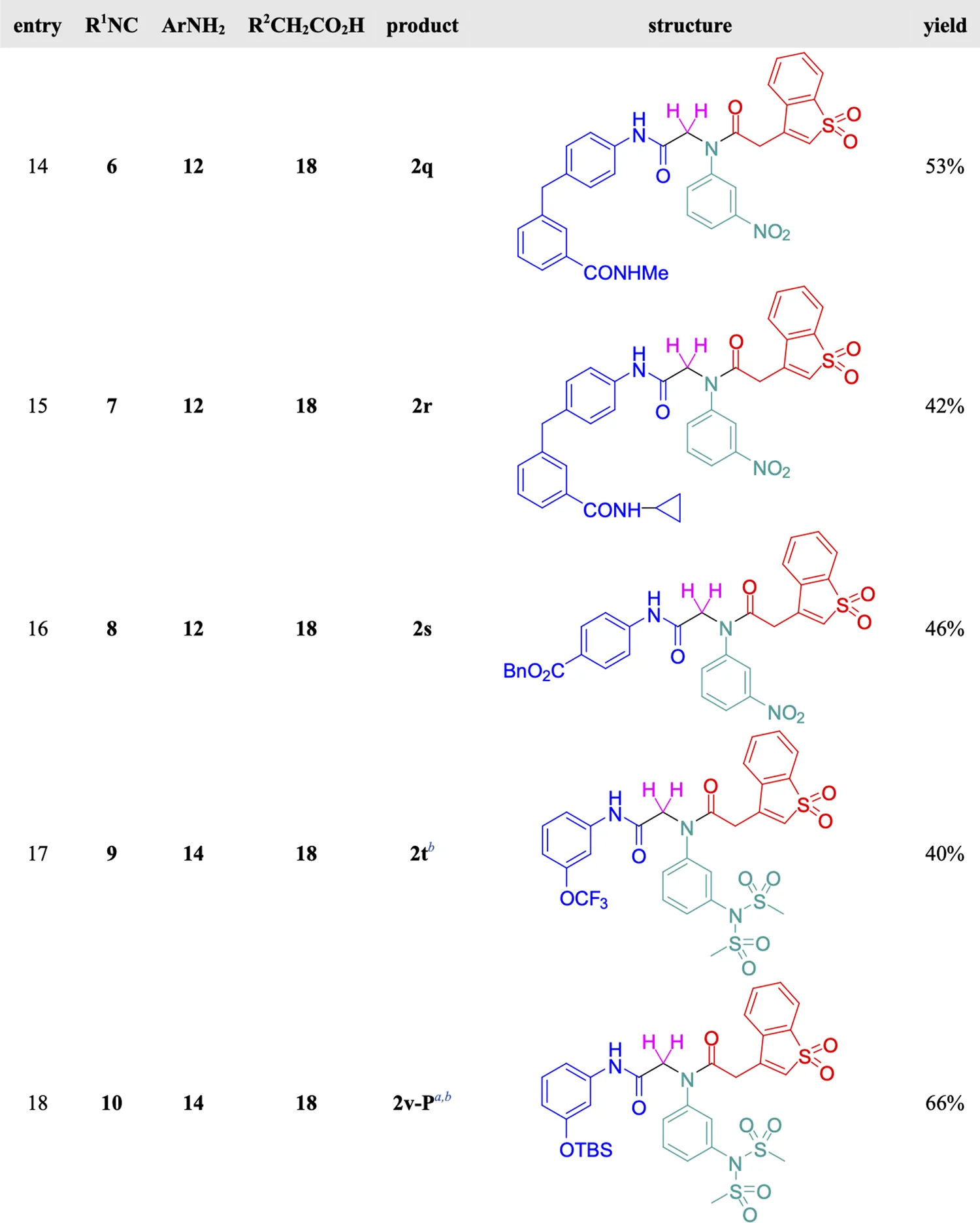
Ugi Reactions^a^ ^,^ b

a **2a-P, 2b-P, and 2v-P** (*P* = protected) were transformed into **2a, 2b, 2v, and 2w** (the latter two from the same precursor under different conditions) respectively, before submitting them to the biological tests (see Scheme 9).

b **2e, 2h 2j, 2t** (compounds tested also as such), were transformed into **2f, 2i, 2k, 2u** respectively, which were in turn subjected to testing (see Scheme 9).

It should be noted that Ugi products **2a-P**, **2b-P**, and **2v-P** (entries 1, 2, and 18) were deprotected (to give **2a**, **2b**, and **2v**) before submitting them to the biological tests, having one or more functional groups that we judged to be poorly suited if embedded in drug candidates. It is noteworthy that the hydrogenolysis of the benzyl groups worked well but, in the case of **2b-P**, the hydrogenation of the benzothiophene moiety occurred as well, and we only isolated **2b** with a dihydrothiophene group (Scheme 9).

**Scheme 9:**
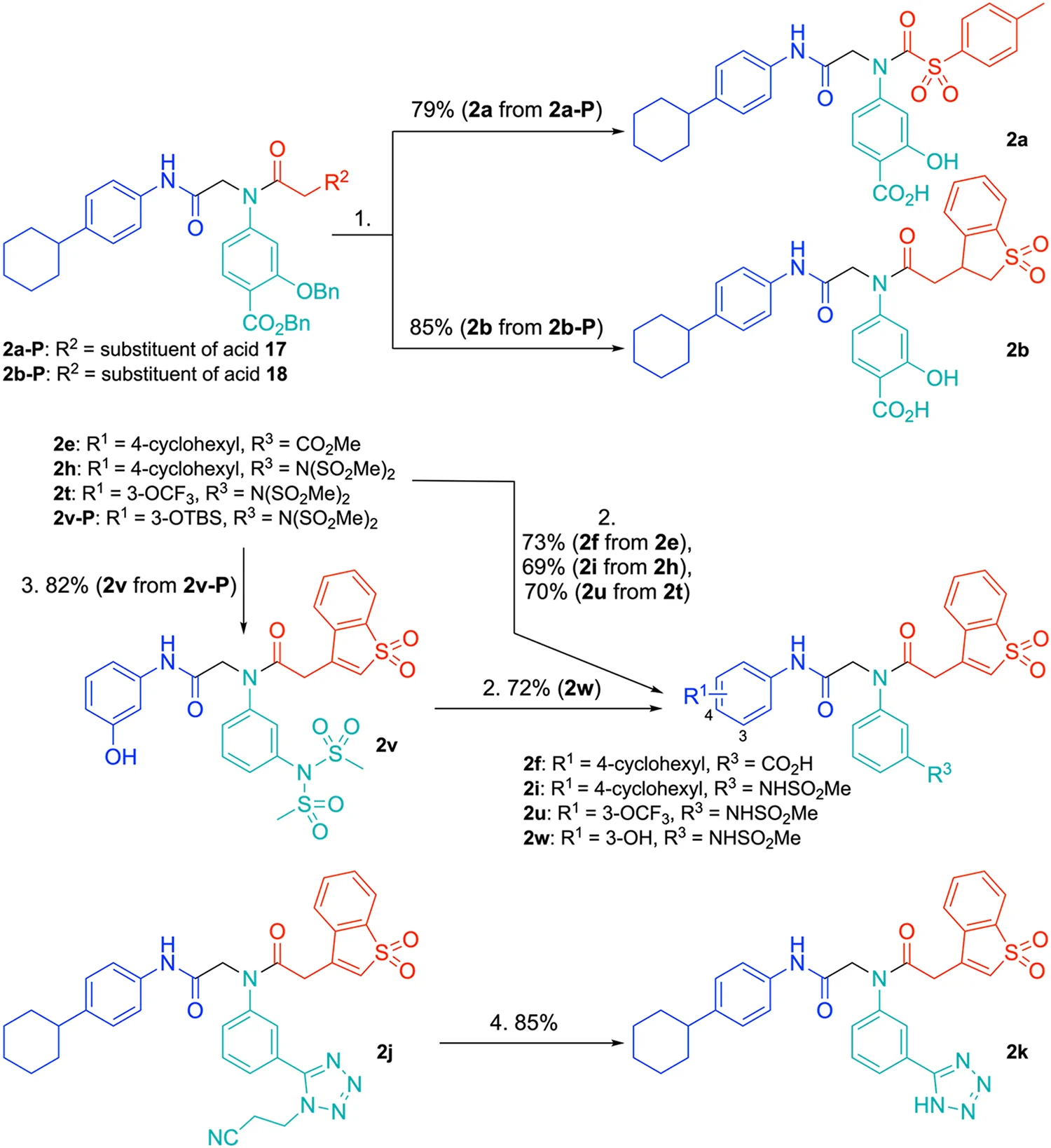
Transformation of Ugi Products into Other Compounds **2** ^a^

a Conditions: 1. H_2_, Pd/C 10%, MeOH/THF, 1:1, rt; 2. NaOH 2.5 M, the solvent was specifically chosen for each reaction (see Materials and Methods), rt; 3. *n*-TBAF, MeOH/MeCN 1:1, rt, 82%; 4. K_2_CO_3_ (1 M in H_2_O), THF:*t*-BuNH_2_ 1:1, rt.

On the other hand, compounds **2e**, **2h**, **2j**, **2t** (entries 5, 7, 8, and 17), which were tested as described below as well as **2v-P** (entry 18), have been further transformed into **2f**, **2i**, **2k**, **2u**, and **2w** to increase the number of molecules available for biological testing to a total of 23. The methyl ester of **2e** was hydrolyzed to the corresponding acid **2f**. The difficulties for obtaining the monosulfonylated derivative of **37** (Scheme 6) and its incompatibility with the Ugi reaction (formation of very complex reaction mixtures or no formation of the expected product), suggested to prepare monosulfonamides **2i**, **2u**, and **2w** by a controlled basic treatment of **2h**, **2t**, and from **2v** (after silyl ether removal of **2v-P**). Finally, unprotected tetrazole **2k**, was readily available from **2j** by a peculiar basic treatment, which removed the 2-cyanoethyl group from N-1 of tetrazole (Scheme 9).

All compounds **2a**–**w** have been fully characterized, and their purity was assessed by HPLC before testing, resulting >95%. It was necessary to use HPLC, because evaluation of purity by ^1^H NMR is troublesome. The spectra typically show broad signals due to conformational equilibrium between the two rotamers at the tertiary amide bond. Usually, this can be solved by recording the spectra at high temperature (up to 120 °C). However, some components of the library were unstable upon heating, so the spectra were almost always taken at room temperature. Nonetheless, the tested molecules are demonstrated to be remarkably stable after prolonged storage at −20 °C (also after a couple of years), displaying no loss of purity (HPLC).

## Evaluation of Antiproliferative Activity

All newly synthesized first-, and second-generation molecules underwent initial preliminary analysis to assess their antiproliferative activity against eight tumor cell lines of various histological origins, as described in the Materials and Methods.

The results of this preliminary analysis (Table 2) highlighted the prevalent lack of antiproliferative activity that met the a priori criteria for a molecule to be considered active in our antiproliferative assay, namely, an IC_50_ < 30 μM, that is, a percent inhibition lower than 50% at this concentration, for a 72 h assay.

**Table 2:** Results of the Preliminary Analysis of All Synthesized Anti-STAT3 Compounds

		**cell lines** ^a^							
entry^b^	compound	A2780	A549	MDA-MB-231	MDA-MB-453	BT474	SHSY5Y	IMR-32	SKMel-28
1	**SF-1–066** ^c^	>50^d^	>50	>50	>50	>50	>50	>50	>50
2	**2a**	>50	>50	>50	>50	>50	>50	>50	>50
3	**2b**	>50	>50	>50	>50	>50	>50	>50	>50
4	**2c**	>50	>50	>50	>50	>50	>50	>50	>50
5	**2d**	=50^e^	<50	>50	>50	>50	>50	<50	<50
6	**2l**	>50	>50	>50	>50	>50	>50	>50	>50
7	**2m**	>50	>50	>50	>50	>50	>50	>50	>50
8	**2g**	<50	<50	<50	<50	<50	<50	<50	=50
9	**2h**	<50	<50	<50	<50	<50	<50	<50	<50
10	**2i**	<50	>50	>50	>50	>50	>50	>50	>50
11	**2j**	>50	>50	>50	>50	>50	>50	>50	>50
12	**2k**	>50	>50	>50	>50	>50	>50	>50	>50
13	**2e**	>50	>50	>50	>50	>50	>50	>50	>50
14	**2f**	>50	>50	>50	>50	>50	>50	>50	>50
15	**2n**	>50	>50	>50	>50	>50	>50	>50	>50
16	**2o**	<50	<50	<50	<50	<50	<50	<50	<50
17	**2p**	>50	>50	>50	>50	>50	>50	>50	>50
18	**2q**	>50	>50	>50	>50	>50	>50	>50	>50
19	**2r**	>50	>50	>50	>50	>50	>50	>50	>50
20	**2s**	>50	>50	>50	>50	>50	>50	>50	>50
21	**2t**	>50	>50	>50	>50	>50	>50	>50	>50
22	**2u**	>50	>50	>50	>50	>50	>50	>50	>50
23	**2v**	>50	>50	>50	>50	>50	>50	>50	>50
24	**2w**	>50	>50	>50	>50	>50	>50	>50	>50

a Cell lines used for antiproliferative tests: A2780 (ovarian adenocarcinoma), A549 (lung carcinoma), MDA-MB-231, BT474, and MDA-MB-453 (breast carcinoma), SHSY5Y and IMR-32 (neuroblastoma), and SK-Mel-28 (melanoma).

b Entries 2–7 represent the first-generation, entries 8–24 the second-generation of molecules.

c Reference compound **SF-1–066** was synthesized as already reported^25^ (see Supporting Information).

d The data semiquantitatively express the percentage of cell proliferation after treatment with 30 μM of the test substances. This limit (30 μM) was arbitrarily chosen to define a pharmacologically significant antiproliferative activity in the 72 h MTT assay. The proliferation values < 50% (here expressed as a percentage) indicate probable IC_50_ values equal to or lower than 30 μM.

e The sign = 50 means that at 30 μM concentration, we have a high probability to find IC_50_ values of about 30 μM.

To validate these findings, a subset of compounds, randomly selected as representative examples of molecules exhibiting low antiproliferative activity without predefined selection criteria, was examined in greater depth by treating four tumor cell lines (A2780, A549, MDA-MB-231 e IMR32) with a range of concentrations (0.01–100 μM), which confirmed the preliminary results (Table 3).

**Table 3:** Confirmatory Analysis of the Antiproliferative Activity of Molecules **2i**, **2t**, **2u, 2v**, **2w** on Some Cell Lines

**cell lines** ^a^	**2i**	**2t**	**2u**	**2v**	**2w**
A2780	40.6 ± 6.0	>100	56.5 ± 10.9	>100	>100
A549	50.7 ± 0.7^b^	>100	46.2 ± 1.9	>100	>100
MDA-MB-231	51.5 ± 6.8	37.6 ± 14.6	46.6 ± 1.8	>100	>100
IMR32	ND	53.4 ± 21.9	51.0 ± 9.4	>100	>100

a The data show the IC_50_ values of the various compounds tested on tumor cell lines. Notably, each value aligns with the results from the preliminary screening at 30 μM.

Our preliminary analysis identified compound **2d**, one of the first-generation molecules, as exhibiting significant antiproliferative activity. Consequently, its chemical scaffold was selected as the reference structure for the design and verification, by molecular docking analysis, of all second-generation derivatives.

This preliminary screening demonstrated that, in addition to **2d**, compounds **2o**, **2g**, and **2h** also met the selectivity criteria described above. These active compounds were subjected not only to molecular docking studies to evaluate their predicted binding interactions with STAT3, but also to ligand efficiency metrics (LEM) analysis to assess their drug-likeness and overall suitability as lead compounds.

## Molecular Modeling and Docking Simulations

Molecular docking studies were performed to evaluate the predicted binding affinity of the synthesized compounds toward the STAT3 target, primarily based on their calculated binding energies. Although favorable binding to STAT3 was considered a necessary prerequisite for biological activity, it was not regarded as sufficient to predict antiproliferative efficacy. Therefore, the antiproliferative activity of the compounds was evaluated in parallel through biological assays, which provide a much more reliable assessment of their pharmacological potential.

Molecular docking analyses identified a ligand-binding region corresponding to a shallow yet well-defined cavity within the SH2 domain of hSTAT3, a structural element known to mediate receptor-dependent dimerization and subsequent transcriptional activation. This pocket, situated near the canonical pTyr705 recognition site, appears particularly suited to host small molecules able to interfere with STAT3 self-association or with phosphotyrosine-containing peptides.

The simulations revealed that a network of polar interactions is established between the docked ligands and key amino acid residues, including Lys591, Glu594, Arg595, Thr620, Lys626, Gln635, Ser636, Tyr657, and Thr714. These residues collectively contribute to hydrogen-bonding and electrostatic stabilization within the binding cleft. In parallel, hydrophobic residues such as Trp623, Ile634, Val637, Pro639, and Phe714 form a complementary nonpolar environment that helps orient and anchor the ligands, favoring a snug fit within the SH2 pocket. This dual contribution of polar and hydrophobic contacts underlies the overall stability of the predicted complexes and may reflect the physiological balance of interactions governing STAT3′s recruitment to phosphorylated partners.

Cluster analysis of the docking poses revealed several conformational families, suggesting alternative binding orientations and degrees of cavity penetration. Among these, candidates **2d, 2o**, and, in second choice, the molecule **2g** consistently exhibited the most favorable binding modes, displaying multiple hydrogen bonds and extensive van der Waals interactions with the residues lining the pocket. Their predicted binding energies (−9.88, −9.81, and −8.66 kcal/mol, respectively) indicate a high degree of complementarity between ligand and target surface. Candidate **2h** followed in terms of docking scores, with a binding energy of −8.41 kcal/mol. It is of note that **2h** is positioned slightly outside the binding site occupied by **2d**, and **2g**. It adopts a rotated conformation, nearly orthogonal to that of the other compounds, with the *N*-methylsulfonyl-methanesulfonamide group exposed toward the solvent.

On the other hand, **2o** is located well outside the binding pocket defined by **2d** and **2g**. It lies closer to Thr714 but loses key contacts with residues Glu594 and Ser636, while the nitrophenyl moiety remains solvent-exposed (Figure 1).

**Figure 1:**
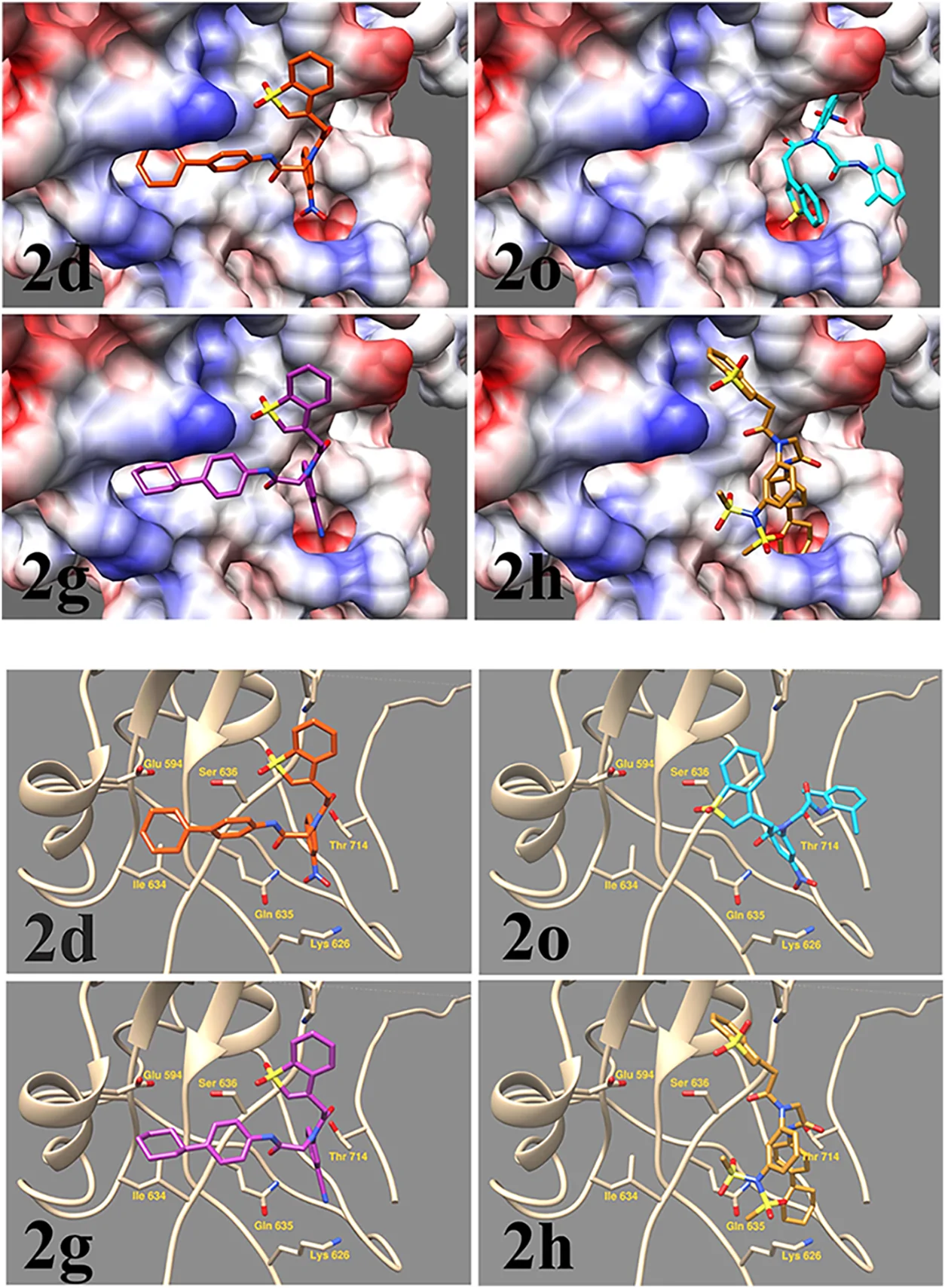
3D-structure of compounds **2d**, **2o**, **2g**, and **2h** bound to the SH2 domain of hSTAT3, as resulting from docking simulations.

## LEM Analysis

The ligand efficiency metrics (LEMs)^40,41^ of the most potent compounds (**2d**, **2o**, **2g** and **2h**), together with **SF-1–066** as a reference compound, have been analyzed through the AtlasCBS web tool (accessed in 2018 and now available as an optibrium facility; https://optibrium.com/resources/atlascbs-script-for-stardrop, accessed March 3, 2026), focusing our attention on the antiproliferative activity (IC_50_ values) registered in the A2780 ovarian cancer cell line. The results are reported in Figure 2. Starting from the consideration that compounds with high drug-likeness typically occupy the upper right quadrant of the plots, where both the efficiency-per-polarity (NSEI) and efficiency-per-size (NBEI) are optimized, we can state that the most potent compounds are also the most efficient ones and perform significantly better than the reference compound. In particular, **2o** performed as the most efficient compound. The same results were confirmed for both SKMeI28 and SHSY5Y cell lines (data not shown), thus indicating **2g**, **2o**, **2h**, **2i**, and **2d**, the best candidates for further studies. Interestingly, **2o** is the only congener in the series that would show moderate CNS penetrant potential (Percepta, ACD/Laboratories Release 2024.2.3).

**Figure 2:**
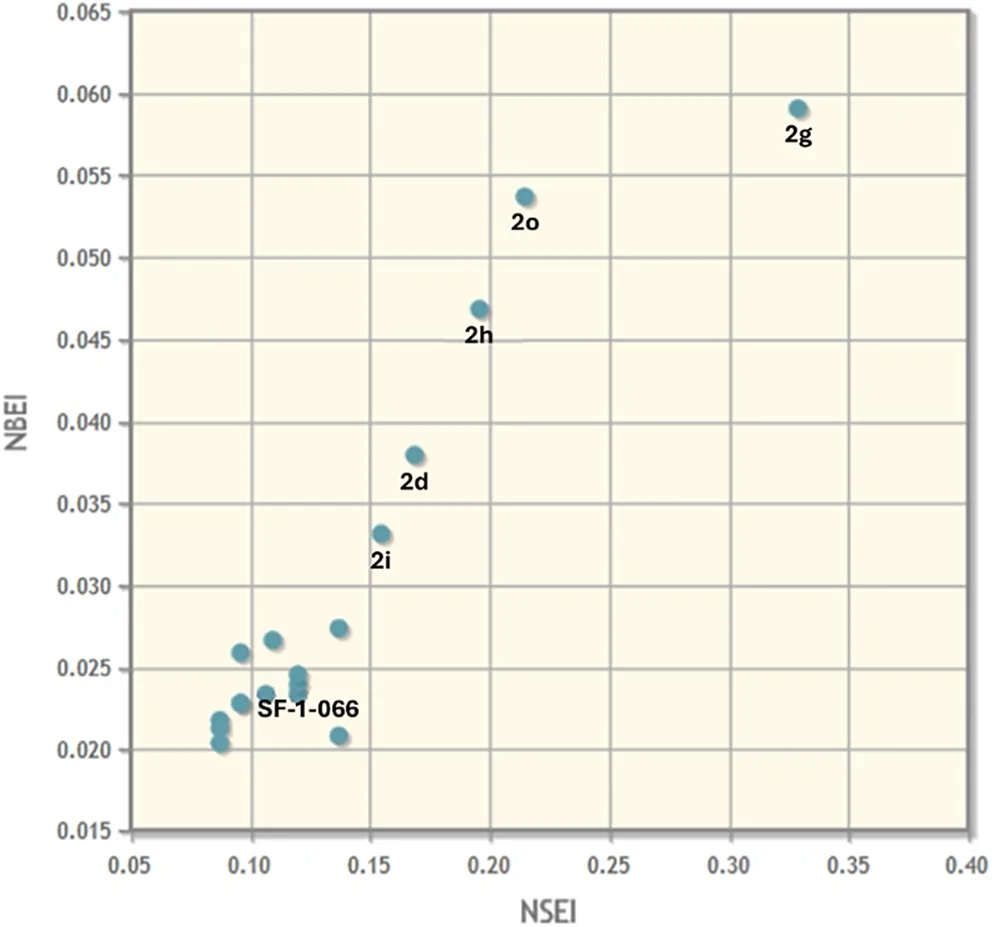
AtlasCBS ligand efficiency metric (LEM) analysis for **2d**, **2g**, **2h**, **2i, 2o**, and **SF-1–066** (reference compound). NSEI: efficiency-per-polarity index; NBEI: efficiency-per-size index.

## Antiproliferative Activity in STAT3 Overexpressing SKOV3 Cells

To confirm the effectiveness of STAT3 inhibition by **2d**, **2o**, **2g**, and **2h**, the SKOV3 ovarian cancer cell line was employed. Upon stimulation with the recombinant human IL-6Rα/IL-6 chimeric protein, these cells exhibit increased activated STAT3 levels, potentially enhancing their sensitivity to compounds targeting this transcription factor. Under basal conditions and following activation with recombinant IL-6, the IC_50_ values of compounds **2o** and **2g** decreased from 10.5 ± 4.4 to 2.8 ± 2.0 μM and from 2.9 ± 1.2 to 1.3 ± 0.4 μM, respectively, indicating a significant enhancement of antiproliferative activity. In contrast, the parent compound **SF-1–066** did not show a significant increase in activity under either condition, with IC_50_ values consistently exceeding 100 μM. For compound **2d**, the slight difference found between the two experimental conditions (4.3 ± 0.8 vs 3.9 ± 1.0 μM) was not statistically significant. A similar pattern was observed for compound **2h** whose IC_50_ values were identical under both experimental conditions (4.7 ± 0.5 vs 4.5 ± 0.2 μM).

## Conclusions

In conclusion a library of 23 new molecules (first- and second-generation) was prepared and tested as possible STAT3 inhibitors through a diversity oriented and highly convergent approach, taking advantage from the well-established Ugi multicomponent reaction. The diversity of the molecules depends on the decorations of the Ugi inputs, which can be modified quite easily according to the results of the biological tests or as suggested by docking experiments. The antiproliferative activity of the synthesized compounds was assessed through a two-step biological evaluation, leading to the identification of four derivatives with significant pharmacological activity. Nevertheless, experiments performed in SKOV3 cells stimulated with a recombinant human IL-6Rα/IL-6 chimeric protein demonstrated that the antiproliferative effects of compounds **2o** and **2g**, and to a lesser extent **2d**, were dependent on STAT3 signaling, in agreement with the binding mode predicted by molecular docking studies. These observations were further corroborated by LEM analysis of the active derivatives. Taken together, the biological findings support the ability of the selected compounds to modulate STAT3 activity, confirming target engagement and validating STAT3 as the molecular target underlying the observed pharmacological effects. These results are consistent with the rational design strategy adopted for this series of compounds. Based on the results obtained, further structural modifications to the molecules could be designed, while also considering strategies to improve their solubility and bioavailability. Moreover, an optimization of the synthetic procedures, especially for the large-scale production of the isocyanides through greener methodologies could represent an important future development. Furthermore, from the perspective of pharmacological activity assessment, compounds identified as active in the preliminary studies described herein will undergo further in vitro and in vivo evaluation to comprehensively characterize their pharmacological profile and validate their biological activity.

## Materials and Methods

### Chemistry

#### General Experimental Details

All chemicals (including dry solvents) used for the synthesis of all compounds were purchased from commercial suppliers and used as received. NMR spectra were recorded on Varian (300 MHz ^1^H and 75 MHz ^13^C) or on Jeol JNM-ECZ400R (400 MHz ^1^H and 101 MHz ^13^C) instruments in CDCl_3_, or DMSO-*d*_6_, or MeOH-*d*_4_ or MeCN-*d*_3_, using TMS as internal standard for CDCl_3_ spectra. If not otherwise specified the spectra have been recorded at 25 °C. Chemical shifts are reported in ppm (δ scale) using tetramethylsilane (TMS, 0.00 ppm) or the residual solvent signal (CDCl_3_ at 77.16 ppm for ^13^C) as internal standards. The data are reported as follows: chemical shift, integration, multiplicity (indicated as s, singlet; d, doublet; t, triplet; q, quartet; m, multiplet; and combinations thereof), coupling constants (*J*) in Hertz (Hz), and assignment (when possible). Peak assignments were performed with the aid of gCOSY (gradient correlation spectroscopy), gHSQC (gradient heteronuclear single-quantum correlation spectroscopy), and gHMBC (gradient heteronuclear multiple-bond correlation spectroscopy). In some compounds a mixture of conformers can be identified; therefore, different signals, that can be attributed to the rotamers, are identified as **A** (= major) and **B** (= minor). Unless otherwise indicated spectra were recorded at rt. All NMR spectra were analyzed using the software MestReNova (Santiago de Compostela, Spain) (Mestrelab Research v. 14.2). FTIR (Fourier transform infrared) spectra were recorded on a PerkinElmer Spectrum 65 instrument on solid, oil or foamy samples with ATR (attenuated total reflectance) technique. Melting points were determined with an electrothermal apparatus Büchi B-535 with a 1 °C/min thermal program, and are uncorrected. HPLC analyses were performed on Agilent HP 1100 instrument equipped with a VWD detector at 220 nm. Columns and conditions are reported for each tested compound. HRMS analyses were recorded on a Waters UPLC Acquity system coupled to a Synapt G2 QToF mass spectrometer, and acquired from 50 to 1200 *m*/*z* in both ESI positive and ESI negative ionization modes. Three Å molecular sieves (MS) were activated by heating at 250 °C for 24 h. TLC analyses were carried out on silica gel plates, which were developed by these detection methods: (**A**) U.V.; (**B**) dipping into a solution of (NH_4_)_4_MoO_4_·4H_2_O (21 g) and Ce­(SO_4_)_2_·4H_2_O (1 g) in H_2_SO_4_ (31 mL) and H_2_O (469 mL) and warming; (**C**) dipping into a solution of ninhydrin (900 mg), *n*-butanol (300 mL) and AcOH (9 mL) and warming; (**D**) 2% KMnO_4_ in H_2_O. Chromatographies were carried out on 220–400 mesh silica gel using the “flash” methodology. In extractive workup, aqueous solutions were always reextracted thrice with the appropriate organic solvent. Organic extracts were washed with brine, dried over Na_2_SO_4_ and filtered, before evaporation of the solvent under reduced pressure. All reactions employing dry solvents were carried out under a nitrogen atmosphere or under ultra pure argon (only the use of argon is specified).

### Ugi Products (Table 1)

#### General Procedure for the Ugi Reactions

(1) Preformation of the imine: aniline (**11–16**, 1.3 equiv), paraformaldehyde (**48**, 1.3 equiv) and 3 Å MS (50 mg/mmol) were placed in a flask backfilled with nitrogen. After addition of 1:1 EtOH/TFE (0.2 M), the resulting suspension was stirred at 70 °C for 16 h; (2) Ugi reaction: the mixture was cooled at 50 °C and isocyanide (**3–10**, 1.3 equiv) and carboxylic acid (**17** or **18**, 1.0 equiv) were added. If necessary additional 1:1 EtOH/TFE (0.2 M) was added, then the mixture was stirred at 50 °C for 24 h. Afterward, the suspension was diluted in 1:1 MeOH/CH_2_Cl_2_ (∼2.5 mM) and filtered through a Celite pad. The solution was concentrated to dryness, and the residue was purified as described for each compound.

#### Benzyl 2-(Benzyloxy)-4-(*N*-(2-((4-cyclohexylphenyl)­amino)-2-oxoethyl)-2-(*N*,4-dimethylphenylsulfonamido)­acetamido)­benzoate **2a-P**

The reaction was performed following the general procedure starting from 97 mg of **17** (with **3**, **11** and **48**), affording 266 mg of **2a-P** after chromatography with PE/EtOAc 1:1 as an ivory solid. Yield 86%. *R*_f_ 0.41 (PE/EtOAc 6:4, A, B). Mp 101.0–103.5 °C. ^1^H NMR (300 MHz, CDCl_3_): δ 8.34 (s, 1H, NH), 7.90 (d, *J* = 8.2 Hz, 1H, 6-CH salicylic ring), 7.58 (d, *J* = 8.3 Hz, 2H, 2 × CH para syst.), 7.48 (d, *J* = 8.5 Hz, 2H, 2 × CH para syst.), 7.43–7.28 (m, 7H, 5 × CH of Ph + 2 × CH para syst.), 7.25–7.19 (m, 5H, 5 × CH of Ph), 7.14 (d, *J* = 8.5 Hz, 2H, 2 × CH para syst.), 7.10 (d, *J* = 1.7 Hz, 1H, 3-CH salicylic ring), 6.97 (dd, *J* = 8.2, 1.9 Hz, 1H, arom. 5-CH salicylic ring), 5.37 (s, 2H, OCH_2_), 5.17 (s, 2H, OCH_2_), 4.38 (s, 2H, CH_2_), 3.63 (s, 2H, CH_2_), 2.67 (s, 3H, NCH_3_), 2.52–2.40 (m, 1H, CH *c*-Hex), 2.36 (s, 3H, CH_3_ tosyl ring), 1.87–1.68 (m, 5H, 5 × C*H*H *c*-Hex), 1.46–1.29 (m, 5H, 5 × CH*H* *c*-Hex). ^13^C NMR (75 MHz, CDCl_3_) [two carbons of Ph overlap]; δ 168.8 (C=O), 166.1 (C=O), 165.5 (C=O), 159.2 (C–O), 146.0 (C quat. Ar), 144.6 (C quat. Ar), 143.9 (C quat. Ar), 135.93 (C quat. Ar), 135.91 (C quat. Ar), 135.5 (C quat. Ar), 134.5 (C quat. Ar), 133.5 (6-CH salicylic ring), 129.8 (CH of Ph), 128.73 (CH of Ph), 128.71 (CH of Ph), 128.4 (CH of Ph), 128.2 (CH of Ph), 127.7 (2 × CH para syst.), 127.37 (2 × CH para syst.), 127.35 (2 × CH para syst.), 121.3 (*C*–CO_2_Bn), 120.3 (2 × CH para syst.), 119.3 (5-CH salicylic ring), 113.5 (3-CH salicylic ring), 71.0 (OCH_2_), 67.2 (OCH_2_), 54.8 (CH_2_), 51.9 (CH_2_), 44.2 (CH *c*-Hex), 36.7 (NCH_3_), 34.6 (2 × CH_2_ *c*-Hex), 27.0 (2 × CH_2_ *c*-Hex), 26.3 (CH_2_ *c*-Hex), 21.6 (CH_3_ tosyl ring). IR (neat): *ν*_max_ (cm^–1^) 3329, 2924, 2851, 1678, 1600, 1517, 1416, 1286, 1236, 1160, 1089, 1001, 936, 816, 777, 731, 696, 652.

#### Benzyl 2-(Benzyloxy)-4-(*N*-(2-((4-cyclohexylphenyl)­amino)-2-oxoethyl)-2-(1,1-dioxidobenzo­[*b*]­thiophen-3-yl)­acetamido)­benzoate **2b-P**

The reaction was performed following the general procedure starting from 103 mg of **18** (with **3**, **11** and **48**), affording 259 mg of **2b-P** after chromatography with PE/EtOAc 6:4 as an off-white solid. Yield 75%. *R*_f_ 0.30 (PE/EtOAc 1:1, A, B). Mp 176.0–177.0 °C (dec.). IR (neat): ^1^H NMR (300 MHz, CDCl_3_): δ 8.38 (s, 1H, NH), 7.90 (d, *J* = 8.2 Hz, 1H, 6-CH salicylic ring), 7.81–7.72 (m, 1H, CH Ar), 7.62–7.46 (m, 2H, 2 × CH Ar), 7.43–7.28 (m, 10H, 8 × CH Ar + 2 × CH para syst.), 7.26–7.14 (m, 4H, 3 × CH Ar + 3-CH salicylic ring), 7.10 (d, *J* = 8.5 Hz, 2H, 2 × CH para syst.), 6.97 (dd, *J* = 8.2, 1.9 Hz, 1H, arom. 5-CH salicylic ring), 6.46 (t, *J* = 2.2 Hz, 1H, CH-SO_2_), 5.36 (s, 2H, OCH_2_), 5.13 (s, 2H, OCH_2_), 4.75 (d, *J* = 2.2 Hz, 2H, C*H*_2_C=CH), 4.45 (s, 2H, NCH_2_C=O), 2.51–2.33 (m, 1H, CH *c*-Hex), 1.86–1.68 (m, 5H, 5 × C*H*H *c*-Hex), 1.41–1.24 (m, 5H, 5 × CH*H* *c*-Hex). ^13^C NMR (75 MHz, CDCl_3_) δ 166.2 (C=O), 165.6 (C=O), 165.1 (C=O), 159.2 (C-O), 146.7 (C-N), 144.6 (C-N), 140.3 (C quat. Ar), 139.9 (C quat. Ar), 135.8 (C quat. Ar), 135.8 (C quat. Ar), 135.3 (C quat. Ar), 134.4 (C quat. Ar), 133.8 (CH Ar), 133.3 (6-CH salicylic ring), 132.6 (CH Ar), 128.7 and 128.6 (CH Ar and 2 × CH para syst.), 128.41 (CH Ar), 128.38 (CH Ar), 128.1 (CH Ar), 127.4 (2 × CH para syst.), 122.9 (CH Ar), 121.8 (CH Ar), 120.7 (*C*-CO_2_Bn), 120.1 (CH Ar), 119.4 (5-CH salicylic ring), 114.2 (CH-SO_2_), 113.1 (CH Ar), 70.9 (OCH_2_), 67.2 (OCH_2_), 55.6 (*C*H_2_C=CH), 54.7 (N*C*H_2_C=O), 44.1 (CH *c*-Hex), 34.6 (2 × CH_2_ *c*-Hex), 26.9 (2 × CH_2_ *c*-Hex), 26.2 (CH_2_ *c*-Hex).*ν*_max_ (cm^–1^) 3341, 2924, 1730, 1682, 1652, 1597, 1542, 1514, 1468, 1417, 1379, 1296, 1227, 1149, 1124, 1064, 1002, 954, 864, 828, 765, 737, 693.

#### *N*-(2-((4-Cyclohexylphenyl)­amino)-2-oxoethyl)-2-(*N*,4-dimethylphenylsulfonamido)-*N*-(3-nitrophenyl)­acetamide **2c**

The reaction was performed following the general procedure starting from 49 mg of **17** (with **3**, **12** and **48**), affording 92 mg of **2c** after chromatography with PE/EtOAc 6:4 as a yellow solid. Yield 79%. *R*_f_ 0.14 (PE/EtOAc 1:1, **A**, **B**). Mp 179.1–179.8 °C. HPLC purity 100%, *R*_t_ 14.05 min (Gemini C18 150 mm × 3 mm, 5 μm column, A: H_2_O/MeCN 1:1 + 0.1% FA to B: MeCN + 0.1% FA in 20 min, flow 0.5 mL/min, temp. 24 °C). ^1^H NMR (300 MHz, DMSO-*d*_6_): δ 9.77 (s, 1H, NH), 8.33 (t, *J* = 2.1 Hz, 1H, 2-CH nitrophenyl ring), 8.20 (ddd, *J* = 8.2, 2.2, 0.9 Hz, 1H, 4-CH nitrophenyl ring), 7.91 (d, *J* = 8.0 Hz, 1H, 6-CH nitrophenyl ring), 7.74 (t, *J* = 8.1 Hz, 1H, 5-CH nitrophenyl ring), 7.59 (d, *J* = 8.3 Hz, 2H, 2 × CH para syst.), 7.46 (d, *J* = 8.5 Hz, 2H, 2 × CH para syst.), 7.32 (d, *J* = 7.9 Hz, 2H, 2 × CH para syst.), 7.16 (d, *J* = 8.5 Hz, 2H, 2 × CH para syst.), 4.49 (s, 2H, CH_2_), 3.92 (s, 2H, CH_2_), 2.76 (s, 3H, NCH_3_), 2.48–2.42 (m, 1H, CH *c*-Hex), 2.35 (s, 3H, CH_3_ tosyl ring), 1.89–1.63 (m, 5H, 5 × C*H*H *c*-Hex), 1.49–1.25 (m, 5H, 5 × CH*H* *c*-Hex). ^13^C NMR (75 MHz, DMSO-*d*_6_): δ 166.8 (C=O), 165.7 (C=O), 148.1 (C quat. Ar), 142.7 (C quat. Ar), 142.6 (2 × C quat. Ar), 135.9 (C quat. Ar), 134.9 (C quat. Ar), 134.0 (6-CH nitrophenyl ring), 130.2 (5-CH nitrophenyl ring), 129.0 (2 × CH para syst.), 126.6 (2 × CH para syst.), 126.2 (2 × CH para syst.), 122.4 (2-CH nitrophenyl ring), 122.0 (4-CH nitrophenyl ring), 119.3 (2 × CH para syst.), 52.5 (CH_2_), 50.8 (CH_2_), 42.7 (CH *c*-Hex), 34.9 (NCH_3_), 33.6 (2 × CH_2_ *c*-Hex), 25.9 (2 × CH_2_ *c*-Hex), 25.2 (CH_2_ *c*-Hex), 20.4 (CH_3_ tosyl ring). IR (neat): *ν*_max_ (cm^–1^) 3303, 3283, 2925, 2853, 2299, 1661, 1608, 1529, 1415, 1349, 1162, 1088, 1007, 938, 808, 695. HRMS (ESI^+^) *m*/*z*: [M + H]^+^ Calcd for C_30_H_35_N_4_O_6_S 579.2272; Found 579.2278.

#### *N*-(2-((4-Cyclohexylphenyl)­amino)-2-oxoethyl)-2-(1,1-dioxidobenzo­[*b*]­thiophen-3-yl)-*N*-(3-nitrophenyl)­acetamide **2d**

The reaction was performed following the general procedure starting from 49 mg of **18** (with **3**, **12** and **48**), affording 82 mg of **2d** after a double trituration with Et_2_O/CH_2_Cl_2_/MeOH 7:3:0.5 as a pale yellow solid with HPLC purity 92%. Yield 67%. *R*_f_ 0.48 (PE/EtOAc 4:6, **A**, **B**). Mp 207.4–208.4 °C. After further trituration with CH_2_Cl_2_/MeOH 1:1 and twice with Et_2_O/CH_2_Cl_2_/MeOH 7:3:0.5 a white solid was obtained and the HPLC purity was improved up to 98%, *R*_t_ 18.11 min (Phenyl C6 150 mm × 3 mm, 3 μm column, A: H_2_O/MeCN 7:3 to B: MeCN in 20 min, flow 0.34 mL/min, temp. 25 °C). ^1^H NMR (300 MHz, DMSO-*d*_6_) [2 conformers are present]: δ 10.17 (s, 1H, NH), 8.38 (s, 1H, CH Ar), 8.27–8.15 (m, 1H, CH Ar), 7.95–7.88 (m, 2H, 2 × CH Ar), 7.83–7.67 (m, 4H, 4 × CH Ar), 7.50 (d, *J* = 8.1 Hz, 2H, 2 × CH para syst.), 7.15 (d, *J* = 8.4 Hz, 2H, 2 × CH para syst.), 6.87 (s, 0.5H, A CH–SO_2_), 6.72 (s, 0.5H, B CH–SO_2_), 4.75–4.57 (m, 4H, 2 × CH_2_), 2.44 (s, 1H, CH *c*-Hex), 1.84–1.64 (m, 5H, 5 × C*H*H *c*-Hex), 1.45–1.08 (m, 5H, 5 × CH*H* *c*-Hex). ^13^C NMR (75 MHz, DMSO-*d*_6_) [2 conformers are present and therefore there are extra signals]: δ 166.4 (A C=O), 166.3 (B C=O), 164.4 (C=O), 148.3 (C quat. Ar), 143.4 (C quat. Ar), 142.8 (C quat. Ar), 139.9 (C quat. Ar), 139.6 (C quat. Ar), 138.1 (C quat. Ar), 136.5 (C quat. Ar), 134.3 (CH Ar), 134.1 (CH Ar), 134.1 (CH Ar), 132.7 (CH Ar), 132.6 (CH Ar), 130.6 (CH Ar), 126.8 (2 × CH para syst.), 123.8, 123.6 (CH Ar), 122.5 (CH Ar), 122.3 (CH Ar), 121.3 (CH Ar), 119.2 (2 × CH para syst.), 117.4 (A CH–SO_2_), 115.9 (B CH–SO_2_), 54.7 (*C*H_2_C=CH), 52.9 (N*C*H_2_C=O), 43.2 (CH *c*-Hex), 34.0 (2 × CH_2_ *c*-Hex), 26.3 (2 × CH_2_ *c*-Hex), 25.6 (CH_2_ *c*-Hex). IR (neat): *ν*_max_ (cm^–1^) 3312, 2992, 2923, 2847, 1684, 1657, 1622, 1602, 1526, 1480, 1466, 1449, 1418, 1388, 1348, 1296, 1258, 1206, 1148, 1121, 1067, 1047, 1015, 965, 885, 863, 846, 823, 803, 783, 765, 740, 713, 694, 687, 668, 640, 620. HRMS (ESI^+^) *m*/*z*: [M + H]^+^ Calcd for C_30_H_30_N_3_O_6_S 560.1850; Found 560.1852.

#### Methyl 3-(*N*-(2-((4-Cyclohexylphenyl)­amino)-2-oxoethyl)-2-(1,1-dioxidobenzo­[*b*]­thiophen-3-yl)­acetamido)­benzoate **2e**

The reaction was performed following the general procedure starting from 78 mg of **18** (with **3**, **16** and **48**), affording 185 mg of **2e** after chromatography with PE/EtOAc/CH_2_Cl_2_/MeOH 4:1:1:0.5 as a white solid. Yield 93%. *R*_f_ 0.37 (PE/CH_2_Cl_2_/EtOAc/MeOH 4:1:1:0.5, **A**). Mp 242.4–243.4 °C (dec.). HPLC purity 99%, *R*_t_ 12.24 min (Gemini C18 150 mm × 3 mm, 5 μm column, A: H_2_O/MeCN 1:1 + 0.1% FA to B: MeCN + 0.1% FA in 20 min, flow 0.5 mL/min, temp. 26 °C). ^1^H NMR (300 MHz, DMSO-*d*_6_): δ 10.10 (s, 1H, NH), 8.07 (s, 1H, CH Ar), 7.97 (d, *J* = 7.1 Hz, 1H, CH Ar), 7.89 (d, *J* = 9.7 Hz, 1H, CH Ar), 7.68 (s, 4H, 4 × CH Ar), 7.62 (t, *J* = 7.9 Hz, 1H, CH Ar), 7.49 (d, *J* = 8.5 Hz, 2H, 2 × CH para syst.), 7.15 (d, *J* = 8.5 Hz, 2H, 2 × CH para syst.), 6.63 (s, 1H, CH–SO_2_), 4.72 (s, 2H, C*H_2_* C=CH), 4.55 (s, 2H, NCH_2_C=O), 3.85 (s, 3H, OCH_3_), 2.45–2.33 (m, 1H, CH *c*-Hex, partially buried by DMSO), 1.89–1.63 (m, 5H, 5 × C*H*H *c*-Hex), 1.48–1.10 (m, 5H, 5 × CH*H* *c*-Hex). ^13^C NMR (75 MHz, DMSO-*d*_6_): δ 166.4 (C=O), 165.6 (C=O), 164.4 (C=O), 142.8 (C quat. Ar), 142.7 (C quat. Ar), 139.6 (C quat. Ar), 137.6 (C quat. Ar), 136.6 (C quat. Ar), 134.3 (C quat. Ar), 134.2 (CH Ar), 132.8 (CH Ar), 132.6 (CH Ar), 131.0 (C quat. Ar), 130.0 (CH Ar), 128.5 (CH Ar), 128.0 (CH Ar), 126.9 (2 × CH para syst.), 123.3 (CH Ar), 121.4 (CH Ar), 119.2 (2 × CH para syst.), 115.9 (CH–SO_2_), 54.7 (*C*H_2_C=CH), 52.9 (N*C*H_2_C=O), 52.4 (OCH_3_), 43.2 (CH *c*-Hex), 34.1 (2 × CH_2_ *c*-Hex), 26.4 (2 × CH_2_ *c*-Hex), 25.6 (CH_2_ *c*-Hex). IR (neat): *ν*_max_ (cm^–1^) 3303, 3001, 2921, 2849, 1721, 1678, 1656, 1620, 1529, 1483, 1467, 1447, 1419, 1382, 1350, 1305, 1286, 1260, 1210, 1150, 1117, 1082, 1049, 1016, 984, 964, 922, 857, 819, 799, 783, 763, 710, 697, 644. HRMS (ESI^+^) *m*/*z*: [M + H]^+^ Calcd for C_32_H_33_N_2_O_6_S 573.2054; Found 573.2071.

#### *N*-(3-Cyanophenyl)-*N*-(2-((4-cyclohexylphenyl)­amino)-2-oxoethyl)-2-(1,1-dioxidobenzo­[*b*]­thiophen-3-yl)­acetamide **2g**

The reaction was performed following the general procedure starting from 200 mg of **18** (with **3**, **13** and **48**), affording 428 mg of **2g** after trituration with CH_2_Cl_2_/MeOH 1:1 as a pale yellow solid with HPLC purity 92%. Yield 89%. R_*f*_ 0.43 (PE/EtOAc 4:6, **A**, **B**). Mp 183.2–184.9 °C. After further two triturations with Et_2_O/CH_2_Cl_2_/MeOH 7:3:0.5 a white solid was obtained and the HPLC purity was improved up to 98%, *R*_t_ 17.68 min (Phenyl C6 150 mm × 3 mm, 3 μm column, A: H_2_O/MeCN 7:3 to B: MeCN in 20 min, flow 0.34 mL/min, temp. 25 °C). ^1^H NMR (300 MHz, DMSO-*d*_6_): δ 10.14 (s, 1H, NH), 7.99 (s, 1H, CH Ar), 7.94–7.62 (m, 7H, 7 × CH Ar), 7.49 (d, *J* = 8.5 Hz, 2H, 2 × CH para syst.), 7.15 (d, *J* = 8.5 Hz, 2H, 2 × CH para syst.), 6.68 (s, 1H, CH–SO_2_), 4.71 (d, *J* = 1.8 Hz, 2H, C*H_2_* C=CH), 4.58 (s, 2H, NCH_2_C=O), 2.47–2.35 (m, 1H, CH *c*-Hex), 1.84–1.65 (m, 5H, 5 × C*H*H *c*-Hex), 1.48–1.19 (m, 5H, 5 × CH*H c*-Hex). ^13^C NMR (75 MHz, DMSO-*d*_6_): δ 166.4 (C=O), 164.5 (C=O), 143.2 (C quat. Ar), 142.8 (C quat. Ar), 139.6 (C quat. Ar), 137.9 (C quat. Ar), 136.5 (C quat. Ar), 134.3 (CH Ar), 134.1 (CH Ar), 132.8 (CH Ar), 132.6 (CH Ar), 131.4 (CH Ar), 130.9 (CH Ar), 130.7 (CH Ar), 126.9 (2 × CH para syst.), 123.5 (C quat. Ar), 121.3 (CN), 119.2 (2 × CH para syst.), 118.3 (C quat. Ar), 116.0 (CH–SO_2_), 112.3 (*C*CN Ar), 54.6 (*C*H_2_C=CH), 52.7 (N*C*H_2_C=O), 43.2 (CH *c*-Hex), 34.1 (2 × CH_2_ *c*-Hex), 26.4 (2 × CH_2_ *c*-Hex), 25.6 (CH_2_ *c*-Hex). IR (neat): *ν*_max_ (cm^–1^) 3248, 3076, 2923, 2849, 2229, 1670, 1655, 1618, 1599, 1580, 1541, 1515, 1486, 1473, 1448, 1417, 1389, 1348, 1305, 1286, 1259, 1223, 1207, 1150, 1118, 1085, 1052, 1015, 999, 971, 931, 916, 873, 830, 818, 798, 786, 765, 704, 693, 668, 651, 610. HRMS (ESI^+^) *m*/*z*: [M + H]^+^ Calcd for 540.1952; Found 540.1959.

#### *N*-(2-((4-Cyclohexylphenyl)­amino)-2-oxoethyl)-2-(1,1-dioxidobenzo­[*b*]­thiophen-3-yl)-*N*-(3-(*N*-(methylsulfonyl)­methylsulfonamido)­phenyl)­acetamide **2h**

The reaction was performed following the general procedure starting from 96 mg of **18** (with **3**, **14** and **48**), affording 253 mg of **2h** after trituration with Et_2_O as an off-white solid with HPLC purity 94%. Yield 86%. *R*_f_ 0.48 (PE/EtOAc 4:6, A, B). Mp 183.8–185.7 °C. After further purification by chromatography (PE/EtOAc 4:6) a white solid was obtained and the HPLC purity was improved up to 97%, *R*_t_ 19.90 min (ACE Excel 3 C18-AR 150 mm × 3 mm, 3 μm column, A: H_2_O/MeCN 9:1 + 0.1% FA to B: MeCN + 0.1% FA in 20 min, flow 0.38 mL/min, temp. 30 °C. ^1^H NMR (300 MHz, DMSO-*d*_6_): δ 10.13 (s, 1H, NH), 7.93–7.84 (m, 1H, CH Ar), 7.78–7.56 (m, 7H, CH Ar), 7.50 (d, *J* = 8.4 Hz, 2H, para system CH Ar), 7.16 (d, *J* = 8.5 Hz, 2H, para system CH Ar), 6.62 (s, 1H, CH–SO_2_), 4.72 (d, *J* = 1.8 Hz, 2H, C*H*_*2*_C=CH), 4.58 (s, 2H, NCH_2_C=O), 3.50 (s, 6H, 2 × CH_3_), 2.47–2.36 (m, 1H, CH *c*-Hex), 1.81–1.65 (m, 5H, 5 × C*H*H *c*-Hex), 1.45–1.22 (m, 5H, 5 × CH*Hc*-Hex). ^13^C NMR (75 MHz, DMSO-*d*_6_): δ 166.4 (C=O), 164.3 (C=O), 143.3 (Cq. Ar), 142.8 (Cq. Ar), 139.6 (Cq. Ar), 137.9 (Cq. Ar), 136.6 (Cq. Ar), 134.3 (Cq. Ar), 134.1 (Cq. Ar), 134.0 (CH Ar), 132.7 (CH Ar), 130.6 (CH Ar), 130.5 (CH Ar), 130.3 (CH Ar), 128.7 (CH Ar), 126.9 (para system CH Ar), 123.0 (CH Ar), 121.4 (CH Ar)), 119.3 (para system CH Ar), 115.2 (CH–SO_2_), 54.7 (*C*H_2_C=CH), 52.9 (N*C*H_2_C=O), 43.2 (CH *c*-Hex), 43.0 (2 × CH_3_), 34.1 (2 × CH_2_ *c*-Hex), 26.4 (2 × CH_2_ *c*-Hex), 25.6 (CH_2_ *c*-Hex). IR (neat): *ν*_max_ (cm^–1^) 3246, 3041, 2925, 2850, 1655, 1597, 1539, 1517, 1487, 1468, 1450, 1415, 1367, 1350, 1303, 1257, 1228, 1206, 1160, 1150, 1124, 1088, 1052, 1018, 1007, 977, 963, 935, 869, 828, 758, 699, 643, 604. HRMS (ESI^+^) *m*/*z*: [M + H]^+^ Calcd for C_32_H_36_N_3_O_8_S_3_ 686.1659; Found 686.1680.

#### *N*-(3-(1-(2-Cyanoethyl)-1*H*-tetrazol-5-yl)­phenyl)-*N*-(2-((4-cyclohexylphenyl)­amino)-2-oxoethyl)-2-(1,1-dioxidobenzo­[*b*]­thiophen-3-yl)­acetamide **2j**

The reaction was performed following the general procedure starting from 107 mg of **18** (with **3**, **15** and **48**), affording 201 mg of **2j** after chromatography with CH_2_Cl_2_/MeOH 98:2 as a pale yellow foam. Yield 66%. The isolated product was further purified by trituration with Et_2_O affording an ivory solid. *R*_f_ 0.28 (CH_2_Cl_2_/MeOH 97:3, A, B). Mp 144.5–145.3 °C. HPLC purity 98%, *R*_t_ 9.84 min (Gemini C18 150 mm × 3 mm, 5 μm column, A: H_2_O/MeCN 1:1 + 0.1% FA to B: MeCN + 0.1% FA in 20 min, flow 0.5 mL/min, temp. 24 °C). ^1^H NMR (300 MHz, DMSO-*d*_6_): δ 10.15 (s, 1H, NH), 7.96–7.85 (m, 2H, 2 × CH Ar), 7.84–7.64 (m, 6H, 6 × CH Ar), 7.50 (d, *J* = 8.2 Hz, 2H, 2 × CH para syst.), 7.15 (d, *J* = 8.3 Hz, 2H, 2 × CH para syst.), 6.75 (s, 1H, CH–SO_2_), 4.76 (t, *J* = 6.4 Hz, 2H, C*H_2_* CH_2_CN), 4.73 (d, *J* = 2.1 Hz, 2H, C*H_2_* C=CH), 4.60 (s, 2H, NCH_2_C=O), 3.12 (t, *J* = 6.4 Hz, 2H, CH_2_C*H*_*2*_CN), 2.47–2.35 (m, 1H, CH *c*-Hex), 1.85–1.62 (m, 5H, 5 × C*H*H *c*-Hex), 1.46–1.18 (m, 5H, 5 × CH*H* *c*-Hex). ^13^C NMR (75 MHz, DMSO-*d*_6_): δ 166.5 (C=O), 164.6 (C=O), 153.8 (C tetrazole), 143.1 (C quat. Ar), 142.8 (C quat. Ar), 139.6 (C quat. Ar), 137.7 (C quat. Ar), 136.6 (C quat. Ar), 134.3 (CH Ar), 134.2 (CH Ar), 132.7 (CH Ar), 130.6 (CH Ar + C quat. Ar), 128.3 (CH Ar), 128.0 (CH Ar), 126.9 (2 × CH para syst.), 124.5 (C quat. Ar), 123.4 (CH Ar), 121.4 (CH Ar), 119.3 (2 × CH para syst.), 117.9 (CN), 116.1 (CH–SO_2_), 54.7 (*C*H_2_C=CH), 53.0 (N*C*H_2_C=O), 43.6 (*C*H_2_CH_2_CN), 43.2 (CH *c*-Hex), 34.1 (2 × CH_2_ *c*-Hex), 26.4 (2 × CH_2_ *c*-Hex), 25.7 (CH_2_ *c*-Hex), 17.7 (CH_2_ *C*H_2_CN). IR (neat): *ν*_max_ (cm^–1^) 3318, 2924, 2850, 2256, 1653, 1608, 1531, 1516, 1478, 1467, 1449, 1414, 1386, 1300, 1252, 1202, 1149, 1122, 1066, 1029, 1014, 1001, 952, 919, 859, 829, 810, 783, 761, 724, 702, 668, 661, 613. HRMS (ESI^+^) *m*/*z*: [M + H]^+^ Calcd for C_34_H_34_N_7_O_4_S 636.2388; Found 636.2372.

#### 2-(1,1-Dioxidobenzo­[*b*]­thiophen-3-yl)-*N*-(2-(methylamino)-2-oxoethyl)-*N*-(3-nitrophenyl)­acetamide **2l**

The reaction was performed following the general procedure starting from 85 mg of **18** (with **4**, **12** and **48**). However, due to the volatility of MeNC (**4**), the reaction was performed in a closed vessel. 148 mg of **2l** were obtained after trituration with Et_2_O as a white solid. Yield 94%. *R*_f_ 0.18 (a highly elongated spot showing pronounced streaking on the TLC plate, probably due to the extremely low solubility of the sample, PE/CH_2_Cl_2_/EtOAc/AcOH 4:2:1:1, **A**, **B**). Mp 218.0–220.0 °C. HPLC purity 99%, *R*_t_ 9.40 min (Phenyl C6 150 mm × 3 mm, 3 μm column, A: H_2_O/MeCN 7:3 + 0.1% FA to B: MeCN + 0.1% FA in 20 min, flow 0.34 mL/min, temp. 26 °C). ^1^H NMR (400 MHz, DMSO-*d*_6_): δ 8.37 (s, 1H, CH Ar), 8.22 (d, *J* = 8.3 Hz, 1H, CH Ar), 8.12–8.00 (m, 1H, NH), 7.93–7.84 (m, 2H, 2 × CH Ar), 7.79 (bs, 1H, CH Ar), 7.75–7.64 (m, 3H, 3 × CH Ar), 6.67 (s, 1H, CH–SO_2_), 4.72 (s, 2H, C*H*_*2*_C=CH), 4.39 (s, 2H, NCH_2_C=O), 2.63 (d, *J* = 4.6 Hz, 3H, CH_3_). ^13^C NMR (101 MHz, DMSO-*d*_6_): δ 168.1 (C=O), 164.4 (C=O), 148.3 (C quat. Ar), 143.4 (C quat. Ar), 139.6 (C quat. Ar), 137.9 (C quat. Ar), 134.4 (C quat. Ar), 134.3 (CH Ar), 134.1 (CH Ar), 132.6 (CH Ar), 130.6 (CH Ar), 123.6 (CH Ar), 122.5 (CH Ar), 122.3 (CH Ar), 121.3 (CH Ar), 116.0 (CH–SO_2_), 54.7 (*C*H_2_C=CH), 52.2 (N*C*H_2_C=O), 25.6 (CH_3_). IR (neat): *ν*_max_ (cm^–1^) 3260, 3062, 1679, 1655, 1617, 1584, 1525, 1481, 1466, 1453, 1415, 1388, 1349, 1305, 1263, 1244, 1213, 1204, 1162, 1149, 1121, 1091, 1052, 1014, 926, 882, 868, 816, 805, 783, 767, 757, 743, 714, 704, 691, 649, 630. HRMS (ESI^+^) *m*/*z*: [M + H]^+^ Calcd for C_19_H_18_N_3_O_6_S 416.0911; Found 416.0922.

#### *N*-(3-Cyanophenyl)-2-(1,1-dioxidobenzo­[*b*]­thiophen-3-yl)-*N*-(2-(methylamino)-2-oxoethyl)­acetamide **2m**

The reaction was performed following the general procedure starting from 85 mg of **18** (with **4**, **13** and **48**). However, due to the volatility of MeNC (**4**), the reaction was performed in a closed vessel. 130 mg of **2m** were obtained after trituration with Et_2_O as a white solid. Yield 87%. *R*_f_ 0.13 (a highly elongated spot showing pronounced streaking on the TLC plate, probably due to the extremely low solubility of the sample, PE/CH_2_Cl_2_/EtOAc/AcOH 4:2:1:1, **A**, **B**). Mp 228.0–229.0 °C. HPLC purity 99%, *R*_t_ 8.51 min (Phenyl C6 150 mm × 3 mm, 3 μm column, A: H_2_O/MeCN 7:3 + 0.1% FA to B: MeCN + 0.1% FA in 20 min, flow 0.34 mL/min, temp. 26 °C). ^1^H NMR (300 MHz, DMSO-*d*_6_): δ 8.05–7.61 (m, 9H, 8 × CH Ar + NH), 6.63 (s, 1H, CH–SO_2_), 4.70 (d, *J* = 2.2 Hz, 2H, C*H*_*2*_C=CH), 4.36 (s, 2H, NCH_2_C=O), 2.62 (d, *J* = 4.5 Hz, 3H, CH_3_). ^13^C NMR (75 MHz, DMSO-*d*_6_): δ 168.1 (C=O), 164.5 (C=O), 143.2 (C quat. Ar), 139.6 (C quat. Ar), 137.7 (C quat. Ar), 134.3 (C quat. Ar), 134.2 (CH Ar), 134.7 (CH Ar), 132.6 (CH Ar), 131.4 (CH Ar), 130.9 (CH Ar), 130.7 (CH Ar), 123.5 (CH Ar), 121.4 (CH Ar), 118.3 (CN), 116.2 (CH–SO_2_), 112.3 (C quat. Ar), 54.7 (*C*H_2_C=CH), 52.2 (N*C*H_2_C=O), 25.7 (CH_3_). IR (neat): *ν*_max_ (cm^–1^) 3228, 3061, 2944, 2230, 1683, 1654, 1619, 1578, 1484, 1467, 1454, 1416, 1389, 1351, 1306, 1263, 1218, 1202, 1165, 1150, 1120, 1057, 1030, 1015, 924, 908, 876, 867, 819, 799, 782, 758, 707, 693, 665, 641, 610. HRMS (ESI^+^) *m*/*z*: [M + H]^+^ Calcd for C_20_H_18_N_3_O_4_S 396,1013; Found 396,1022.

#### *N*-(2-((2,6-Dimethylphenyl)­amino)-2-oxoethyl)-2-(*N*,4-dimethylphenylsulfonamido)-*N*-(3-nitrophenyl)­acetamide **2n**

The reaction was performed following the general procedure starting from 107 mg of **17** (with **5**, **12** and **48**), affording 134 mg of **2n** after trituration with Et_2_O as an off-white solid. Yield 71%. *R*_f_ 0.20 (PE/EtOAc 1:1, **A**, **B**). Mp 212.0–213.3 °C. HPLC purity 97%, *R*_t_ 7.79 min (Gemini C18 150 mm × 3 mm, 5 μm column, A: H_2_O/MeCN 1:1 + 0.1% FA to B: MeCN + 0.1% FA in 20 min, flow 0.5 mL/min, temp. 24 °C). ^1^H NMR (300 MHz, DMSO-*d*_6_, 90 °C): δ 9.24 (s, 1H, NH), 8.34 (t, *J* = 2.3 Hz, 1H, 2-CH nitrophenyl ring), 8.22 (ddd, *J* = 8.2, 2.3, 1.1 Hz, 1H, 4-CH nitrophenyl ring), 7.92 (d, *J* = 8.0 Hz, 1H, 6-CH nitrophenyl ring), 7.75 (t, *J* = 8.1 Hz, 1H, 5-CH nitrophenyl ring), 7.63–7.55 (m, 2H, 2 × CH para syst.), 7.38–7.30 (m, 2H, 2 × CH para syst.), 7.10–7.00 (m, 3H, 3 × CH dimethylphenyl ring), 4.56 (s, 2H, CH_2_), 3.93 (s, 2H, CH_2_), 2.74 (s, 3H, NCH_3_), 2.38 (s, 3H, CH_3_ of tosyl), 2.09 (s, 6H, 2 × CH_3_ dimethylphenyl ring). ^13^C NMR (75 MHz, DMSO-*d*_6_, 90 °C): δ 166.9 (C=O), 165.7 (C=O), 148.1 (C quat. Ar), 142.7 (C quat. Ar), 142.6 (C quat. Ar), 134.88 (C quat. Ar), 134.86 (C quat. Ar) 134.2 (C quat. Ar), 133.9 (6-CH nitrophenyl ring), 130.2 (5-CH nitrophenyl ring), 129.1 (2 × CH para syst.), 127.1 (CH dimethylphenyl ring), 126.6 (2 × CH dimethylphenyl ring), 126.1 (2 × CH para syst.), 122.3 (2-CH nitrophenyl ring), 122.0 (4-CH nitrophenyl ring), 52.0 (CH_2_), 50.9 (CH_2_), 34.9 (NCH_3_), 20.4 (CH_3_ of tosyl), 17.3 (2 × CH_3_ dimethylphenyl ring). IR (neat): *ν*_max_ (cm^–1^) 3237, 3058, 2979, 2919, 1671, 1599, 1531, 1475, 1445, 1405, 1348, 1335, 1306, 1284, 1271, 1250, 1216, 1162, 1122, 1088, 1040, 1019, 989, 966, 937, 917, 902, 873, 842, 808, 777, 766, 756, 743, 719, 702, 678, 655, 638, 608. HRMS (ESI^+^) *m*/*z*: [M + H]^+^ Calcd for C_26_H_29_N_4_O_6_S: 525.1802; Found: 525.1802.

#### *N*-(2-((2,6-Dimethylphenyl)­amino)-2-oxoethyl)-2-(1,1-dioxidobenzo­[*b*]­thiophen-3-yl)-*N*-(3-nitrophenyl)­acetamide **2o**

The reaction was performed following the general procedure starting from 81 mg of **18** (with **5**, **12** and **48**), affording 144 mg of **2o** after trituration with Et_2_O as an off-white solid with HPLC purity 97%. Yield 79%. After further trituration with Et_2_O/CH_2_Cl_2_/MeOH 7:3:0.5 an off-white solid was obtained, and the HPLC purity was improved up to 98%. *R*_f_ 0.19 (CH_2_Cl_2_ + 1% MeOH, **A**, **B**). Mp 275.5–276.1 °C. HPLC purity 98%, *R*_t_ 14.39 min (Phenyl C6 150 mm × 3 mm, 3 μm column, A: H_2_O/MeCN 7:3 + 0.1% FA to B: MeCN + 0.1% FA in 20 min, flow 0.34 mL/min, temp. 25 °C). ^1^H NMR (300 MHz, DMSO-*d*_6_): δ 9.52 (s, 1H, NH), 8.43 (s, 1H, CH Ar), 8.25 (d, *J* = 8.8 Hz, 1H, CH Ar), 7.99–7.60 (m, 6H, 6 × CH Ar), 7.07 (s, 3H, 3 × CH Ar), 6.73 (s, 1H, CH–SO_2_), 4.83–4.55 (m, 4H, C*H*_*2*_C=CH + NCH_2_C=O), 2.09 (s, 6H, 2 × CH_3_). ^13^C NMR (75 MHz, DMSO-*d*_6_): δ 166.4 (C=O), 164.6 (C=O), 148.3 (C quat. Ar), 143.3 (C quat. Ar), 139.6 (C quat. Ar), 137.9 (C quat. Ar), 135.3 (C quat. Ar), 134.6 (C quat. Ar), 134.3 (CH Ar), 134.2 (C quat. Ar), 134.1 (CH Ar), 132.6 (CH Ar), 130.7 (CH Ar), 127.7 (2 × CH Ar), 126.7 (CH Ar), 123.6 (CH Ar), 122.5 (CH Ar), 122.2 (CH Ar), 121.3 (CH Ar), 116.2 (CH–SO_2_), 54.7 (*C*H_2_C=CH), 52.2 (N*C*H_2_C=O), 18.0 (2 × CH_3_). IR (neat): *ν*_max_ (cm^–1^) 3190, 3066, 3023, 1656, 1616, 1524, 1466, 1419, 1386, 1346, 1307, 1263, 1241, 1213, 1146, 1120, 1074, 1048, 1014, 975, 930, 883, 864, 809, 773, 758, 744, 714, 705, 684, 619. HRMS (ESI^+^) *m*/*z*: [M + H]^+^ Calcd for C_26_H_24_N_3_O_6_S 506.1380; Found 506.1375.

#### *N*-(3-Cyanophenyl)-*N*-(2-((2,6-dimethylphenyl)­amino)-2-oxoethyl)-2-(1,1-dioxidobenzo­[*b*]­thiophen-3-yl)­acetamide **2p**

The reaction was performed following the general procedure starting from 78 mg of **18** (with **5**, **13** and **48**), affording 157 mg of **2p** after trituration with Et_2_O as an off-white solid. Yield 93%. *R*_f_ 0.31 (an elongated spot showing streaking on the TLC plate, probably due to the extremely low solubility of the sample, PE/CH_2_Cl_2_/EtOAc/AcOH 4:2:1:1, **A**, **B**). Mp 281.0–281.5 °C. HPLC purity 99%, *R*_t_ 5.36 min (Gemini C18 150 mm × 3 mm, 5 μm column, A: H_2_O/MeCN 1:1 + 0.1% FA to B: MeCN + 0.1% FA in 20 min, flow 0.5 mL/min, temp. 26 °C). ^1^H NMR (300 MHz, DMSO-*d*_6_): δ 9.47 (s, 1H, NH), 8.02 (s, 1H, CH Ar), 7.92–7.78 (m, 4H, 4 × CH Ar), 7.73–7.63 (m, 3H, 3 × CH Ar), 7.06 (s, 3H, 3 × CH dimethylphenyl ring), 6.69 (s, 1H, CH–SO_2_), 4.73 (s, 2H, C*H_2_* C=CH), 4.66 (s, 2H, NCH_2_C=O), 2.08 (s, 6H, 2 × CH_3_). ^13^C NMR (75 MHz, DMSO-*d*_6_): δ 166.4 (C=O), 164.7 (C=O), 143.0 (C quat. Ar), 139.6 (C quat. Ar), 137.7 (C quat. Ar), 135.3 (2 × C quat. Ar), 134.6 (C quat. Ar), 134.3 (C quat. Ar), 134.2 (CH Ar), 132.7 (CH Ar), 132.6 (CH Ar), 131.3 (CH Ar), 130.9 (CH Ar), 130.7 (CH Ar), 127.7 (2 × CH Ar), 126.7 (CH Ar), 123.5 (CH Ar), 121.4 (CH Ar), 118.3 (CN), 116.4 (CH–SO_2_), 112.3 (*C*CN Ar), 54.7 (*C*H_2_C=CH), 52.0 (N*C*H_2_C=O), 18.0. (2 × CH_3_). IR (neat): *ν*_max_ (cm^–1^) 3192, 3066, 2231, 1655, 1615, 1582, 1537, 1484, 1468, 1417, 1387, 1347, 1309, 1264, 1242, 1218, 1203, 1170, 1147, 1119, 1081, 1052, 1015, 968, 922, 869, 801, 774, 761, 708, 689, 633, 607. HRMS (ESI^+^) *m*/*z*: [M + H]^+^ Calcd for C_27_H_24_N_3_O_4_S 486.1482; Found 486.1477.

#### 3-(4-(2-(2-(1,1-Dioxidobenzo­[*b*]­thiophen-3-yl)-*N*-(3-nitrophenyl)­acetamido)­acetamido)­benzyl)-*N*-methylbenzamide **2q**

The reaction was performed following the general procedure starting from 62 mg of **18** (with **6**, **12** and **48**), affording 92 mg of **2q** after a trituration with Et_2_O and a chromatography with PE/CH_2_Cl_2_/EtOAc/MeOH 1:1:3:0.2 and then with CH_2_Cl_2_/MeOH 1:1 as an off-white solid. Yield 53%. *R*_f_ 0.31 (PE/CH_2_Cl_2_/EtOAc/MeOH 1:1:3:0.2, **A**, **B**). Mp 227.2–229.9 °C. HPLC purity 97%, *R*_t_ 7.33 min (ACE Excel 3 C18-AR 150 mm × 3 mm, 3 μm column, A: H_2_O/MeCN 1:1 + 0.1% FA to B: MeCN + 0.1% FA in 20 min, flow 0.38 mL/min, temp. 30 °C). ^1^H NMR (400 MHz, DMSO-*d*_6_): δ 10.21 (s, 1H; Ar-NH), 8.42–8.33 (m, 2H, N*H*-CH_3_ + CH Ar), 8.29–8.18 (m, 1H, CH Ar), 7.93–7.85 (m, 2H, 2 × CH Ar), 7.85–7.78 (m, 1H, CH Ar), 7.73 (t, *J* = 8.1 Hz, 1H, CH Ar), 7.71–7.66 (m, 3H, 3 × CH Ar), 7.65–7.61 (m, 1H, CH Ar), 7.52 (d, *J* = 8.2 Hz, 2H, 2 × CH para syst.), 7.38–7.32 (m, 2H, 2 × CH Ar), 7.18 (d, *J* = 8.4 Hz, 2H, 2 × CH para syst.), 6.71 (s, 1H, CH–SO_2_), 4.72 (d, *J* = 2.1 Hz, 2H, C*H_2_* C=CH), 4.60 (s, 2H, NCH_2_C=O), 3.94 (s, 2H, Ar–CH_2_–Ar), 2.76 (d, *J* = 4.6 Hz, 3H, CH_3_). ^13^C NMR (101 MHz, DMSO-*d*_6_): δ 166.64 (C=O), 166.56 (C=O), 164.6 (C=O), 148.3 (C quat. Ar), 143.4 (C quat. Ar), 141.6 (C quat. Ar), 139.6 (C quat. Ar), 138.2 (C quat. Ar), 136.9 (C quat. Ar), 136.0 (C quat. Ar), 134.7 (CH Ar), 134.6 (C quat. Ar), 134.3 (C quat. Ar), 134.1 (CH Ar), 132.6 (CH Ar), 131.3 (CH Ar), 130.6 (CH Ar), 129.1 (2 × CH para syst.), 128.4 (CH Ar), 127.4 (CH Ar), 124.6 (CH Ar), 123.6 (CH Ar), 122.5 (CH Ar), 122.3 (CH Ar), 121.3 (CH Ar), 119.4 (2 × CH para syst.), 115.8 (CH–SO_2_), 54.7 (*C*H_2_C=CH), 52.9 (N*C*H_2_C=O), 40.4 (Ar–CH_2_–Ar), 26.2 (CH_3_). IR (neat): *ν*_max_ (cm^–1^) 3263, 3068, 2925, 1657, 1604, 1584, 1530, 1512, 1481, 1467, 1415, 1388, 1348, 1301, 1262, 1221, 1205, 1149, 1121, 1068, 1048, 1015, 970, 934, 883, 863, 836, 797, 755, 743, 713, 689, 650, 632, 608. HRMS (ESI^+^) *m*/*z*: [M + H]^+^ Calcd for C_33_H_29_N_4_O_7_S 625.1751; Found 625,1739.

#### *N*-Cyclopropyl-3-(4-(2-(2-(1,1-dioxidobenzo­[*b*]­thiophen-3-yl)-*N*-(3-nitrophenyl)­acetamido)­acetamido)­benzyl)­benzamide **2r**

The reaction was performed following the general procedure starting from 60 mg of **18** (with **7**, **12** and **48**), affording 73 mg of **2r** after a trituration with Et_2_O and a double chromatography, with PE/CH_2_Cl_2_/EtOAc/MeOH 1:0.7:2.5:0.1 as an off-white solid. Yield 42%. *R*_f_ 0.24 (PE/CH_2_Cl_2_/EtOAc/MeOH 1:0.7:2.5:0.1, **A**, **B**). Mp 220 °C (dec.). HPLC purity 96%, *R*_t_ 6.44 min (Gemini C18 150 mm × 3 mm, 5 μm column, A: H_2_O/MeCN 1:1 + 0.1% FA to B: MeCN + 0.1% FA in 20 min, flow 0.5 mL/min, temp. 24 °C). ^1^H NMR (400 MHz, DMSO-*d*_6_): δ 10.21 (s, 1H, Ar-NH), 8.38 (d, *J* = 4.1 Hz, 2H, NH-*c*Pr + CH Ar), 8.24 (d, *J* = 8.3 Hz, 1H, CH Ar), 7.93–7.85 (m, 2H, 2 × CH Ar), 7.86–7.77 (m, 1H, CH Ar), 7.73 (t, *J* = 8.1 Hz, 1H, CH Ar), 7.70–7.65 (m, 3H, 3 × CH Ar), 7.64–7.59 (m, 1H, CH Ar), 7.52 (d, *J* = 8.2 Hz, 2H, 2 × CH para syst.), 7.39–7.31 (m, 2H, 2 × CH Ar), 7.17 (d, *J* = 8.3 Hz, 2H, 2 × CH para syst.), 6.71 (s, 1H, CH–SO_2_), 4.72 (s, 2H, C*H_2_* C=CH), 4.60 (s, 2H, NCH_2_C=O), 3.93 (s, 2H, Ar–CH_2_–Ar), 2.81 (tq, *J* = 7.8, 4.0 Hz, 1H, CH of *c*Pr), 0.67 (td, *J* = 7.1, 4.6 Hz, 2H, C*H*H of *c*Pr), 0.59–0.51 (m, 2H, CH*H* of *c*Pr). ^13^C NMR (101 MHz, DMSO-*d*_6_): δ 167.5 (C=O), 166.6 (C=O), 164.6 (C=O), 147.6 (C quat. Ar), 143.5 (C quat. Ar), 141.5 (C quat. Ar), 139.6 (C quat. Ar), 138.7 (C quat. Ar), 137.5 (C quat. Ar), 136.4 (C quat. Ar), 134.6 (CH Ar), 134.5 (C quat. Ar), 132.6 (C quat. Ar), 131.4 (CH Ar), 130.6 (CH Ar), 129.1 (2 × CH para syst.), 128.3 (CH Ar), 127.5 (CH Ar), 124.8 (CH Ar), 123.6 (CH Ar), 122.5 (CH Ar), 122.2 (CH Ar), 121.3 (CH Ar), 119.3 (2 × CH para syst.), 115.8 (CH–SO_2_), 54.7 (*C*H_2_C=CH), 52.5 (N*C*H_2_C=O), 40.3 (Ar–CH_2_–Ar), 23.0 (CH of *c*Pr), 5.7 (CH_2_ of *c*Pr). IR (neat): *ν*_max_ (cm^–1^) 3258, 3039, 2933, 2231, 1653, 1629, 1584, 1525, 1481, 1469, 1442, 1417, 1388, 1349, 1301, 1263, 1208, 1149, 1123, 1077, 1049, 1017, 977, 961, 932, 900, 885, 856, 835, 798, 781, 762, 746, 715, 693, 651, 631. HRMS (ESI^+^) *m*/*z*: [M + H]^+^ Calcd for C_13_H_17_NO_3_S 651.1908; Found 651.1915.

#### Benzyl 4-(2-(2-(1,1-Dioxidobenzo­[*b*]­thiophen-3-yl)-*N*-(3-nitrophenyl)­acetamido)­acetamido)­benzoate **2s**

The reaction was performed following the general procedure starting from 325 mg of **18** (with **8**, **12** and **48**), affording 408 mg of **2s** after a trituration with Et_2_O and a chromatography with PE/CH_2_Cl_2_/EtOAc/MeOH 3.5:1:1:0.3 as a white solid. Yield 46%. *R*_f_ 0.36 (PE/CH_2_Cl_2_/EtOAc/MeOH 3.5:1:1:0.3, **A**, **B**). Mp 238.3–239.9 °C (dec.). HPLC purity 99%, *R*_t_ 9.76 min (Gemini C18 150 mm × 3 mm, 5 μm column, A: H_2_O/MeCN 1:1 + 0.1% FA to B: MeCN + 0.1% FA in 20 min, flow 0.5 mL/min, temp. 26 °C). ^1^H NMR (300 MHz, DMSO-*d*_6_): δ 10.65 (s, 1H, NH), 8.38 (s, 1H, CH Ar), 8.25 (d, *J* = 8.0 Hz, 1H, CH Ar), 8.05–7.64 (m, 10H, 10 × CH Ar), 7.53–7.28 (m, 5H, 5 × CH Ar), 6.72 (s, 1H, CH–SO_2_), 5.32 (s, 2H, OCH_2_), 4.73 (d, *J* = 1.8 Hz, 2H, C*H*_*2*_C=CH), 4.66 (s, 2H, NCH_2_C=O). ^13^C NMR (75 MHz, DMSO-*d*_6_): δ 167.5 (C=O), 165.2 (C=O), 164.6 (C=O), 148.4 (C quat. Ar), 143.4 (C quat. Ar), 143.3 (C quat. Ar), 139.6 (C quat. Ar), 138.4 (C quat. Ar), 136.3 (C quat. Ar), 134.6 (CH Ar), 134.3 (C quat. Ar), 134.1 (CH Ar), 132.7 (CH Ar), 130.7 (CH Ar), 130.6 (CH Ar), 128.6 (CH Ar), 128.2 (CH Ar), 128.1 (CH Ar), 124.1 (C quat. Ar), 123.7 (CH Ar), 122.6 (CH Ar), 122.4 (CH Ar), 121.4 (CH Ar), 118.7 (CH Ar), 115.7 (CH–SO_2_), 66.0, (OCH_2_) 54.7 (*C*H_2_C=CH), 53.2 (N*C*H_2_C=O). IR (neat): *ν*_max_ (cm^–1^) 1719, 1658, 1603, 1528, 1466, 1411, 1389, 1348, 1307, 1270, 1205, 1172, 1149, 1122, 1098, 1017, 862, 758, 714, 696, 611. HRMS (ESI^+^) *m*/*z*: [M + H]^+^ Calcd for C_32_H_26_N_3_O_8_S 612.1435; Found 612.1465.

#### 2-(1,1-Dioxidobenzo­[*b*]­thiophen-3-yl)-*N*-(3-(*N*-(methylsulfonyl)­methylsulfonamido)­phenyl)-*N*-(2-oxo-2-((3-(trifluoromethoxy)­phenyl)­amino)­ethyl)­acetamide **2t**

The reaction was performed following the general procedure starting from 110 mg of **18** (with **9**, **14** and **48**), affording 135 mg of **2t** after a chromatography with PE/EtOAc 1:1 to PE/EtOAc 1:1 + 2% MeOH and a trituration with Et_2_O and as a white solid with HPLC purity 94%. Yield 40%. *R*_f_ 0.36 (PE/EtOAc 1:1+ 1% MeOH, A, B). Mp 186.5–188.2 °C. After further purification by chromatography (PE/EtOAc 1:1+ 1% MeOH) a white solid was obtained and the HPLC purity was improved up to 96%, *R*_t_ 9.69 min (Phenyl C6 150 mm × 3 mm, 3 μm column, A: H_2_O/MeCN 1:1 + 0.1% FA to B: MeCN + 0.1% FA in 20 min, flow 0.34 mL/min, temp. 25 °C). ^1^H NMR (400 MHz, DMSO-*d*_6_): δ 10.55 (s, 1H, NH), 7.93–7.86 (m, 1H, CH Ar), 7.84–7.77 (m, 1H, CH Ar), 7.74–7.57 (m, 7H, 7 × CH Ar), 7.53–7.43 (m, 2H, 2 × CH Ar), 7.11–7.01 (m, 1H, CH Ar), 6.62 (s, 1H, CH–SO_2_), 4.73 (d, *J* = 2.2 Hz, 2H, C*H*_*2*_C=CH), 4.61 (s, 2H, NCH_2_C=O), 3.50 (s, 6H, 2 × CH_3_). ^13^C NMR (101 MHz, DMSO-*d*_6_): [triple decoupled {^1^H} and {^19^F}] δ 167.3 (C=O), 164.3 (C=O), 148.5 (C quat. Ar), 143.2 (C quat. Ar), 140.4 (C quat. Ar), 139.6 (C quat. Ar), 138.1 (C quat. Ar), 134.3 (C quat. Ar), 134.1 (C quat. Ar), 134.0 (CH Ar), 132.7 (CH Ar), 130.7 (CH Ar), 130.6 (CH Ar), 130.4 (CH Ar), 130.3 (CH Ar), 128.7 (CH Ar), 123.0 (CH Ar), 121.4 (CH Ar), 120.1 (CF_3_), 117.7 (CH Ar), 115.5 (CH Ar), 115.0 (CH Ar), 111.2 (CH Ar), 54.6 (*C*H_2_C=CH), 53.2 (N*C*H_2_C=O), 43.0 (2 × CH_3_). IR (neat): *ν*_max_ (cm^–1^) 3276, 3213, 3145, 3096, 3053, 2941, 1678, 1652, 1611, 1558, 1486, 1467, 1445, 1418, 1388, 1372, 1350, 1306, 1263, 1230, 1209, 1186, 1150, 1125, 1088, 1051, 1003, 974, 963, 934, 914, 870, 822, 803, 759, 703, 657, 645, 633, 602. HRMS (ESI^+^) *m*/*z*: [M + H]^+^ Calcd for C_27_H_25_F_3_N_3_O_9_S_3_ 688.0700; Found 688.0709.

#### *N*-(2-((3-((*tert*-butyldimethylsilyl)­oxy)­phenyl)­amino)-2-oxoethyl)-2-(1,1-dioxidobenzo­[*b*]­thiophen-3-yl)-*N*-(3-(*N*-(methylsulfonyl)­methylsulfonamido)­phenyl)­acetamide 2v-P

The reaction was performed following the general procedure starting from 85 mg of **18** (with **10**, **14** and **48**), affording 184 mg of **2v-P** after a trituration with Et_2_O as an ochre solid. Yield 66%. *R*_f_ 0.20 (PE/EtOAc 1:1, A, B). Mp 223 °C (dec.). ^1^H NMR (300 MHz, CDCl_3_ + small amount of MeOH-*d*_4_) [2 conformers in a ratio of ∼8:2. Only the peaks of the major conformer are reported, when two distinct signals are present]: δ 7.78 (d, *J* = 6.7 Hz, 1H, CH Ar), 7.67–7.56 (m, 6H, 6 × CH Ar), 7.50–7.41 (m, 1H, CH Ar), 7.24–7.07 (m, 3H, 3 × CH Ar), 6.67 (s, 1H, CH Ar), 6.59 (d, *J* = 7.8 Hz, 1H, CH Ar), 4.76 (d, *J* = 2.2 Hz, 2H, C*H*_*2*_C=CH), 4.55 (s, 2H, NCH_2_C=O), 3.44 (s, 6H, 2 × CH_3_ of sulfonimide), 0.98 (s, 9H, 3 × CH_3_ of *t*-Bu), 0.20 (s, 6H, 2 × CH_3_ of TBS). ^13^C NMR (75 MHz, CDCl_3_ + small amount of MeOH-*d*_4_): δ 166.2 (C=O), 165.2 (C=O), 156.0 (C–O), 143.3 (C quat. Ar), 139.8 (C quat. Ar), 139.0 (C quat. Ar), 134.4 (C quat. Ar), 134.3 (C quat. Ar), 133.8 (C quat. Ar), 132.5 (CH Ar), 131.1 (CH Ar), 130.4 (CH Ar), 129.4 (CH Ar), 129.3 (CH Ar), 123.3 (CH Ar), 122.5 (CH Ar), 121.4 (CH Ar), 121.3 (CH Ar), 115.9 (CH Ar), 114.2 (CH Ar), 112.7 (CH Ar), 111.7 (CH Ar), 55.1 (*C*H_2_C=CH), 53.3 (N*C*H_2_C=O), 42.7 (2 × CH_3_ of sulfonimide), 25.5 (3 × CH_3_ of *t*-Bu), 18.0 (C quat. of *t*-Bu), −4.7 (2 × CH_3_ of TBS). IR (neat): *ν*_max_ (cm^–1^) 1594, 1367, 1351, 1306, 1255, 1233, 1209, 1150, 1125, 970, 932, 860, 842, 777, 758, 702, 690, 603.

### Post-Ugi Transformations (Scheme 9)

#### General Procedure for Catalytic Hydrogenation

To a solution of the substrate (0.144 mmol) in dry MeOH/THF 1:1 (1.4 mL) Pd/C 10% (25% w/w) was added. The reaction mixture was placed under a hydrogen atmosphere and stirred at rt overnight. The reaction was filtered over a Celite pad, which was washed with MeOH. When a different solvent was used (see specific procedures), the same solvent was employed to wash the Celite. The solution was concentrated to dryness, and the residue was purified as described for each compound.

#### 4-(*N*-(2-((4-Cyclohexylphenyl)­amino)-2-oxoethyl)-2-(*N*,4-dimethylphenylsulfonamido)­acetamido)-2-hydroxybenzoic Acid **2a**

The reaction was performed following the general procedure starting from 112 mg of **2a-P**, affording 68 mg of **2a** after trituration with pentane/Et_2_O 3:1 as a lilac solid. Yield 79%. *R*_f_ 0.45 (PE/CH_2_Cl_2_/EtOAc/AcOH 4:2:1:1, **A**, **B**). Mp 144.8–146.2 °C. HPLC purity 99%, *R*_t_ 13.63 min (Gemini C18 150 mm × 3 mm, 5 μm column, A: H_2_O/MeCN 1:1 + 0.1% FA to B: MeCN + 0.1% FA in 20 min, flow 0.5 mL/min, temp. 26 °C). ^1^H NMR (300 MHz, DMSO-*d*_6_, OH peaks not visible due to exchange with water): δ 10.00 (s, 1H, NH), 7.83 (d, *J* = 8.4 Hz, 1H, 6-CH salicylic ring), 7.56 (d, *J* = 8.1 Hz, 2H, 2 × CH para syst.), 7.48 (d, *J* = 8.5 Hz, 2H, 2 × CH para syst.), 7.30 (d, *J* = 8.1 Hz, 2H, 2 × CH para syst.), 7.16 (d, *J* = 8.5 Hz, 2H, 2 × CH para syst.), 7.05 (s, 1H, 3-CH salicylic ring), 6.99 (d, *J* = 7.6 Hz, 1H, 5-CH salicylic ring), 4.38 (s, 2H, CH_2_), 3.92 (s, 2H, CH_2_), 2.72 (s, 3H, NCH_3_), 2.48–2.36 (m, 1H, CH *c*-Hex), 2.31 (s, 3H, CH_3_ tosyl ring), 1.85–1.58 (m, 5H, 5 × C*H*H *c*-Hex), 1.48–1.22 (m, 5H, 5 × CH*H* *c*-Hex). ^13^C NMR (75 MHz, DMSO-*d*_6_): δ 171.2 (C=O), 166.8 (C=O), 166.2 (C=O), 161.8 (C–OH), 147.7 (C–N salicylic ring), 143.0 (C quat. Ar), 142.7 (C quat. Ar), 136.5 (C quat. Ar), 135.0 (C quat. Ar), 131.3 (6-CH salicylic ring), 129.5 (2 × CH para syst.), 127.1 (2 × CH para syst.), 126.8 (2 × CH para syst.), 119.2 (2 × CH para syst.), 118.1 (5-CH salicylic ring), 115.7 (3-CH salicylic ring), 113.0 (*C*–CO_2_H), 52.7 (CH_2_), 50.9 (CH_2_), 43.2 (CH *c*-Hex), 35.4 (NCH_3_), 34.0 (2 × CH_2_ *c*-Hex), 26.3 (2 × CH_2_ *c*-Hex), 25.6 (CH_2_ *c*-Hex), 20.9 (CH_3_ tosyl ring). IR (neat): *ν*_max_ (cm^–1^) 3320, 2924, 2851, 1664, 1607, 1535, 1515, 1447, 1414, 1307, 1288, 1240, 1213, 1157, 1089, 1035, 1019, 1000, 935, 881, 815, 776, 708, 676, 651. HRMS (ESI^+^) *m*/*z*: [M + H]^+^ Calcd for C_31_H_36_N_3_O_7_S 594.2268; Found 594.2275.

#### 4-(*N*-(2-((4-Cyclohexylphenyl)­amino)-2-oxoethyl)-2-(1,1-dioxido-2,3-dihydrobenzo­[*b*]­thiophen-3-yl)­acetamido)-2-hydroxybenzoic Acid **2b**

The reaction was performed following the general procedure starting from 62 mg of **2b-P**, affording 40 mg of **2b** after trituration with pentane/Et_2_O 3:1 as a lilac solid. Yield 85%. It is worth noting that, under these conditions, the double bond of the thiophene ring was also hydrogenated. *R*_f_ 0.31 (PE/CH_2_Cl_2_/EtOAc/AcOH 4:2:1:1, A, B). Mp 130.1–132.0 °C. HPLC purity 99%, *R*_t_ 10.89 min (Gemini C18 150 mm × 3 mm, 5 μm column, A: H_2_O/MeCN 1:1 + 0.1% FA to B: MeCN + 0.1% FA in 20 min, flow 0.5 mL/min, temp. 26 °C). ^1^H NMR (300 MHz, DMSO-*d*_6_, OH peaks not visible due to exchange with water): δ 10.03 (s, 1H, NH), 7.77 (d, *J* = 8.2 Hz, 1H, 6-CH salicylic ring), 7.72–7.51 (m, 4H, 4 × CH Ar), 7.49 (d, *J* = 8.0 Hz, 2H, 2 × CH para syst.), 7.16 (d, *J* = 8.2 Hz, 2H, 2 × CH para syst.), 6.99 (s, 1H, 3-CH salicylic ring), 6.92 (d, *J* = 8.1 Hz, 1H, 6-CH salicylic ring), 4.44 (s, 2H, NCH_2_C=O), 4.06–3.91 (m, 1H, C*H*-CH_2_–C=O), 3.80–3.65 (m, 1H, CH–C*H*H–C=O), ca. 3.42 (CH–CH*H*–C=O buried by water), ca. 2.40 (CH_2_–SO_2_ + CH *c*-Hex, buried by solvent) 1.88–1.61 (m, 5H, 5 × C*H*H *c*-Hex), 1.44–1.17 (m, 5H, 5 × CH*H* *c*-Hex). ^13^C NMR (75 MHz, DMSO-*d*_6_): δ 171.1 (C=O), 169.0 (C=O), 166.4 (C=O), 161.8 (C–OH), 148.2 (C–N salicylic ring), 142.7 (C quat. Ar), 140.8 (C quat. Ar), 138.8 (C quat. Ar), 136.5 (C quat. Ar), 133.6 (CH Ar), 131.2 (6-CH salicylic ring), 129.0 (CH Ar), 126.8 (2 × CH para syst.), 126.6 (CH Ar), 120.7 (CH Ar), 119.2 (2 × CH para syst.), 118.1 (5-CH salicylic ring), 115.9 (3-CH salicylic ring), 112.9 (*C*–CO_2_H), 55.3 (CH–*C*H_2_–C=O), 52.7 (N*C*H_2_C=O), 43.2 (CH *c*-Hex), 38.1 (CH_2_–SO_2_), 34.5 (*C*H–CH_2_–C=O), 34.0 (2 × CH_2_ *c*-Hex), 26.3 (2 × CH_2_ *c*-Hex), 25.6 (CH_2_ *c*-Hex). IR (neat): *ν*_max_ (cm^–1^) 3323, 2924, 2851, 1655, 1606, 1537, 1515, 1447, 1414, 1288, 1241, 1187, 1151, 1114, 1060, 1033, 1000, 949, 871, 830, 758, 708, 672. HRMS (ESI^+^) *m*/*z*: [M + H]^+^ Calcd for C_31_H_31_N_2_O_7_S 575.1846; Found 575.1835.

#### General Procedure for Ester Hydrolysis and Monodesulfonylation

The substrate (**2e**, **2h**, **2t** or **2v-P**) was dissolved (completely or partially) in the appropriate solvent mixture, selected for each compound due to their generally poor solubility. Then aqueous 2.5 M NaOH (5.0 equiv) was added, and the reaction was stirred until complete at rt. The solution was partitioned between CH_2_Cl_2_/H_2_O, the pH was adjusted to 3 by addition of 2 M HCl and the extraction was performed with CH_2_Cl_2_. The combined organic layers were washed with brine, dried over Na_2_SO_4_, and concentrated. The crude product was dried under high vacuum for 1 h and then triturated with Et_2_O. Specific information are given for each product.

#### 3-(*N*-(2-((4-Cyclohexylphenyl)­amino)-2-oxoethyl)-2-(1,1-dioxidobenzo­[*b*]­thiophen-3-yl)­acetamido)­benzoic Acid **2f**

The reaction was performed following the general procedure starting from 100 mg of **2e**, partially dissolved in MeOH/CH_2_Cl_2_/DMSO 1:1:0.013 (3 + 3 mL + 40 μL), and the reaction lasted 3 h. An ivory solid (**2f**, 71 mg) was obtained. Yield 73%. *R*_f_ 0.11 (PE/CH_2_Cl_2_/EtOAc/MeOH 4:1:1:0.5, **A**). Mp 210 °C (dec). HPLC purity 97%, *R*_t_ 9.17 min (Gemini C18 150 mm × 3 mm, 5 μm column, A: H_2_O/MeCN 1:1 + 0.1% FA to B: MeCN + 0.1% FA in 20 min, flow 0.5 mL/min, temp. 24 °C). ^1^H NMR (300 MHz, DMSO-*d*_6_) [CO_2_H exchanges with water]: δ 10.10 (s, 1H, NH), 8.04 (s, 1H, CH Ar), 7.95 (d, *J* = 7.2 Hz, 1H, CH Ar), 7.92–7.84 (m, 1H, CH Ar), 7.77–7.65 (m, 4H, 4 × CH Ar), 7.58 (t, *J* = 7.8 Hz, 1H, CH Ar), 7.49 (d, *J* = 8.4 Hz, 2H, 2 × CH para syst.), 7.15 (d, *J* = 8.5 Hz, 2H, 2 × CH para syst.), 6.64 (s, 1H, CH–SO_2_), 4.72 (s, 2H, C*H_2_* C=CH), 4.56 (s, 2H, NCH_2_C=O), 2.47–2.36 (m, 1H, CH *c*-Hex), 1.85–1.64 (m, 5H, 5 × C*H*H *c*-Hex), 1.35 (t, *J* = 10.2 Hz, 5H, 5 × CH*H* *c*-Hex). ^13^C NMR (75 MHz, DMSO-*d*_6_): δ 166.7 (C=O), 166.4 (C=O), 164.4 (C=O), 142.7 (C quat. Ar), 142.6 (C quat. Ar), 139.6 (C quat. Ar), 137.5 (C quat. Ar), 136.6 (C quat. Ar), 134.3 (C quat. Ar), 134.2 (C quat. Ar), 132.5 (CH Ar), 132.3 (CH Ar), 132.1 (CH Ar), 129.7 (CH Ar), 128.6 (CH Ar), 128.1 (CH Ar), 126.8 (2 × CH para syst.), 123.2 (CH Ar), 121.3 (CH Ar), 119.2 (2 × CH para syst.), 115.9 (CH–SO_2_), 54.7 (*C*H_2_C=CH), 52.9 (N*C*H_2_C=O), 43.2 (CH *c*-Hex), 34.0 (2 × CH_2_ *c*-Hex), 26.4 (2 × CH_2_ *c*-Hex), 25.6 (CH_2_ *c*-Hex). IR (neat): *ν*_max_ (cm^–1^) 3284, 2997, 2921, 2851, 1682, 1655, 1603, 1583, 1533, 1488, 1453, 1416, 1383, 1299, 1259, 1210, 1149, 1120, 1081, 1049, 1015, 1004, 962, 932, 880, 854, 820, 783, 761, 706, 691, 644. HRMS (ESI^+^) *m*/*z*: [M + H]^+^ Calcd for C_31_H_31_N_2_O_6_S 559.1897; Found 559.1878.

#### *N*-(2-((4-Cyclohexylphenyl)­amino)-2-oxoethyl)-2-(1,1-dioxidobenzo­[*b*]­thiophen-3-yl)-*N*-(3-(methylsulfonamido)­phenyl)­acetamide **2i**

The reaction was performed following the general procedure starting from 40 mg of **2h**, dissolved at 50 °C in MeOH/CH_2_Cl_2_/DMSO 1:1:0.14 (1.5 mL), and the reaction lasted 1 h at rt. A pale yellow solid (**2i**, 25 mg) was obtained. Yield 69%. *R*_f_ 0.17 (PE/CH_2_Cl_2_/EtOAc/MeOH 4:1:1:0.5, **A**). Mp 180.5–181.9 °C. HPLC purity 98%, *R*_t_ 9.57 min (Gemini C18 150 mm × 3 mm, 5 μm column, A: H_2_O/MeCN 1:1 + 0.1% FA to B: MeCN + 0.1% FA in 20 min, flow 0.5 mL/min, temp. 24 °C). ^1^H NMR (400 MHz, DMSO-*d*_6_): δ 10.07 (s, 1H, NH amide), 9.90 (s, 1H, NH sulfonamide), 7.93–7.86 (m, 1H, CH Ar), 7.77–7.65 (m, 3H, 3 × CH Ar), 7.49 (d, *J* = 8.3 Hz, 2H, 2 × CH para syst.), 7.42 (t, *J* = 8.0 Hz, 1H, CH Ar), 7.29 (s, 1H, CH Ar), 7.24–7.17 (m, 2H, 2 × CH Ar), 7.15 (d, *J* = 8.6 Hz, 2H, 2 × CH para syst.), 6.73 (s, 1H, CH–SO_2_), 4.70 (d, *J* = 2.2 Hz, 2H, C*H*_*2*_C=CH), 4.52 (s, 2H, NCH_2_C=O), 2.97 (s, 3H, CH_3_), 2.48–2.38 (m, 1H, CH *c*-Hex), 1.85–1.66 (m, 5H, 5 × C*H*H *c*-Hex), 1.47–1.14 (m, 5H, 5 × CH*H* *c*-Hex). ^13^C NMR (101 MHz, DMSO-*d*_6_): δ 166.1 (C=O), 164.2 (C=O), 143.1 (C quat. Ar), 142.6 (C quat. Ar), 139.4 (C quat. Ar), 139.3 (C quat. Ar), 137.1 (C quat. Ar), 136.5 (C quat. Ar), 134.2 (CH Ar), 134.0 (C quat. Ar), 132.4 (CH Ar), 130.2 (CH Ar), 126.7 (2 × CH para syst.), 123.1 (CH Ar), 122.3 (CH Ar), 121.2 (CH Ar), 119.1 (2 × CH para syst.), 118.7 (CH Ar), 118.5 (CH Ar), 116.0 (CH–SO_2_), 54.6 (*C*H_2_C=CH), 52.8 (N*C*H_2_C=O), 43.1 (CH *c*-Hex), 39.9 (CH_3_, buried by DMSO), 33.9 (2 × CH_2_ *c*-Hex), 26.3 (2 × CH_2_ *c*-Hex), 25.5 (CH_2_ *c*-Hex). IR (neat): *ν*_max_ (cm^–1^) 3262, 2923, 2850, 1673, 1648, 1599, 1529, 1468, 1450, 1416, 1386, 1337, 1301, 1256, 1230, 1205, 1147, 1122, 1056, 1016, 1002, 971, 915, 869, 829, 766, 708, 650. HRMS (ESI^+^) *m*/*z*: [M + H]^+^ Calcd for C_31_H_34_N_3_O_6_S_2_ 608.1884; Found 608.1892.

#### 2-(1,1-Dioxidobenzo­[*b*]­thiophen-3-yl)-*N*-(3-(methylsulfonamido)­phenyl)-*N*-(2-oxo-2-((3-(trifluoromethoxy)­phenyl)­amino)­ethyl)­acetamide **2u**

The reaction was performed following the general procedure starting from 45 mg of **2t**, dissolved in MeOH/MeCN 1:1 (1.4 mL), and the reaction lasted 1 h. A white solid (**2u**, 28 mg) was obtained. Yield 70%. *R*_f_ 0.32 (PE/EtOAc 1:1 + 2% MeOH, A, B). Mp 205.0–206.0 °C (dec.). HPLC purity 96%, *R*_t_ 8.18 min (Phenyl C6 150 mm × 3 mm, 3 μm column, A: H_2_O/MeCN 1:1 + 0.1% FA to B: MeCN + 0.1% FA in 20 min, flow 0.34 mL/min, temp. 25 °C). ^1^H NMR (300 MHz, MeCN-*d*_3_): δ 8.75 (s, 1H, NH), 7.86–7.55 (m, 6H, 6 × CH Ar), 7.50–7.32 (m, 4H, 4 × CH Ar), 7.31–7.20 (m, 2H, CH Ar + NH), 7.02 (d, *J* = 7.6 Hz, 1H, CH Ar), 6.70 (s, 1H, CH–SO_2_), 4.67 (d, *J* = 2.1 Hz, 2H, C*H*_*2*_C=CH), 4.52 (s, 2H, NCH_2_C=O), 2.91 (s, 3H, CH_3_). ^13^C NMR (75 MHz, MeCN-*d*_3_): δ 168.2 (C=O), 166.0 (C=O), 150.2 (C quat. Ar), 144.4 (C quat. Ar), 141.1 (C quat. Ar), 141.0 (C quat. Ar), 140.0 (C quat. Ar), 139.3 (C quat. Ar), 135.6 (C quat. Ar), 134.8 (CH Ar), 133.5 (CH Ar), 131.6 (CH Ar), 131.4 (CH Ar), 124.5 (CH Ar), 121.50 (q, *J* = 255.5 Hz, CF_3_), 122.1 (CH Ar), 120.8 (CH Ar), 120.6 (CH Ar), 118.9 (CH Ar), 117.0 (CH Ar), 116.7 (CH Ar), 112.9 (CH Ar), 56.0 (*C*H_2_C=CH), 54.4 (N*C*H_2_C=O), 39.7 (CH_3_). IR (neat): *ν*_max_ (cm^–1^) 3272, 3083, 2930, 1675, 1650, 1603, 1542, 1482, 1422, 1403, 1389, 1336, 1306, 1258, 1237, 1208, 1143, 1126, 1058, 1016, 1003, 976, 918, 885, 868, 858, 777, 755, 703, 678, 651, 628. HRMS (ESI^+^) *m*/*z*: [M + H]^+^ Calcd for C_26_H_23_F_3_N_3_O_7_S_2_ 610.0924; Found 610.0932.

#### 2-(1,1-Dioxidobenzo­[*b*]­thiophen-3-yl)-*N*-(2-((3-hydroxyphenyl)­amino)-2-oxoethyl)-*N*-(3-(*N*-(methylsulfonyl)­methylsulfonamido)­phenyl)­acetamide **2v**

97 mg (0.132 mmol) of **2v-P**, not completely dissolved in MeOH/MeCN 1:1 (4 mL), were treated at rt with *n*-TBAF (1 M in THF, 264 μL, 264 μmol, 2.0 equiv) and an orange solution was soon obtained. After 1 h the reaction was complete; it was partitioned between H_2_O/EtOAc and extracted with EtOAc. The combined organic layers were washed with brine, dried over Na_2_SO_4_, and concentrated. Chromatography with PE/EtOAc from 4:6 to 1:3 afforded 67 mg of **2v** as a pale yellow solid. Yield 82%. In some instances, little amounts (around 10%) of **2w** can also be isolated. *R*_f_ 0.49 (PE/EtOAc 1:3, A, B). Mp 185.1–187.0 °C (dec.). HPLC purity 98%, *R*_t_ 14.68 min (ACE Excel 3 C18-AR 150 mm × 3 mm, 3 μm column, A: H_2_O/MeCN 9:1 + 0.1% FA to B: MeCN + 0.1% FA in 20 min, flow 0.38 mL/min, temp. 30 °C). ^1^H NMR (400 MHz, MeCN-*d*_3_): δ 8.47 (s, 1H, OH), 7.78 (d, *J* = 6.8 Hz, 1H, CH Ar), 7.71–7.58 (m, 5H, 5 × CH Ar), 7.57 (s, 1H, CH Ar), 7.49 (d, *J* = 7.6 Hz, 1H, CH Ar), 7.20 (s, 1H, CH Ar), 7.13 (t, *J* = 8.1 Hz, 1H, CH Ar), 7.01 (s, 1H, NH), 6.97 (d, *J* = 8.0 Hz, 1H, CH Ar), 6.64 (s, 1H, CH–SO_2_), 6.54 (dd, *J* = 8.1, 2.1 Hz, 1H, CH Ar), 4.68 (d, *J* = 1.9 Hz, 2H, C*H*_2_C=CH), 4.53 (s, 2H, NCH_2_CO), 3.35 (s, 6H, 2 × CH_3_). ^13^C NMR (101 MHz, MeCN-*d*_3_): δ 167.6 (C=O), 165.9 (C=O), 158.3 (C quat. Ar), 144.5 (C quat. Ar), 141.1 (C quat. Ar), 140.6 (C quat. Ar), 139.8 (C quat. Ar), 135.6 (C quat. Ar), 135.5 (C quat. Ar), 134.8 (CH Ar), 133.6 (CH Ar), 131.8 (CH Ar), 131.8 (CH Ar), 131.4 (CH Ar), 130.8 (CH Ar), 130.0 (CH Ar), 124.4 (CH Ar), 122.0 (CH Ar), 116.2 (CH–SO_2_), 111.8 (2 × CH Ar), 107.4 (CH Ar), 56.0 (*C*H_2_C=CH), 54.3 (N*C*H_2_C=O) 3290, 2929, 1654, 1599, 1551, 1487, 1467, 1451, 1364, 1297, 1204, 1149, 1121, 1050. HRMS (ESI^+^) *m*/*z*: [M + H]^+^ Calcd for C_26_H_26_N_3_O_9_S_3_ 620.0826; Found 620.0837.

#### 2-(1,1-Dioxidobenzo­[*b*]­thiophen-3-yl)-*N*-(2-((3-hydroxyphenyl)­amino)-2-oxoethyl)-*N*-(3-(methylsulfonamido)­phenyl)­acetamide **2w**

The reaction was performed following the general procedure starting from 56 mg of **2v**, dissolved in MeOH/MeCN 1:1 (0.7 mL), and the reaction lasted 1 h. An ivory solid (**2w**, 35 mg) was obtained. Yield 72%. *R*_f_ 0.19 (PE/EtOAc 1:3, **A**, **B**). Mp 203.1–205.4 °C. HPLC purity 96%, *R*_t_ 13.66 min (Phenyl C6 150 mm × 3 mm, 3 μm column, A: H_2_O/MeCN 9:1 + 0.1% FA to B: MeCN + 0.1% FA in 20 min, flow 0.34 mL/min, temp. 25 °C). ^1^H NMR (300 MHz, MeCN-*d*_3_ and small amount of MeOH-*d*_4_): δ 7.84–7.75 (m, 1H, CH Ar), 7.70–7.54 (m, 3H, 3 × CH Ar), 7.43 (t, *J* = 8.0 Hz, 1H, CH Ar), 7.31 (s, 1H, CH Ar), 7.29–7.20 (m, 2H, 2 × CH Ar), 7.17–7.08 (m, 2H, 2 × CH Ar), 6.95 (d, *J* = 8.1 Hz, 1H, CH Ar), 6.69 (s, 1H, CH Ar), 6.52 (dd, *J* = 8.0, 2.4 Hz, 1H, CH Ar), 4.67 (d, *J* = 2.3 Hz, 2H, C*H*_*2*_C=CH), 4.50 (s, 2H, NCH_2_C=O), 2.91 (s, 3H, CH_3_). ^13^C NMR (75 MHz, MeCN-*d*_3_ and small amount of MeOH-*d*_4_): δ 167.7 (C=O), 166.0 (C=O), 158.4 (C quat. Ar), 144.3 (C quat. Ar), 140.9 (C quat. Ar), 140.4 (C quat. Ar), 140.0 (C quat. Ar), 138.9 (C quat. Ar), 135.6 (C quat. Ar), 134.8 (CH Ar), 133.3 (CH Ar), 131.4 (CH Ar), 130.6 (CH Ar), 124.2 (CH Ar), 124.0 (CH Ar), 122.0 (CH Ar), 120.5 (CH Ar), 120.3 (CH Ar), 116.8 (CH Ar), 111.7 (CH Ar), 111.6 (CH Ar), 107.4 (CH Ar), 55.9 (*C*H_2_C=CH, very broad), 54.1 (N*C*H_2_C=O), 39.5 (CH_3_). IR (neat): *ν*_max_ (cm^–1^) 3226, 2929, 1654, 1597, 1551, 1493, 1387, 1300, 1209, 1146, 968, 859, 761, 704. HRMS (ESI^+^) *m*/*z*: [M + H]^+^ Calcd for C_25_H_24_N_3_O_7_S_2_ 542.1050; Found 542.1058.

#### *N*-(3-(1*H*-Tetrazol-5-yl)­phenyl)-*N*-(2-((4-cyclohexylphenyl)­amino)-2-oxoethyl)-2-(1,1-dioxidobenzo­[*b*]­thiophen-3-yl)­acetamide **2k**

Compound **2j** (91 mg, 0.143 mmol) was suspended in THF/*t*-BuNH_2_ 1:1 (2.8 mL). Then an aqueous 1 M solution of K_2_CO_3_ (286 μL, 0.286 mmol, 2.0 equiv) was added at rt and, after 1.5 h, a solution was obtained. After 4 h the reaction was complete; it was partitioned between 2 M HCl/EtOAc and extracted multiple times with EtOAc. The combined organic layers were washed with brine, dried over Na_2_SO_4_, and concentrated. Trituration with Et_2_O afforded 71 mg of **2k** as a white solid. Yield 85%. *R*_f_ 0.28 (CH_2_Cl_2_/MeOH 97:3 + 1% AcOH, A, B). Mp 268.0–269.0 °C (dec). HPLC purity 100%, *R*_t_ 9.47 min (Gemini C18 150 mm × 3 mm, 5 μm column, A: H_2_O/MeCN 9:1 + 0.1% FA to B: MeCN + 0.1% FA in 20 min, flow 0.5 mL/min, temp. 24 °C). ^1^H NMR (400 MHz, DMSO-*d*_6_) [NH of tetrazole exchanges with water]: δ 10.11 (s, 1H, NH), 8.14 (s, 1H, CH Ar), 8.04 (d, *J* = 7.7 Hz, 1H, CH Ar), 7.88 (d, *J* = 6.3 Hz, 1H, CH Ar), 7.73–7.60 (m, 4H, 4 × CH Ar), 7.56 (d, *J* = 7.9 Hz, 1H, CH Ar), 7.50 (d, *J* = 8.2 Hz, 2H, 2 × CH para syst.), 7.15 (d, *J* = 8.6 Hz, 2H, 2 × CH para syst.), 6.71 (s, 1H, CH–SO_2_), 4.74 (d, *J* = 2.2 Hz, 2H, C*H*_*2*_C=CH), 4.59 (s, 2H, NCH_2_C=O), 2.47–2.39 (m, 1H, CH *c*-Hex), 1.84–1.62 (m, 5H, 5 × C*H*H *c*-Hex), 1.43–1.17 (m, 5H, 5 × CH*H* *c*-Hex). ^13^C NMR (101 MHz, DMSO-*d*_6_): δ 166.3 (C=O), 164.4 (C=O), 155.1 (C tetrazole), 143.3 (C quat. Ar), 142.7 (C quat. Ar), 139.6 (C quat. Ar), 137.7 (C quat. Ar), 136.6 (C quat. Ar), 134.3 (C quat. Ar), 134.2 (CH Ar), 132.6 (CH Ar), 130.6, (CH Ar), 130.5 (C quat. Ar), 126.9 (2 × CH para syst.), 126.2 (CH Ar), 125.8 and 125.7 (CH Ar and C quat. Ar), 123.3 (CH Ar), 121.4 (CH Ar), 119.2 (2 × CH para syst.), 115.9 (CH–SO_2_), 54.8 (*C*H_2_C=CH), 53.0 (N*C*H_2_C=O), 43.2 (CH *c*-Hex), 34.1 (2 × CH_2_ *c*-Hex), 26.4 (2 × CH_2_ *c*-Hex), 25.6 (CH_2_ *c*-Hex). IR (neat): *ν*_max_ (cm^–1^) 3267, 3092, 2924, 2851, 1663, 1639, 1587, 1548, 1515, 1485, 1467, 1425, 1397, 1300, 1257, 1206, 1147, 1123, 1069, 1011, 960, 900, 867, 816, 771, 742, 723, 708, 647. HRMS (ESI^+^) *m*/*z*: [M + H]^+^ Calcd for C_31_H_31_N_6_O_4_S 583.2122; Found 583.2131.

### Synthesis of Isocyanides

### Isocyanide 3 (Scheme 3)

#### Benzyl (4-Cyclohexylphenyl)­carbamate 20

Acid **19** (500 mg, 2.45 mmol) was suspended in toluene (10 mL) and Et_3_N (410 μL, 2.94 mmol, 1.2 equiv) was added. The resulting solution was heated at 80 °C and DPPA (553 μL, 2.57 mmol, 1.1 equiv) was added dropwise. After 2 h at 80 °C, benzyl alcohol (382 μL, 3.67 mmol, 1.5 equiv) was added and the mixture was refluxed for 2 h. Then the reaction was partitioned between 5% NaHCO_3_ solution/EtOAc. The phases were separated and the aqueous phase was extracted twice with EtOAc. The combined organic layers were washed with brine, dried over Na_2_SO_4_, and concentrated. The residue was triturated with pentane affording **20** (669 mg) as a pale yellow solid. Yield 88%. *R*_f_ 0.59 (PE/EtOAc 8:2, **A**, **C**). M. p.114.6–115.0 °C. ^1^H NMR (300 MHz, CDCl_3_): δ 7.47–7.22 (m, 7H, 7 × CH Ar), 7.19–7.06 (m, 2H, 2 × CH Ar), 6.62 (bs, 1H, NH), 5.19 (s, 2H, CH_2_), 2.58–2.36 (m, 1H, CH *c*-Hex), 1.97–1.67 (m, 5H, 5 × C*H*H *c*-Hex), 1.53–1.17 (m, 5H 5 × CH*H* *c*-Hex). ^13^C NMR (75 MHz, CDCl_3_): δ 153.6 (C=O), 143.7 (Cq. Ar), 136.3 (Cq. Ar), 135.5 (Cq. Ar), 128.7 (CH Ar), 128.5 (CH Ar), 128.4 (CH Ar), 127.5 (CH Ar), 119.0 (CH Ar), 67.1 (CH_2_), 44.0 (CH *c*-Hex), 34.7 (2 × CH_2_ *c*-Hex), 27.0 (2 × CH_2_ *c*-Hex), 26.3 (CH_2_ *c*-Hex). IR (neat): *ν*_max_ (cm^–1^): 3364, 3041, 2923, 2853, 1697, 1615, 1593, 1526, 1452, 1417, 1375, 1357, 1310, 1285, 1228, 1201, 1154, 1134, 1105, 1081, 1066, 1027, 1020, 998, 903, 887, 855, 817, 766, 745, 728, 695, 639, 612.

#### *N*-(4-Cyclohexylphenyl)­formamide **21**

(1) Cbz was removed by hydrogenolysis following the general procedure reported above, starting from **20** (1.259 g, 4.07 mmol), using *i*-PrOH (20 mL) as solvent. The reaction lasted 5 h. After the above-described workup, the crude amine was directly submitted to the next step without further purification. *R*_f_ 0.34 (PE/EtOAc 8:2, **A**, **C**). (2) Following a reported procedure,^20^ to a solution of the crude amine in dry CH_2_Cl_2_ (40 mL) was added formic acid (185 μL, 4.88 mmol, 1.2 equiv) and 4-DMAP (100 mg, 0.801 mmol, 0.2 equiv) under a nitrogen atmosphere at rt. Then the mixture was cooled at 0 °C and DCC (925 mg, 4.48 mmol, 1.2 equiv) was added portion-wise. After stirring for 1 h at rt, the mixture was concentrated and filtered through a Celite pad to remove DCU. The collected solution was concentrated to dryness, and the residue was purified by chromatography (PE/EtOAc 7:3) affording **21** (761 mg) as a white solid. Overall yield: 92%. *R*_f_ 0.24 (PE/EtOAc 7:3, **A**, **B**). Mp 148.5–149.8 °C. ^1^H NMR (300 MHz, CDCl_3_) [two conformers A and B are present in a 52:48 ratio. Fractional NMR integrals are reported to account for the presence of two conformers]: δ 8.62 (d, *J* = 11.5 Hz, 0.52H, A NHC*H*O), 8.36 (d, *J* = 1.8 Hz, 0.48H, B NHC*H*O), 7.51 (bd, *J* = 11.8 Hz, 0.5H, A NH), 7.44 (d, *J* = 8.5 Hz, 1H, A or B 2 × CH para syst.), 7.23–7.15 (m, 2H, A + B 4 × CH para syst.), 7.11 (bs, 0.5 H, B NH), 7.04–6.97 (m, 1H, A or B 2 × CH para syst.), 2.59–2.37 (m, 1H, CH *c*-Hex), 2.00–1.68 (m, 5H, 2 × C*H*H *c*-Hex), 1.49–1.14 (m, 5H, 2 × CH*H* *c*-Hex). ^13^C NMR (75 MHz, CDCl_3_) δ 163.0 and 159.2 (A and B C=O), 145.6 and 144.9 (A and B C quat. Ar), 134.7 and 134.5 (A and B C quat. Ar), 128.1 and 127.4 (A and B CH Ar), 120.3 and 119.3 (A and B CH Ar), 44.1 and 44.0 (A and B CH *c*-Hex), 34.6 (A and B 2 × CH_2_ *c*-Hex), 27.0 and 26.9 (A and B 2 × CH_2_ *c*-Hex), 26.21 and 26.16 (A and B CH_2_ *c*-Hex). IR (neat): *ν*_max_ (cm^–1^) 3049, 2976, 2920, 2849, 1896, 1689, 1612, 1523, 1496, 1448, 1415, 1392, 1315, 1295, 1215, 1202, 1186, 1135, 1107, 1020, 998, 963, 937, 918, 887, 857, 817, 772, 725, 657, 639.

#### 1-Cyclohexyl-4-isocyanobenzene **3**

To a solution of **21** (165 mg, 0.812 mmol) in dry CH_2_Cl_2_ (2.7 mL), under a nitrogen atmosphere, was added Et_3_N (340 μL, 2.44 mmol, 3.0 equiv) at rt. The mixture was cooled at 0 °C and difosgene (76 μL, 0.65 mmol, 0.8 equiv) was added dropwise.^20^ The resulting yellow suspension was stirred at 0 °C for 1 h and then partitioned between 5% NaHCO_3_ solution/CH_2_Cl_2_. The phases were separated and the aqueous phase was extracted with CH_2_Cl_2_. The combined organic layers were washed with brine, dried over Na_2_SO_4_, and concentrated (rotavapor bath at 25 °C). The crude product was rapidly filtered over silica gel (PE/Et_2_O 98:2) affording **3** as a pale green oil (134 mg) (the fractions containing the isocyanide were colorless but, upon concentration to dryness, the liquid became colored), which was used immediately for the subsequent Ugi reaction, without characterization, because of its suspected instability. Yield 89%. *R*_f_ 0.88 (PE/EtOAc 7:3, **A**, **D**).

### Isocyanides **6** and **7** (Scheme 4)

#### Methyl 3-(4-Formylbenzyl)­benzoate **24**

In a round-bottomed flask, under an argon atmosphere, boronic acid **22** (523 mg, 3.49 mmol 1.0), methyl 3-(bromomethyl)­benzoate **23** (800 mg, 3.49 mmol, 1.0 equiv), K_3_PO_4_ (1.85 g, 8.73 mmol, 2.5 equiv), and PdCl_2_(PPh_3_)_2_ (73 mg, 0.104 mmol, 0.03 equiv) were placed, before adding a 10:2 degassed (Ar) solution of 1,4-dioxane/H_2_O (20 mL). After heating at 80 °C for 1 h, the crude mixture was filtered over a Celite pad, which was washed with 1,4-dioxane. After concentration at reduced pressure the residue was partitioned between H_2_O/EtOAc and extracted multiple times with EtOAc. The combined organic layers were washed with brine, dried over Na_2_SO_4_, and concentrated to dryness. The residue was purified by chromatography (PE/EtOAc 9:1), affording **24** as a colorless oil (834 mg). Yield 94%. *R*_f_ 0.25 (PE/EtOAc 9:1, **A**). ^1^H NMR (300 MHz, CDCl_3_): δ 9.94 (s, 1H, CHO), 7.95–7.86 (m, 2H, 2 × CH Ar), 7.79 (d, *J* = 8.2 Hz, 2H, 2 × CH para syst.), 7.41–7.29 (m, 4H, 2 × CH Ar + 2 × CH para syst.), 4.07 (s, 2H, CH_2_), 3.87 (s, 3H, CH_3_). ^13^C NMR (75 MHz, CDCl_3_): δ 191.7 (CHO), 166.8 (*C*O_2_CH_3_), 147.6 (C quat. Ar), 140.1 (C quat. Ar), 134.7 (C quat. Ar), 133.5 (CH Ar), 130.5 (CH Ar), 130.0 (2 × CH para syst.), 129.9 (CH Ar), 129.4 (2 × CH para syst.), 128.7 (CH Ar), 127.7 (CH Ar), 52.0 (CH_3_), 41.7 (CH_2_). IR (neat): *ν*_max_ (cm^–1^) 2951, 2841, 2559, 1719, 1681, 1603, 1575, 1488, 1429, 1321, 1287, 1264, 1195, 1182, 1169, 1128, 1099, 1084, 1019, 989, 967, 939, 921, 879, 863, 836, 821, 797, 744, 705, 689, 671, 637.

#### Methyl 3-(4-(((Benzyloxy)­carbonyl)­amino)­benzyl)­benzoate **26**

(1) Compound **24** (784 mg, 3.20 mmol) was dissolved in THF (16 mL), and 2-methyl-2-butene (5.1 mL, 48.0 mmol, 15 equiv) was added at 0 °C. Then a solution of NaClO_2_ (867 mg, 9.60 mmol, 3.0 equiv) and NaH_2_PO_4_ (1.33 g, 9.60 mmol, 3.0 equiv) in H_2_O (7 mL) was added dropwise through a Pasteur pipet. The resulting mixture was stirred for 2 h at rt. Volatile components were removed under reduced pressure, and the residue was partitioned between H_2_O/EtOAc and extracted with EtOAc. The combined organic layers were washed with brine, dried over Na_2_SO_4_, filtered, and concentrated to dryness affording **25** which was used directly in the next step without further purification. *R*_f_ 0.49 (PE/EtOAc 8:2, **A**). (2) The same procedure used for the transformation of **19** into **20** was used, starting from crude **25** (3.20 mmol). The crude product was purified by chromatography (PE/CH_2_Cl_2_/Et_2_O 6:4:0.5) affording **26** (670 mg) as a pale yellow oil. Overall yield form **24**: 58%. *R*_f_ 0.30 (PE/EtOAc 8:2, **A**, **B**). ^1^H NMR (300 MHz, CDCl_3_): δ 7.91–7.80 (m, 2H, 2 × CH Ar), 7.40–7.24 (m, 7 × CH Ar + 2 × CH para syst.), 7.07 (d, *J* = 8.4 Hz, 2H, 2 × CH para syst.), 6.98 (bs, 1H, NH), 5.14 (s, 2H, OCH_2_), 3.92 (s, 2H, CH_2_), 3.85 (s, 3H, CH_3_); ^13^C NMR (75 MHz, CDCl_3_): δ 167.2 (*C*O_2_CH_3_), 153.5 (CONH), 141.6 (C quat. Ar), 136.2 (C quat. Ar), 136.1 (C quat. Ar), 135.6 (C quat. Ar), 133.5 (CH Ar), 130.3 (C quat. Ar), 129.9 (CH Ar), 129.5 (2 × CH para syst.), 128.6 (2 × CH para syst.), 128.30 (CH Ar), 128.27 (2 × CH Ar), 127.5 (CH Ar), 119.1 (CH Ar), 66.9 (OCH_2_), 52.1 (OCH_3_), 41.0 (CH_2_). IR (neat): *ν*_max_ (cm^–1^) 3314, 2952, 1734, 1700, 1599, 1537, 1514, 1455, 1432, 1413, 1309, 1293, 1273, 1223, 1208, 1106, 1083, 1064 1029, 1016, 976, 912, 840, 810, 787, 758, 734, 692, 615.

#### 3-(4-Formamidobenzyl)-*N*-methylbenzamide **30**

(1) A solution of **26** (1.398 g, 3.72 mmol) in THF (18.6 mL) was treated with LiOH solution (1 M, 9.3 mL, 9.3 mmol, 2.5 equiv), and the initial solution became cloudy. After 24 h the reaction was still incomplete and another portion of LiOH solution (1 M, 1.9 mml, 1.9 mmol, 0.5 equiv) was added, and the reaction was complete after 1 h. The crude mixture was concentrated under reduced pressure and partitioned between H_2_O/EtOAc. After adjusting the pH to 3 with 2 M HCl the extraction was performed with EtOAc. The combined organic layers were washed with brine, dried over Na_2_SO_4_, filtered, and concentrated to dryness affording **27** as a pale yellow solid which was used directly in the next step without further purification. *R*_f_ 0.29 (PE/EtOAc 1:1 + 1% AcOH, **A**, **B**). (2) **27** (1.72 mmol, 46% of the above obtained crude product) was dissolved in dry THF (15 mL) under a nitrogen atmosphere. Then CDI (444 mg, 2.74 mmol, 1.6 equiv) was added and the reaction was stirred at rt overnight until **27** was consumed (TLC PE/EtOAc 6:4 + 1% AcOH). Then methylamine hydrochloride (289 mg, 4.28 mmol, 2.5 equiv) was added followed by DBU (897 μL, 6.00 mmol, 3.5 equiv) and the reaction was stirred for about 24 h. The mixture was diluted with 5% (NH_4_)­H_2_PO_4_ and was extracted with EtOAc. The combined organic layers were washed with brine, dried over Na_2_SO_4_, filtered, and concentrated to dryness affording **28** which was used directly in the next step without further purification. *R*_f_ 0.25 (PE/EtOAc 1:1 + 1% EtOH, **A**, **B**). (3) a. Cbz was removed by hydrogenolysis following the general procedure reported above, starting from crude **28**, using MeOH (8 mL) as solvent and Pd/C 10% (20% w/w). The reaction was stirred overnight. After the above-described workup, the crude amine was directly submitted to the next step without further purification. *R*_f(amine)_ 0.30 (PE/EtOAc 2:8 + 1% EtOH, **A**, **B**). b. The crude amine (1.72 mmol) was dissolved in ethyl formate (10 mL, 124 mmol, 72 equiv) and refluxed for about 30 h. After solvent removal the residue was purified by chromatography (PE/EtOAc 1:9), affording **30** as a pale yellow viscous oil (340 mg). Overall yield form **26**: 74%. *R*_f_ 0.18 (PE/EtOAc 2:8 + 1% EtOH, **A**, **B**). ^1^H NMR (400 MHz, CDCl_3_) [two conformers A and B are present in a 56:44 ratio. Fractional NMR integrals are reported to account for the presence of two conformers]: δ 8.62 (d, *J* = 11.5 Hz, 0.4H, B NHC*H*O), 8.33 (d, *J* = 1.9 Hz, 0.6H, A NHC*H*O), 7.63–7.60 (m, 0.5H, A or B CH Ar), 7.59–7.53 (m, 2H, A + B CH Ar + B N*H*CHO), 7.44 (d, *J* = 8.5 Hz, 1H, A or B 2 × CH para syst.), 7.37–7.29 (m, 2H, A + B Ar + A N*H*CHO), 7.16 (d, *J* = 8.2 Hz, 1H, A or B 2 × CH para syst.), 7.13 (d, *J* = 7.9 Hz, 1H, A or B 2 × CH para syst.), 6.99 (d, *J* = 8.4 Hz, 1H, A or B 2 × CH para syst.), 6.17 (bs, 1H, A+B N*H*CH_3_), 3.99 (s, 0.9H, B CH_2_), 3.97 (s, 1.1H, A CH_2_), 3.00 (d, *J* = 3.1 Hz, 1.5H, B CH_3_), 2.99 (d, *J* = 3.1 Hz, 1.7H, A CH_3_). ^13^C NMR (101 MHz, CDCl_3_): δ 168.5 and 168.4 (A and B *C*ONHCH_3_), 162.4 (B CHO), 159.1 (A CHO), 141.7 (A or B C quat. Ar), 141.5 (A or B C quat. Ar), 137.9 (A or B C quat. Ar), 137.2 (A or B C quat. Ar), 135.3 (A or B C quat. Ar), 135.1 (A or B C quat. Ar), 135.1 (A or B C quat. Ar), 135.0 (A or B C quat. Ar), 132.1 (A or B CH Ar), 132.0 (A or B CH Ar), 130.4 (A or B 2 × CH para syst.), 129.7 (A or B 2 × CH para syst.), 128.94 (A or B CH Ar), 128.89 (A or B CH Ar), 127.7 (A or B CH Ar), 127.5 (A or B CH Ar), 124.7 (A or B CH Ar), 124.6 (A or B CH Ar), 120.5 (A or B 2 × CH para syst.), 119.5 (A or B 2 × CH para syst.), 41.3 and 41.2 (A and B CH_2_), 27.00 and 26.98 (A and B CH_3_). IR (neat): *ν*_max_ (cm^–1^) 3270, 3058 2874, 2246, 1681, 1634, 1605, 1583, 1536, 1515, 1482, 1439, 1410, 1300, 1214, 1183, 1154, 1019, 906, 843, 811, 725, 669, 645.

#### 3-(4-Isocyanobenzyl)-*N*-methylbenzamide **6**

A solution of **30** (172 mg, 0.64 mmol) in dry CH_2_Cl_2_ (6.5 mL), under a nitrogen atmosphere, was cooled to 0 °C. Then Et_3_N (304 μL, 2.18 mmol, 3.4 equiv) was added, followed by dropwise addition of POCl_3_ (84 μL, 0.90 mmol, 1.4 equiv). The reaction lasted 4 h at 0 °C. Then it was diluted with 5% NaHCO_3_ solution and extracted with CH_2_Cl_2_. The combined organic layers were washed with brine, dried over Na_2_SO_4_, and concentrated (98 mg), which was used immediately for the subsequent Ugi reaction, without characterization, because of its suspected instability. Yield 61%. *R*_f_ 0.46 (PE/EtOAc 3:7 + 2% EtOH, **A**, **D**).

#### *N*-Cyclopropyl-3-(4-formamidobenzyl)­benzamide **31**

(1) The preparation is described for the synthesis of **30** and this time 54% of the crude **26** was used in the following steps. (2) The same procedure described for the synthesis of **28** was used, starting from a solution of crude **26** (2.01 mmol) in THF (18 mL). Reagents: CDI (522 mg, 3.22 mmol, 1.6 equiv), cyclopropylamine (279 μL, 4.02 mmol, 2.0 equiv), Et_3_N (561 μL, 4.02 mmol, 2.0 equiv). (3) Cbz removal and formamide **31** synthesis followed the procedure already described for **30**. Chromatography (PE/EtOAc 3:7 + 2% EtOH), afforded **31** as a white foam (461 mg). Overall yield form **26**: 83%. *R*_f(amine)_ 0.42 (PE/EtOAc 3:7 + 2% EtOH, **A**, **B**, **C**). *R*_f(**31**)_ 0.27 (PE/EtOAc 3:7 + 2% EtOH, **A**, **B**, **C**). ^1^H NMR (300 MHz, CDCl_3_) [two conformers A and B are present in a 54:46 ratio. Fractional NMR integrals are reported to account for the presence of two conformers]: δ 8.62 (d, *J* = 11.5 Hz, 0.5H, B NHC*H*O), 8.35 (d, *J* = 1.8 Hz, 0.5H, B NHC*H*O), 7.63–7.57 (m, 1H, A + B CH Ar), 7.56–7.50 (m, 1H, A + B CH Ar), 7.45 (d, *J* = 8.5 Hz, 1H, A or B 2 × CH para syst.), 7.38–7.29 (m, 2H, A + B CH Ar), 7.21–7.09 (m, 2H, A + B 4 × CH para syst.), 6.99 (d, *J* = 8.4 Hz, 1H, A or B 2 × CH para syst.), 6.20 (bs, 1H, N*Hc*Pr), 3.99 (s, 1.1H, B CH_2_), 3.98 (s, 1.4H, B CH_2_), 2.88 (tq, *J* = 6.8, 3.5 Hz, 1H, CH *c*Pr), 0.91–0.82 (m, 2H, C*H*H of *c*Pr), 0.65–0.57 (m, 2H, CH*H* of *c*Pr). ^13^C NMR (75 MHz, CDCl_3_): δ 169.6 and 169.4 (A and B *C*ONHCH_3_), 162.7 and 159.8 (A and B CHO), 141.6 (A or B C quat. Ar), 141.4 (A or B C quat. Ar), 137.5 (A or B C quat. Ar), 136.8 (A or B C quat. Ar), 135.6 (A or B C quat. Ar), 135.2 (A or B C quat. Ar), 134.6 (A or B C quat. Ar), 134.5 (A or B C quat. Ar), 132.1 (A or B CH Ar), 132.0 (A or B CH Ar), 130.0 (A or B 2 × CH para syst.), 129.3 (A or B 2 × CH para syst.), 128.7 (A or B CH Ar), 128.6 (A or B CH Ar), 127.7 (A or B CH Ar), 127.6 (A or B CH Ar), 124.94 (A or B CH Ar), 124.89 (A or B CH Ar), 120.5 (A or B 2 × CH para syst.), 119.1 (A or B 2 × CH para syst.), 41.1 and 41.0 (A and B CH_2_), 23.3 (CH *c*Pr), 6.6 and 6.5 (CH_2_ *c*Pr). IR (neat): *ν*_max_ (cm^–1^) 3255, 3017, 2922, 2861, 1664, 1632, 1607, 1579, 1516, 1481, 1436, 1411, 1388, 1365, 1308, 1238, 1182, 1138, 1112, 1049, 1032, 975, 907, 849, 807, 784, 761, 745, 693, 642, 609.

#### *N*-Cyclopropyl-3-(4-isocyanobenzyl)­benzamide **7**

The same procedure described for the synthesis of **6** was used, starting from **31** (132 mg, 0.49 mmol). The combined organic layers were washed with brine, dried over Na_2_SO_4_, and concentrated to dryness. The crude product was rapidly filtered over silica gel (PE/EtOAc 3:7) affording **7** as a white solid (74 mg), which was used immediately for the subsequent Ugi reaction, without characterization, because of its suspected instability. Yield 60%. *R*_f_ 0.71 (PE/EtOAc 3:7 + 2% EtOH, **A**, **D**).

### Isocyanides **9** and **10** (Scheme 5)

### *N*-(3-(Trifluoromethoxy)­phenyl)­formamide **33**

A solution of formic acid (102 μL, 2.71 mmol, 1.2 equiv) in dry CH_2_Cl_2_ (25 mL), under a nitrogen atmosphere, was treated with CDI (586 mg, 3.61 mmol, 1.6 equiv) and stirred at rt for 5 h. Then **32** (400 mg, 2.26 mmol) and Et_3_N (631 μL, 4.52 mmol, 2.0 equiv) were added. After 18 h saturated NH_4_Cl solution was added, and the extraction was performed with CH_2_Cl_2_. The combined organic layers were washed with brine, dried over Na_2_SO_4_, and concentrated to dryness. The crude product was purified by chromatography (PE/EtOAc 2:1) affording **33** (352 mg) as a white solid. Yield: 76%. *R*_f_ 0.24 (PE/EtOAc 2:1, **A**, **B**). Mp 70.9–72.1 °C. ^1^H NMR (300 MHz, CDCl_3_) [two conformers A and B are present in a 56:44 ratio. Fractional NMR integrals are reported to account for the presence of two conformers]: δ 8.74 (d, *J* = 11.1 Hz, 0.4H, B CHO), 8.65 (bd, *J* = 10.8 Hz, 0.4H, B NH), 8.40 (d, *J* = 1.8 Hz, 0.6H, A CHO), 7.72 (s, 0.6H, A NH), 7.58 (s, 0.5H, A or B CH Ar), 7.47–7.30 (m, 1.5H, A+B CH Ar), 7.12–6.94 (m, 2H, A + B CH Ar). ^13^C NMR (75 MHz, CDCl_3_): δ 162.6 and 159.3 (CHO), 150.2 and 149.7 (C–O), 138.4 and 138.3 (C–N), 131.2 (A or B CH Ar), 130.3 (A or B CH Ar), 120.51 (q, *J* = 257.7 Hz, CF_3_), 118.0 (A or B CH Ar), 117.4 (A or B CH Ar), 117.1 (A or B CH Ar), 116.8 (A or B CH Ar), 113.0 (A or B CH Ar), 111.4 (A or B CH Ar). IR (neat): *ν*_max_ (cm^–1^) 3260, 3203, 3143, 3086, 1940, 1695, 1667, 1609, 1556, 1489, 1470, 1441, 1423, 1401, 1322, 1282, 1250, 1208, 1199, 1148, 1083, 1000, 980, 878, 832, 790, 771, 747, 699, 677, 631, 624.

#### 1-Isocyano-3-(trifluoromethoxy)­benzene **9**

The same procedure described for the synthesis of **3** was used, starting from **33** (271 mg, 1.32 mmol). The only difference is that the addition of diphosgene was performed at −15 °C and the temperature was allowed to reach 0 °C during 1 h. After completion of the reaction and workup, the crude product was rapidly filtered over silica gel (PE/CH_2_Cl_2_ 1:1) affording **9** as a blue-greenish liquid (188 mg) (the fractions containing the isocyanide were colorless but, upon concentration to dryness, the liquid became colored), which was used immediately for the subsequent Ugi reaction, without characterization, because of its suspected instability. Yield 76%. *R*_f_ 0.43 (PE/CH_2_Cl_2_ 7:3, the spot is very difficult to detect, **A**, **B**).

#### 3-((*tert-*Butyldimethylsilyl)­oxy)­aniline **35**

This compound was prepared from **34** (500 mg, 4.58 mmol) following an already reported procedure, using CH_2_Cl_2_ (15 mL) as solvent instead, affording **35** (1.02 g, 100% yield), whose spectroscopic data are consistent with those reported in the literature yield.^42^

#### *N*-(3-((*tert-*Butyldimethylsilyl)­oxy)­phenyl)­formamide **36**

The same procedure used for **21** was followed, starting from **35** (1.02 g). Chromatography (PE/EtOAc 8:2) afforded **36** (930 mg) as a pale-yellow oil. Yield: 81%. *R*_f_ 0.26 (PE/EtOAc 8:2, **A**, **B**).^1^H NMR (300 MHz, CDCl_3_) [two conformers A and B are present in a 55:45 ratio. Fractional NMR integrals are reported to account for the presence of two conformers]: δ 8.67 (d, *J* = 11.3 Hz, 0.55H A CHO), 8.35 (d, *J* = 1.9 Hz, 0.45H, B CHO), 8.22 (s, 0.5H, A NH), 7.36 (s, 0.5H, B NH), 7.247.01 (m, 2H, 2 × CH Ar), 6.746.51 (m, 2H, 2 × CH Ar), 0.99 (s, 4.5H, A or B 3 × CH_3_ of *t*Bu), 0.98 (s, 4.5H, A or B 3 × CH_3_ of *t*Bu), 0.21 (s, 3H, A or B 2 × Si-CH_3_), 0.21 (s, 3H, 2 × Si-CH_3_). ^13^C NMR (75 MHz, CDCl_3_): δ 162.7 and 159.0 (CHO), 157.0 and 156.4 (C–O), 138.04 and 137.99 (C–N), 130.6 (A or B CH Ar), 129.9 (A or B CH Ar), 117.0 (A or B CH Ar), 116.6 (A or B CH Ar), 112.8 (A or B CH Ar), 112.1 (A or B CH Ar), 111.7 (A or B CH Ar), 110.8 (A or B CH Ar), 25.79 and 25.75 (3 × CH_3_ of *t*Bu), 18.3 (C quat. of *t*Bu), −4.30 and −4.28 (2 × Si-CH_3_). IR (neat): *ν*_max_ (cm^–1^) 3324, 2952, 2933, 2883, 2857, 1684, 1670, 1596, 1541, 1472, 1440, 1361, 1318, 1295, 1253, 1225, 1163, 1126, 1004, 990, 937, 867, 856, 837, 779, 735, 697, 683, 665, 621.

#### *tert*-Butyl­(3-isocyanophenoxy)­dimethylsilane **10**

The same procedure described for the synthesis of **3** was used, starting from **36** (350 mg, 1.39 mmol). The only difference is that the addition of diphosgene was performed at −15 °C and the temperature was allowed to reach 0 °C during 1 h. After completion of the reaction and workup, the crude product was rapidly filtered over silica gel (PE/CH_2_Cl_2_ 7:3) affording **10** as a yellowish-green oil (260 mg) (the fractions containing the isocyanide were colorless but, upon concentration to dryness, the liquid became colored), which was used immediately for the subsequent Ugi reaction, without characterization, because of its suspected instability. Yield 80%. *R*_f_ 0.44 (PE/CH_2_Cl_2_ 7:3, **A**, **B**).

### Synthesis of Amines

#### *N*-(3-Aminophenyl)-*N*-(methylsulfonyl)­methanesulfonamide **14** (Scheme 6)

(1) To a solution of 3-nitroaniline **37** (500 mg, 3.62 mmol) in dry CH_2_Cl_2_ (20 mL), under a nitrogen atmosphere, Et_3_N (1.57 mL, 11.22 mmol, 3.1 equiv) was added. After cooling to 0 °C, MeSO_2_Cl (588 μL, 7.60 mmol, 2.1 equiv) was added. The reaction was stirred at rt for 2 h, then it was partitioned between H_2_O/CH_2_Cl_2_ and extracted with CH_2_Cl_2_. The combined organic layers were washed with brine, dried over Na_2_SO_4_, and concentrated to dryness affording crude **38**, used as such without further purification. *R*_f_ 0.55 (PE/EtOAc 6:4, **A**). (2) The nitro group of crude **38** (3.62 mmol) was hydrogenated using the general procedure reported above, using MeOH (8 mL) as solvent and Pd/C 10% (20% w/w). The reaction was stirred for 18 h. After the above-described workup crude **14** was purified by trituration with Et_2_O affording a pale-yellow solid (708 mg). Overall yield form **37**: 74%. *R*_f_ 0.26 (PE/EtOAc 6:4, **A**, **D**). Mp 165 °C (dec). ^1^H NMR (300 MHz, DMSO-*d*_6_): δ 7.10 (t, *J* = 7.8 Hz, 1H, CH Ar), 6.73–6.56 (m, 3H, 3 × CH Ar), 5.49 (bs, 2H, NH_2_), 3.47 (s, 6H, 2 × CH_3_). ^13^C NMR (75 MHz, DMSO-*d*_6_): δ 149.4 (C–N), 134.4 (C–N), 129.6 (CH Ar), 118.0 (CH Ar), 115.9 (CH Ar), 115.5 (CH Ar), 42.9 (2 × CH_3_). IR (neat): *ν*_max_ (cm^–1^) 3479, 3387, 3224, 3038, 3013, 2931, 1622, 1607, 1585, 1496, 1465, 1414, 1359, 1340, 1326, 1292, 1152, 997, 980, 969, 926, 880, 856, 844, 784, 757, 692, 661, 603.

### Amine **15** (Scheme 7)

#### *N*-(2-Cyanoethyl)-3-nitrobenzamide 41

(1) Method A: To a solution of **39** (400 mg, 2.39 mmol) in dry DMF (15 mL), under a nitrogen atmosphere, was added HOBT (505 mg, 2.63 mmol, 1.1 equiv) at rt. Upon addition of EDC hydrochloride (355 mg, 2.63 mmol, 1.1 equiv), a suspension formed, which redissolved after 5 min. Finally, 3-aminopropanenitrile (193 μL, 2.63 mmol, 1.1. equiv) was added and the mixture was stirred overnight. The solution was diluted with EtOAc and washed with 5% aqueous LiCl solution (5 times). The aqueous phase was extracted with EtOAc. The combined organic layers were washed with brine, dried over Na_2_SO_4_, and concentrated to dryness. The crude product was purified by chromatography (PE/EtOAc 4:6) affording **41** (497 mg) as a white solid. Yield: 95%. *R*_f_ 0.32 (PE/EtOAc 4:6, **A**). Mp 106.0–107.0 °C. ^1^H NMR (300 MHz, MeOH-*d*_4_): δ 9.16 (bs, 1H, NH), 8.73 (t, *J* = 1.9 Hz, 1H, 2-CH Ar), 8.40 (ddd, *J* = 8.2, 2.3, 1.1 Hz, 1H, 4-CH Ar), 8.24 (ddd, *J* = 7.8, 1.7, 1.1 Hz, 1H, 6-CH Ar), 7.72 (t, *J* = 8.0 Hz, 1H, 5-CH Ar), 3.69 (td, *J* = 6.6, 2.4 Hz, 2H, C*H*_*2*_CH_2_CN), 2.83 (t, *J* = 6.6 Hz, 2H, CH_2_C*H_2_* CN). ^13^C NMR (75 MHz, MeOH-*d*_4_): δ 167.4 (C=O), 149.2 (C-NO_2_), 136.3 (*C*–C=O), 134.1 (6-CH Ar), 130.7 (5-CH Ar), 127.0 (4-CH Ar), 123.1 (2-CH Ar), 119.0 (CN), 36.9 (*C*H_2_CH_2_CN), 18.2 (CH_2_ *C*H_2_CN). IR (neat): *ν*_max_ (cm^–1^) 3337, 3097, 2962, 2876, 2255, 1980, 1925, 1816, 1750, 1640, 1619, 1555, 1528, 1479, 1447, 1417, 1351, 1325, 1307, 1273, 1248, 1216, 1185, 1164, 1102, 1071, 1042, 1004, 994, 939, 909, 877, 840, 813, 803, 774, 708, 670, 651, 627. (2) Method B: a. To a suspension of **39** (500 mg, 2.99 mmol) in dry CH_2_Cl_2_ (30 mL), under a nitrogen atmosphere and cooled to 0 °C, were added oxalyl chloride (835 μL, 5.98 mmol, 2.0 equiv) and DMF (7 μL, 0.090 mmol, 0.03 equiv), and the resulting solution was stirred at rt for 4 h. Volatile components were removed under reduced pressure by placing a linear trap filled with soda lime before the membrane pump. The white solid residue *R*_f_ 0.81 (PE/EtOAc 4:6, **A**, **B**) was washed with dry CH_2_Cl_2_ (3 × 5 mL), removing the solvent after each wash. b. Crude **40** was suspended in dry CH_2_Cl_2_ (30 mL); Et_3_N (835 μL, 5.98 mmol, 2 quiv.) and 3-aminopropanenitrile (286 μL, 3.89 mmol, 1.3 equiv) were added at rt and the solution was stirred for 18 h. The mixture was concentrated at reduced pressure; then it was poured into 5% NaHCO_3_ and an extraction with EtOAc was performed. The crude product was purified as described in Method A, affording **41** (602 mg). Overall yield form **39**: 92%. (3) Method C: a. Crude **40** (6.02 mmol), prepared following Method B (step a.) from 1.01 g of **39**, was suspended in dry CH_2_Cl_2_ (40 mL), under a nitrogen atmosphere; β-alanine methyl ester hydrochloride (1.09 g, 7.83 mmol, 1.3 equiv) and Et_3_N (2.52 mL, 18.1 mmol, 3.0 quiv.) were added (286 μL, 3.89 mmol, 1.3 equiv) at rt, and the solution was stirred for overnight. The mixture was concentrated at reduced pressure; then it was poured into 5% NaHCO_3_ solution (pH ≈ 9) and a first extraction with EtOAc was performed. Then the pH of the aqueous phase was adjusted to ≈7 by addition of saturated NH_4_Cl solution, and the extraction was completed. The combined organic layers were washed with brine, dried over Na_2_SO_4_, and concentrated to dryness. The crude product was purified by chromatography (PE/EtOAc 4:6) affording methyl 3-(3-nitrobenzamido)­propanoate **42**, as a pale-yellow oil. Overall yield form **39**: 94%. *R*_f_ 0.47 (PE/EtOAc 4:6, **A**, **D**). ^1^H NMR (400 MHz, CDCl_3_): δ 8.61 (td, *J* = 1.9, 0.4 Hz, 1H, CH Ar), 8.36 (ddd, *J* = 8.2, 2.3, 1.1 Hz, 1H, CH Ar), 8.13 (ddd, *J* = 7.7, 1.7, 1.1 Hz, 1H, CH Ar), 7.65 (ddd, *J* = 8.2, 7.7, 0.5 Hz, 1H, CH Ar), 7.07 (bs, 1H, NH), 3.80–3.74 (m, 2H, C*H* *_2_* NH), 3.74 (s, 3H, OCH_3_), 2.74–2.64 (m, 2H, C*H_2_* CO_2_CH_3_). ^13^C NMR (101 MHz, CDCl_3_): δ 173.4 (C=O), 165.1 (C=O), 148.4 (C quat. Ar), 136.2 (C quat. Ar), 133.1 (CH Ar), 130.0 (CH Ar), 126.2 (CH Ar), 122.1 (CH Ar), 52.1 (CH_3_), 35.7 (CH_2_NH), 33.6 (*C*H_2_CO_2_CH_3_). IR (neat): *ν*_max_ (cm^–1^) 3319, 3086, 2954, 1732, 1643, 1617, 1524, 1476, 1438, 1349, 1323, 1304, 1270, 1198, 1178, 1160, 1078, 1013, 906, 845, 817, 773, 716, 672, 650. b. Compound **42** (1.31 g, 5.18 mmol) was placed in a cylindrical closed vessel along with a ≈7 M solution of NH_3_ in MeOH (8.9 mL, 62.3 mmol, ≈12 equiv). The solution was heated at 80 °C overnight and finally concentrated under reduced pressure affording a white solid. Crude *N*-(3-amino-3-oxopropyl)-3-nitrobenzamide **43** was directly used in the following step without purification. *R*_f_ 0.04 (EtOAc, **A**, **D**). c. A solution of crude **43** (5.18 mmol) was dissolved in dry pyridine (14 mL), under a nitrogen atmosphere, and was cooled to −15 °C, followed by the slow addition of POCl_3_ (1.3 mL, 14.1 mmol, 2.7 equiv), and the solution was stirred for 1.5 h.^43^ Then it was partitioned between H_2_O/EtOAc and extracted with EtOAc. The combined organic layers were washed with 1 M HCl, 5% NaHCO_3_ and brine, dried over Na_2_SO_4_, and concentrated to dryness. The crude product was purified as described in Method A, affording **41** (795 mg). Overall yield form **42**: 70%.

#### *tert*-Butyl (3-((2-cyanoethyl)­carbamoyl)­phenyl)­carbamate **45**

(1) a. To a solution of **41** (595 mg, 2.71 mmol) in EtOH (27 mL) a solution of NH_4_Cl (580 mg, 10.8 mmol, 4.0 equiv) in H_2_O (5.8 mL) and iron powder (1.21 g, 21.7 mmol, 8.0 equiv) were added. The mixture was vigorously stirred at 70 °C for 2 h, then filtered through a Celite pad using MeOH for washing, and the solvent was removed under reduced pressure. The residue was partitioned between H_2_O/EtOAc and extracted with EtOAc. The combined organic layers were washed with brine, dried over Na_2_SO_4_, and concentrated to dryness affording crude **44**, used as such without further purification. *R*_f_ 0.20 (PE/EtOAc 3:7, **A**, **C**). b. Crude **44** (2.71 mmol) was dissolved in dry 1,4-dioxane (27 mL) under a nitrogen atmosphere, then Boc_2_O (1.9 mL, 8.13 mmol, 3.0 equiv) was added. After 16 h at 70 °C, the volatile components were removed under reduced pressure, and the crude yellow solid was kept under high vacuum overnight. The crude product was triturated with Et_2_O affording **45** (752 mg) as a pale-yellow solid. Overall yield form **41**: 96%. *R*_f_ 0.44 (PE/EtOAc 3:7, **A**, **B**). Mp 139.9–141.0 °C. ^1^H NMR (400 MHz, CDCl_3_): δ 7.85 (t, *J* = 2.0 Hz, 1H, CH Ar), 7.52 (ddd, *J* = 8.1, 2.3, 1.1 Hz, 1H, CH Ar), 7.44 (dt, *J* = 7.8, 1.3 Hz, 1H, CH Ar), 7.33 (t, *J* = 7.9 Hz, 1H, CH Ar), 7.00 (t, *J* = 6.2 Hz, 1H, N*H*CH_2_), 6.86 (s, 1H, NHBoc), 3.68 (q, *J* = 6.3 Hz, 2H, NHC*H_2_*), 2.73 (t, *J* = 6.4 Hz, 2H, CH_2_CN), 1.52 (s, 9H, 3 × CH_3_). ^13^C NMR (101 MHz, CDCl_3_): δ 167.9 (C=O), 152.9 (C=O), 139.0, (C quat. Ar), 134.5, (C quat. Ar), 129.5 (CH Ar), 122.0 (CH Ar), 121.8 (CH Ar), 118.4 (CN), 117.0 (CH Ar), 81.2 (C quat. of *t*Bu), 36.4 (NHCH_2_), 28.4 (3 × CH_3_), 18.5 (*C*H_2_CN). IR (neat): *ν*_max_ (cm^–1^) 3351, 3070, 3025, 2999, 2972, 2936, 2255, 1720, 1603, 1595, 1559, 1519, 1478, 1467, 1436, 1413, 1391, 1368, 1363, 1321, 1285, 1262, 1232, 1200, 1155, 1117, 1050, 1026, 998, 939, 926, 879, 856, 791, 772, 757, 747, 722, 711, 686, 678, 668, 632.

#### *tert*-Butyl (3-(1-(2-cyanoethyl)-1*H*-tetrazol-5-yl)­phenyl)­carbamate **46**

To a solution of **45** (750 mg, 2.59 mmol) in dry THF (26 mL), under a nitrogen atmosphere, PPh_3_ (1.36 g, 5.18 mmol, 2.0 equiv) was added. The mixture was cooled to 0 °C and DIAD (1.02 mL, 5.18 mmol, 2.0 equiv) was added. After 30 min at rt, TMSN_3_ (684 μL, 5.18 mmol, 2.0 equiv) was added and the mixture was stirred at rt for 3 days. The mixture was poured in freshly prepared 5% NaHCO_3_ solution and extracted with EtOAc. The combined organic layers were washed with brine, dried over Na_2_SO_4_, and concentrated to dryness. The crude product was purified by chromatography (PE/EtOAc 6:4) affording **46** (495 mg) as a pale-yellow solid. Further elution with PE/EtOAc 1:1, afforded unreacted **45** (155 mg, 21%). Yield 61% (77% on unrecovered **45**). *R*_f_ 0.30 (PE/EtOAc 1:1, **A**, **B**). Mp 130.0–131.4 °C.^1^H NMR (300 MHz, MeOH-*d*_4_): δ 7.91 (t, *J* = 1.8 Hz, 1H, CH Ar), 7.63 (ddd, *J* = 8.2, 2.1, 1.1 Hz, 1H, CH Ar), 7.51 (t, *J* = 7.9 Hz, 1H, CH Ar), 7.38 (dt, *J* = 7.6, 1.3 Hz, 1H, CH Ar), 4.80 (t, *J* = 6.6 Hz, 2H, NCH_2_), 3.22 (t, *J* = 6.6 Hz, 2H, CH_2_CN), 1.53 (s, 9H, 3 × CH_3_). ^13^C NMR (75 MHz, MeOH-*d*_4_): δ 156.3 and 155.0 (C=O and Cq. tetrazole), 141.9 (C–NH_2_), 131.0 (CH Ar), 124.8 (C-tetrazole), 123.9 (CH Ar), 122.4 (CH Ar), 119.7 (CH Ar), 117.8 (CN), 81.3 (*C*(CH_3_)_3_), 45.0 (NCH_2_), 28.6 (3 × CH_3_), 18.8 (*C*H_2_CN). IR (neat): *ν*_max_ (cm^–1^) 3351, 3070, 3025, 2999, 2972, 2936, 2255, 1720, 1603, 1595, 1559, 1519, 1478, 1467, 1436, 1413, 1391, 1368, 1363, 1321, 1285, 1262, 1232, 1200, 1155, 1117, 1050, 1026, 998, 939, 926, 879, 856, 791, 772, 757, 747, 722, 711, 686, 678, 668, 632.

#### 3-(5-(3-Aminophenyl)-1*H*-tetrazol-1-yl)­propanenitrile **15**

A solution of **45** (490 mg, 1.56 mmol) in dry CH_2_Cl_2_ (16 mL), under a nitrogen atmosphere, was cooled to 0 °C and TFA (8 mL) was added. After 1 h, the volatile components were removed under reduced pressure, and the residue was extracted with freshly prepared 5% NaHCO_3_ solution and EtOAc. The combined organic layers were washed with brine, dried over Na_2_SO_4_, and concentrated to dryness, and crude product **15** was used in the next step without further purification. *R*_f_ 0.11 (PE/EtOAc 1:1, **A**, **C**).

#### 2-(1,1-Dioxidobenzo­[*b*]­thiophen-3-yl)­acetic Acid **18** (Scheme 8)

Acid **47** (500 mg, 2.60 mmol) was dissolved in AcOH (9 mL) and 30% H_2_O_2_ (1.2 mL, 15.6 mmol) was added. After stirring at 65 °C for 16 h, the mixture was cooled at rt and the product precipitated as white solid. The mixture was kept at 17 °C for 1 month to maximize the precipitation (this step, although beneficial, is not mandatory), and then filtered affording **18** (486 mg) as a white fluffy solid. Yield: 83%. *R*_f_ 0.27 (CH_2_Cl_2_/MeOH 98:2, **A**, **B**). Mp 261.5–262.8 °C. ^1^H NMR (300 MHz, DMSO-*d*_6_): δ 12.94 (brs, 1H, CO_2_H), 8.24 (dd, *J* = 6.9, 1.2 Hz, 1H, CH Ar), 7.96–7.91 (m, 1H, CH Ar), 7.85–7.72 (m, 2H, 2 × CH Ar), 6.88 (t, *J* = 2.3 Hz, 1H, CH–SO_2_), 4.72 (d, *J* = 2.3 Hz, 2H, CH_2_). ^13^C NMR (75 MHz, DMSO-*d*_6_): δ 166.6 (CO_2_H), 140.6 (C_q._ Ar), 140.0 (C_q._ Ar), 134.2 (CH Ar), 134.1 (C_q._ Ar), 132.9 (CH Ar), 123.9 (CH Ar), 121.4 (CH Ar), 116.4 (CH–SO_2_), 54.7 (CH_2_). IR (neat): *ν*_max_ (cm^–1^) 3074, 2989, 2944, 2833, 2758, 2621, 1832, 1684, 1624, 1587, 1573, 1465, 1451, 1428, 1386, 1344, 1303, 1291, 1267, 1256, 1229, 1210, 1181, 1163, 1147, 1121, 1067, 1001, 966, 952, 885, 840, 783, 769, 749, 711, 692, 619.

### Biological Evaluation

#### Assessment of Cell Proliferation Inhibition Using the MTT Assay

Human cell lines A2780 (ovarian adenocarcinoma), A549 (lung carcinoma), MDA-MB-231, BT474, and MDA-MB-453 (breast carcinoma), SHSY5Y and IMR-32 (neuroblastoma), and finally SK-Mel-28 (melanoma) were seeded at suitable densities in 180 μL of either RPMI 1640 or DMEM complete medium in flat-bottom 96-well microtiter plates. Once the cells had adhered, after approximately 6–8 h, they were exposed in an initial set of experiments to 20 μL of each compound (10×), resulting in a final concentration of 30 μM. Plates were then handled according to procedures described elsewhere.^44,45^

In a subsequent set of assays, SKOV3 cells were treated in the presence of IL-6 recombinant human α/IL-6 with 20 μL (10×) containing five serial 1:10 dilutions of the compounds (starting from 100 μM) selected in the previous set of assays. This pretreatment was able to activate STAT3 in these cells, making them more sensitive to anti-STAT3 activity.

Each experiment was repeated 4–6 times. IC_50_ values were determined from the analysis of individual concentration–response curves.

### Molecular Modeling and Docking Simulations

The three-dimensional model of human STAT3 was generated by homology modeling using the Phyre2 web server,^46^ and the resulting coordinates were employed as the receptor structure for all subsequent docking simulations. To evaluate the binding modes and predicted affinities of the newly synthesized hSTAT3 ligands, we adopted a blind-docking approach, in which no a priori information regarding the binding site was provided. This strategy revealed that all compounds preferentially bind near the C-terminal region of the protein, within the SH2 domain.

Molecular docking studies were performed using AutoDock^47^ in combination with ADT,^48^ employing a search grid that encompassed the entire protein. All ligand structures were generated and energy-minimized in three dimensions using MarvinSketch (ChemAxon Ltd., Budapest, HU). Docking simulations consisted of 100 runs of the Lamarckian Genetic Algorithm. The resulting docking poses were ordered by their calculated binding energies and subsequently grouped into clusters using an RMSD threshold of 2.0 Å. This workflow enabled the identification of binding modes and the accurate estimation of binding energies. All molecular graphics were produced using Chimera.^49^

### LEM Analysis

LEM analysis was performed through the AtlasCBS web tool (https://optibrium.com/resources/atlascbs-script-for-stardrop).

The molecular structures were first generated in 2D using ChemSketch and converted into SMILES codes. The SMILES strings of each molecule, together with the corresponding activity data, were collected in a CSV file, then uploaded to the AtlasCBS tool.

## Supplementary Material



## Notes

SMILES codes for compounds **2a**–**w**, the synthesis of standard **SF-1–066**, and the copies of ^1^H NMR and ^13^C NMR of intermediates and target compounds, as well as the copies of HPLC traces of all tested compounds are provided (PDF)

## ⊥

Istituto Zooprofilattico Sperimentale della Puglia e della Basilicata, Via Manfredonia 20, 71121 Foggia, Italy

## Abbreviations

DPPA: diphenyl phosphoryl azide
CDI: 1,1′-carbonyldiimidazole
EDC: *N*-(3-(dimethylamino)­propyl)- *N*′-ethylcarbodiimide
HOBT: 1-hydroxybenzotriazole
DEAD: diethylazo dicarboxylate
TMSN_3_: trimethylsilyl azide
TFE: 2,2,2-trifluoroethanol
PE: petroleum ether (40–60 °C)
FA: formic acid
