# Supplementary material for: Synthesis and Biological Evaluation of a New Family of STAT3 Inhibitors by a Diversity Oriented Approach Based on the Ugi-Multicomponent Reaction
Source: ACS Omega. 2026 Sep 9;11(37):56468–91. doi: 10.1021/acsomega.6c07733 (PMC13613459; doi:10.1021/acsomega.6c07733)

SYNTHESIS AND BIOLOGICAL EVALUATION OF A NEW FAMILY OF STAT3 INHIBITORS BY A DIVERSITY ORIENTED APPROACH BASED ON THE UGI-MULTICOMPONENT REACTION

Chiara Lambruschini,[a] Manuel Anselmo,[a] Luca Banfi,[a] Nadia Bertola,[b] Grazia Carbotti,[b,1] Maria Maddalena Cavalluzzi,[c] Giovanni Lentini,[c] Valeria Marisa Rocca,[a] Camillo Rosano,[d] Maurizio Viale,[b] Renata Riva[a*]

[a] Dipartimento di Chimica e Chimica Industriale, Università di Genova, Via Dodecaneso 31, 16146 Genova, Italy.
[b] IRCCS Azienda Ospedaliera Metropolitana, U.O.C. Bioterapie, Largo R. Benzi 10, 16132 Genova, Italy
[c] Dipartimento di Farmacia – Scienze del Farmaco, Università degli Studi di Bari Aldo Moro, via Orabona 4, 70125 Bari, Italy
[d] IRCCS Azienda Ospedaliera Metropolitana, U.O.S. Proteomica e Spettrometria di Massa, Largo R. Benzi 10, 16132 Genova, Italy

SUMMARY

1. SMILES codes for compounds **2a-w** 2
2. Synthesis of **11**, **17**, and **SF-1-066** 3
2. Copies of ^{1}H and ^{13}C NMR spectra 6
3. HPLC copies for compounds **2a-w**, and **SF-1-066** 50

[1] Present affiliation: Istituto Zooprofilattico Sperimentale della Puglia e della Basilicata, Via Manfredonia 20, 71121 Foggia, Italy

1. SMILES codes for compounds 2a-w

The following table is a copy of Table 2 reported in the paper. Here each compound has been assigned its own SMILES.

Table S1: Results of the preliminary analysis of all synthesized anti-STAT3 compounds.

			Cell Lines[a]							
**Entry[b]**	**Compound**	**SMILES Code**	**A2780**	**A549**	**MDA-MB-231**	**MDA-MB-453**	**BT474**	**SHSY5Y**	**IMR-32**	**SKMel-28**
1	**SF-1-066**	O=C(C([H])([H])N(C1=CC=C(C(O)=O)C(O)=C1)C(CN(C)S(C2=CC=C(C)C=C2)(=O)=O)=O)NC3=CC=C(C4CCCCC4)C=C3	>50[c]	>50	>50	>50	>50	>50	>50	>50
2	**2a**	O=C(C([H])([H])N(C1=CC=C(C(O)=O)C(O)=C1)C(CN(C)S(C2=CC=C(C)C=C2)(=O)=O)=O)NC3=CC=C(C4CCCCC4)C=C3	>50	>50	>50	>50	>50	>50	>50	>50
3	**2b**	O=C(C([H])([H])N(C1=CC=C(C(O)=O)C(O)=C1)C(CC2=CS(C3=C2C=CC=C3)(=O)=O)=O)NC4=CC=C(C5CCCCC5)C=C4	>50	>50	>50	>50	>50	>50	>50	>50
4	**2c**	O=C(C([H])([H])N(C1=CC=CC([N+]([O-])=O)=C1)C(CN(C)S(C2=CC=C(C)C=C2)(=O)=O)=O)NC3=CC=C(C4CCCCC4)C=C3	>50	>50	>50	>50	>50	>50	>50	>50
5	**2d**	O=C(C([H])([H])N(C1=CC=CC([N+]([O-])=O)=C1)C(CC2=CS(C3=C2C=CC=C3)(=O)=O)=O)NC4=CC=C(C5CCCCC5)C=C4	=50[d]	<50	>50	>50	>50	>50	<50	<50
6	**2l**	CNC(C([H])([H])N(C1=CC=CC([N+]([O-])=O)=C1)C(CC2=CS(C3=C2C=CC=C3)(=O)=O)=O)=O	>50	>50	>50	>50	>50	>50	>50	>50
7	**2m**	CNC(C([H])([H])N(C1=CC=CC(C#N)=C1)C(CC2=CS(C3=C2C=CC=C3)(=O)=O)=O)=O	>50	>50	>50	>50	>50	>50	>50	>50
8	**2g**	O=C(C([H])([H])N(C1=CC=CC(C#N)=C1)C(CC2=CS(C3=C2C=CC=C3)(=O)=O)=O)NC4=CC=C(C5CCCCC5)C=C4	<50	<50	<50	<50	<50	<50	<50	=50
9	**2h**	O=C(C([H])([H])N(C1=CC=CC(N(S(C)(=O)=O)S(C)(=O)=O)=C1)C(CC2=CS(C3=C2C=CC=C3)(=O)=O)=O)NC4=CC=C(C5CCCCC5)C=C4	<50	<50	<50	<50	<50	<50	<50	<50
10	**2i**	O=C(C([H])([H])N(C1=CC=CC(NS(C)(=O)=O)=C1)C(CC2=CS(C3=C2C=CC=C3)(=O)=O)=O)NC4=CC=C(C5CCCCC5)C=C4	<50	>50	>50	>50	>50	>50	>50	>50
11	**2j**	O=C(C([H])([H])N(C1=CC=CC(C2=NN=NN2CCC#N)=C1)C(CC3=CS(C4=C3C=CC=C4)(=O)=O)=O)NC5=CC=C(C6CCCCC6)C=C5	>50	>50	>50	>50	>50	>50	>50	>50
12	**2k**	O=C(C([H])([H])N(C1=CC=CC(C2=NN=NN2)=C1)C(CC3=CS(C4=C3C=CC=C4)(=O)=O)=O)NC5=CC=C(C6CCCCC6)C=C5	>50	>50	>50	>50	>50	>50	>50	>50
13	**2e**	O=C(C([H])([H])N(C1=CC=CC(C(OC)=O)=C1)C(CC2=CS(C3=C2C=CC=C3)(=O)=O)=O)NC4=CC=C(C5CCCCC5)C=C4	>50	>50	>50	>50	>50	>50	>50	>50
14	**2f**	O=C(C([H])([H])N(C1=CC=CC(C(O)=O)=C1)C(CC2=CS(C3=C2C=CC=C3)(=O)=O)=O)NC4=CC=C(C5CCCCC5)C=C4	>50	>50	>50	>50	>50	>50	>50	>50
15	**2n**	O=C(C([H])([H])N(C1=CC=CC([N+]([O-])=O)=C1)C(CN(C)S(C2=CC=C(C)C=C2)(=O)=O)=O)NC3=C(C)C=CC=C3C	>50	>50	>50	>50	>50	>50	>50	>50
16	**2o**	O=C(C([H])([H])N(C1=CC=CC([N+]([O-])=O)=C1)C(CC2=CS(C3=C2C=CC=C3)(=O)=O)=O)NC4=C(C)C=CC=C4C	<50	<50	<50	<50	<50	<50	<50	<50
17	**2p**	O=C(C([H])([H])N(C1=CC=CC(C#N)=C1)C(CC2=CS(C3=C2C=CC=C3)(=O)=O)=O)NC4=C(C)C=CC=C4C	>50	>50	>50	>50	>50	>50	>50	>50
18	**2q**	O=C(C([H])([H])N(C1=CC=CC([N+]([O-])=O)=C1)C(CC2=CS(C3=C2C=CC=C3)(=O)=O)=O)NC4=CC=C(CC5=CC(C(NC)=O)=CC=C5)C=C4	>50	>50	>50	>50	>50	>50	>50	>50
19	**2r**	O=C(C([H])([H])N(C1=CC=CC([N+]([O-])=O)=C1)C(CC2=CS(C3=C2C=CC=C3)(=O)=O)=O)NC4=CC=C(CC5=CC(C(NC6CC6)=O)=CC=C5)C=C4	>50	>50	>50	>50	>50	>50	>50	>50
20	**2s**	O=C(C([H])([H])N(C1=CC=CC([N+]([O-])=O)=C1)C(CC2=CS(C3=C2C=CC=C3)(=O)=O)=O)NC4=CC=C(C(OCC5=CC=CC=C5)=O)C=C4	>50	>50	>50	>50	>50	>50	>50	>50
21	**2t**	O=C(C([H])([H])N(C1=CC=CC(N(S(C)(=O)=O)S(C)(=O)=O)=C1)C(CC2=CS(C3=C2C=CC=C3)(=O)=O)=O)NC4=CC=CC(OC(F)(F)F)=C4	>50	>50	>50	>50	>50	>50	>50	>50
22	**2u**	O=C(C([H])([H])N(C1=CC=CC(NS(C)(=O)=O)=C1)C(CC2=CS(C3=C2C=CC=C3)(=O)=O)=O)NC4=CC=CC(OC(F)(F)F)=C4	>50	>50	>50	>50	>50	>50	>50	>50
23	**2v**	O=C(C([H])([H])N(C1=CC=CC(N(S(C)(=O)=O)S(C)(=O)=O)=C1)C(CC2=CS(C3=C2C=CC=C3)(=O)=O)=O)NC4=CC=CC(O)=C4	>50	>50	>50	>50	>50	>50	>50	>50
24	**2w**	O=C(C([H])([H])N(C1=CC=CC(NS(C)(=O)=O)=C1)C(CC2=CS(C3=C2C=CC=C3)(=O)=O)=O)NC4=CC=CC(O)=C4	>50	>50	>50	>50	>50	>50	>50	>50

Notes: [a] Cell lines used for antiproliferative tests: A2780 (ovarian adenocarcinoma), A549 (lung, carcinoma), MDA-MB-231, BT474, and MDA-MB-453 (breast carcinoma), SHSY5Y and IMR-32 (neuroblastoma), and SK-Mel-28 (melanoma). [b] Entries 2-7 represent the first-generation, entries 8-24 the second-generation of molecules. [c] The data semi-quantitatively express the percentage of cell proliferation after treatment with 30 μM of the test substances. This limit (30 μM) was arbitrarily chosen to define a pharmacologically significant antiproliferative activity in the 72-hour MTT assay. The proliferation values highlighted in grey (here expressed as a percentage) indicate probable IC_{50} values equal to or lower than 30 μM. [d] The sign =50 means that at 30 μM concentration, we have a high probability to find IC_{50} values of about 30 μM.

2. Synthesis of 11, 17, and SF-1-066

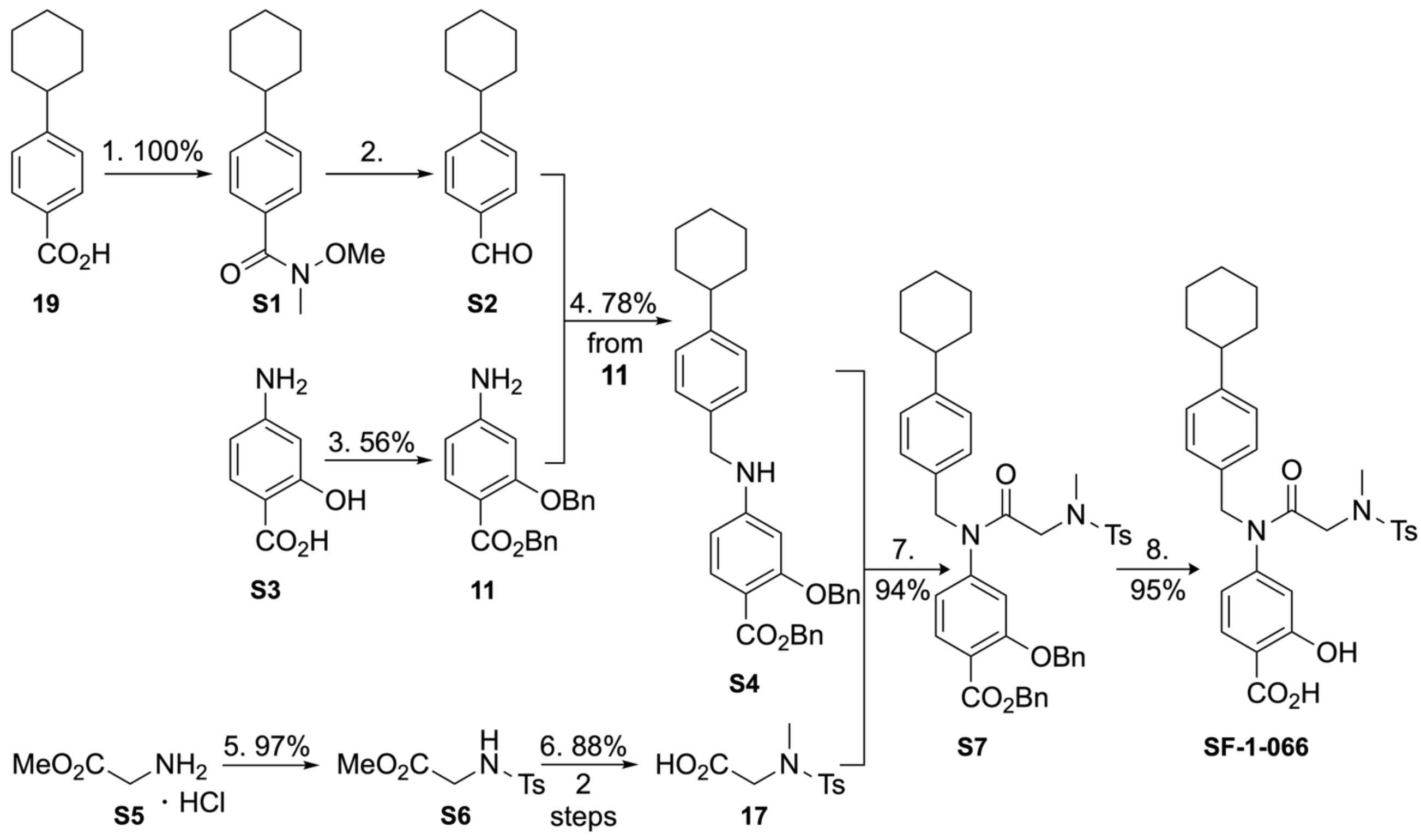

Scheme S1: Synthesis of **11**, **17** and reference **SF-1-1066**. Conditions: 1. a) $(COCl)_2$, DMF, CH_2Cl_2 0 °C; b) Et_3N, MeNHOMe·HCl, rt; 2. $LiAlH_4$, THF, –78 °C to –40 °C; 3. a) BnBr, *t*-BuOK, DMF, 0 °C to rt; b) BnBr, *t*-BuOK, DMF, 0 °C to rt; 4. a) **S1**, AcOH, 3 Å MS, MeOH, 40 °C b) $NaCNBH_3$, rt; 5. *p*-TsCl, *i*-Pr_2EtN, MeCN, 0 °C to rt; 6. a) MeI, Cs_2CO_3, DMF, rt; b) LiOH, THF/MeOH/H_2O, 3:1:1, rt; 7. PPh_3Cl_2, $CHCl_3$, 60 °C; 8. H_2, Pd/C 10%, MeOH/THF, 1:1, rt.

4-Cyclohexyl-*N*-methoxy-*N*-methylbenzamide S1. To a suspension of **19** (400 mg, 1.96 mmol) in dry CH_2Cl_2 (10 mL) oxalyl chloride (335 μL, 3.92 mmol, 2 equiv.) was added under an argon atmosphere at 0 °C. Then DMF (5 μL, 64 μmol, 0.03 equiv.) was added and the suspension became a solution, which was stirred for 3.5 h at rt. The solvent and volatile components were removed, the residue was washed with dry CH_2Cl_2 (3 × 5 mL), and then dissolved again in dry CH_2Cl_2 (10 mL). Et_3N (820 μL, 5.87 mmol, 3 equiv.) and *N,O*-dimethylhydroxylamine hydrochloride (250 mg, 2.55 mmol, 1.3 equiv.) were added stirring the mixture at rt overnight. Then it was diluted with 5% $NaHCO_3$ solution and extracted with EtOAc. The combined organic layers were washed with brine, dried over Na_2SO_4, and concentrated. Chromatography (PE/EtOAc 8:2), afforded **S1** as a colorless oil (484 mg). Yield 100%. R_f 0.31 (PE/EtOAc 8:2, **A**). ^1H NMR (300 MHz, $CDCl_3$): δ 7.61 (d, *J* = 8.3 Hz, 2H, 2×CH para syst.), 7.23 (d, *J* = 8.1 Hz, 2H, 2×CH para syst.), 3.58 (s, 3H, CH_3), 3.35 (s, 3H, CH_3), 2.60–2.46 (m, 1H, CH *c*-Hex), 1.96–1.74 (m, 5H, 5×C*H*H *c*-Hex), 1.53–1.26 (m, 5H, 5×CH*H* *c*-Hex).

4-Cyclohexylbenzaldehyde S2. To a solution of **S1** (275 mg, 1.11 mol) in dry THF (10 mL) cooled to –78 °C, $LiAlH_4$ (51 mg, 1.33 mmol, 1.2 equiv.) was added under an argon atmosphere, then the mixture was stirred for 2 h between –50 °C and –40 °C. The reaction was quenched by adding 0.1 M HCl (15 mL) and extracted with EtOAc. The combined organic layers were washed with brine, dried over Na_2SO_4, and concentrated affording **S2** as a colorless oil, which was used as such for the following step, due to its instability over silica gel. R_f 0.32 (PE/Et_2O 95:5, **A**).

Benzyl 4-amino-2-(benzyloxy)benzoate 11. *t*-BuOK (1.61 g, 14.37 mmol, 1.1 equiv.) was added to a solution of **S3** (2.00 g, 13.06 mmol) in dry DMF (90 mL) at 0 °C under an argon atmosphere. After 15 min BnBr (1.72 mL, 14.37 mmol, 1.1 equiv.) was added dropwise. After 4 h stirring at rt, the reaction was cooled to 0 °C, and further *t*-BuOK (1.61 g, 14.37 mmol, 1.1 equiv.) and BnBr (1.72 mL, 14.37, 1.1 equiv.) were added. The reaction was stirred over the weekend and then it was cautiously quenched with water and extracted with Et_2O. The combined organic layers were washed with brine, dried over Na_2SO_4, and concentrated. Chromatography (PE/EtOAc 7:3 to 6:4), followed by trituration with Et_2O, afforded **11** as a white solid (2.42 g). Yield 56%. R_f 0.32 (PE/EtOAc 6:4, **A**, **B**). ^{1}H NMR (400 MHz, $CDCl_3$): δ 7.81 (d, *J* = 8.9 Hz, 1H, CH Ar), 7.48–7.43 (m, 2H, 2×CH Ar), 7.41–7.37 (m, 2H, 2×CH Ar), 7.36–7.27 (m, 6H, 6×CH Ar), 6.26–6.22 (m, 2H, 2×CH Ar), 5.30 (s, 2H, CH_2), 5.12 (s, 2H, CH_2), 4.01 (s, 2H, NH_2).

Benzyl 2-(benzyloxy)-4-((4-cyclohexylbenzyl)amino)benzoate S4. **11** (349 mg, 1.05 mmol) was added to a solution of crude **S1** (1.05 mmol, 1 equiv.) in dry MeOH (10 mL) in the presence of powdered 3 Å MS (50 mg). Then AcOH (180 μL, 3.14 mmol, 3.0 equiv.) was added and the mixture was stirred for 1.75 h at 40 °C. Then $NaCNBH_3$ (85 mg, 1.36 mmol, 1.3 equiv.) was added together with additional MeOH (2 mL) to allow better stirring the suspension overnight. Then it was diluted with 5% $NaHCO_3$ solution and extracted with CH_2Cl_2. The combined organic layers were washed with brine, dried over Na_2SO_4, and concentrated. Chromatography (PE/CH_2Cl_2 1:1 + 0.5% to 1% Me_2CO), afforded **S4** as a white solid (412 mg). Yield 78%. R_f 0.29 (PE/CH_2Cl_2 1:1 + 0.5% Me_2CO, **A**, **B**). ^{1}H NMR (300 MHz, $CDCl_3$): δ 7.82 (d, *J* = 8.6 Hz, 1H, CH Ar), 7.47–7.35 (m, 4H, 4×CH Ar), 7.35–7.26 (m, 6H, 6×CH Ar), 7.25–7.13 (m, 4H, 4×CH Ar), 6.19 (dd, *J* = 8.6, 2.2 Hz, 1H, CH Ar), 6.15 (d, *J* = 2.1 Hz, 1H, CH Ar), 5.29 (s, 2H, CH_2O), 5.06 (s, 2H, CH_2O), 4.42 (s, 1H, NH), 4.26 (s, 2H, CH_2N), 2.55–2.41 (m, 1H, CH *c*-Hex), 1.93–1.68 (m, 5H, 5×C*H*H *c*-Hex), 1.48–1.28 (m, 5H, 5×CH*H* *c*-Hex).

Methyl 2-(4-methylphenylsulfonamido)acetate S6. *i*-Pr_2EtN (4.2 mL, 23.89 mmol, 5.0 equiv.) was added to a suspension of **S5** (600 mg, 4.78 mmol) in dry MeCN (mL) under an argon atmosphere; then *p*-TsCl (1.00 g, 5.26 mmol, 1.1 eq) was added at 0 °C, and the mixture was stirred 4 h at rt. The solvent was removed and the residue was redissolved in CH_2Cl_2. The organic phase was washed with 0.1 M HCl, 5% $NaHCO_3$ and brine, dried over Na_2SO_4, and concentrated. Chromatography (PE/EtOAc 6:4), afforded **S6** a pale yellow solid (1.13 g). Yield 97%. R_f 0.38 (PE/EtOAc 6:4, **A**, **D**). ^{1}H NMR (300 MHz, $CDCl_3$): δ 7.75 (d, *J* = 8.3 Hz, 2H, 2×CH Ar), 7.35–7.29 (m, 2H, 2×CH Ar), 5.05 (bs, 1H, NH), 3.79 (d, *J* = 4.7 Hz, 2H, CH_2), 3.64 (s, 3H, OCH_3), 2.43 (s, 3H, CH_3 of Ts).

2-(*N*,4-Dimethylphenylsulfonamido)acetic acid 17. a) Cs_2CO_3 (2.98 g, 9.14 mmol, 2.0 equiv.) was added to a solution of **S6** (1.11 g, 4.57 mmol) in dry DMF (25 mL) under an argon atmosphere at rt. After 10 min MeI (315 μL, 5.02 mmol, 1.1 equiv.) was added and the suspension was stirred for 1.5 h. It was diluted with EtOAc and washed with brine. The aqueous phase was extracted with EtOAc (× 5). The combined organic layers were washed with brine (× 6), dried over Na_2SO_4, and concentrated. Since some DMF was still present, it was removed azeotropically with toluene and high vacuum. The crude yellow oil (methyl 2-(4-methylphenylsulfonamido)acetate) was used as such for the following step. R_f 0.51 (PE/EtOAc 6:4, **A**, **B**). b) The crude product of the previous methylation, dissolved in MeOH (24 mL) and THF (8 mL), was treated at rt with a solution of LiOH (140 mg, 5.80 mmol, 1.3 equiv.) in water (8 mL) to have a MeOH/THF/H_2O 3:1:1 solution. After 4 h the volatile solvents were removed. The remaining basic solution was washed with EtOAc, and then the pH was adjusted to 1 by addition of 0.1 M HCl and extracted with EtOAc. The combined organic layers were washed with brine, dried over Na_2SO_4, and concentrated affording **17** as a white solid pure enough for further reactions (968 mg). Yield 88% from **S6**. R_f ≈0.16 (a highly elongated spot showing pronounced

streaking on the TLC plate, CH_2Cl_2/MeOH 95:5). ^{1}H NMR (300 MHz, $CDCl_3$): δ 7.70 (d, *J* = 8.3 Hz, 2H, 2×CH para syst.), 7.32 (d, *J* = 8.0 Hz, 2H, 2×CH para syst.), 3.99 (s, 2H, CH_2), 2.88 (s, 3H, NCH_3), 2.44 (s, 3H, CH_3 of Ts).

Benzyl 2-(benzyloxy)-4-(*N*-(4-cyclohexylbenzyl)-2-(*N*,4-dimethylphenylsulfonamido)acetamido)benzoate S7. An equimolar mixture of **S4** (200 mg, 0.40 mmol) and **17** (96 mg, 0.40 mmol) was dissolved in dry $CHCl_3$ under an argon atmosphere. PPh_3Cl_2 (325 mg, 0,98 mmol, 2.5 equiv.), from a freshly opened vial, was added and then the mixture was heated at 60 °C overnight. The solvent was evaporated, and the crude product was directly purified by chromatography (PE/EtOAc 1:1 + 3% Me_2CO) affording **S7** as a yellow glassy solid (272 mg). Yield 94%. R_f 0.29 (PE/CH_2Cl_2 1:1 + 3% MeOH, **A**, **B**). ^{1}H NMR (300 MHz, $CDCl_3$): δ 7.83 (d, *J* = 8.2 Hz, 1H, CH Ar), 7.61 (d, *J* = 8.3 Hz, 2H, 2×CH Ar), 7.43–7.22 (m, 12H, 12×CH Ar), 7.10 (d, *J* = 8.1 Hz, 2H, 2×CH Ar), 7.01 (d, *J* = 8.2 Hz, 2H, 2×CH Ar), 6.67 (dd, *J* = 8.2, 1.8 Hz, 1H, CH Ar), 6.50 (s, 1H, CH Ar), 5.35 (s, 2H, OCH_2), 4.94 (s, 2H, OCH_2), 4.75 (s, 2H, CH_2N), 3.65 (s, 2H, CH_2N), 2.81 (s, 3H, CH_3N), 2.57–2.42 (m, 1H, CH *c*-Hex), 2.40 (s, 3H, CH_3 of Ts), 1.91–1.67 (m, 5H, 5×C*H*H *c*-Hex), 1.45–1.25 (m, 5H, 5×CH*H* *c*-Hex).

4-(*N*-(4-cyclohexylbenzyl)-2-(*N*,4-dimethylphenylsulfonamido)acetamido)-2-hydroxybenzoic acid SF-1-066. To a solution of **S7** (259 mg, 0.35 mmol) in MeOH/THF, 1:1 Pd/C 10% (65 mg, 25% w/w) was added, and the suspension was hydrogenated at rt overnight. The reaction was filtered over a celite pad, which was washed with MeOH. After solvent evaporation, the crude mixture was purified by chromatography (CH_2Cl_2/MeOH 95:5 + 1% AcOH) affording **SF-1-066** as an off-white solid (184 mg). Yield 95%. R_f 0.38 (PE/CH_2Cl_2 95:5 + 1% AcOH, **A**, **B**). HPLC purity 100%, R_t 12.04 min (Phenyl C6 150 x 3 mm, 3 μm column, A: H_2O/MeCN 3:7+ 0.1% FA to B: MeCN + 0.1% FA in 20 min, flow 0.34 mL/min, temp. 25 °C). ^{1}H NMR (300 MHz, $CDCl_3$): δ 7.74 (d, *J* = 8.3 Hz, 1H, CH Ar), 7.55 (d, *J* = 8.3 Hz, 2H, 2×CH para syst.), 7.36 (d, *J* = 7.9 Hz, 2H, 2×CH para syst.), 7.14 (d, *J* = 8.2 Hz, 2H, 2×CH para syst.), 7.07 (d, *J* = 8.2 Hz, 2H, 2×CH para syst.), 6.78 (d, *J* = 2.0 Hz, 1H, CH Ar), 6.70 (dd, *J* = 8.3, 2.1 Hz, 1H, CH Ar), 4.78 (s, 2H, CH_2), 3.85 (s, 2H, CH_2), 2.78 (s, 3H, CH_3), 2.45 (s, 1H, CH *c*-Hex), 2.37 (s, 3H, CH_3), 1.84–1.62 (m, 5H, 5×C*H*H *c*-Hex), 1.45–1.15 (m, 5H, 5×CH*H* *c*-Hex).

2. Copies of ^{1}H and ^{13}C NMR spectra

2a

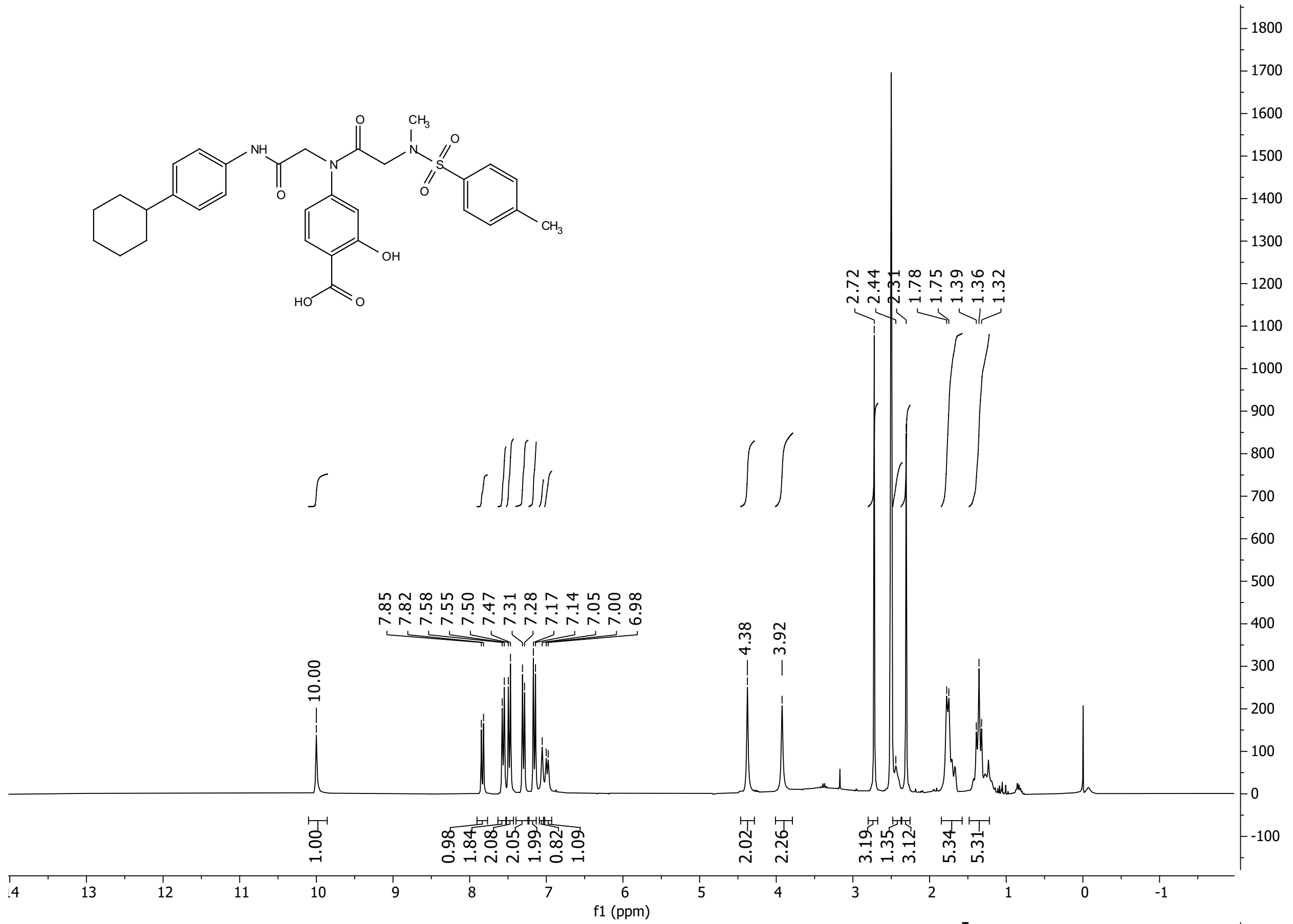

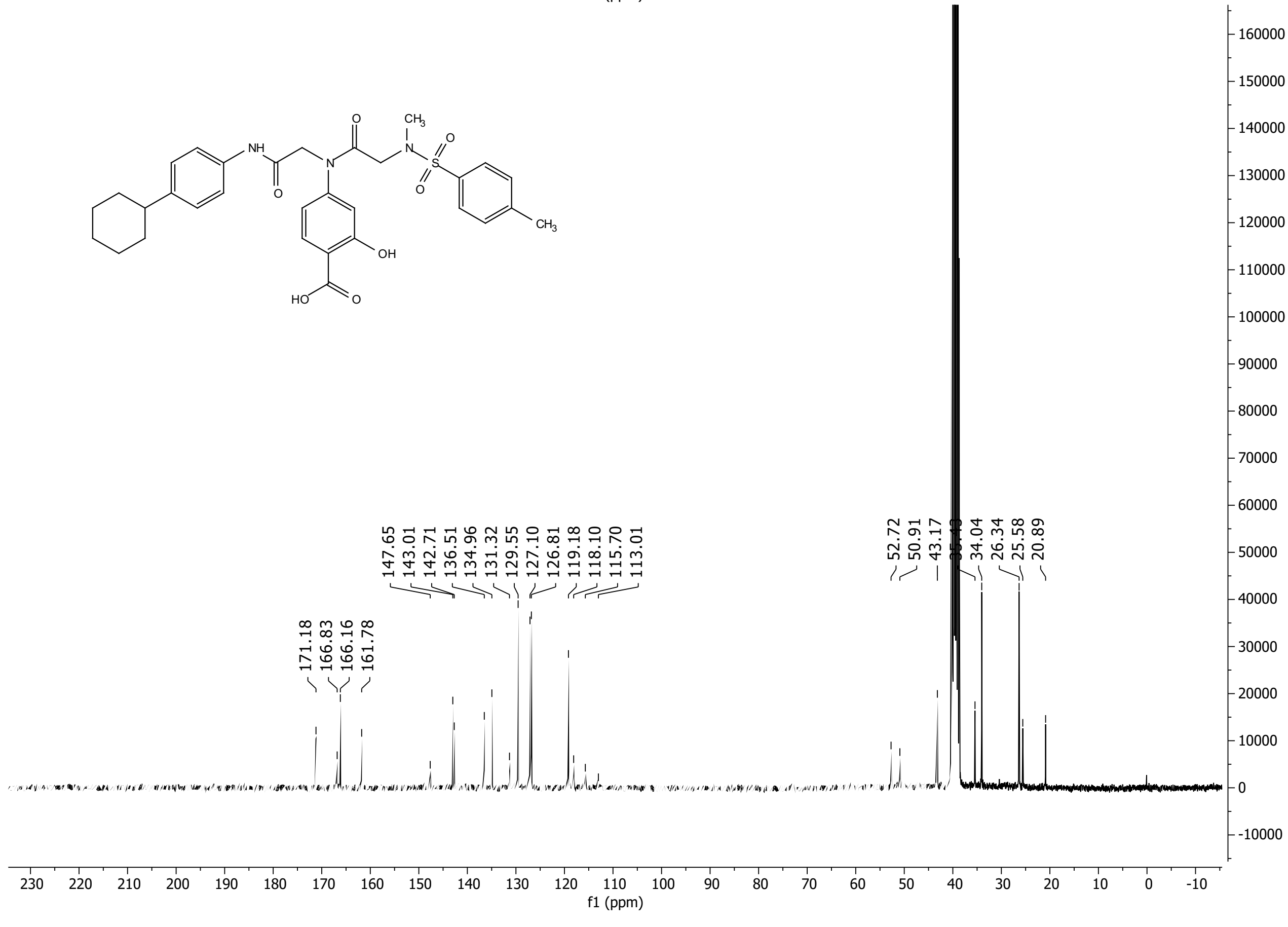

2b

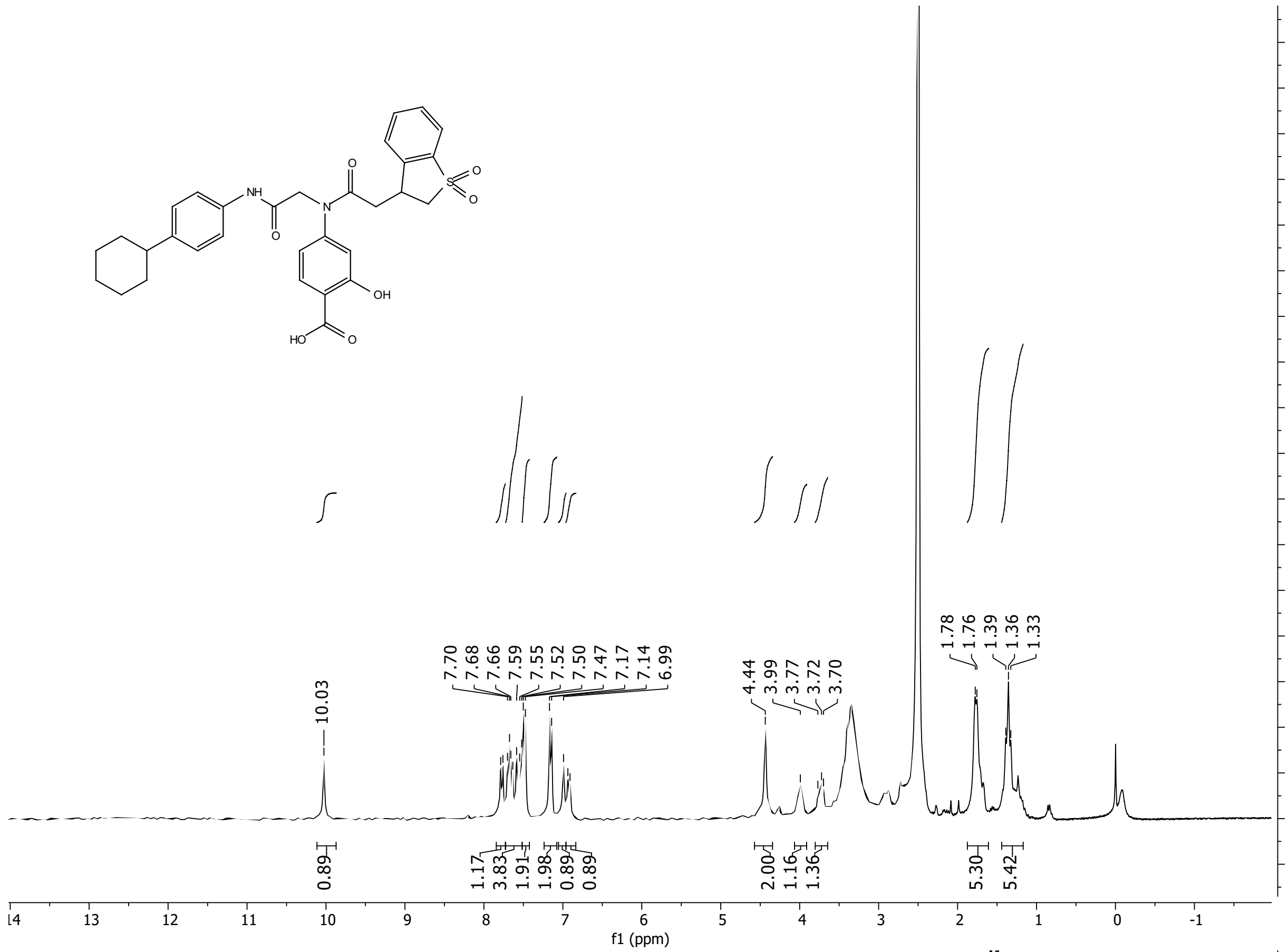

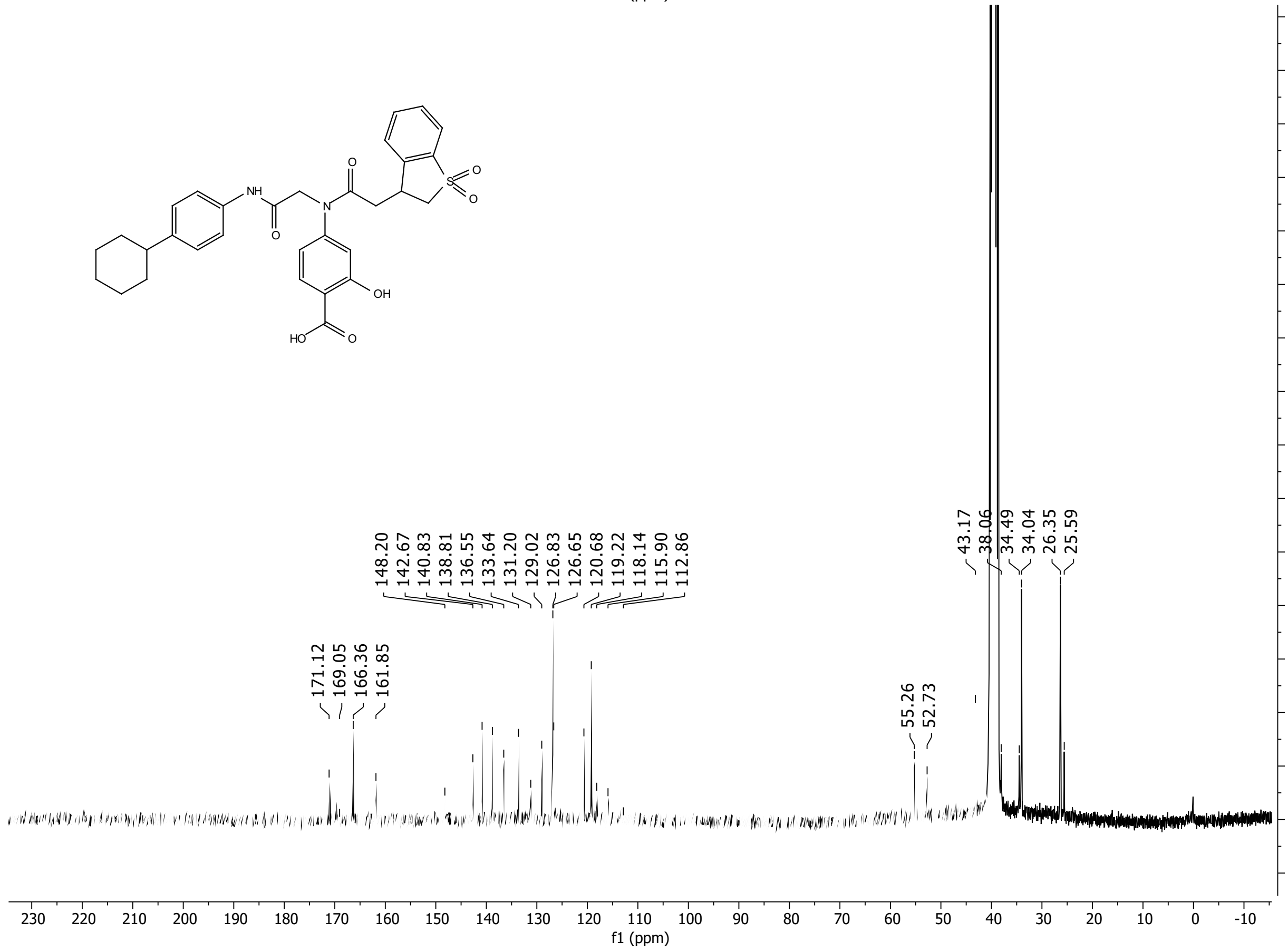

2c

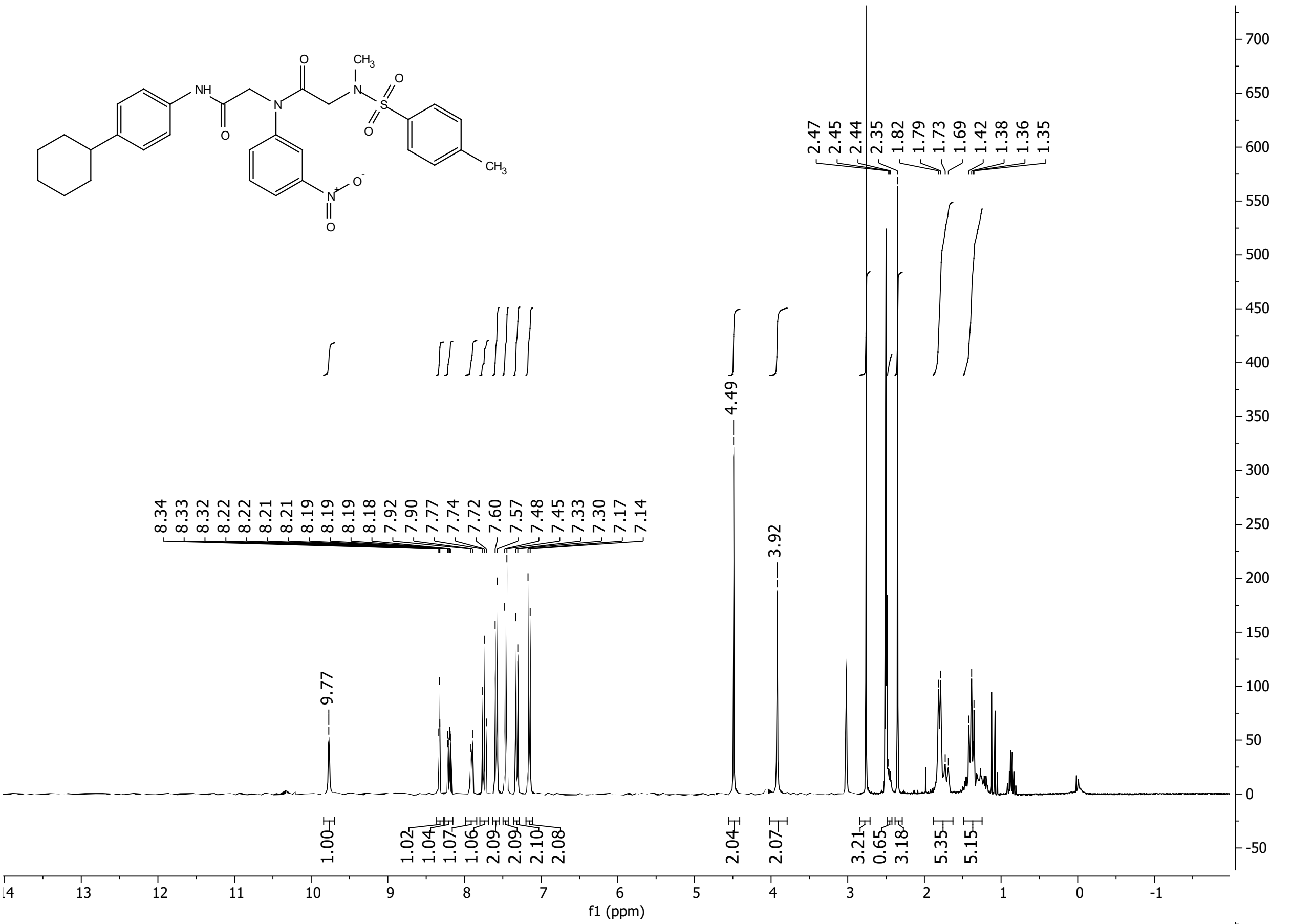
8.34
8.33
8.32
8.22
8.22
8.21
8.21
8.19
8.19
8.19
8.18
7.92
7.90
7.77
7.74
7.72
7.60
7.57
7.48
7.45
7.33
7.30
7.17
7.14
9.77
4.49
3.92
2.47
2.45
2.44
2.35
1.82
1.79
1.73
1.69
1.42
1.38
1.36
1.35
1.00
1.02
1.04
1.07
1.06
2.09
2.09
2.10
2.08
2.04
2.07
3.21
0.65
3.18
5.35
5.15
f1 (ppm)

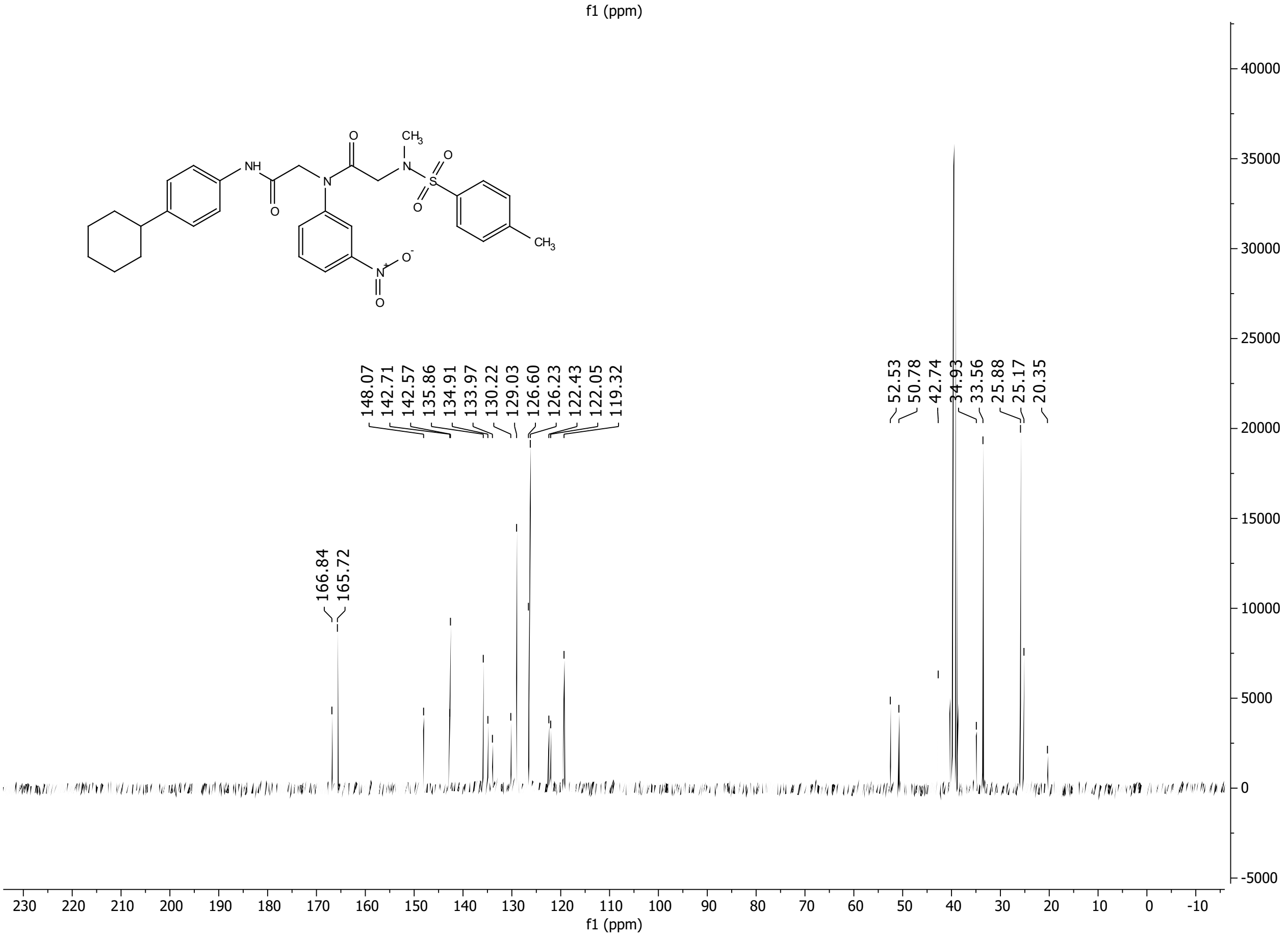
148.07
142.71
142.57
135.86
134.91
133.97
130.22
129.03
126.60
126.23
122.43
122.05
119.32
166.84
165.72
52.53
50.78
42.74
34.93
33.56
25.88
25.17
20.35
f1 (ppm)

2d

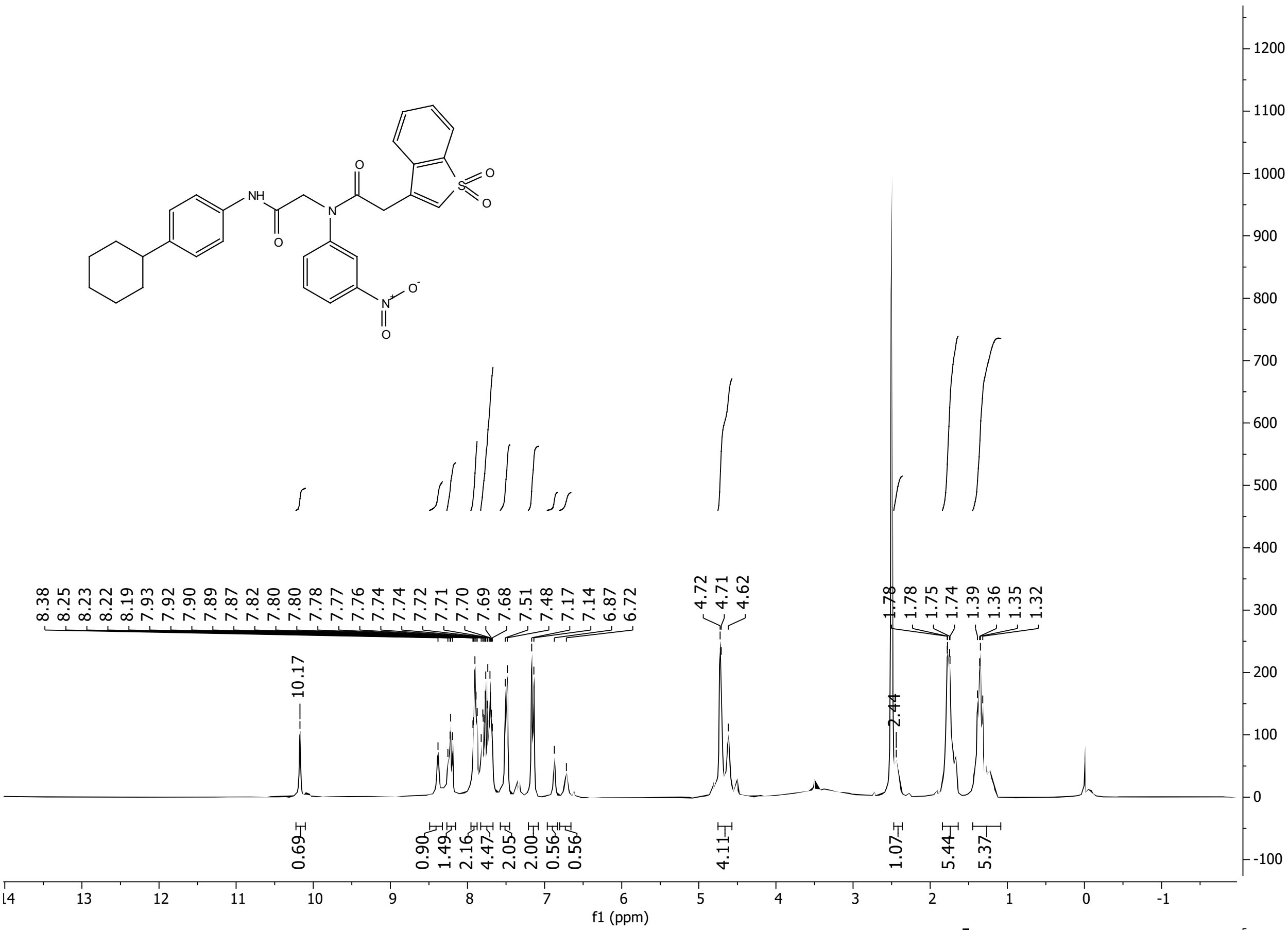

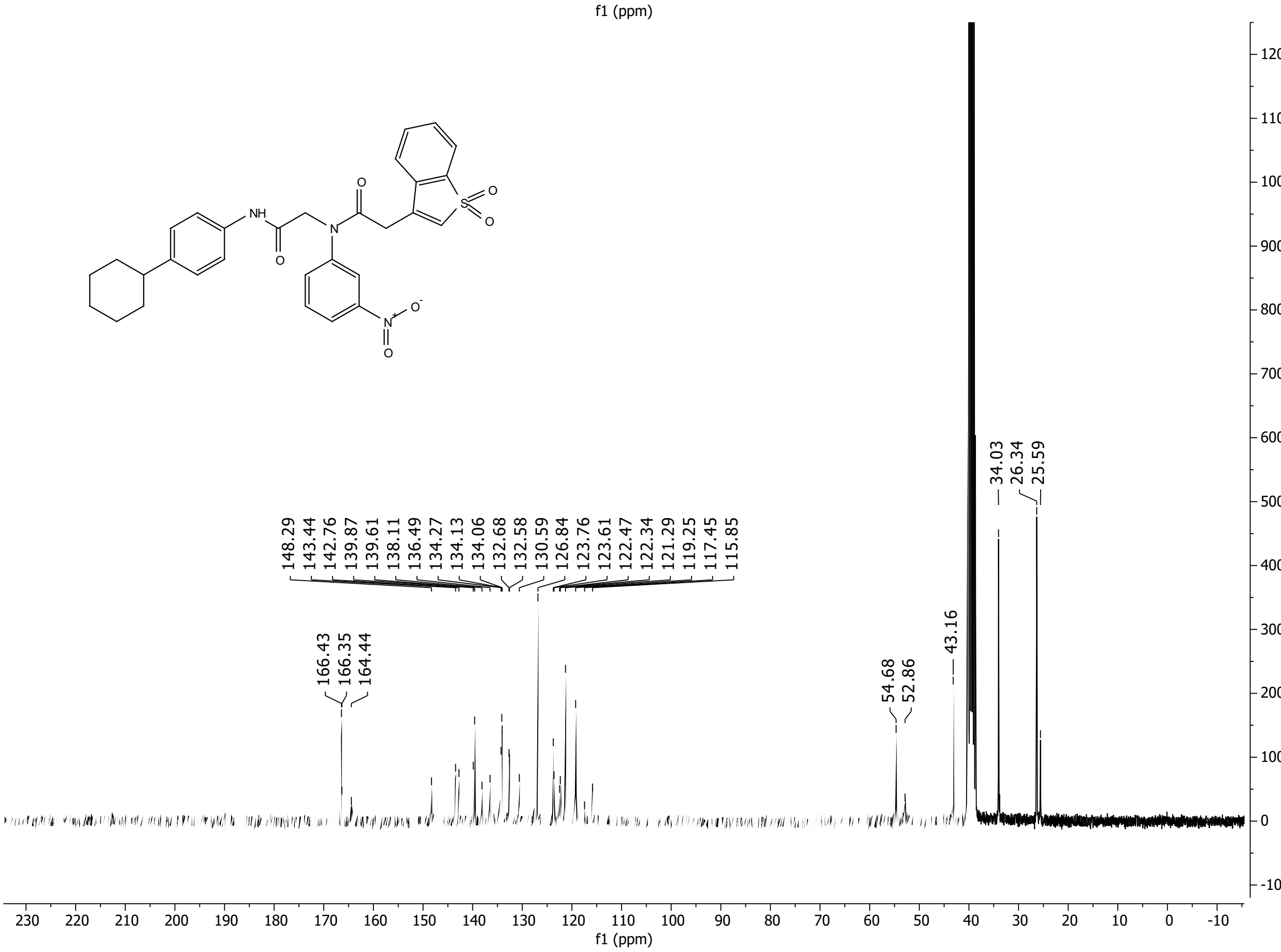

2e

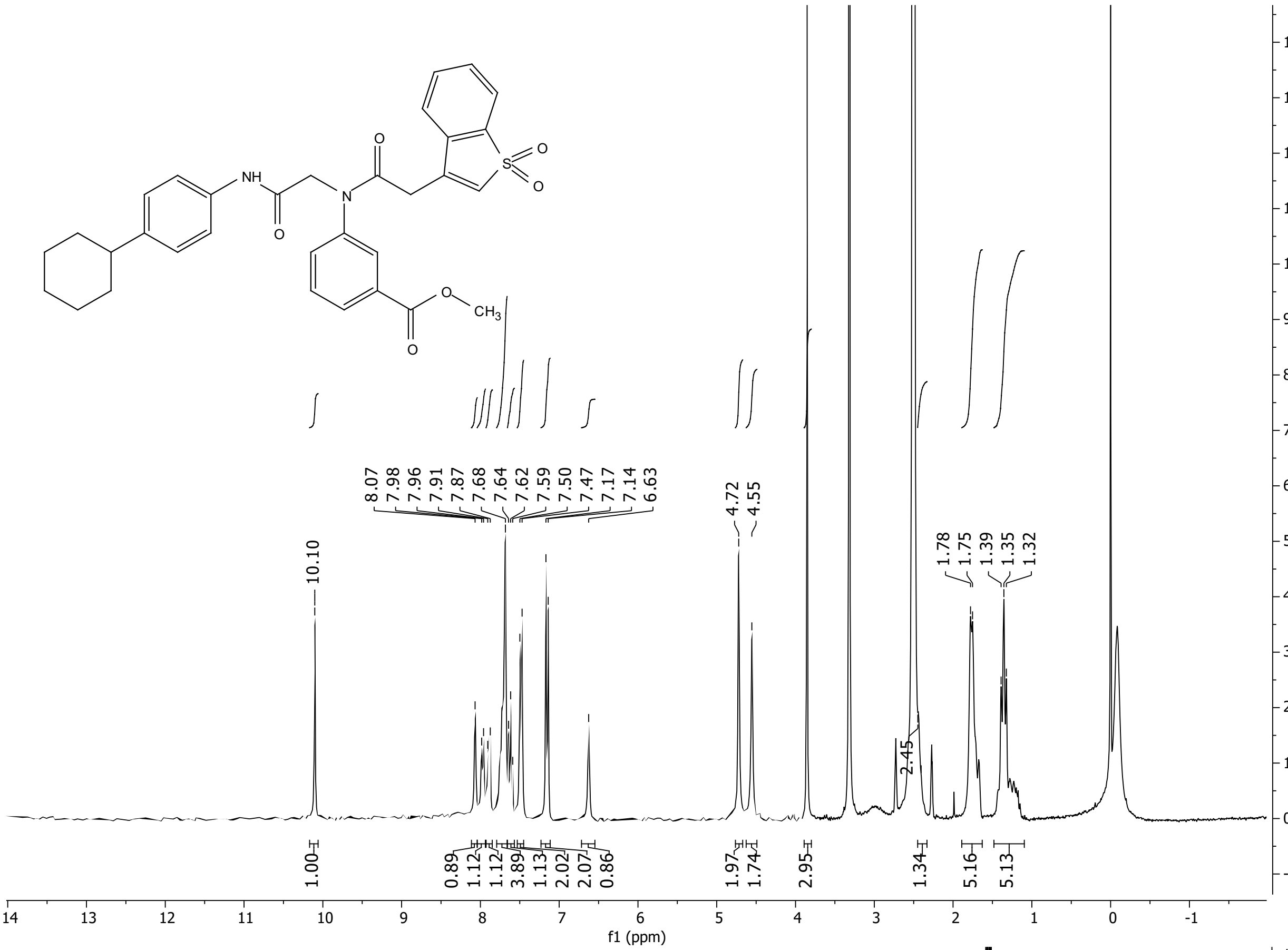
NH
O
N
O
S
O
O
O
O
CH3
10.10
8.07
7.98
7.96
7.91
7.87
7.68
7.64
7.62
7.59
7.50
7.47
7.17
7.14
6.63
4.72
4.55
2.45
1.78
1.75
1.39
1.35
1.32
1.00
0.89
1.12
1.12
3.89
1.13
2.02
2.07
0.86
1.97
1.74
2.95
1.34
5.16
5.13
f1 (ppm)

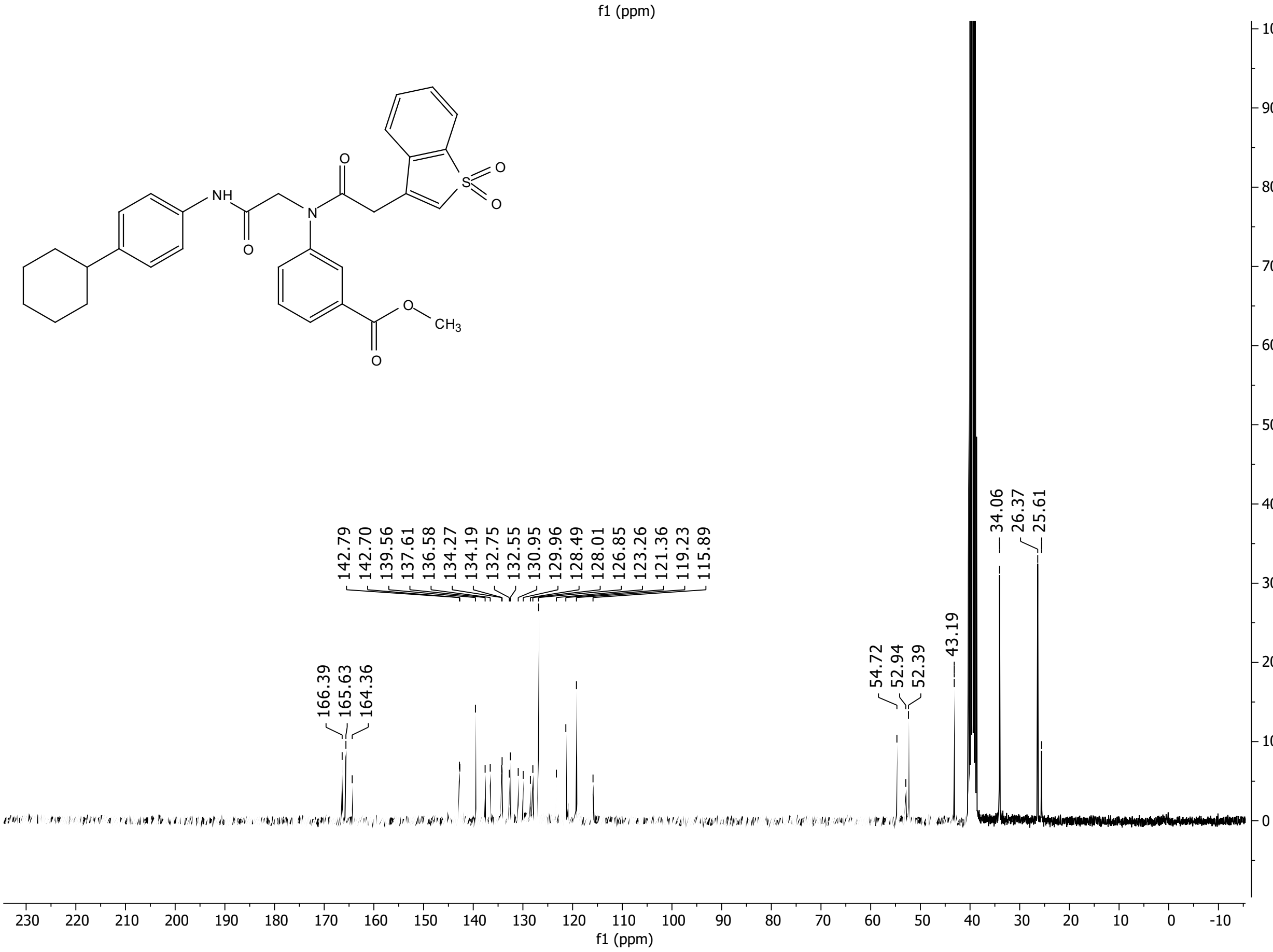
NH
O
N
O
S
O
O
O
O
CH3
166.39
165.63
164.36
142.79
142.70
139.56
137.61
136.58
134.27
134.19
132.75
132.55
130.95
129.96
128.49
128.01
126.85
123.26
121.36
119.23
115.89
54.72
52.94
52.39
43.19
34.06
26.37
25.61
f1 (ppm)

2f

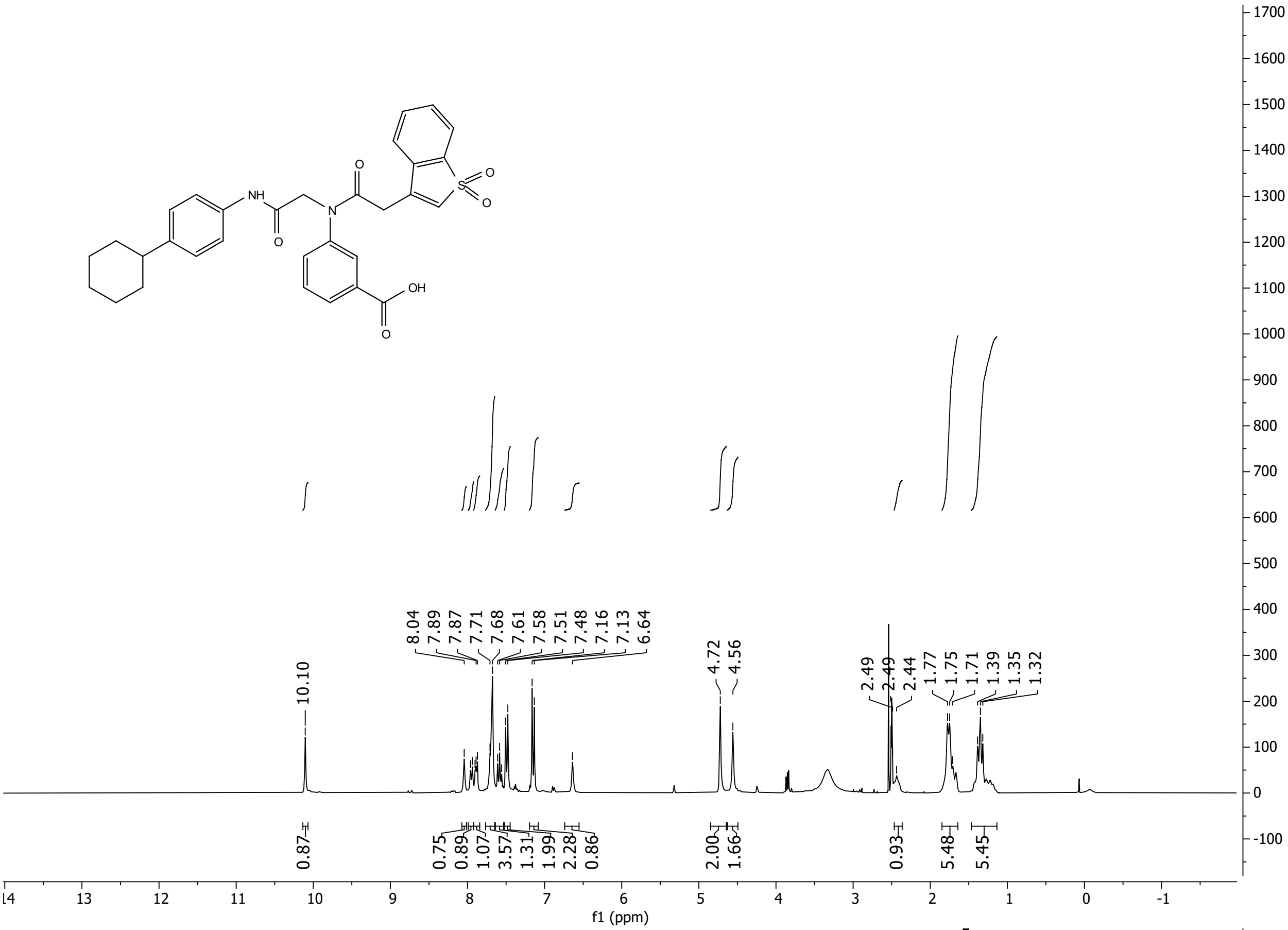
NH
O
N
O
S
O
O
OH
O
10.10
8.04 7.89 7.87 7.71 7.68 7.61 7.58 7.51 7.48 7.16 7.13 6.64
4.72 4.56
2.49 2.49 2.44 1.77 1.75 1.71 1.39 1.35 1.32
0.87
0.75 0.89 1.07 3.57 1.31 1.99 2.28 0.86
2.00 1.66
0.93
5.48
5.45
f1 (ppm)

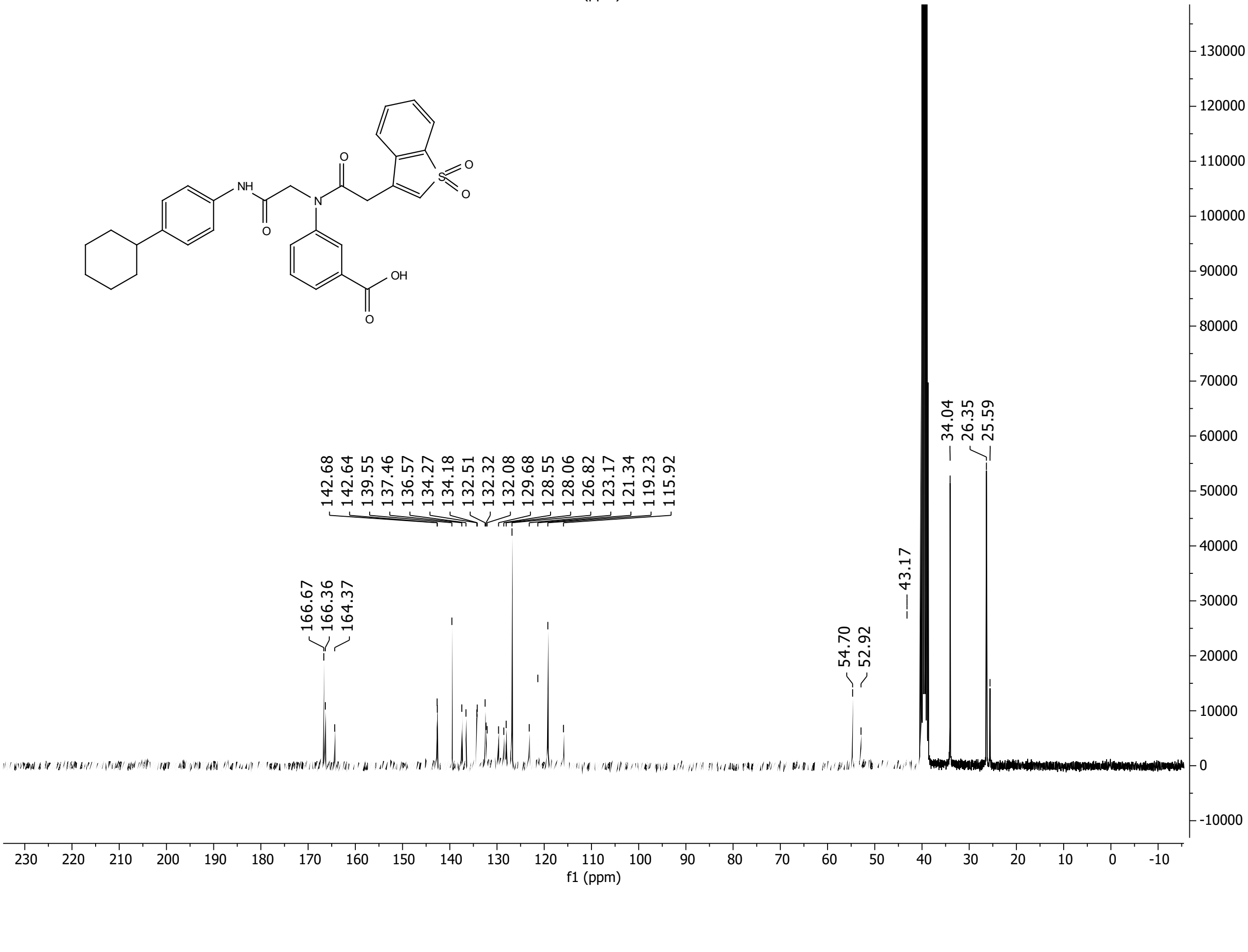
NH
O
N
O
S
O
O
OH
O
166.67 166.36 164.37
142.68 142.64 139.55 137.46 136.57 134.27 134.18 132.51 132.32 132.08 129.68 128.55 128.06 126.82 123.17 121.34 119.23 115.92
54.70 52.92
43.17
34.04 26.35 25.59
f1 (ppm)

2g

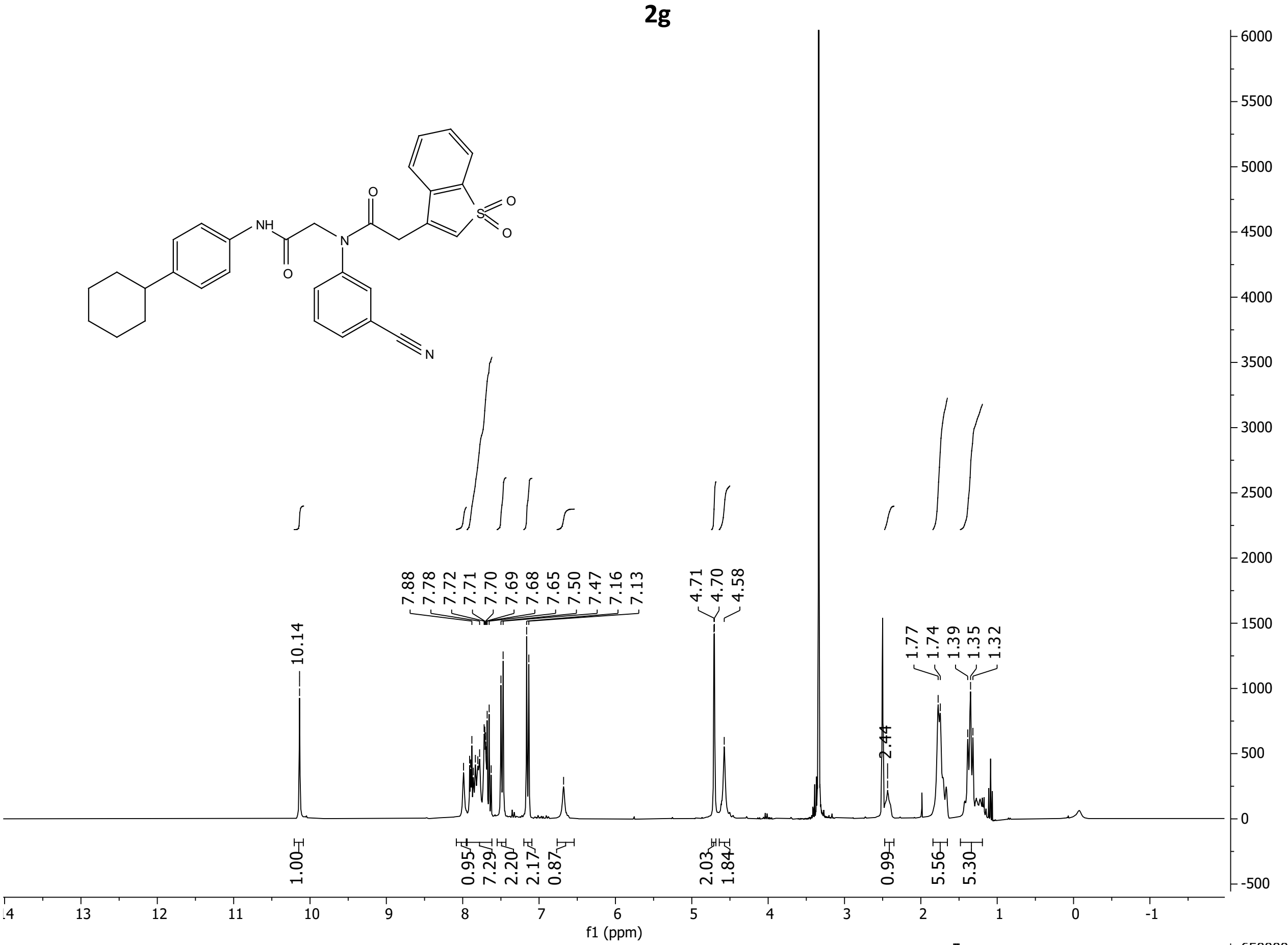
NH
O
N
O
S
O
O
N
10.14
7.88
7.78
7.72
7.71
7.70
7.69
7.68
7.65
7.50
7.47
7.16
7.13
4.71
4.70
4.58
2.44
1.77
1.74
1.39
1.35
1.32
1.00
0.95
7.29
2.20
2.17
0.87
2.03
1.84
0.99
5.56
5.30
f1 (ppm)

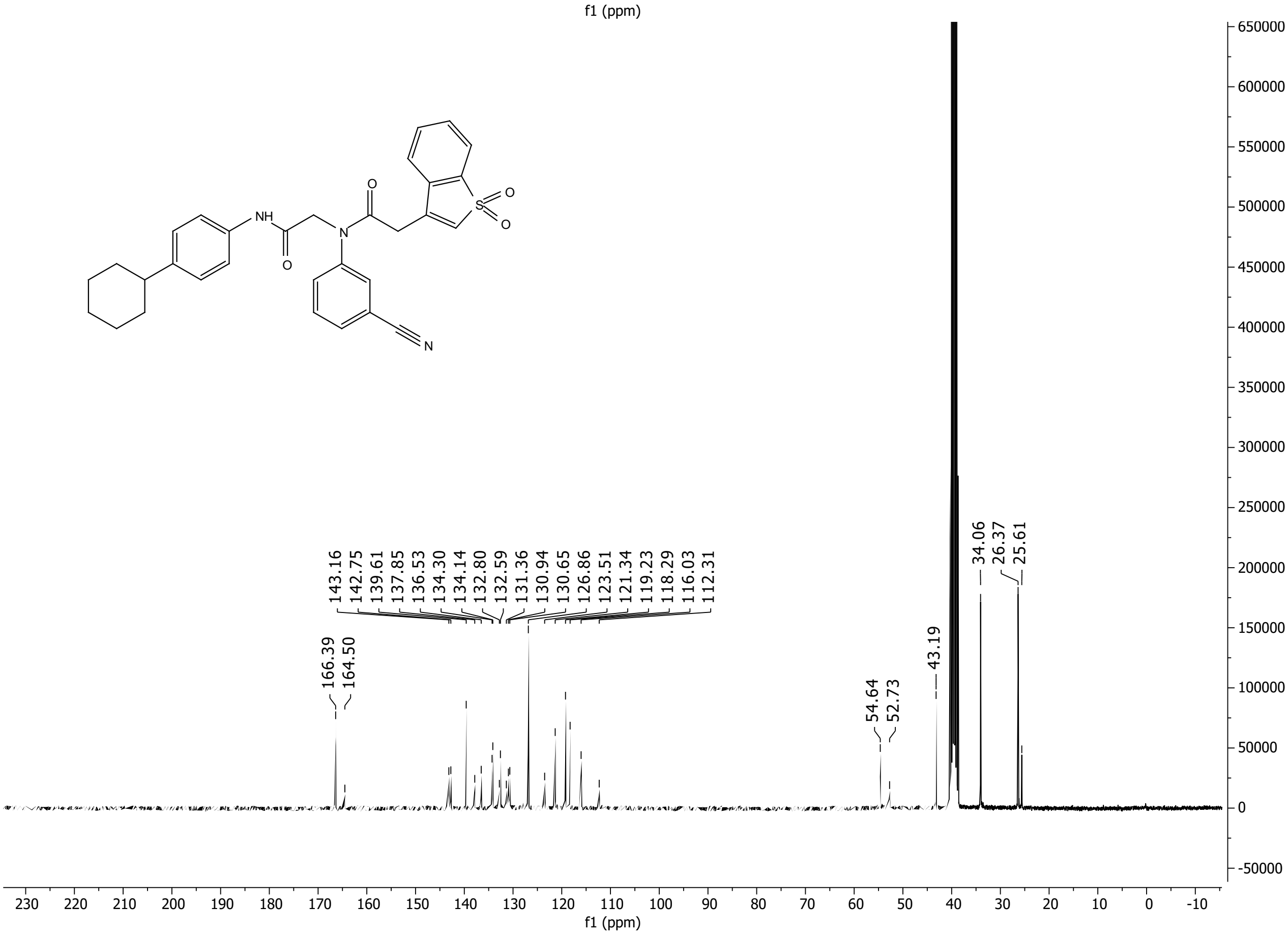
NH
O
N
O
S
O
O
N
166.39
164.50
143.16
142.75
139.61
137.85
136.53
134.30
134.14
132.80
132.59
131.36
130.94
130.65
126.86
123.51
121.34
119.23
118.29
116.03
112.31
54.64
52.73
43.19
34.06
26.37
25.61
f1 (ppm)

2h

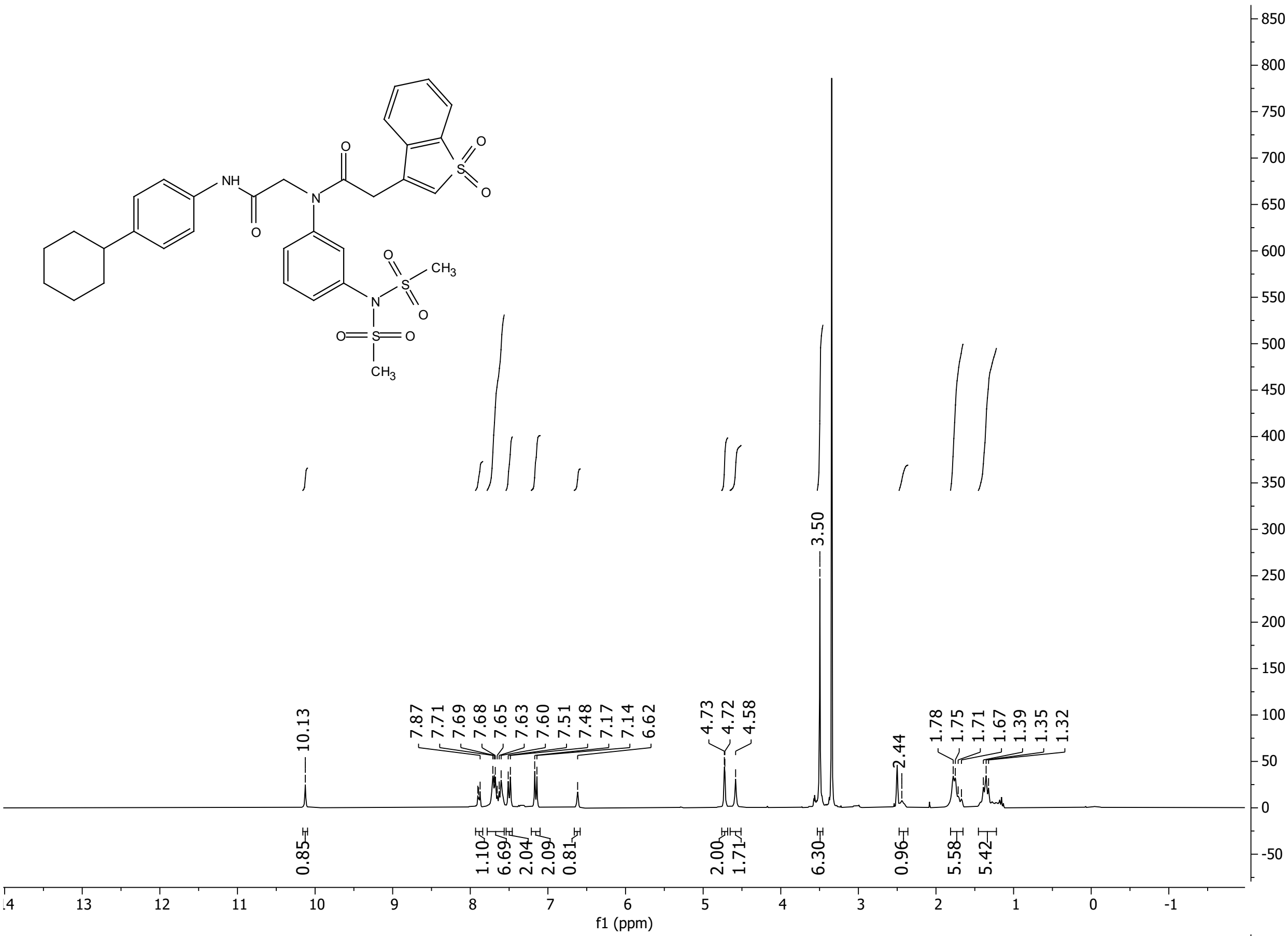
NH
O
N
O
S
O
O
CH3
N
O=S=O
CH3
10.13
7.87
7.71
7.69
7.68
7.65
7.63
7.60
7.51
7.48
7.17
7.14
6.62
4.73
4.72
4.58
3.50
2.44
1.78
1.75
1.71
1.67
1.39
1.35
1.32
0.85
1.10
6.69
2.04
2.09
0.81
2.00
1.71
6.30
0.96
5.58
5.42
f1 (ppm)

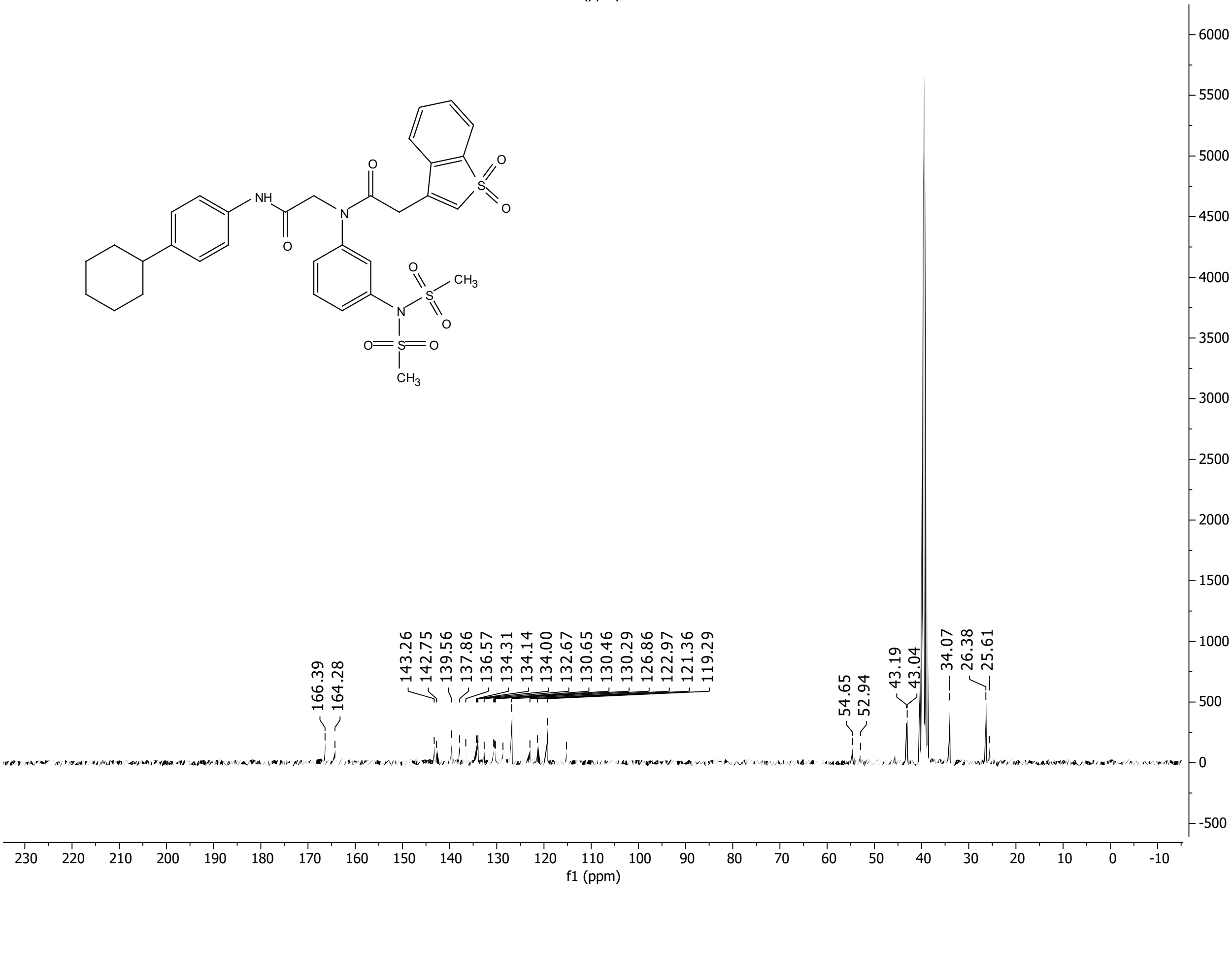
NH
O
N
O
S
O
O
CH3
N
O=S=O
CH3
166.39
164.28
143.26
142.75
139.56
137.86
136.57
134.31
134.14
134.00
132.67
130.65
130.46
130.29
126.86
122.97
121.36
119.29
54.65
52.94
43.19
43.04
34.07
26.38
25.61
f1 (ppm)

2i

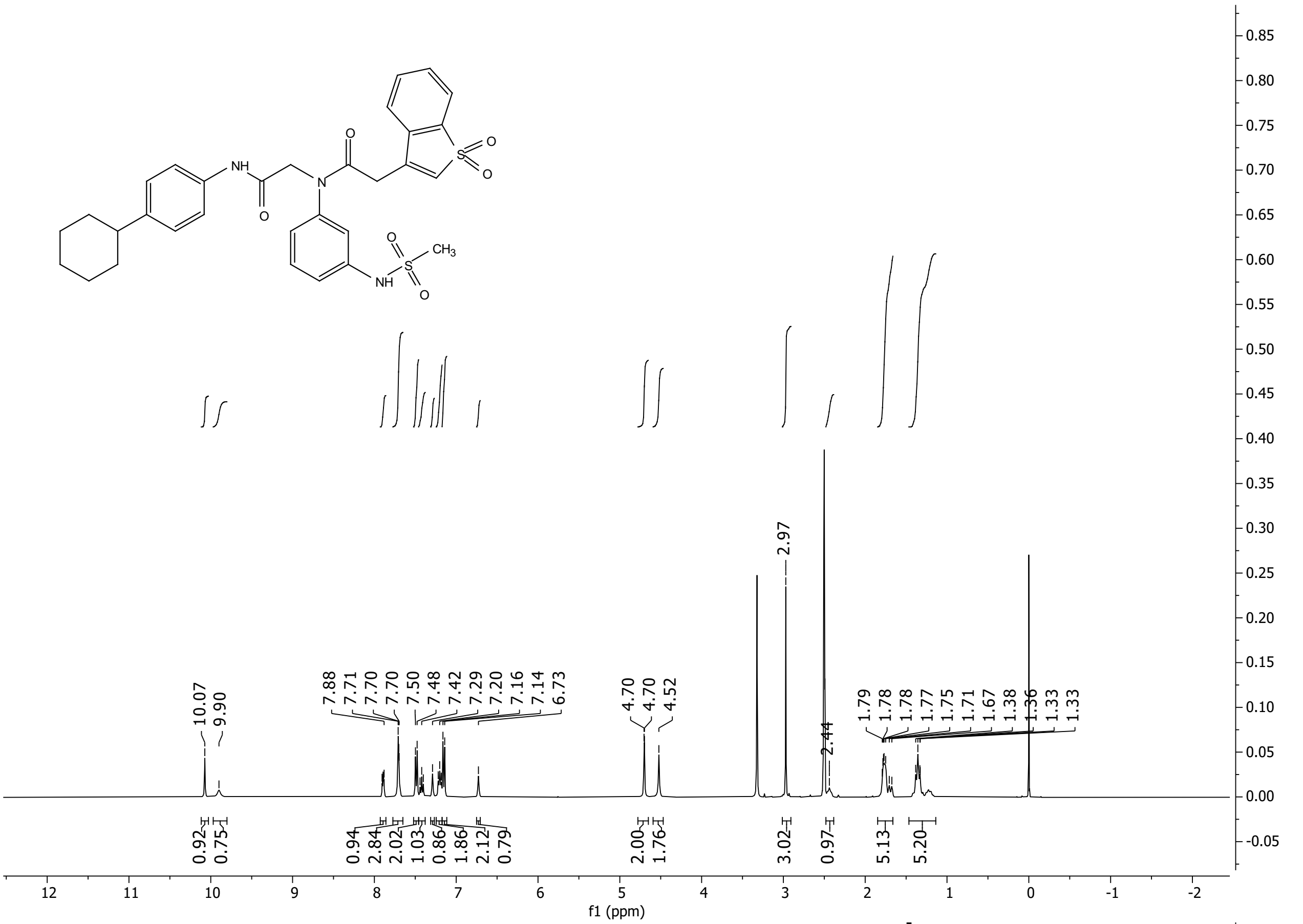
NH
O
N
O
S
O
O
NH
S
O
O
CH3
10.07
9.90
7.88
7.71
7.70
7.70
7.50
7.48
7.42
7.29
7.20
7.16
7.14
6.73
4.70
4.70
4.52
2.97
2.44
1.79
1.78
1.78
1.77
1.75
1.71
1.67
1.38
1.36
1.33
1.33
0.92
0.75
0.94
2.84
2.02
1.03
0.86
1.86
2.12
0.79
2.00
1.76
3.02
0.97
5.13
5.20
f1 (ppm)

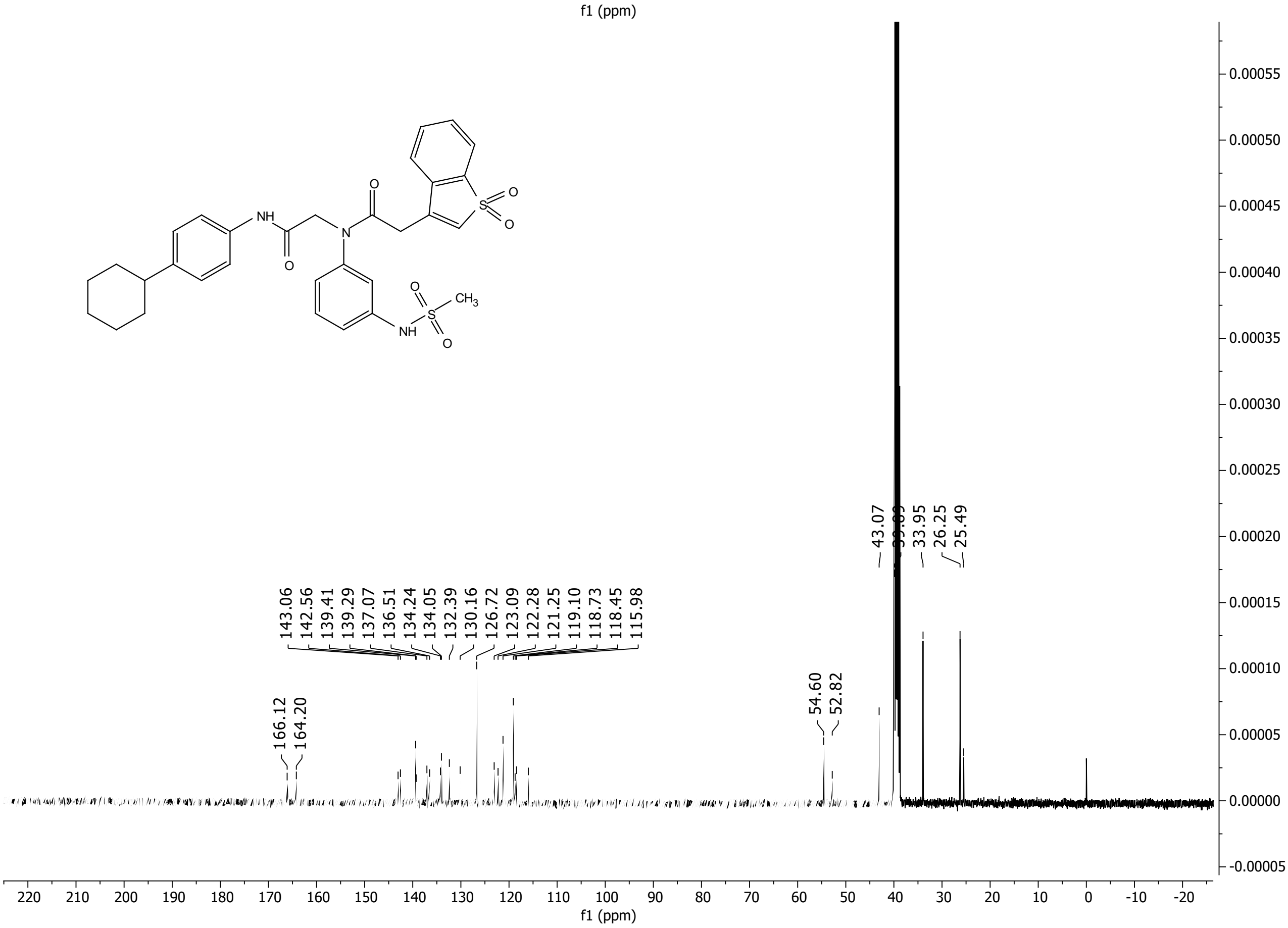
NH
O
N
O
S
O
O
NH
S
O
O
CH3
166.12
164.20
143.06
142.56
139.41
139.29
137.07
136.51
134.24
134.05
132.39
130.16
126.72
123.09
122.28
121.25
119.10
118.73
118.45
115.98
54.60
52.82
43.07
33.95
26.25
25.49
f1 (ppm)

2j

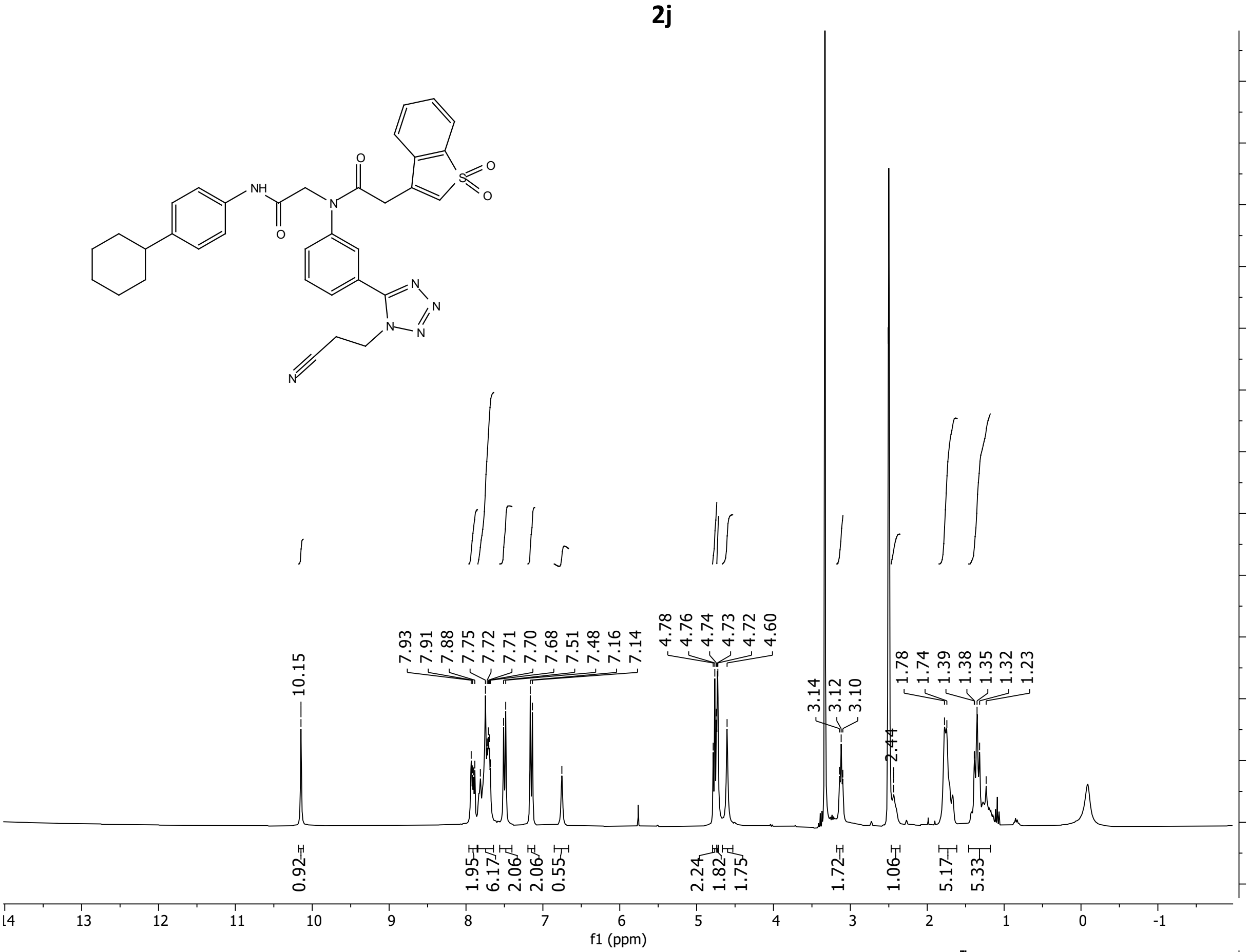

10.15
7.93 7.91 7.88 7.75 7.72 7.71 7.70 7.68 7.51 7.48 7.16 7.14
4.78 4.76 4.74 4.73 4.72 4.60
3.14 3.12 3.10
2.44
1.78 1.74 1.39 1.38 1.35 1.32 1.23
0.92 1.95 6.17 2.06 2.06 0.55 2.24 1.82 1.75 1.72 1.06 5.17 5.33
f1 (ppm)

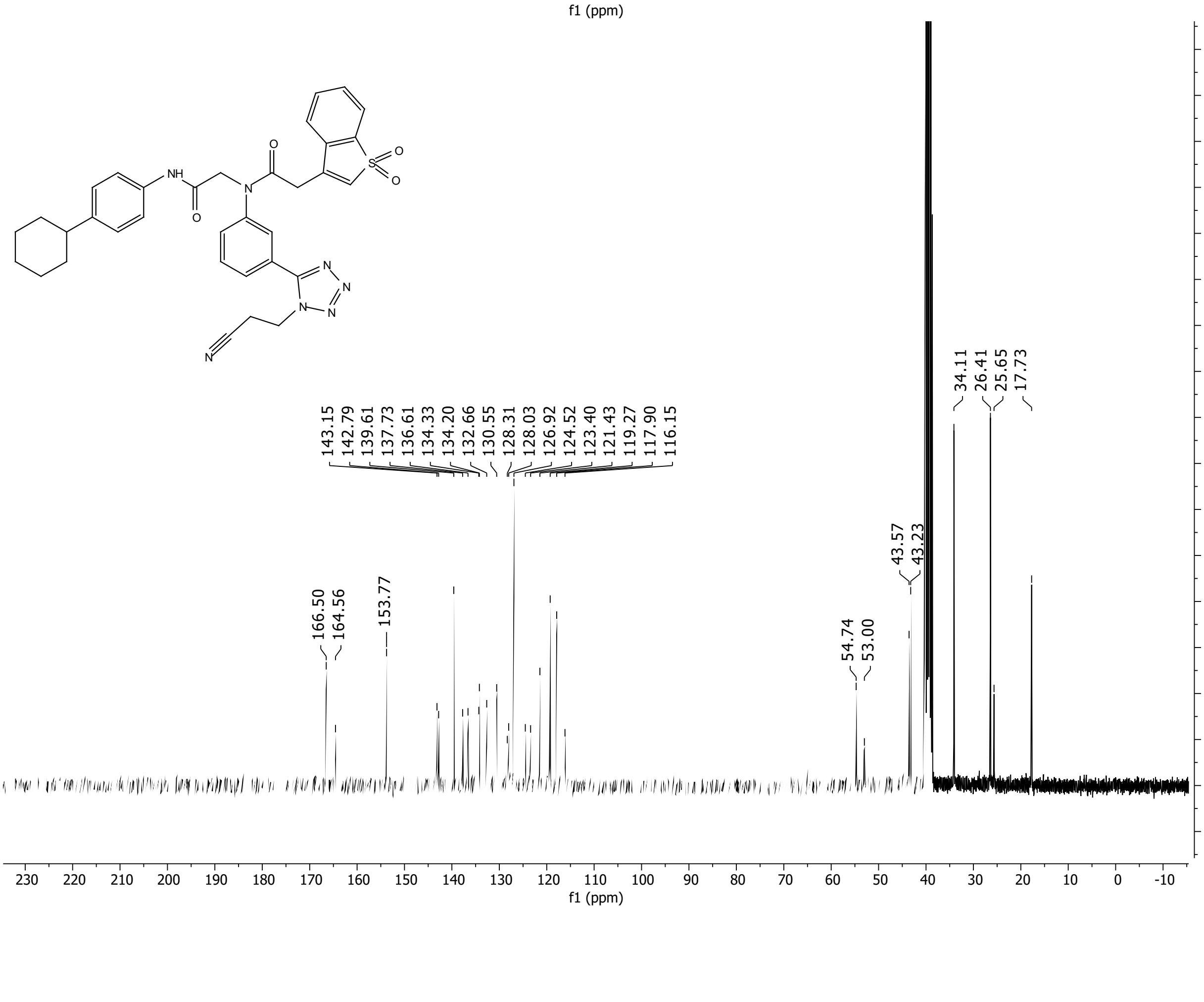

166.50 164.56 153.77
143.15 142.79 139.61 137.73 136.61 134.33 134.20 132.66 130.55 128.31 128.03 126.92 124.52 123.40 121.43 119.27 117.90 116.15
54.74 53.00 43.57 43.23
34.11 26.41 25.65 17.73
f1 (ppm)

2k

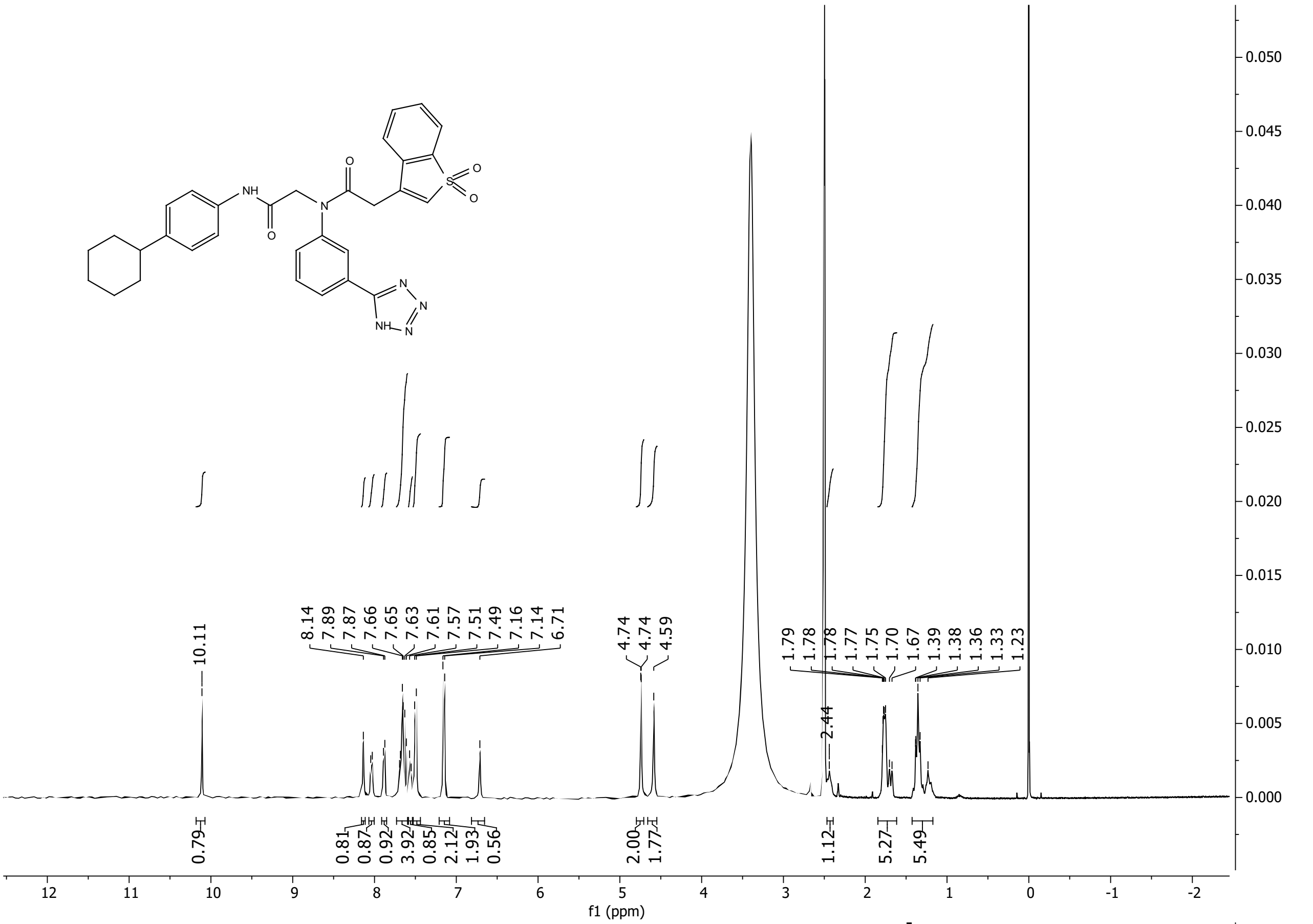

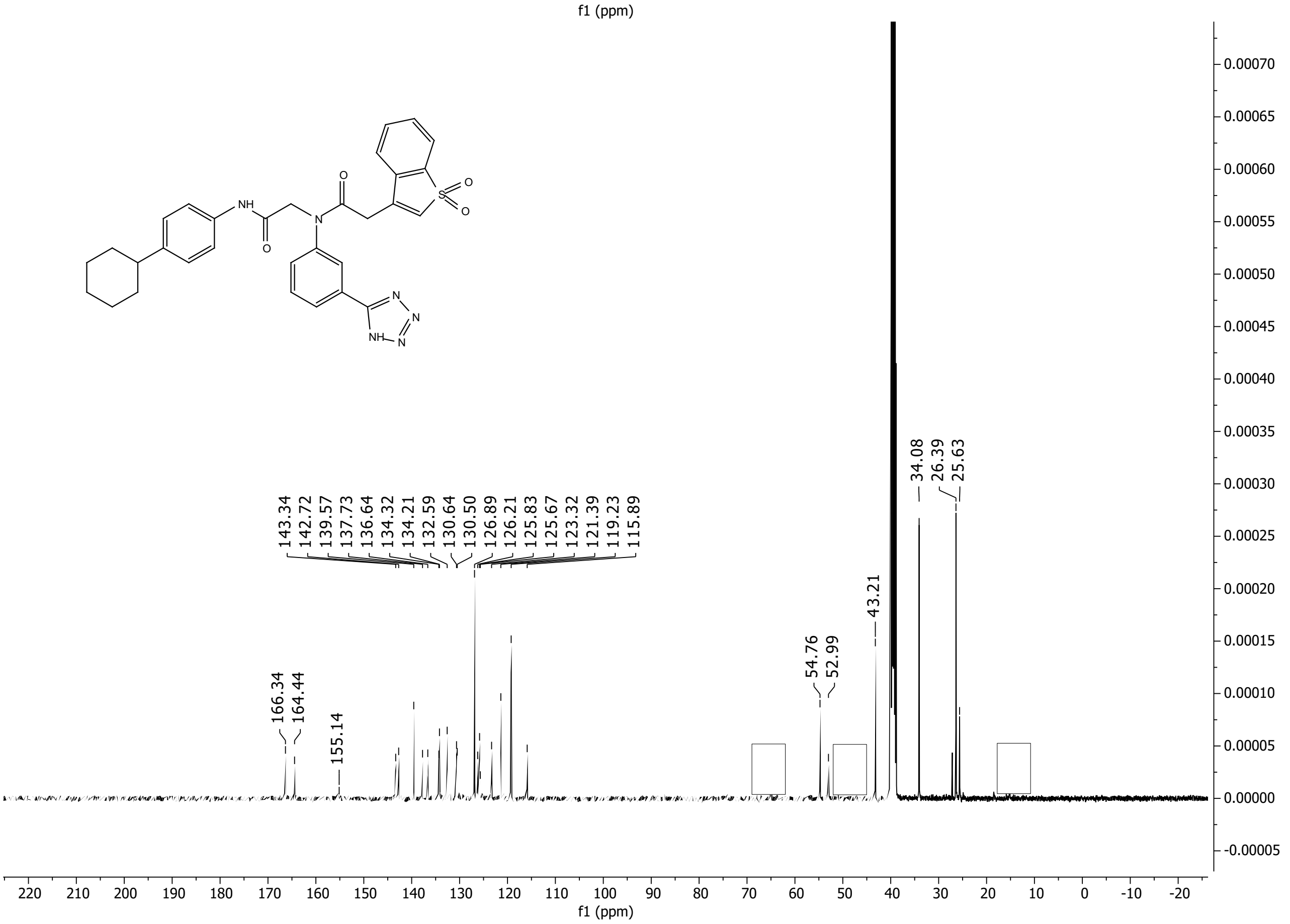

2l

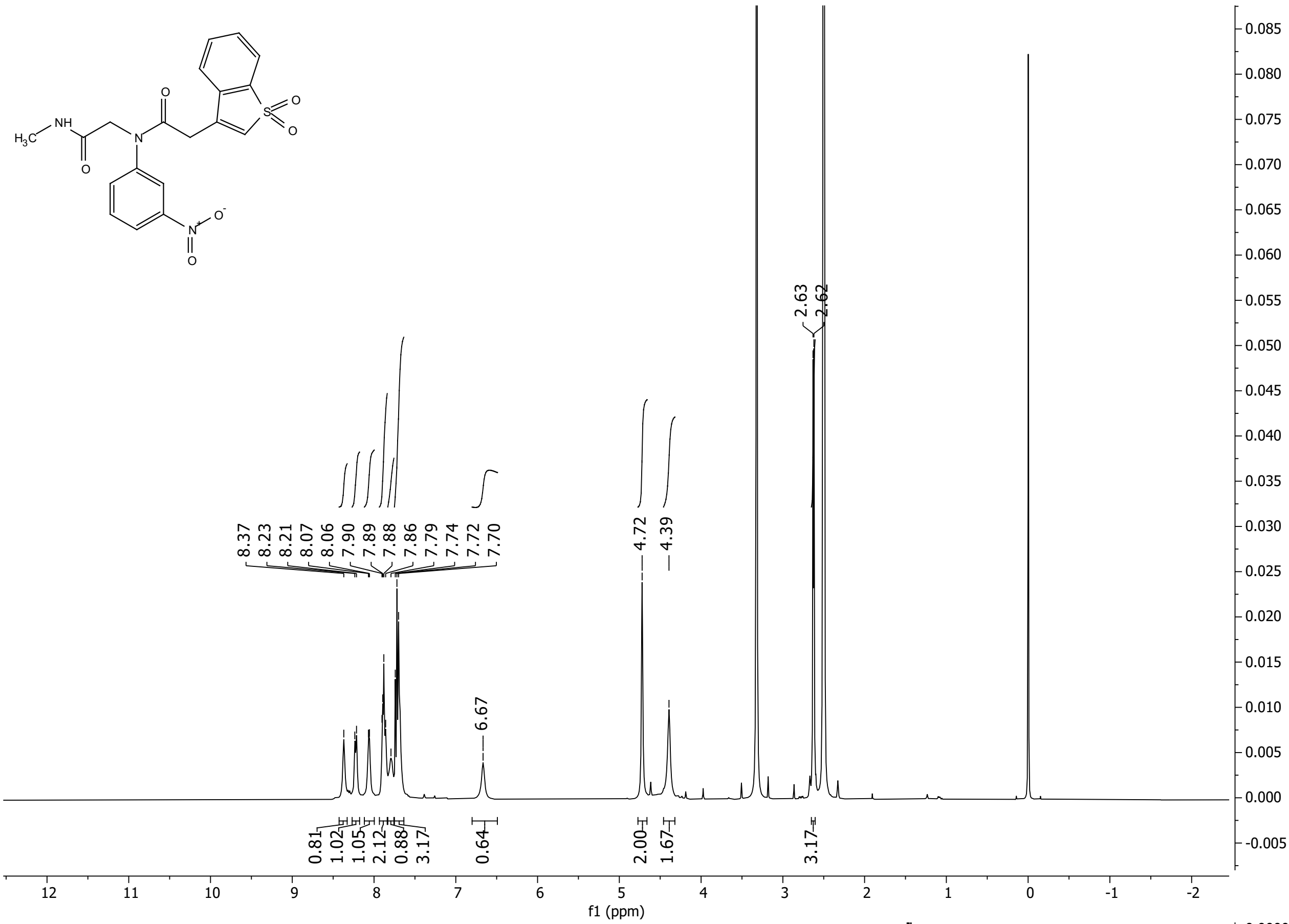
8.37 8.23 8.21 8.07 8.06 7.90 7.89 7.88 7.86 7.79 7.74 7.72 7.70
6.67
4.72
4.39
2.63 2.62
0.81 1.02 1.05 2.12 0.88 3.17
0.64
2.00
1.67
3.17
f1 (ppm)

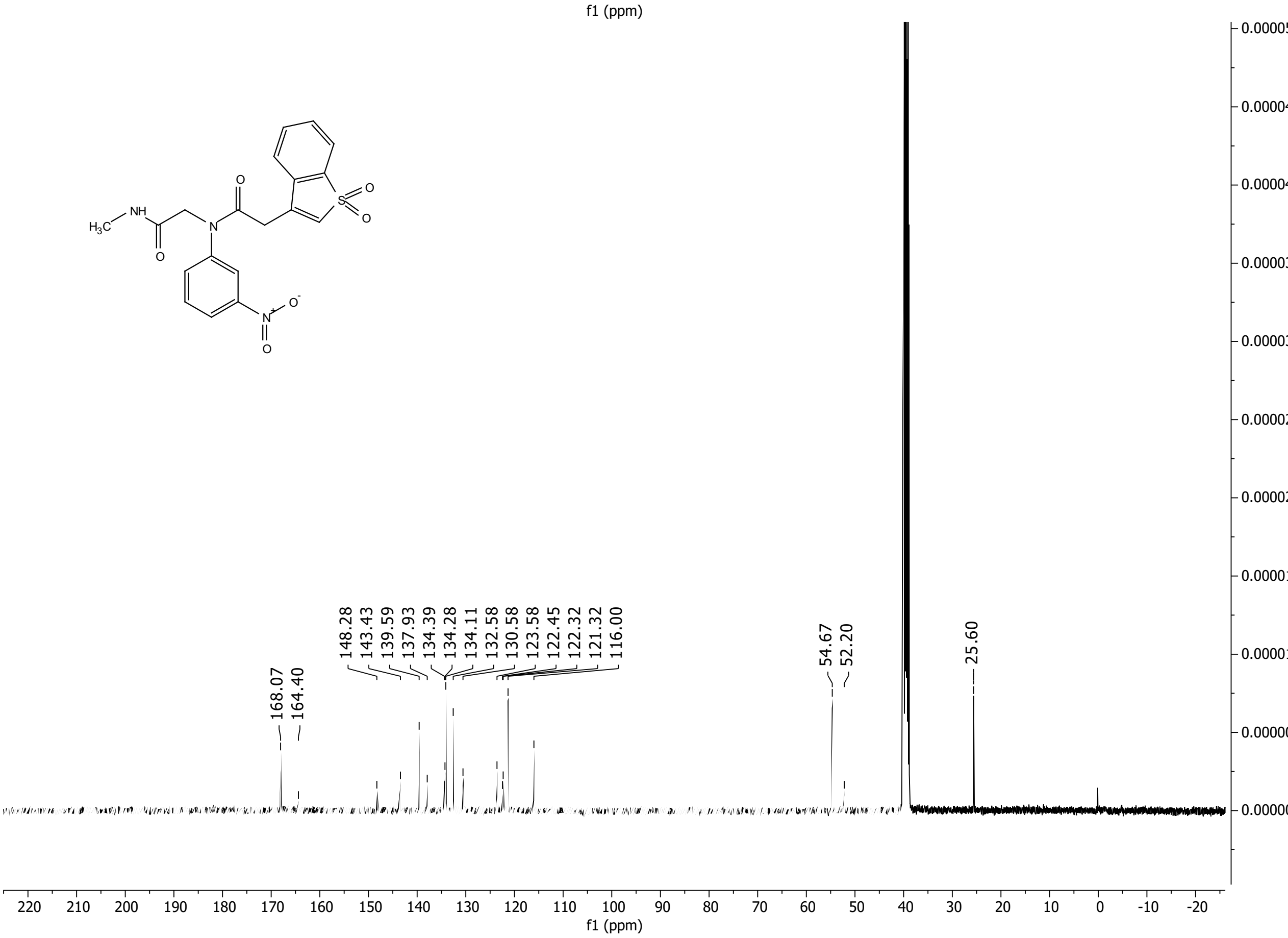
168.07 164.40
148.28 143.43 139.59 137.93 134.39 134.28 134.11 132.58 130.58 123.58 122.45 122.32 121.32 116.00
54.67 52.20
25.60
f1 (ppm)

2m

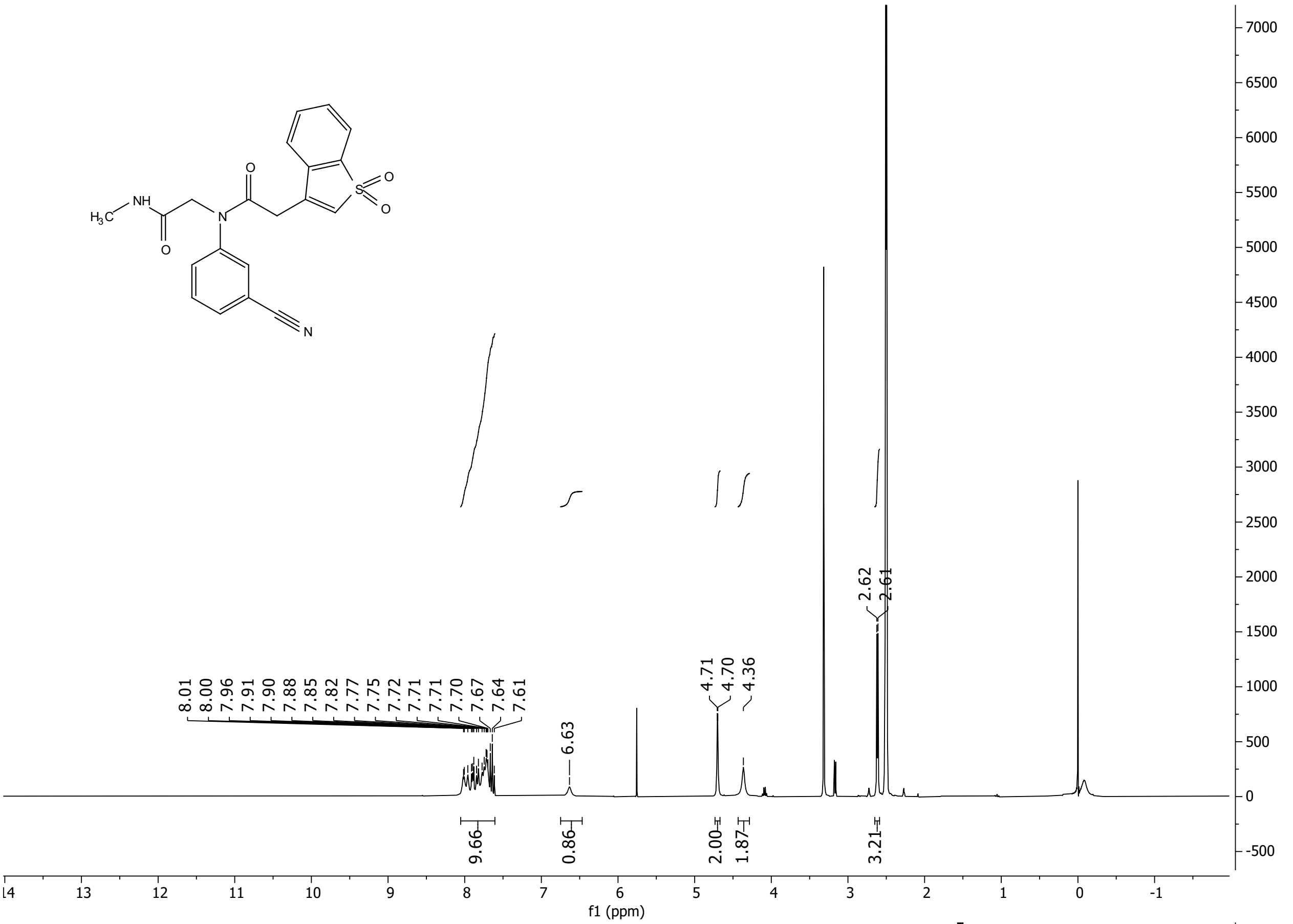

8.01 8.00 7.96 7.91 7.90 7.88 7.85 7.82 7.77 7.75 7.72 7.71 7.71 7.70 7.67 7.64 7.61
6.63
4.71 4.70 4.36
2.62 2.61
9.66 0.86 2.00 1.87 3.21
f1 (ppm)

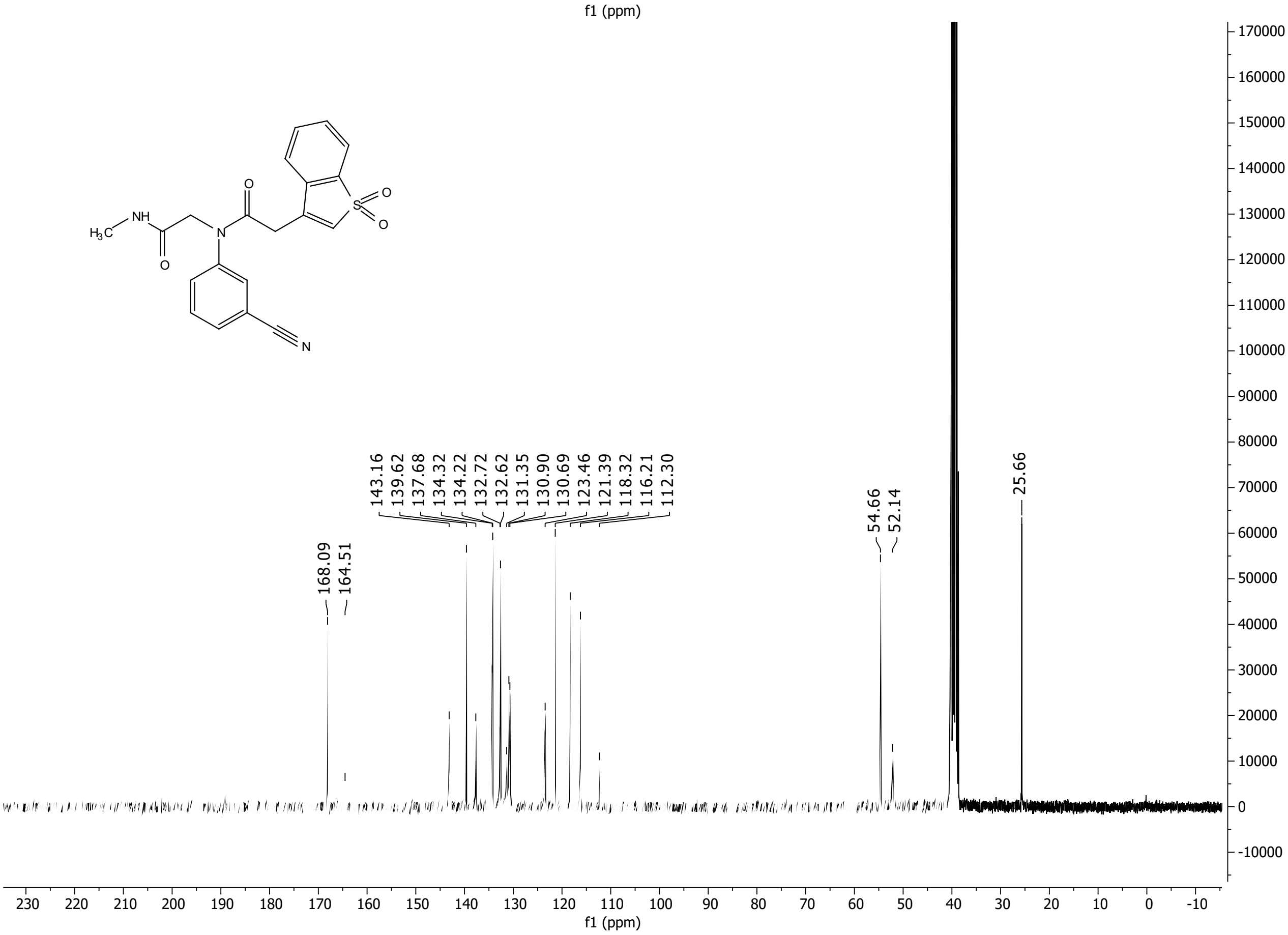

168.09 164.51
143.16 139.62 137.68 134.32 134.22 132.72 132.62 131.35 130.90 130.69 123.46 121.39 118.32 116.21 112.30
54.66 52.14
25.66
f1 (ppm)

2n

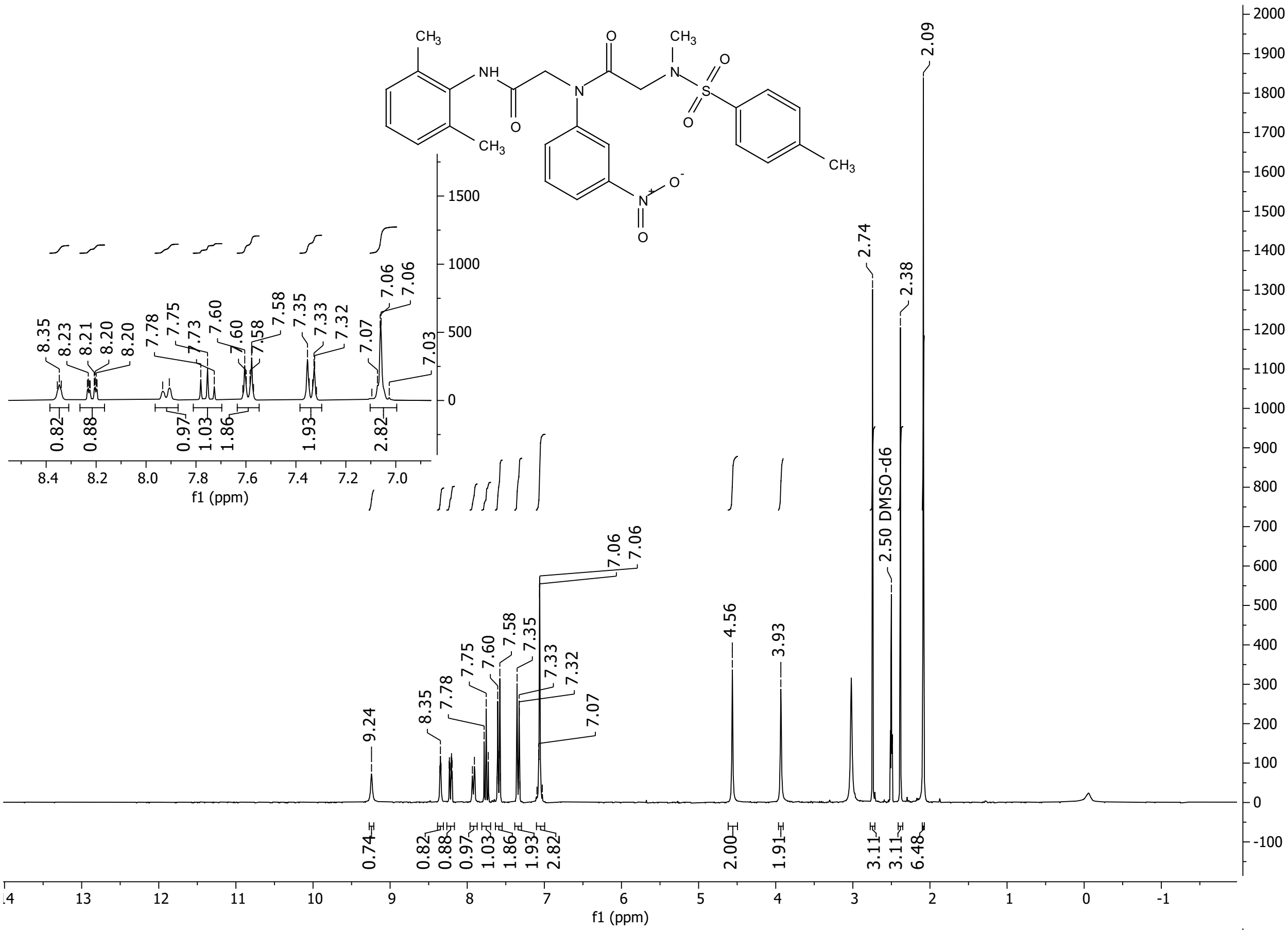

CH3
NH
O
N
O
CH3
N
O
S
O
CH3
CH3
N+
O-
O
9.24
8.35
7.78
7.75
7.60
7.58
7.35
7.33
7.32
7.07
7.06
7.06
4.56
3.93
2.74
2.50 DMSO-d6
2.38
2.09
0.74
0.82
0.88
0.97
1.03
1.86
1.93
2.82
2.00
1.91
3.11
3.11
6.48
8.35
8.23
8.21
8.20
8.20
7.78
7.75
7.73
7.60
7.60
7.58
7.58
7.35
7.33
7.32
7.07
7.06
7.06
7.03
f1 (ppm)

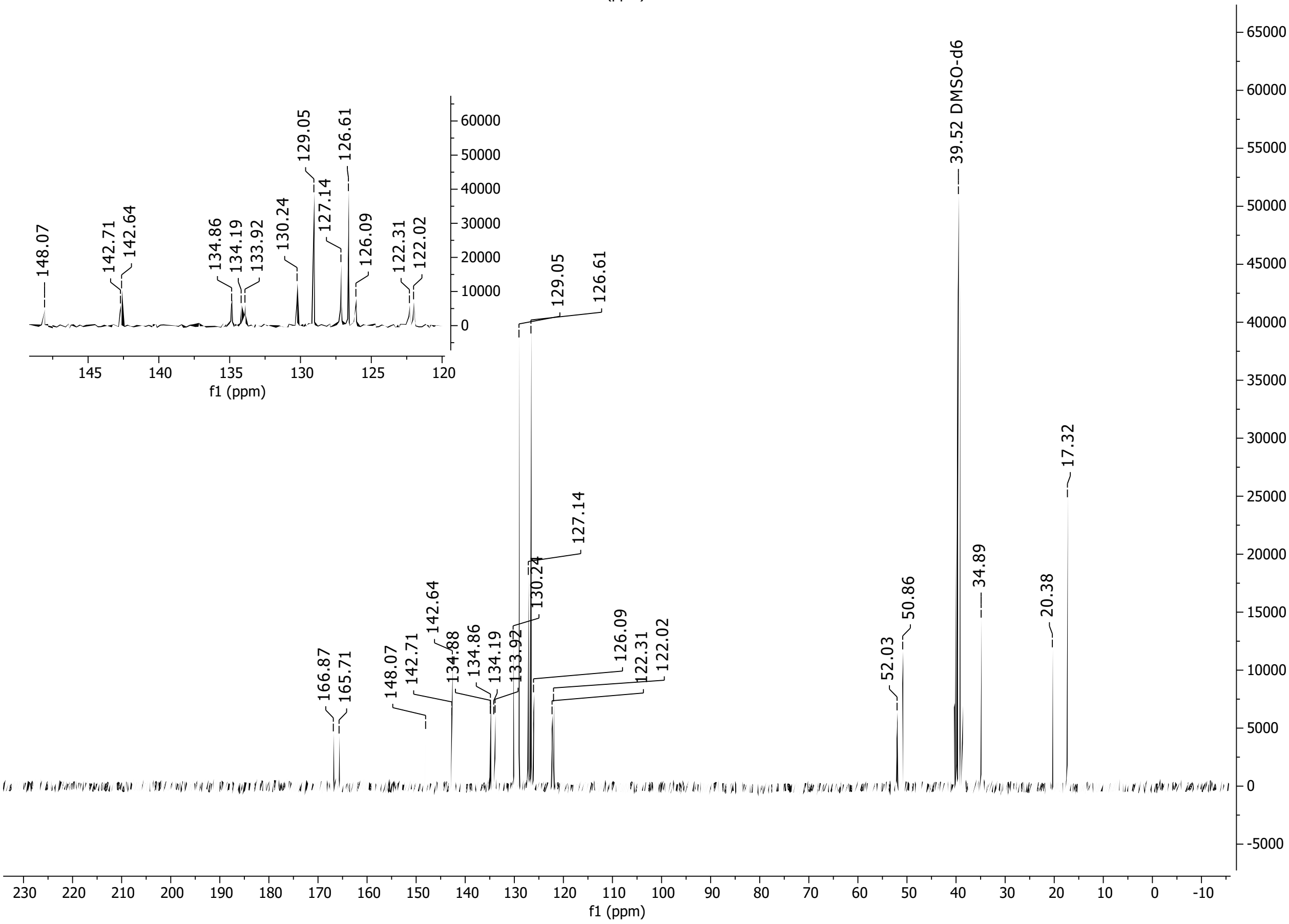

166.87
165.71
148.07
142.71
142.64
134.88
134.86
134.19
133.92
130.24
129.05
127.14
126.61
126.09
122.31
122.02
52.03
50.86
39.52 DMSO-d6
34.89
20.38
17.32
f1 (ppm)

2o

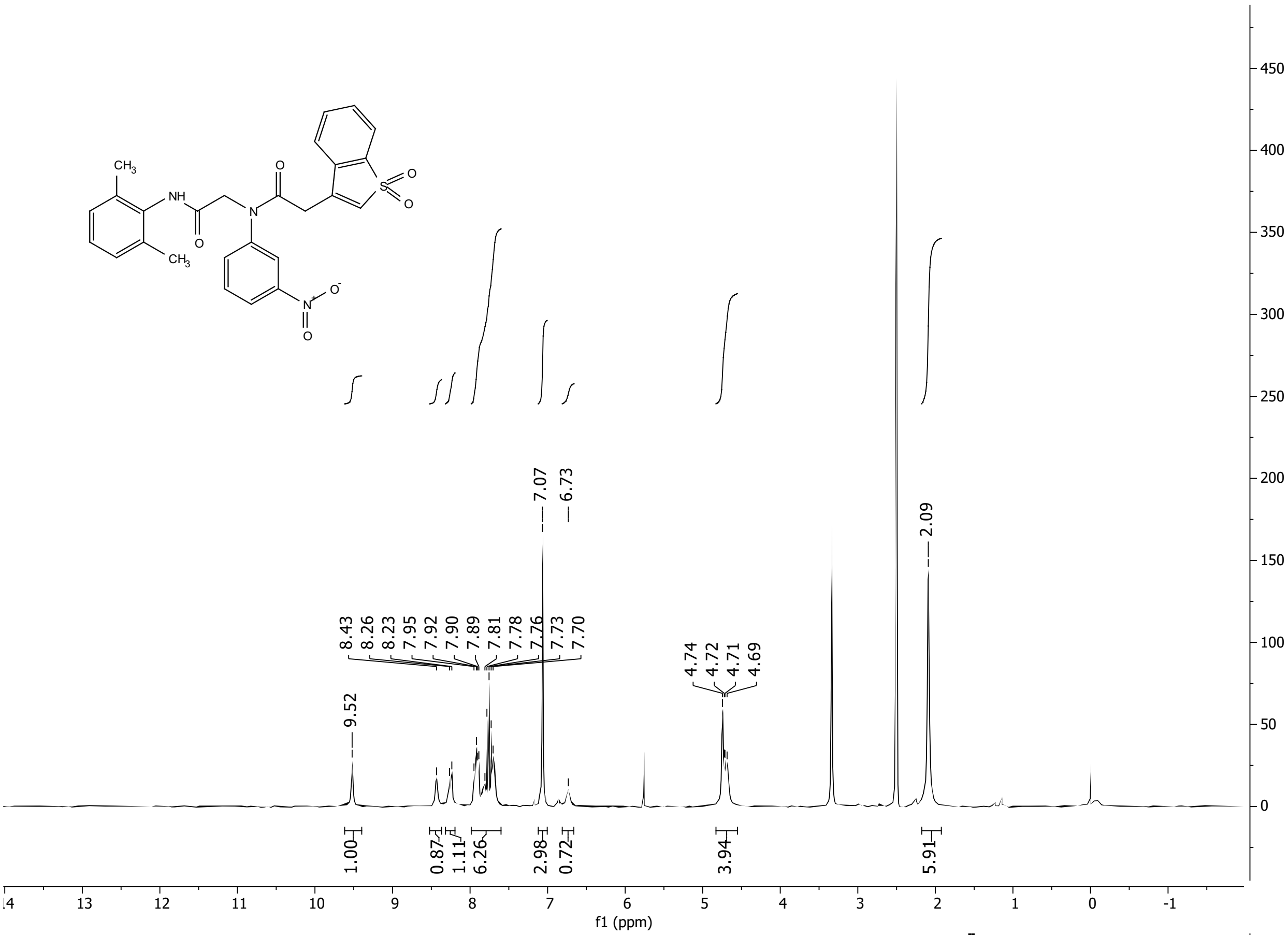

9.52
8.43
8.26
8.23
7.95
7.92
7.90
7.89
7.81
7.78
7.76
7.73
7.70
7.07
6.73
4.74
4.72
4.71
4.69
2.09
1.00
0.87
1.11
6.26
2.98
0.72
3.94
5.91
f1 (ppm)

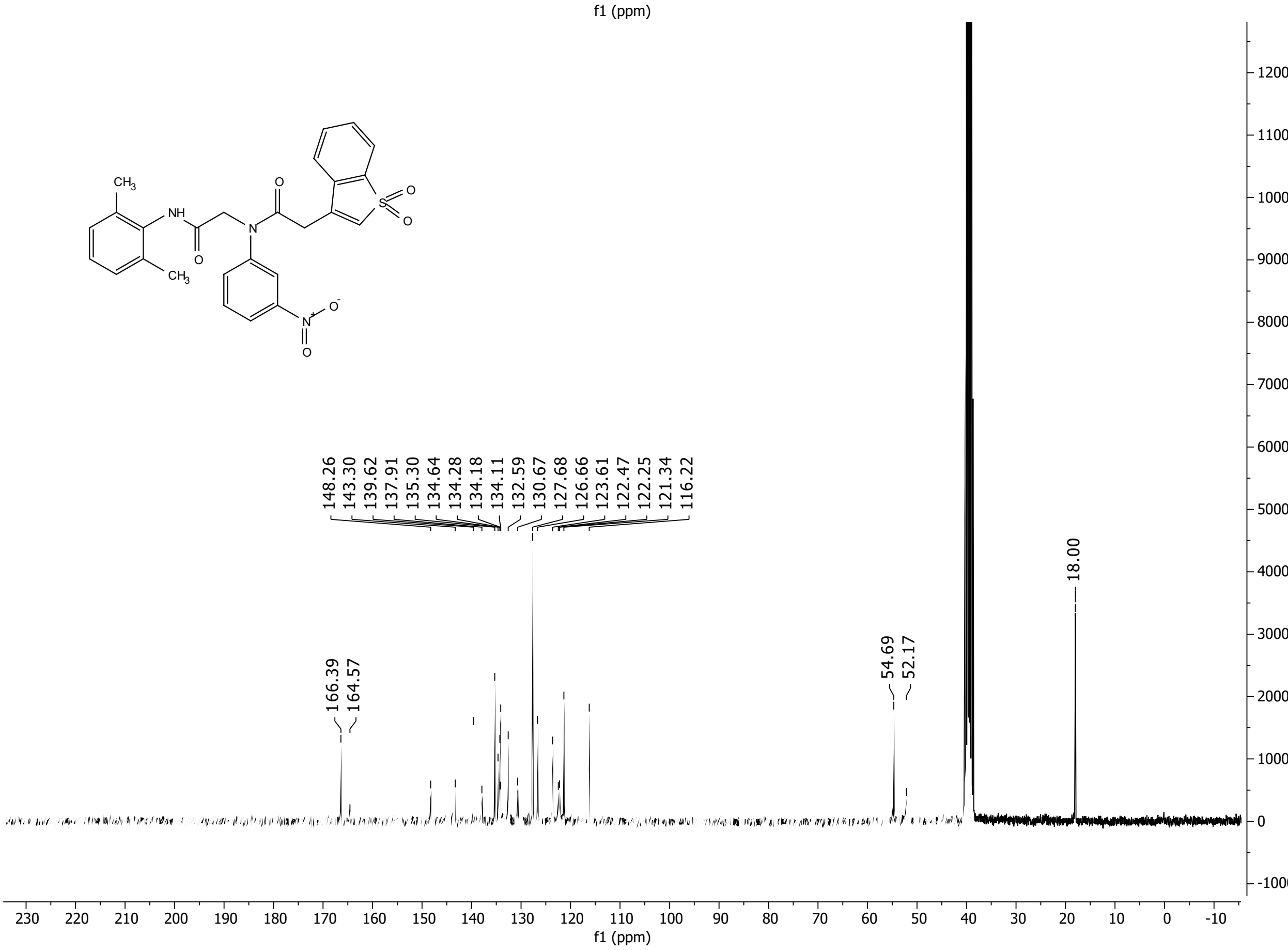

148.26
143.30
139.62
137.91
135.30
134.64
134.28
134.18
134.11
132.59
130.67
127.68
126.66
123.61
122.47
122.25
121.34
116.22
166.39
164.57
54.69
52.17
18.00
f1 (ppm)

2p

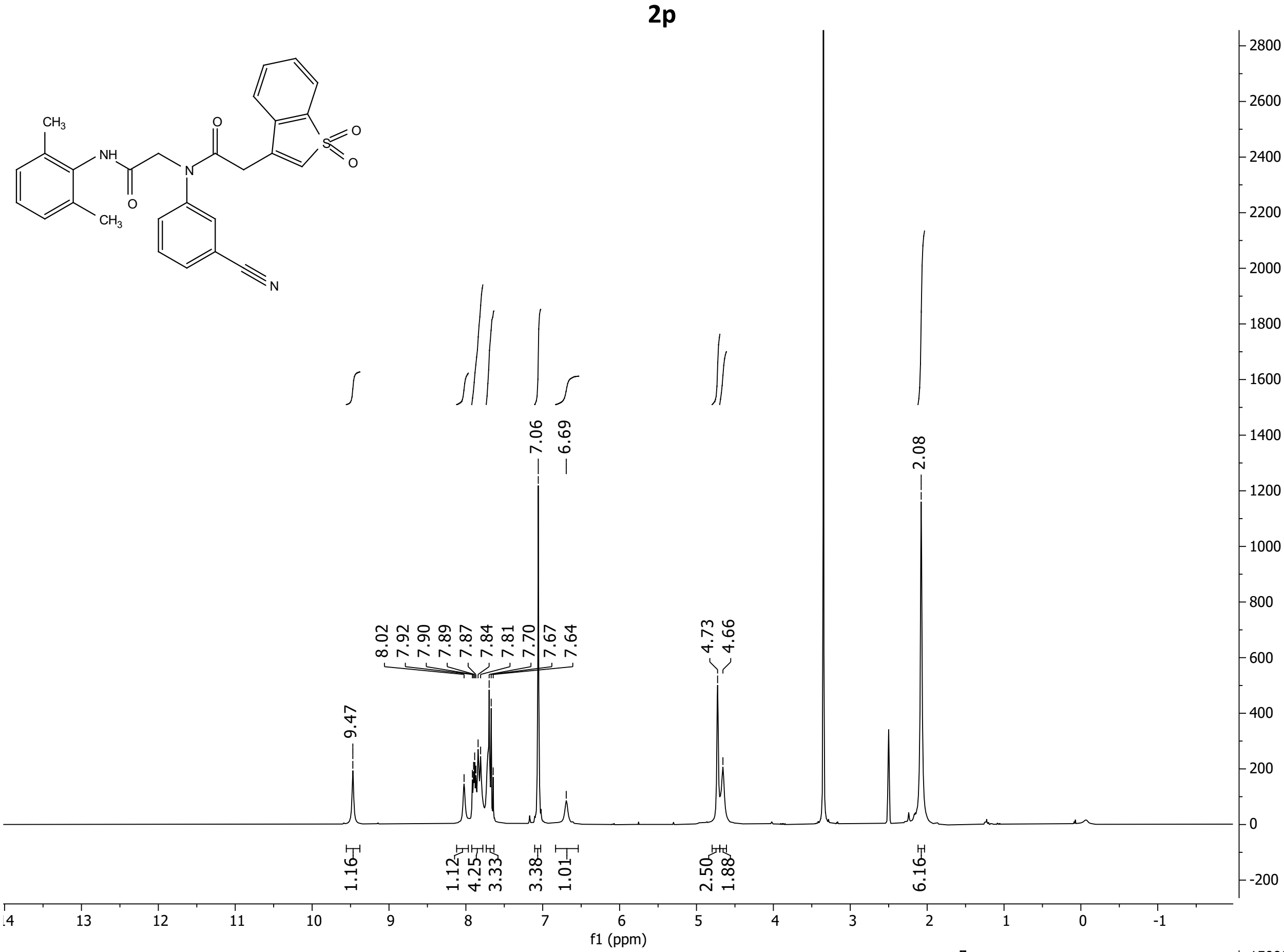
CH3
NH
O
O
N
S
O
O
CH3
N
9.47
8.02
7.92
7.90
7.89
7.87
7.84
7.81
7.70
7.67
7.64
7.06
6.69
4.73
4.66
2.08
1.16
1.12
4.25
3.33
3.38
1.01
2.50
1.88
6.16
f1 (ppm)

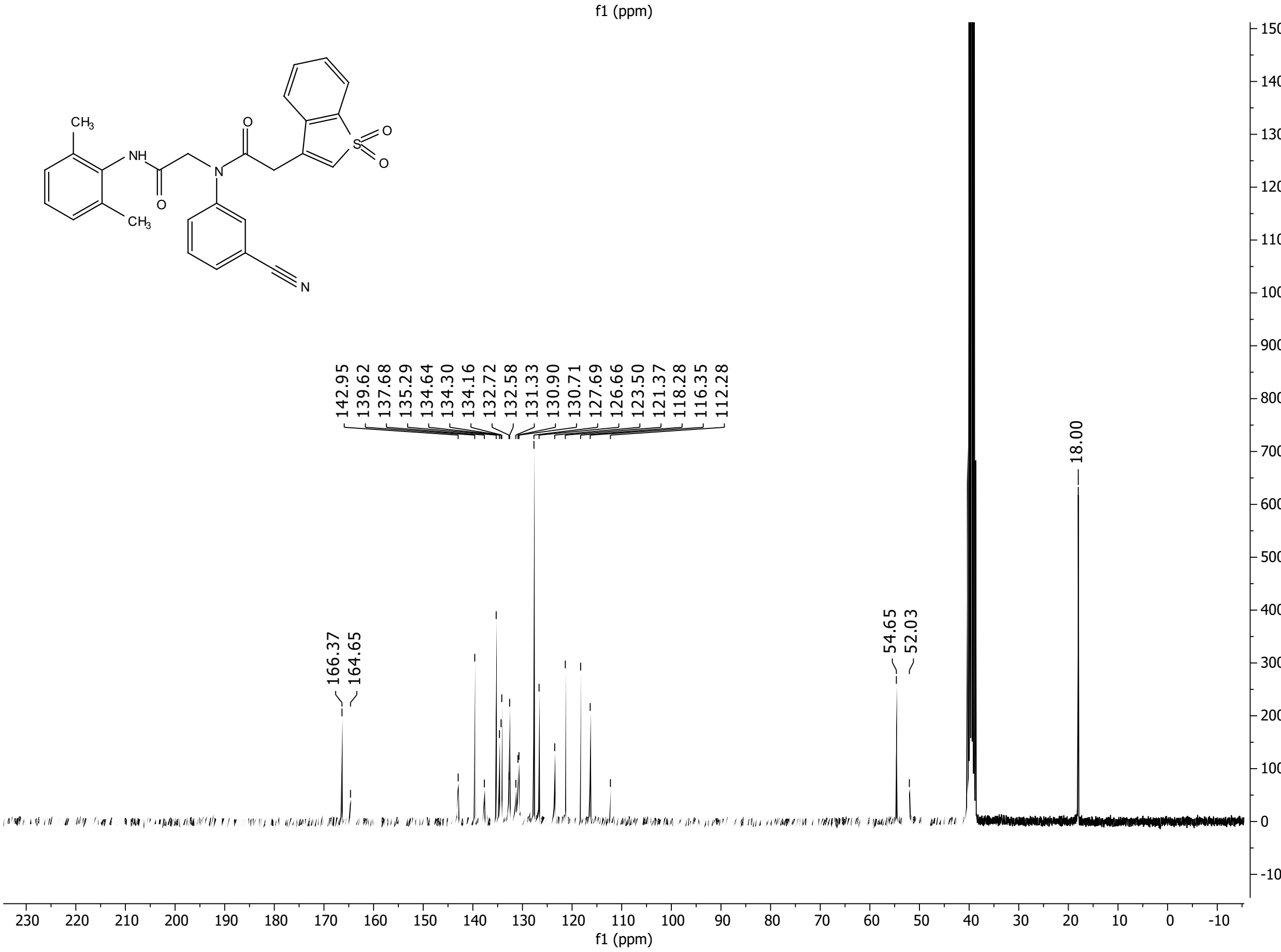
CH3
NH
O
O
N
S
O
O
CH3
N
166.37
164.65
142.95
139.62
137.68
135.29
134.64
134.30
134.16
132.72
132.58
131.33
130.90
130.71
127.69
126.66
123.50
121.37
118.28
116.35
112.28
54.65
52.03
18.00
f1 (ppm)

2q

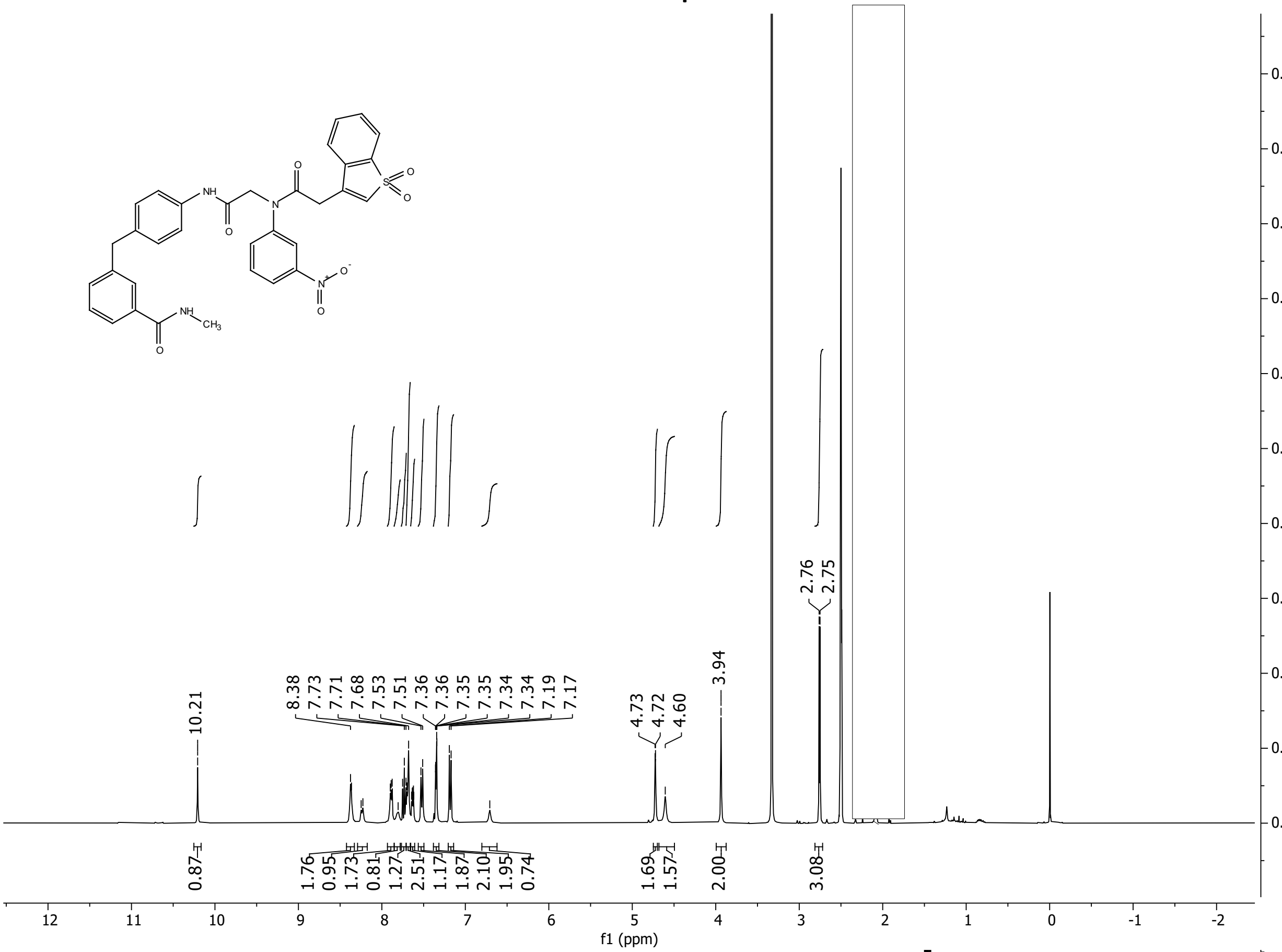
10.21
8.38 7.73 7.71 7.68 7.53 7.51 7.36 7.36 7.35 7.35 7.34 7.34 7.19 7.17
4.73 4.72 4.60
3.94
2.76 2.75
0.87
1.76 0.95 1.73 0.81 1.27 2.51 1.17 1.87 2.10 1.95 0.74
1.69 1.57
2.00
3.08
f1 (ppm)

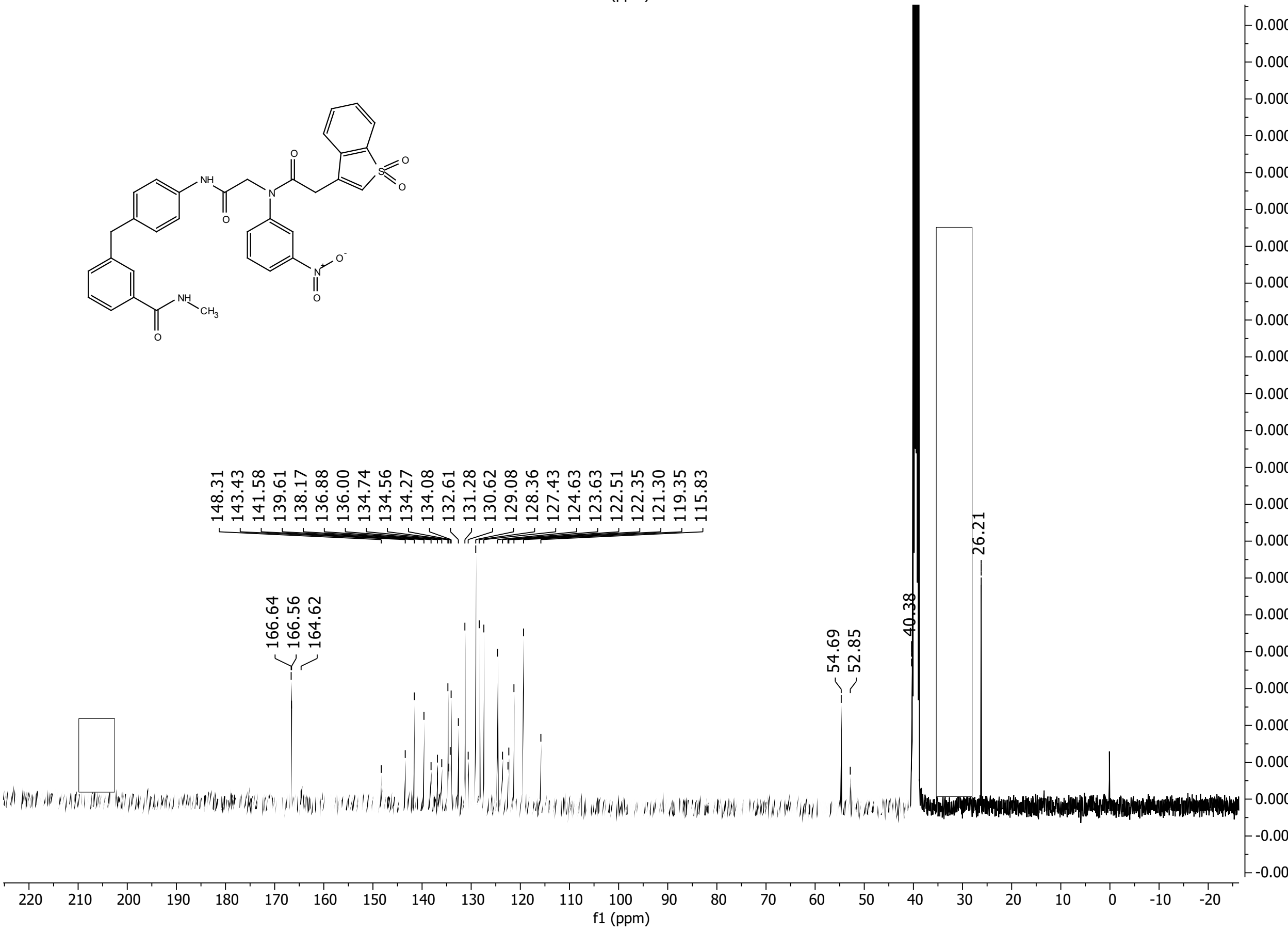
148.31 143.43 141.58 139.61 138.17 136.88 136.00 134.74 134.56 134.27 134.08 132.61 131.28 130.62 129.08 128.36 127.43 124.63 123.63 122.51 122.35 121.30 119.35 115.83
166.64 166.56 164.62
54.69 52.85
40.38
26.21
f1 (ppm)

2r

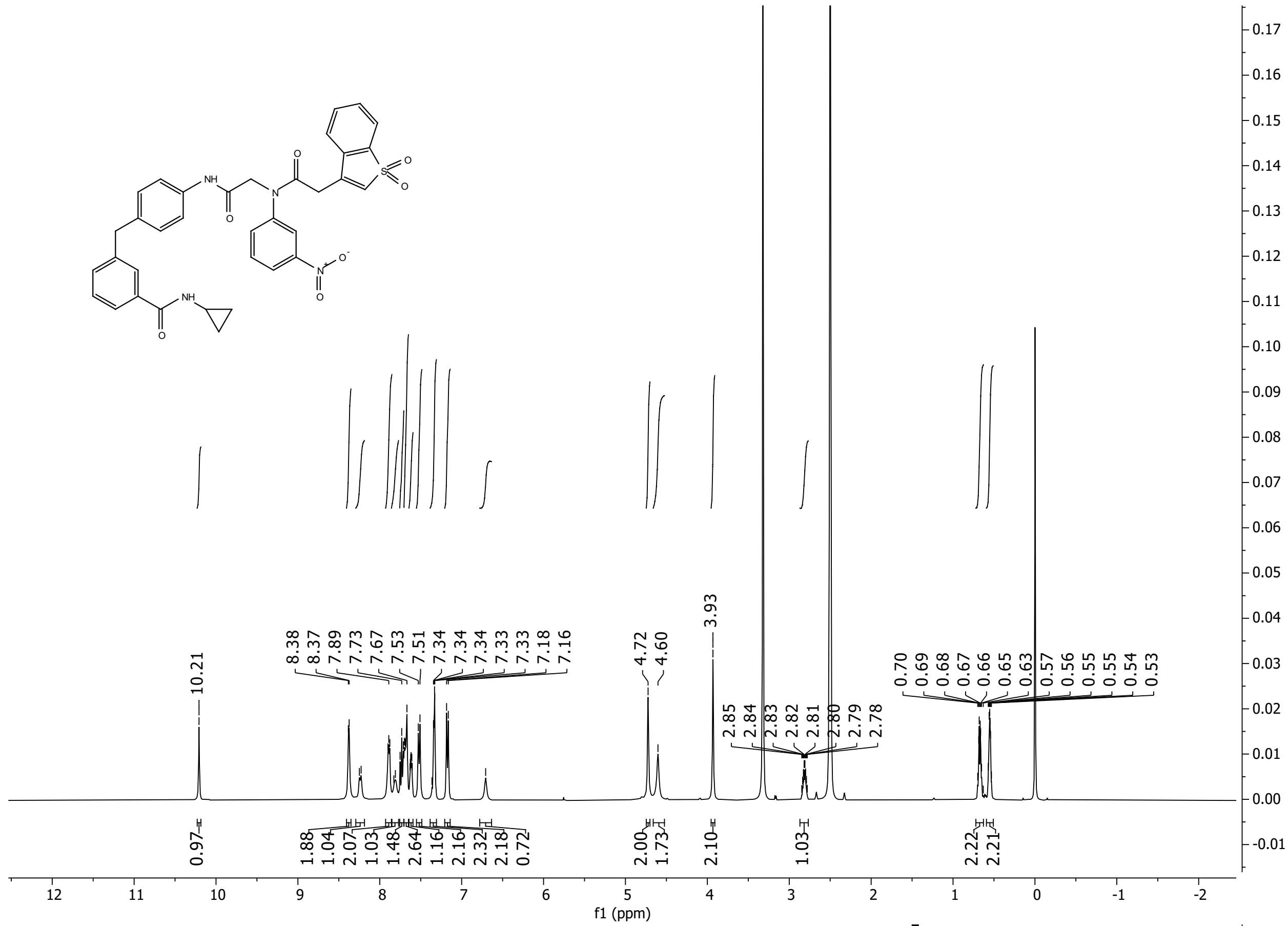

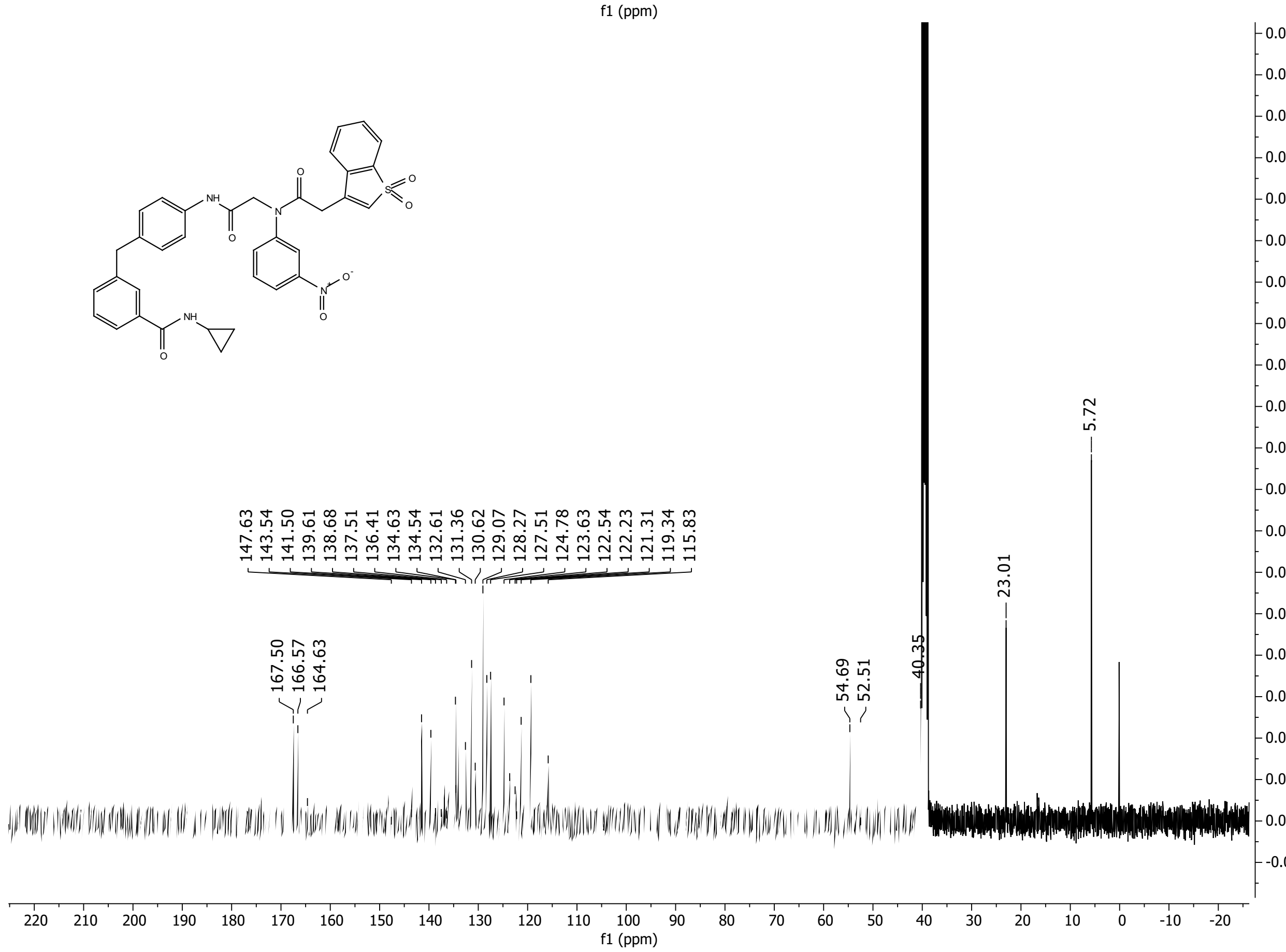

2s

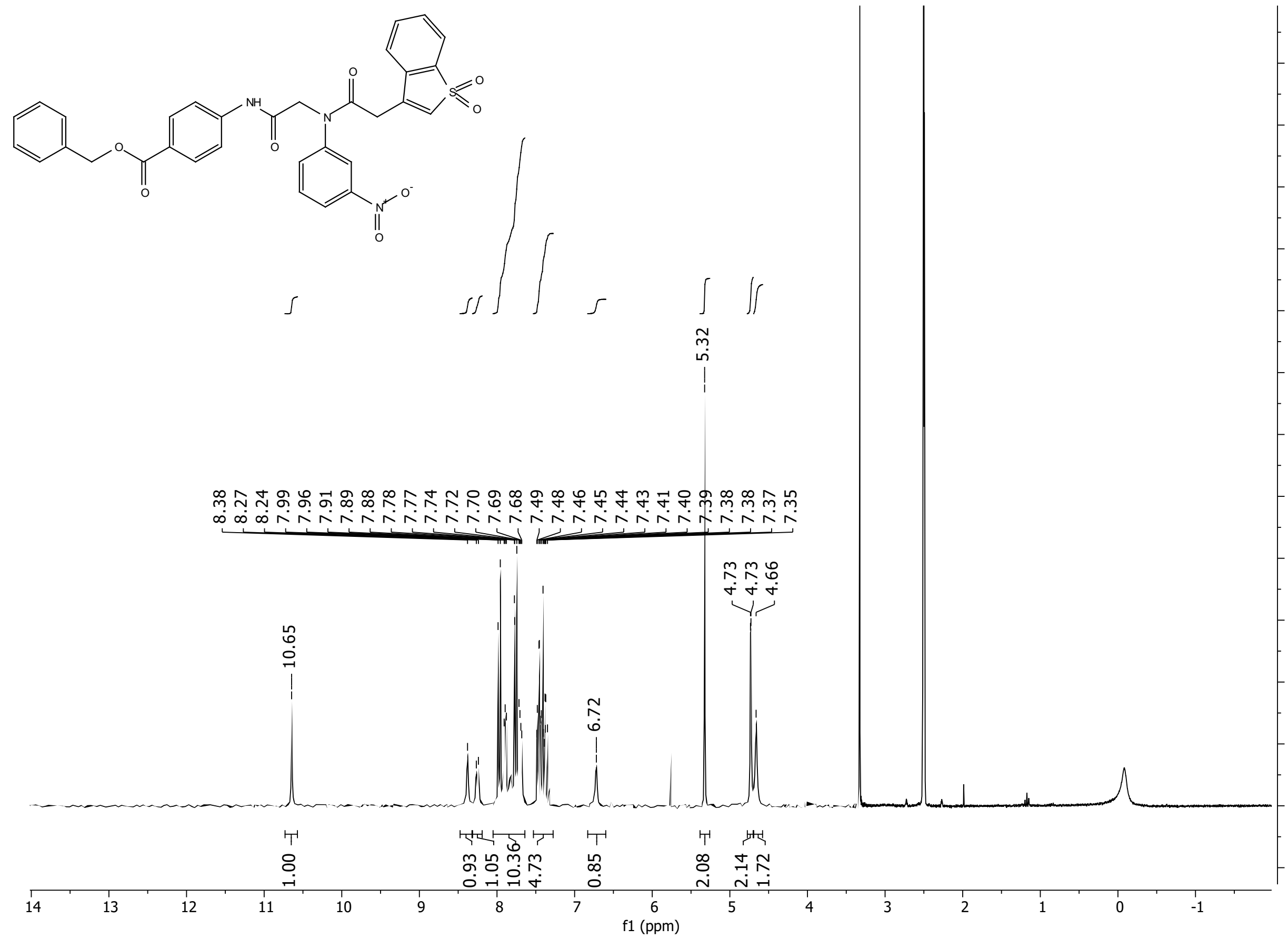

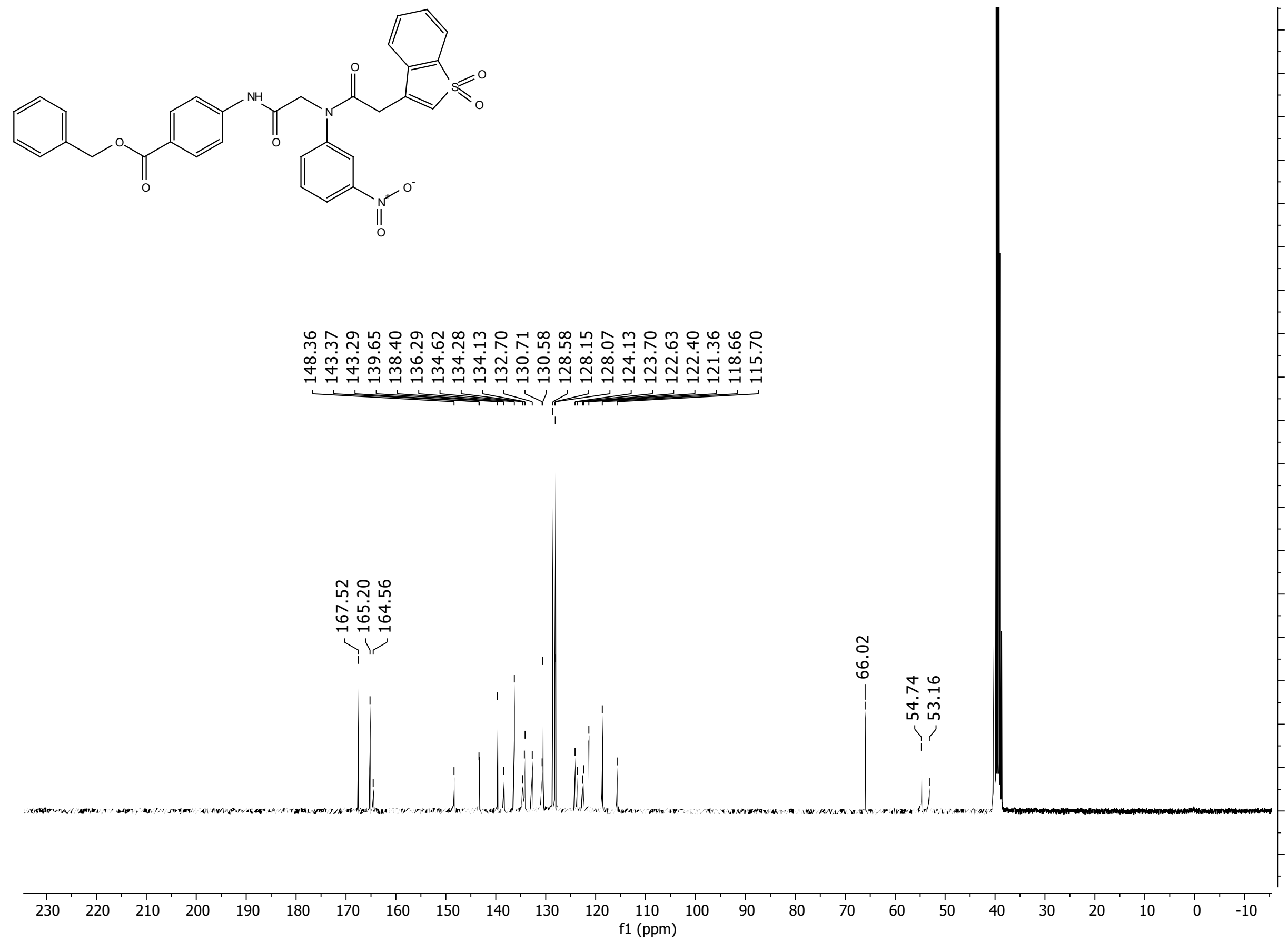

2t

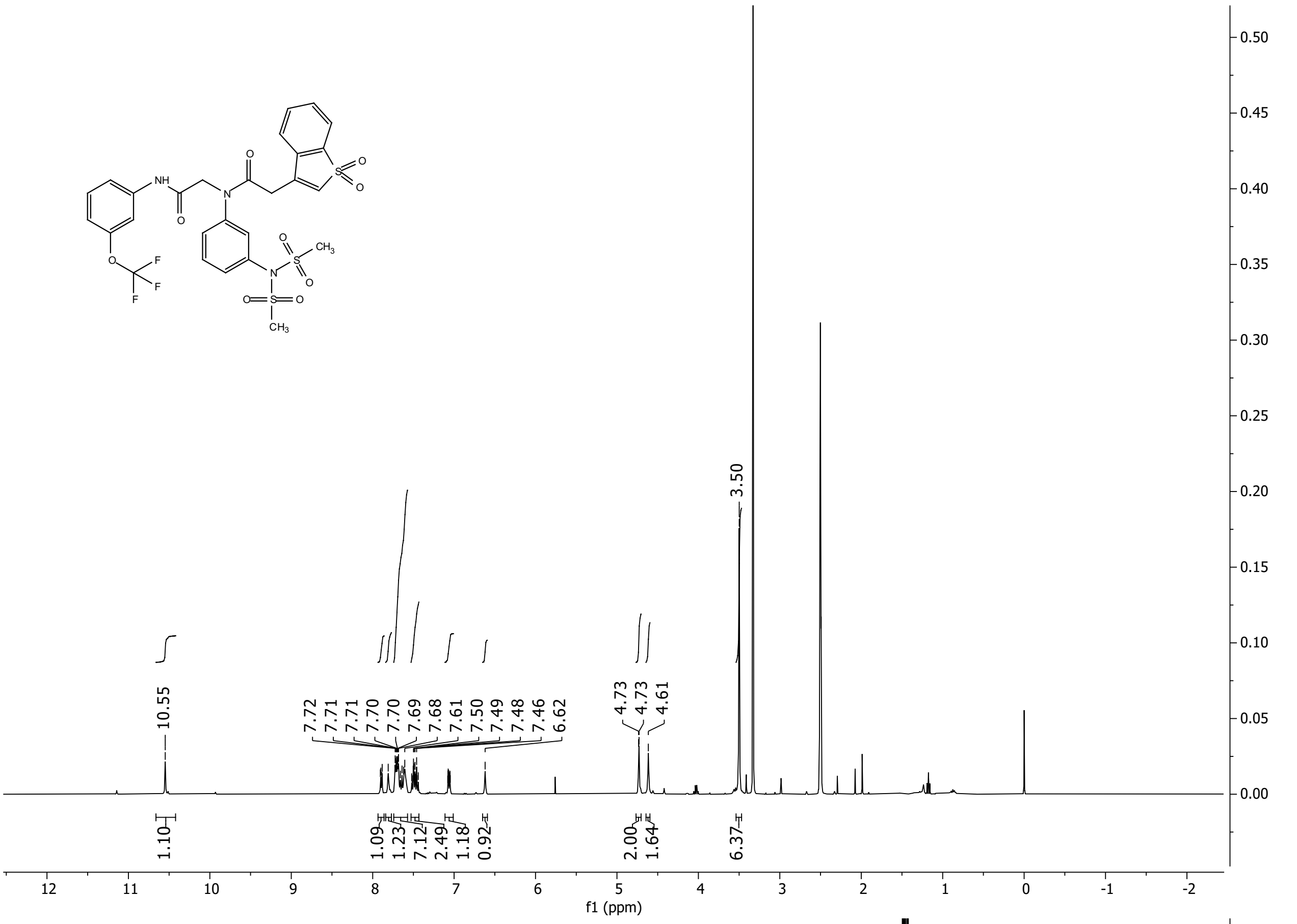

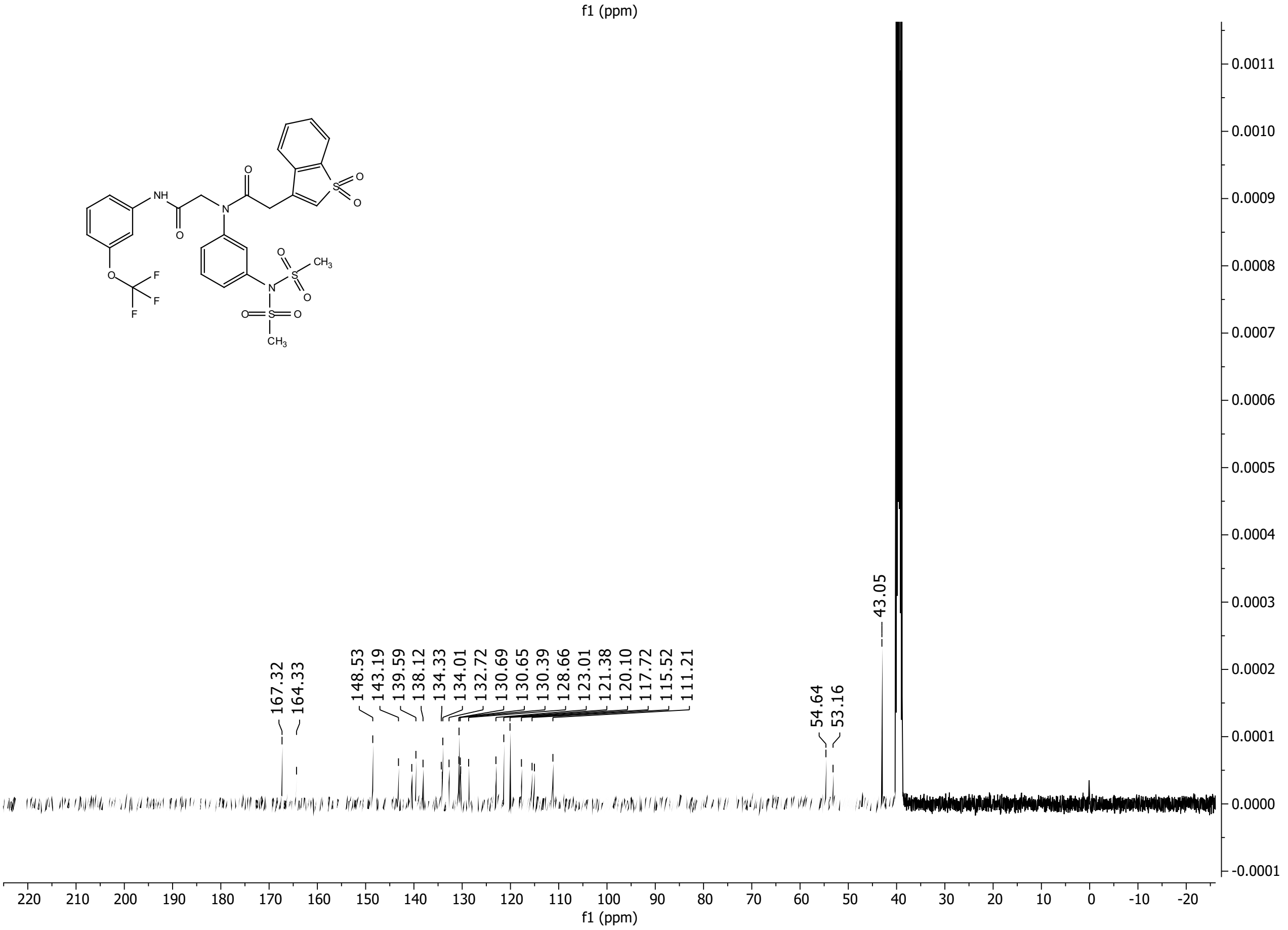

2u

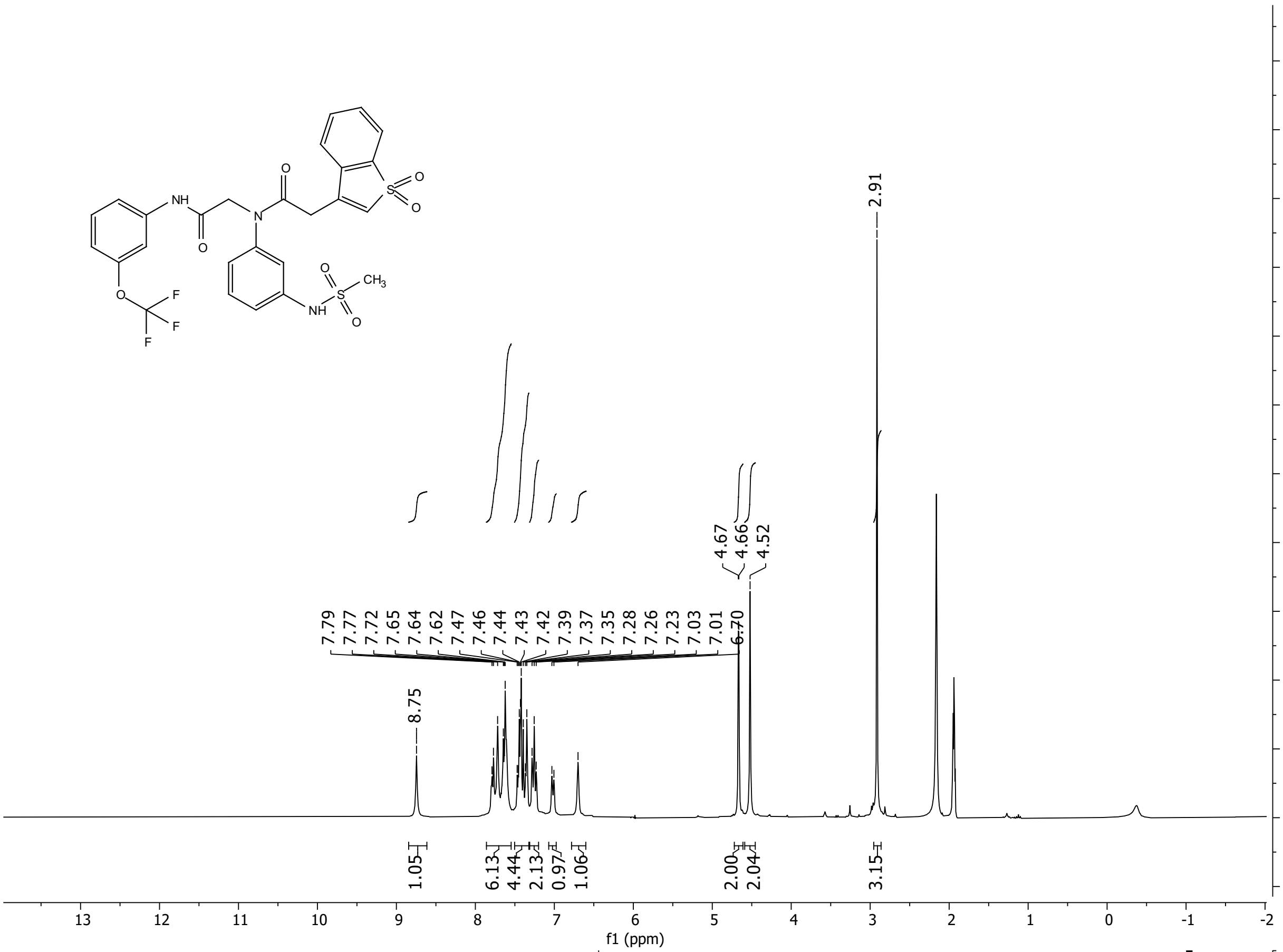
2.91
4.67
4.66
4.52
7.79 7.77 7.72 7.65 7.64 7.62 7.47 7.46 7.44 7.43 7.42 7.39 7.37 7.35 7.28 7.26 7.23 7.03 7.01 6.70
8.75
1.05
6.13
4.44
2.13
0.97
1.06
2.00
2.04
3.15
f1 (ppm)

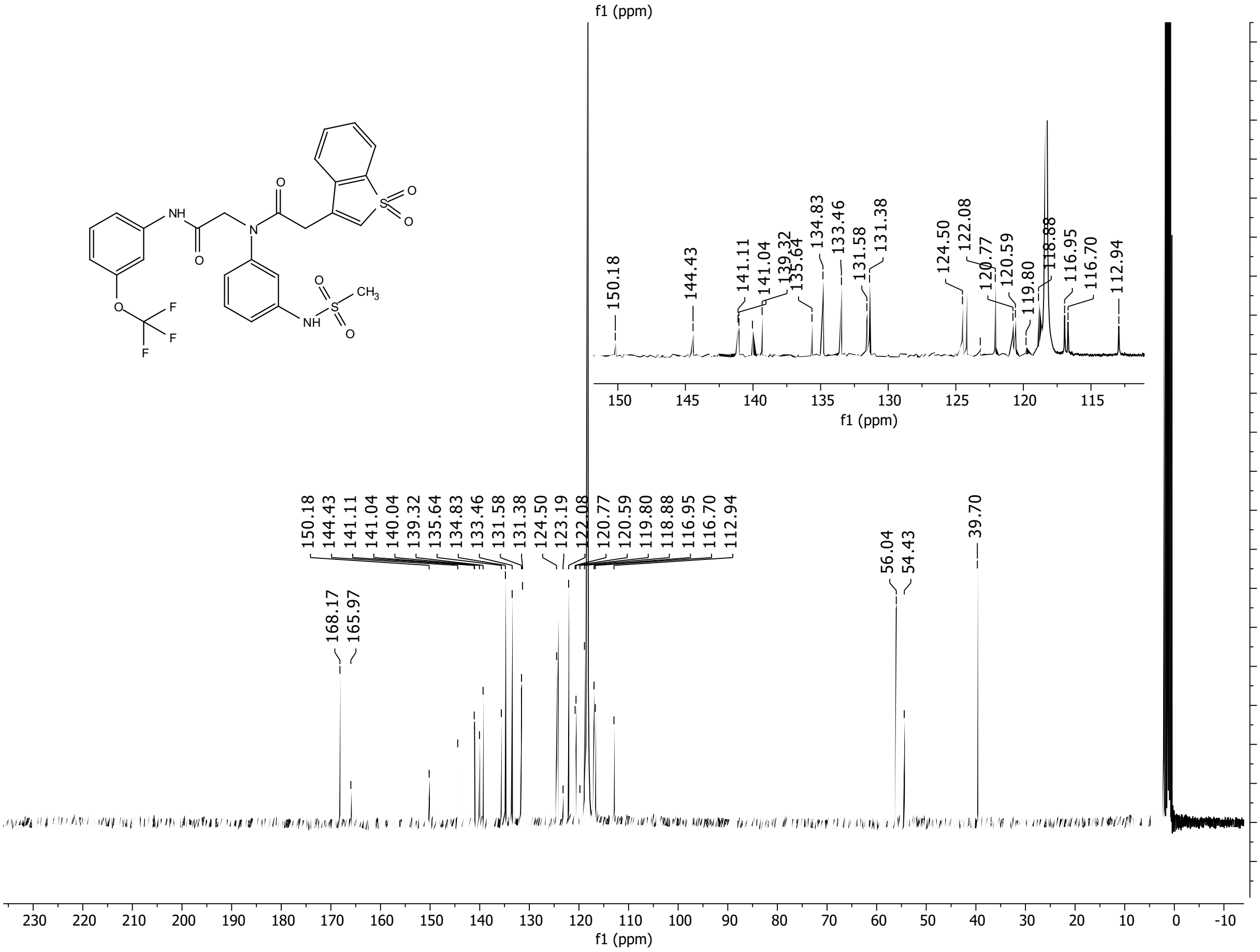
150.18
144.43
141.11
141.04
139.32
135.64
134.83
133.46
131.58
131.38
124.50
122.08
120.77
120.59
119.80
118.88
116.95
116.70
112.94
150.18 144.43 141.11 141.04 140.04 139.32 135.64 134.83 133.46 131.58 131.38 124.50 123.19 122.08 120.77 120.59 119.80 118.88 116.95 116.70 112.94
168.17
165.97
56.04
54.43
39.70
f1 (ppm)

2v

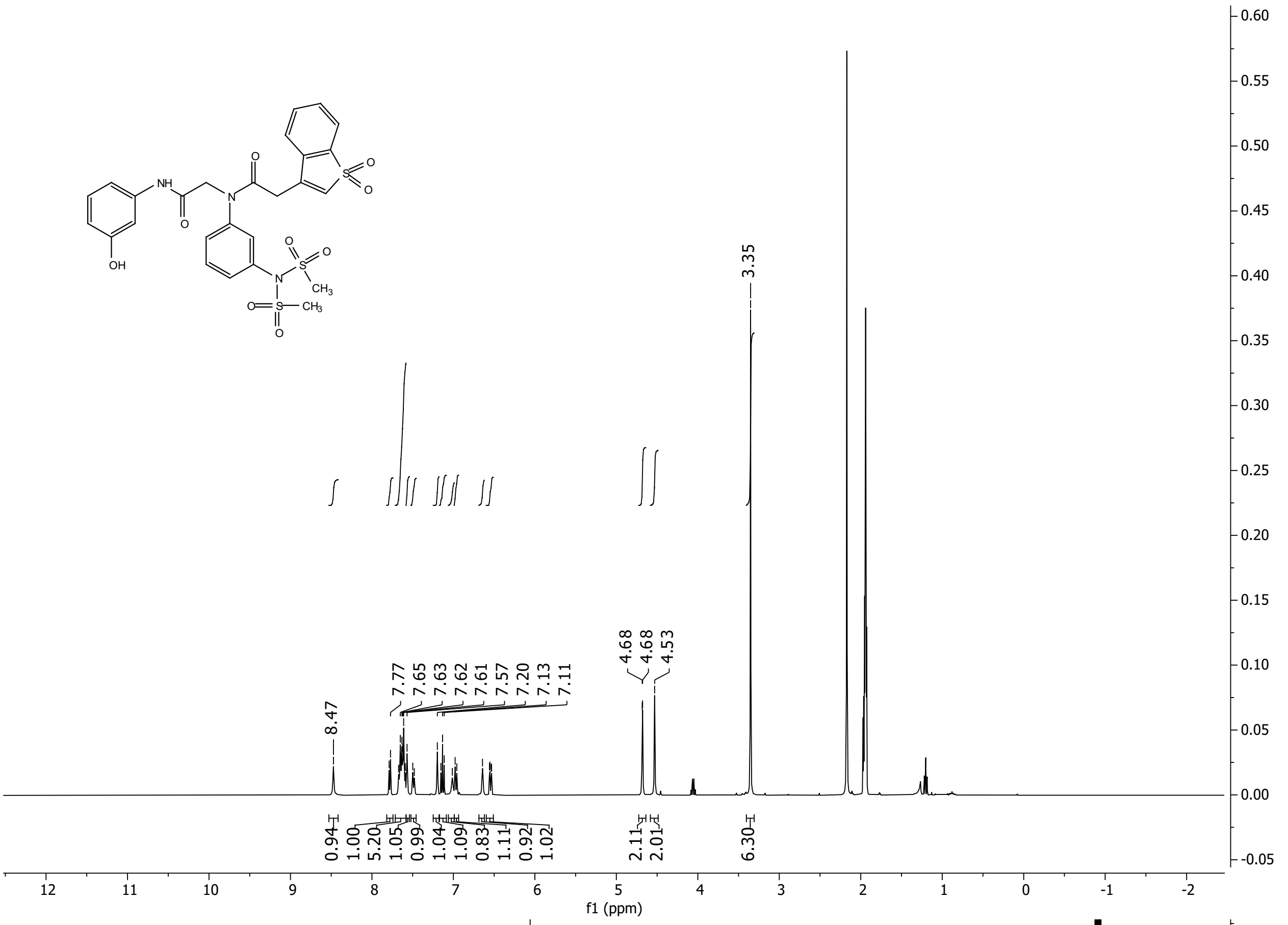

8.47
7.77
7.65
7.63
7.62
7.61
7.57
7.20
7.13
7.11
4.68
4.68
4.53
3.35
0.94
1.00
5.20
1.05
0.99
1.04
1.09
0.83
1.11
0.92
1.02
2.11
2.01
6.30
f1 (ppm)

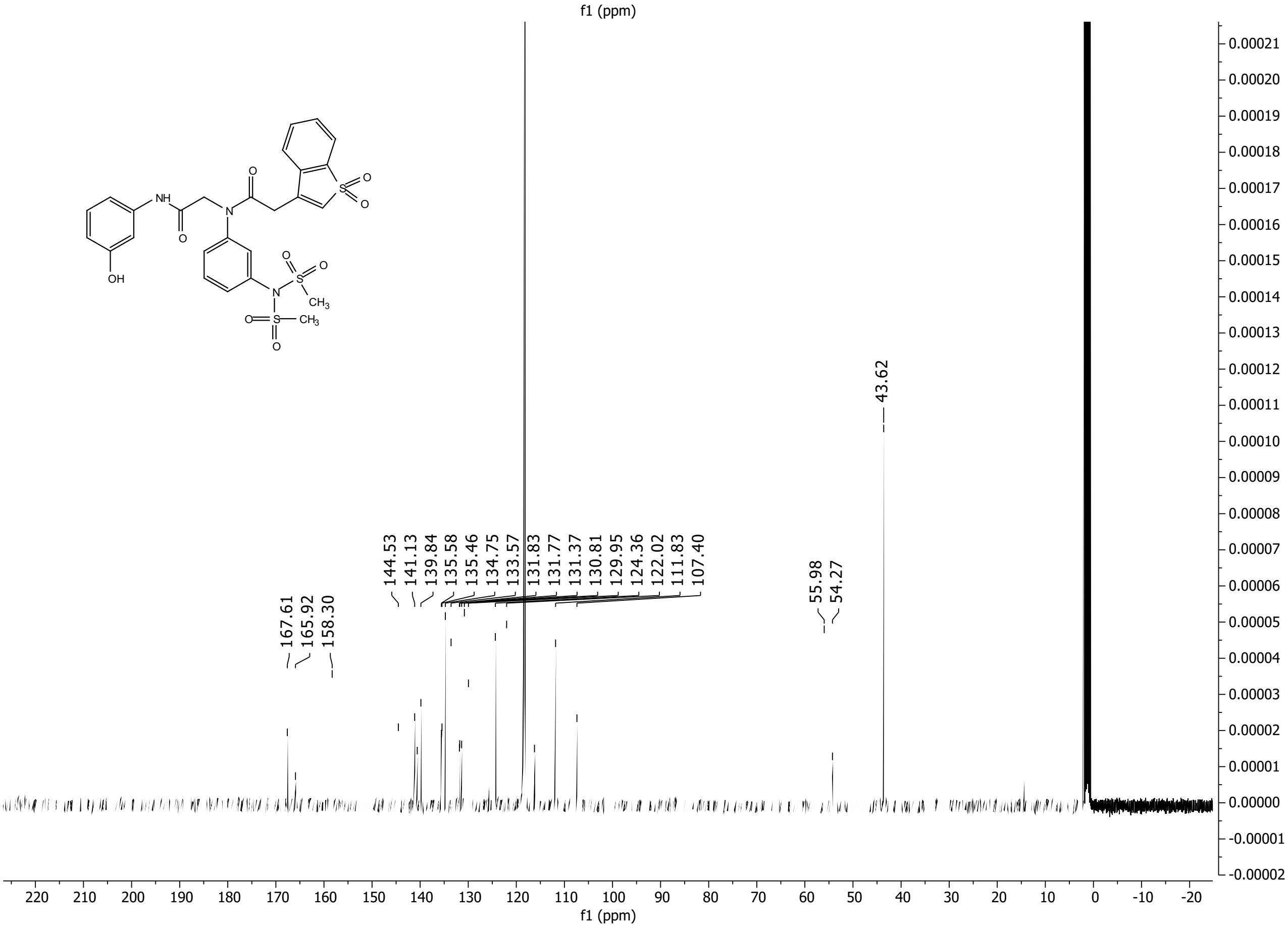

167.61
165.92
158.30
144.53
141.13
139.84
135.58
135.46
134.75
133.57
131.83
131.77
131.37
130.81
129.95
124.36
122.02
111.83
107.40
55.98
54.27
43.62
f1 (ppm)

2w

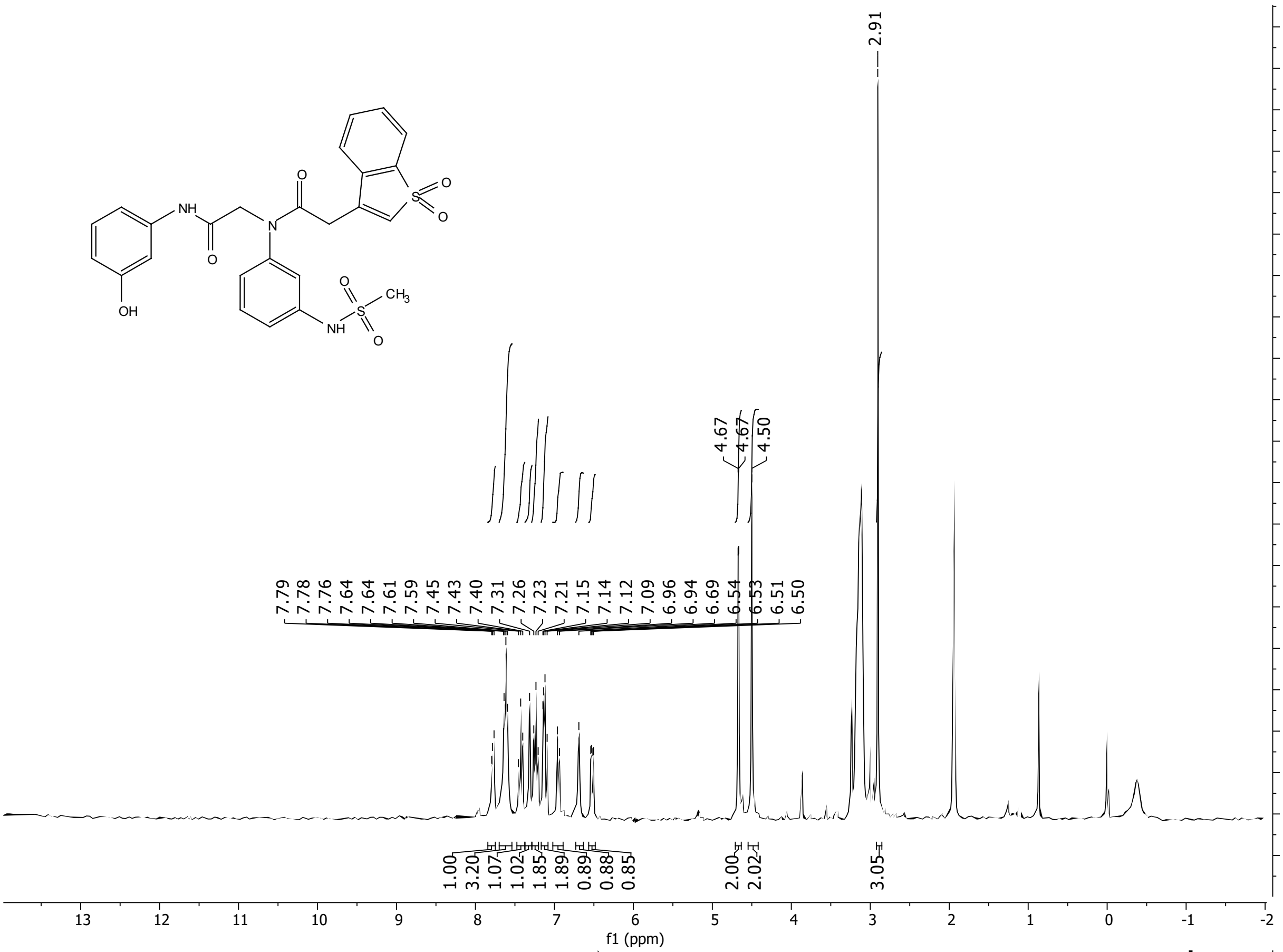

NH
O
N
O
S
O
O
OH
O
S
O
CH3
NH
2.91
4.67
4.67
4.50
7.79
7.78
7.76
7.64
7.64
7.61
7.59
7.45
7.43
7.40
7.31
7.26
7.23
7.21
7.15
7.14
7.12
7.09
6.96
6.94
6.69
6.54
6.53
6.51
6.50
1.00
3.20
1.07
1.02
1.85
1.89
0.89
0.88
0.85
2.00
2.02
3.05
f1 (ppm)

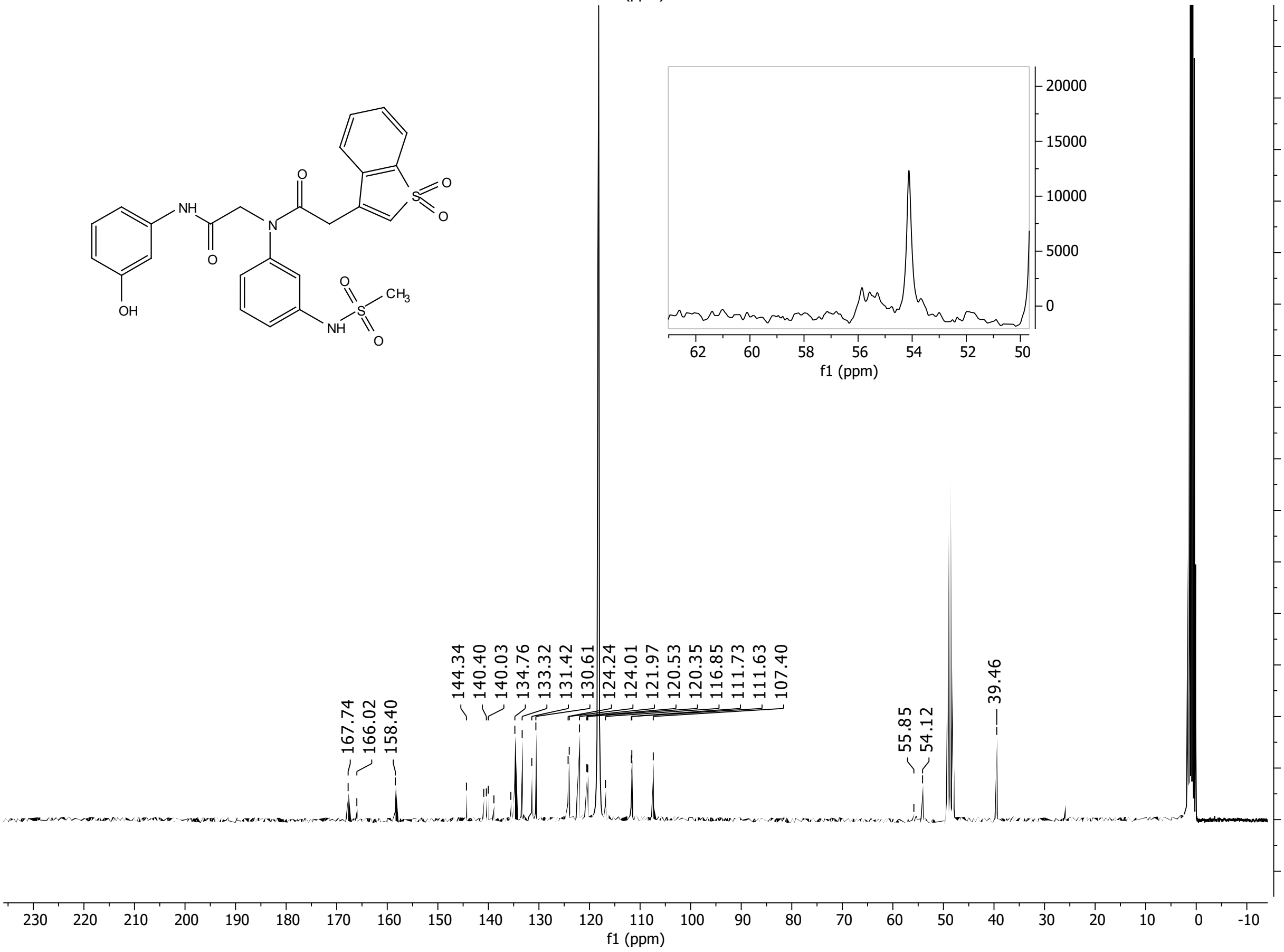

NH
O
N
O
S
O
O
OH
O
S
O
CH3
NH
f1 (ppm)
167.74
166.02
158.40
144.34
140.40
140.03
134.76
133.32
131.42
130.61
124.24
124.01
121.97
120.53
120.35
116.85
111.73
111.63
107.40
55.85
54.12
39.46
f1 (ppm)

2a-P

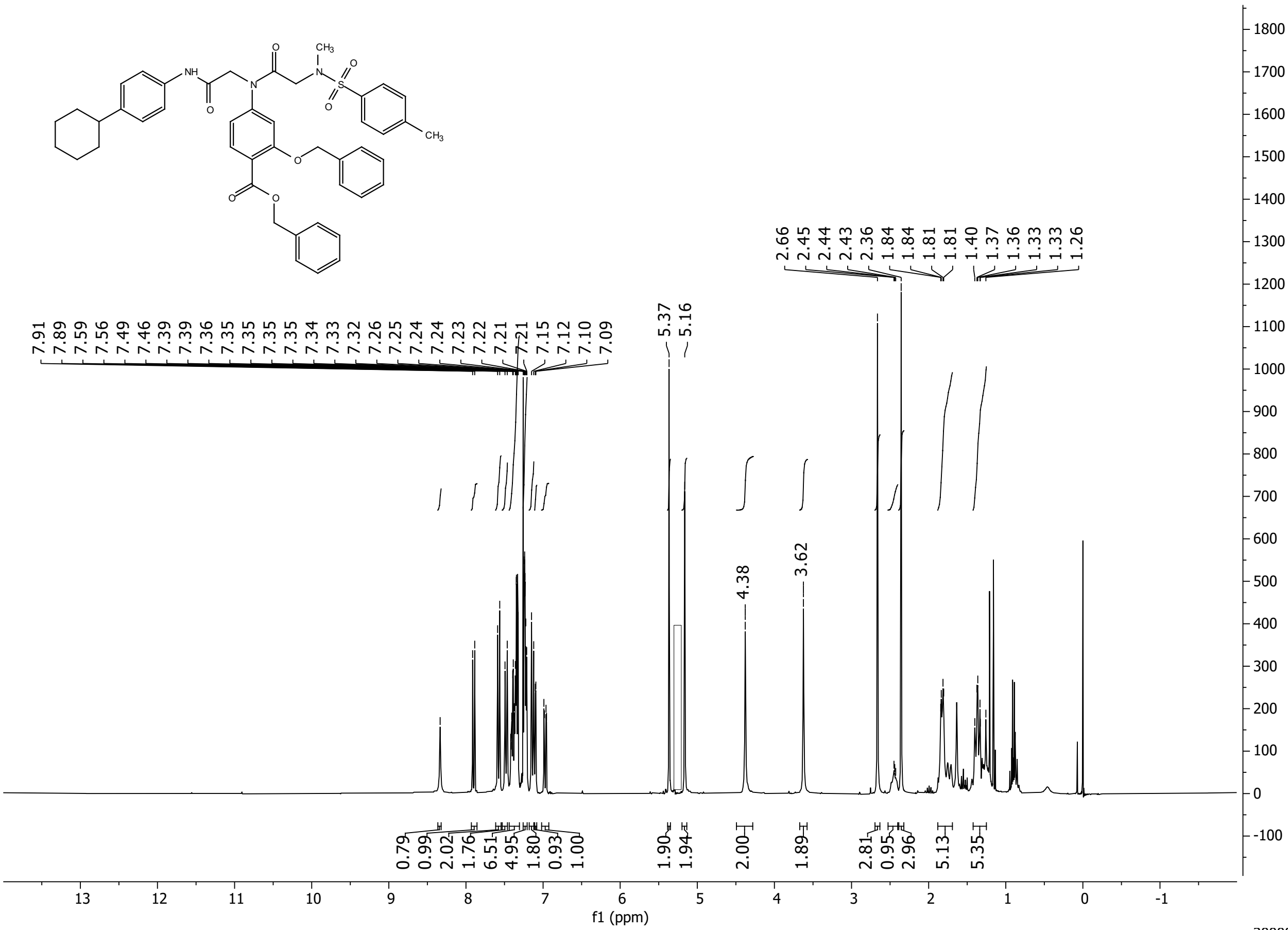

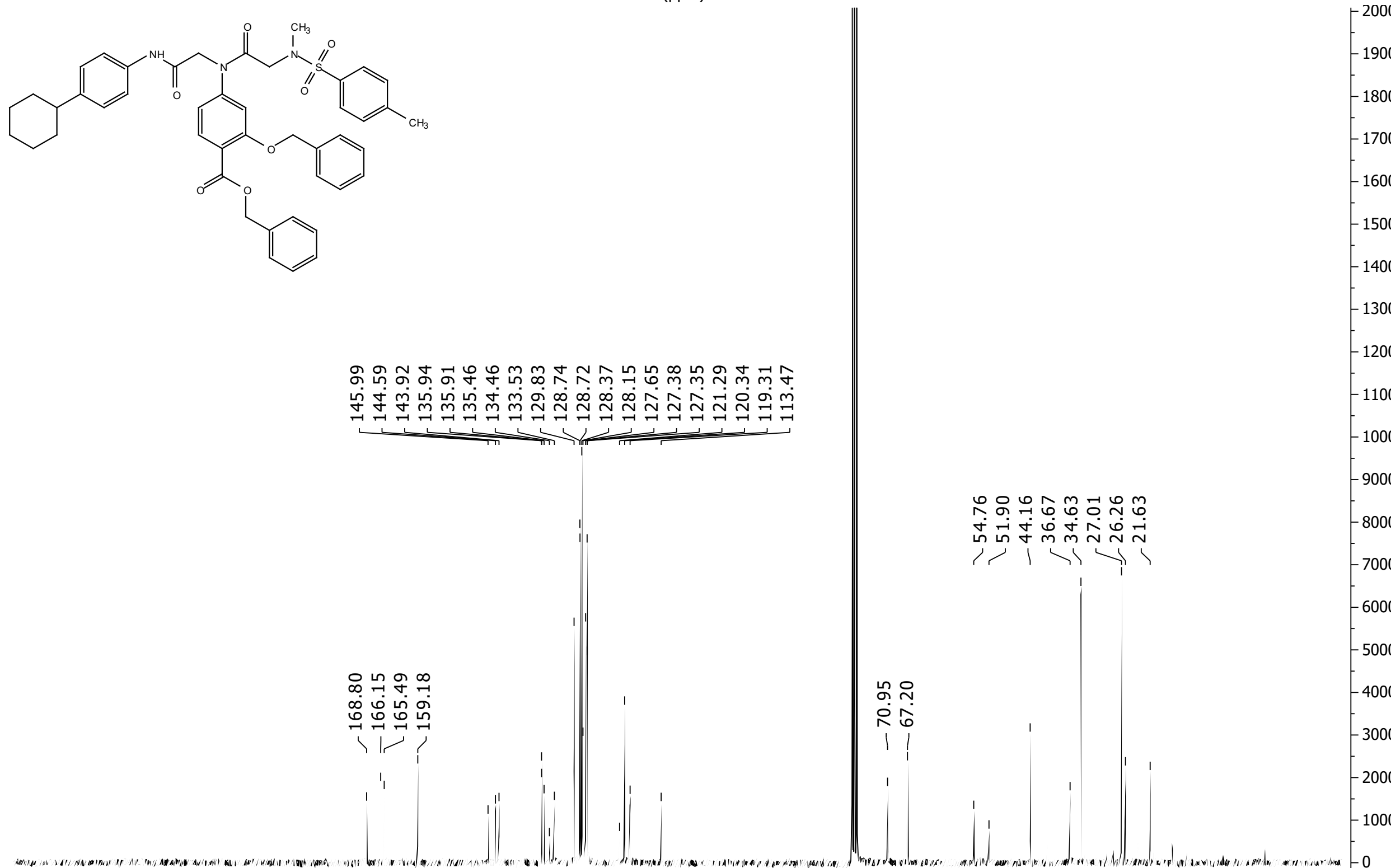

2b-P

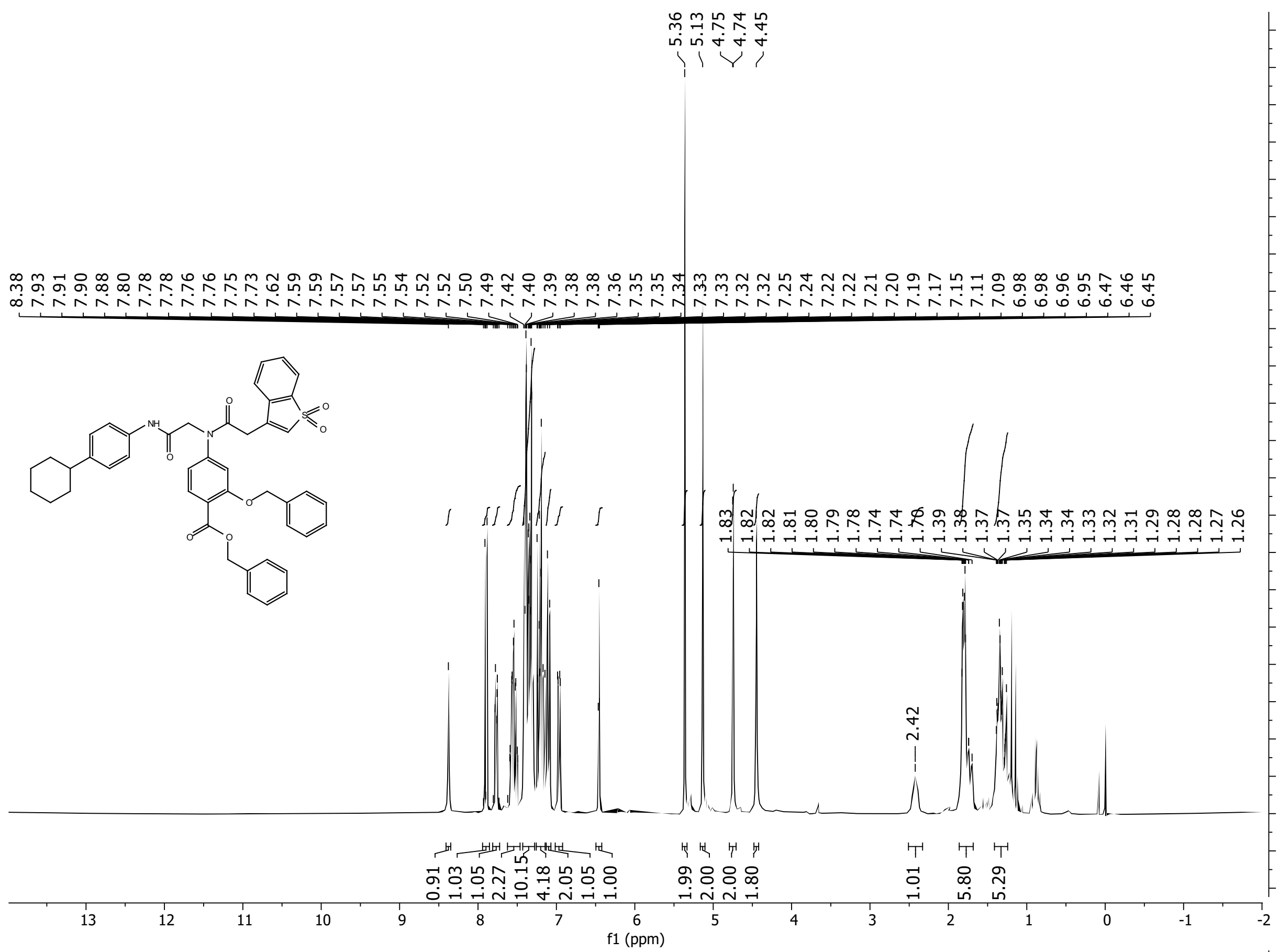

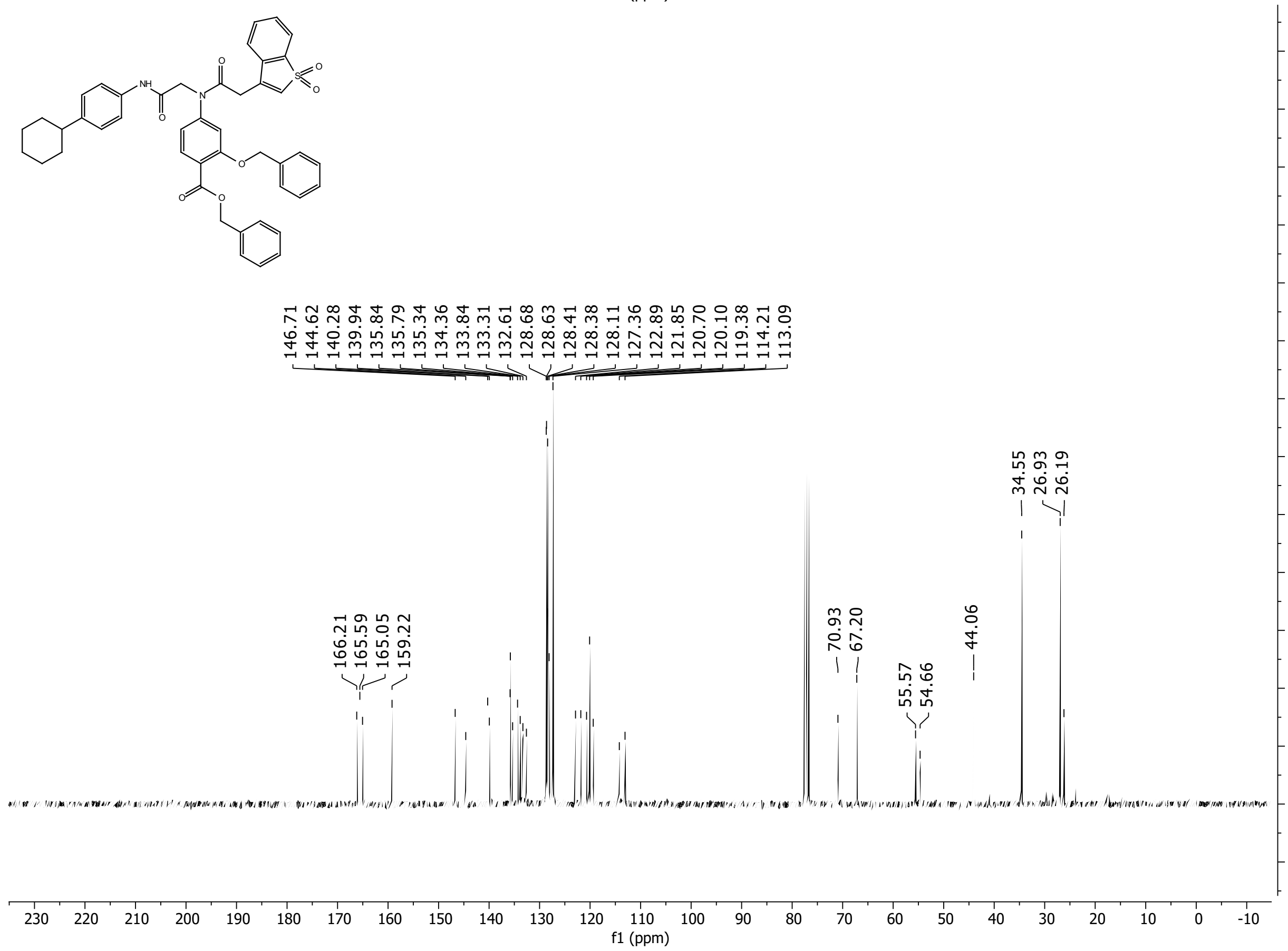

2v-P

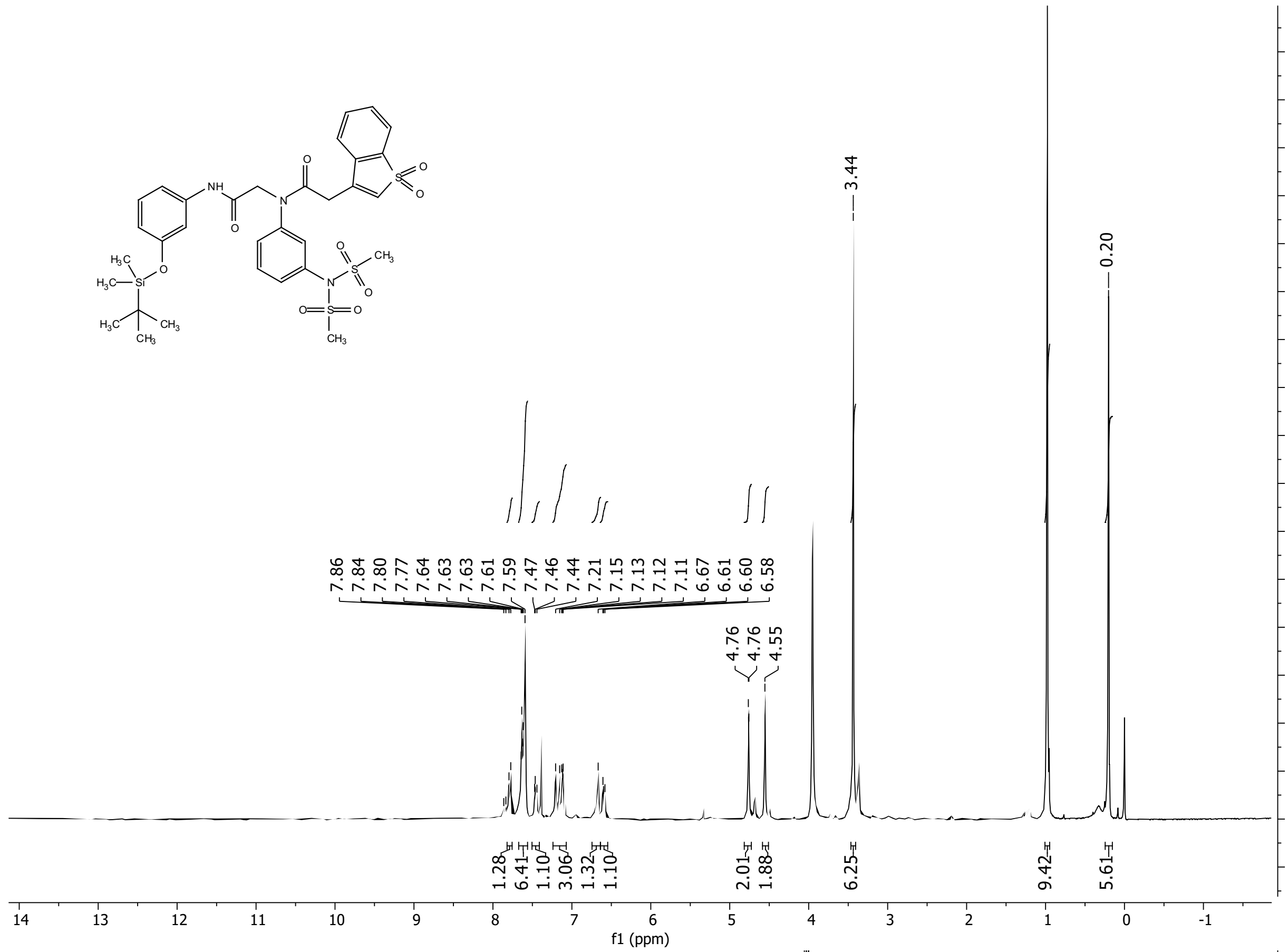

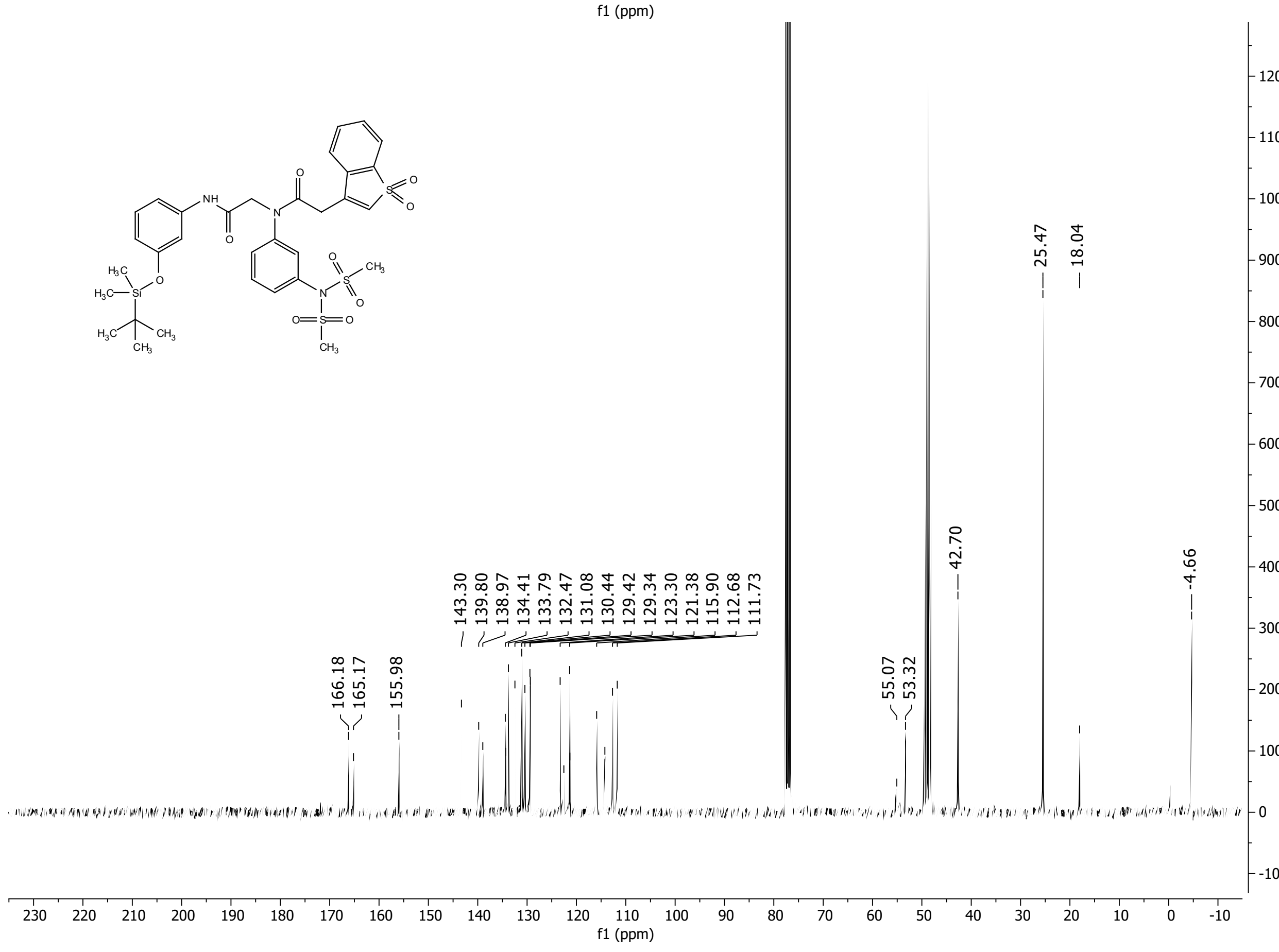

20

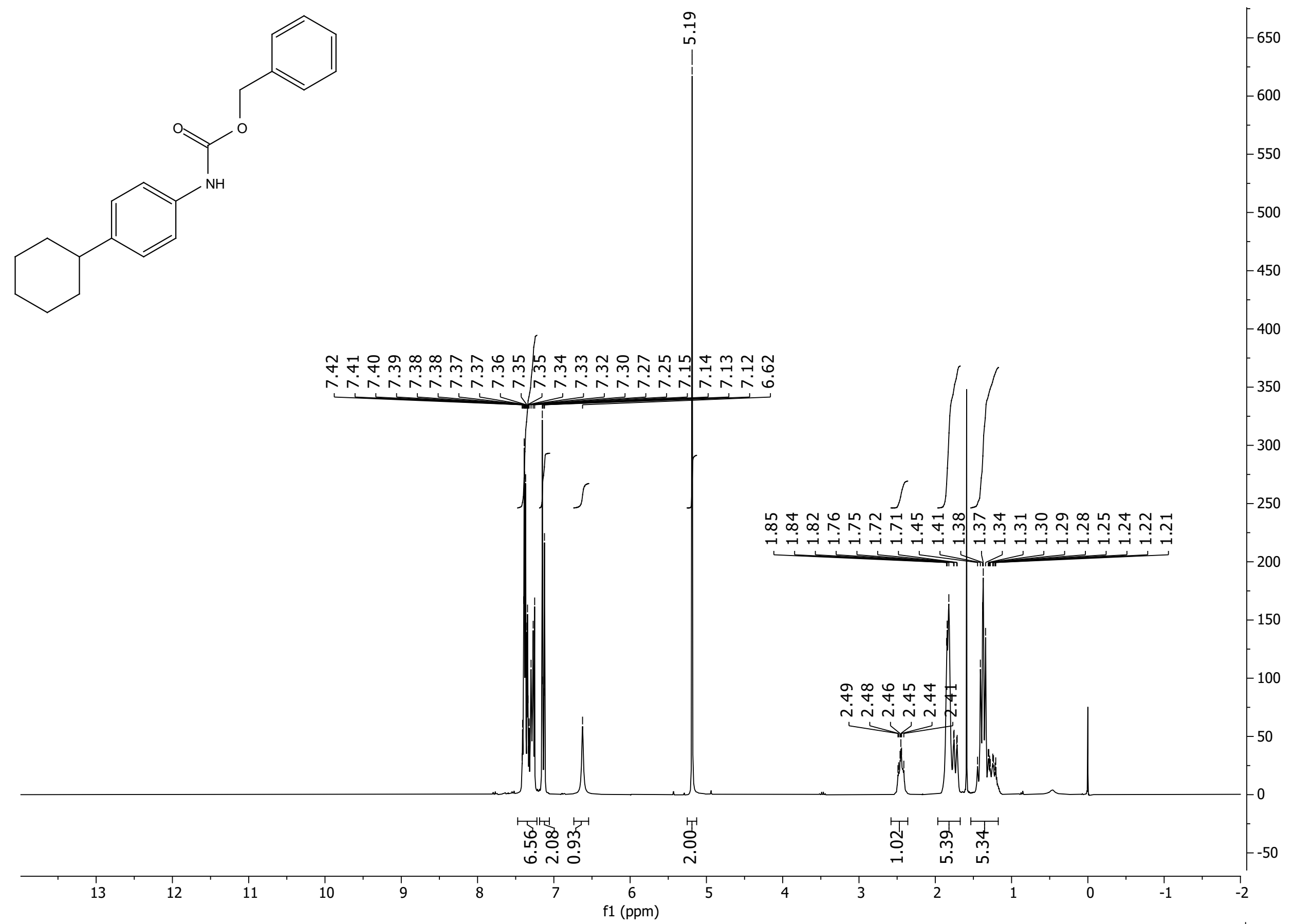

NH
O
O
5.19
7.42
7.41
7.40
7.39
7.38
7.38
7.37
7.37
7.36
7.35
7.35
7.34
7.33
7.32
7.30
7.27
7.25
7.15
7.14
7.13
7.12
6.62
1.85
1.84
1.82
1.76
1.75
1.72
1.71
1.45
1.41
1.38
1.37
1.34
1.31
1.30
1.29
1.28
1.25
1.24
1.22
1.21
2.49
2.48
2.46
2.45
2.44
2.41
6.56
2.08
0.93
2.00
1.02
5.39
5.34
f1 (ppm)

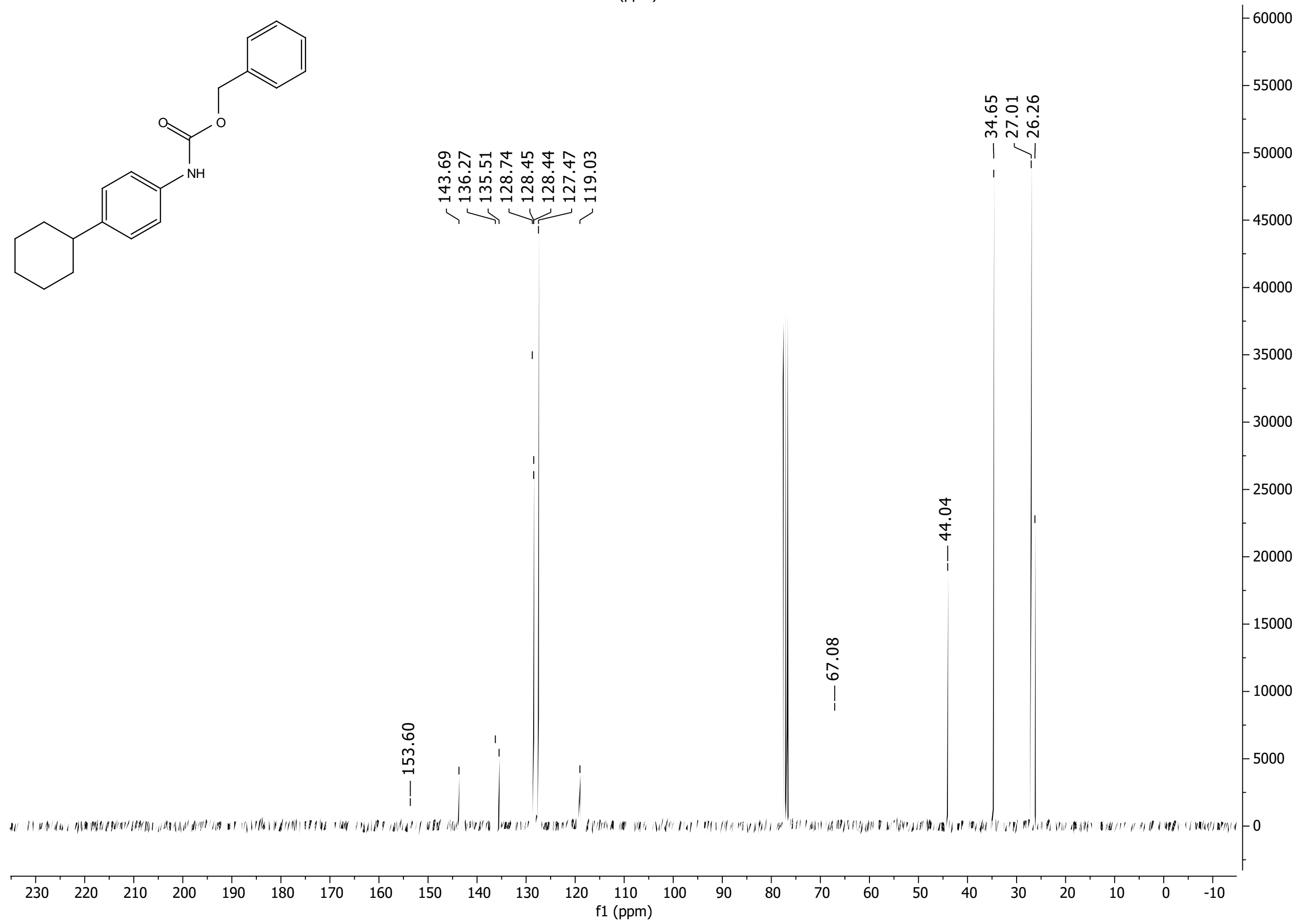

NH
O
O
153.60
143.69
136.27
135.51
128.74
128.45
128.44
127.47
119.03
67.08
44.04
34.65
27.01
26.26
f1 (ppm)

21

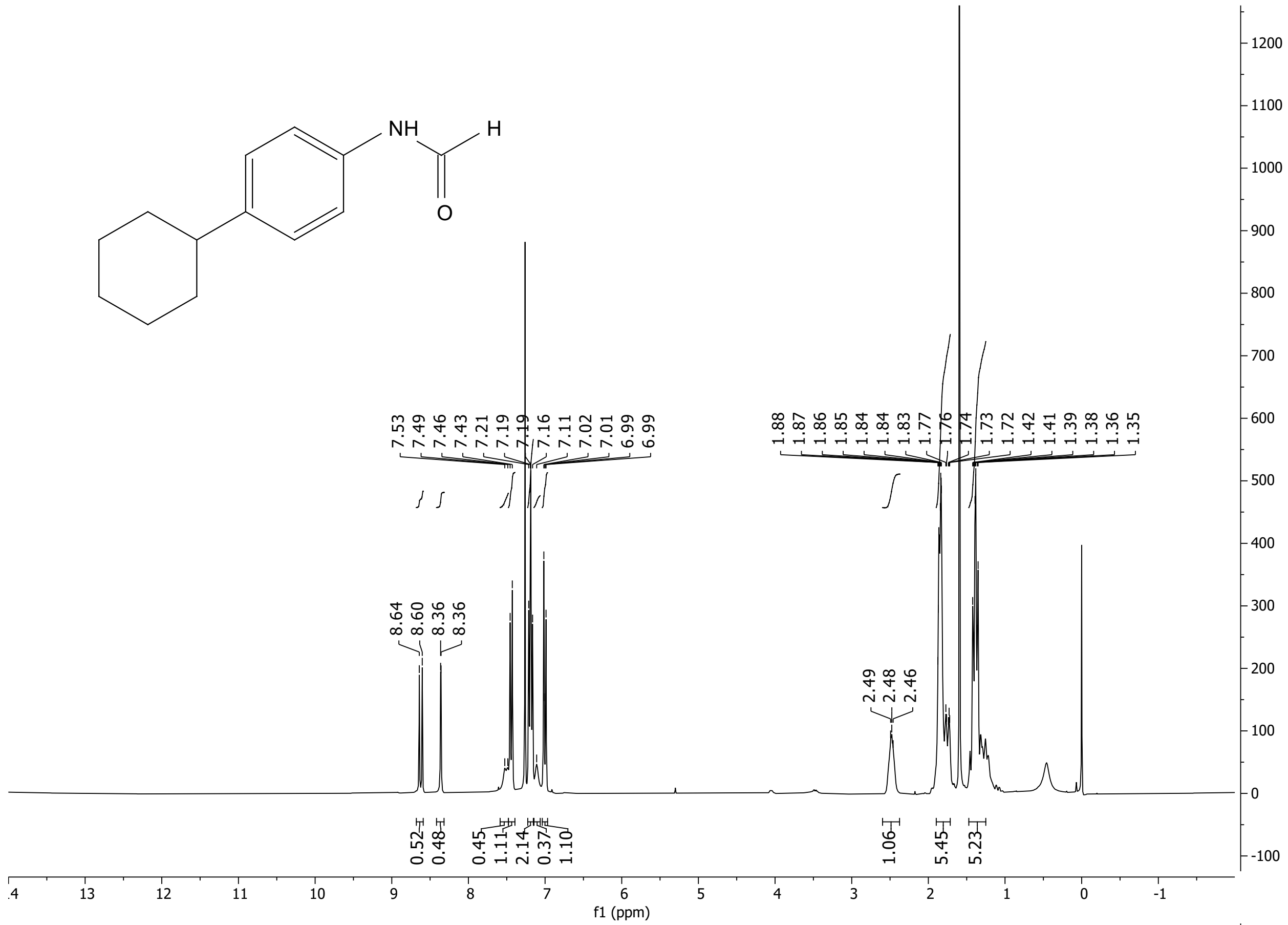
NH
H
O
7.53
7.49
7.46
7.43
7.21
7.19
7.19
7.16
7.11
7.02
7.01
6.99
6.99
1.88
1.87
1.86
1.85
1.84
1.84
1.83
1.77
1.76
1.74
1.73
1.72
1.42
1.41
1.39
1.38
1.36
1.35
8.64
8.60
8.36
8.36
2.49
2.48
2.46
0.52
0.48
0.45
1.11
2.14
0.37
1.10
1.06
5.45
5.23
f1 (ppm)

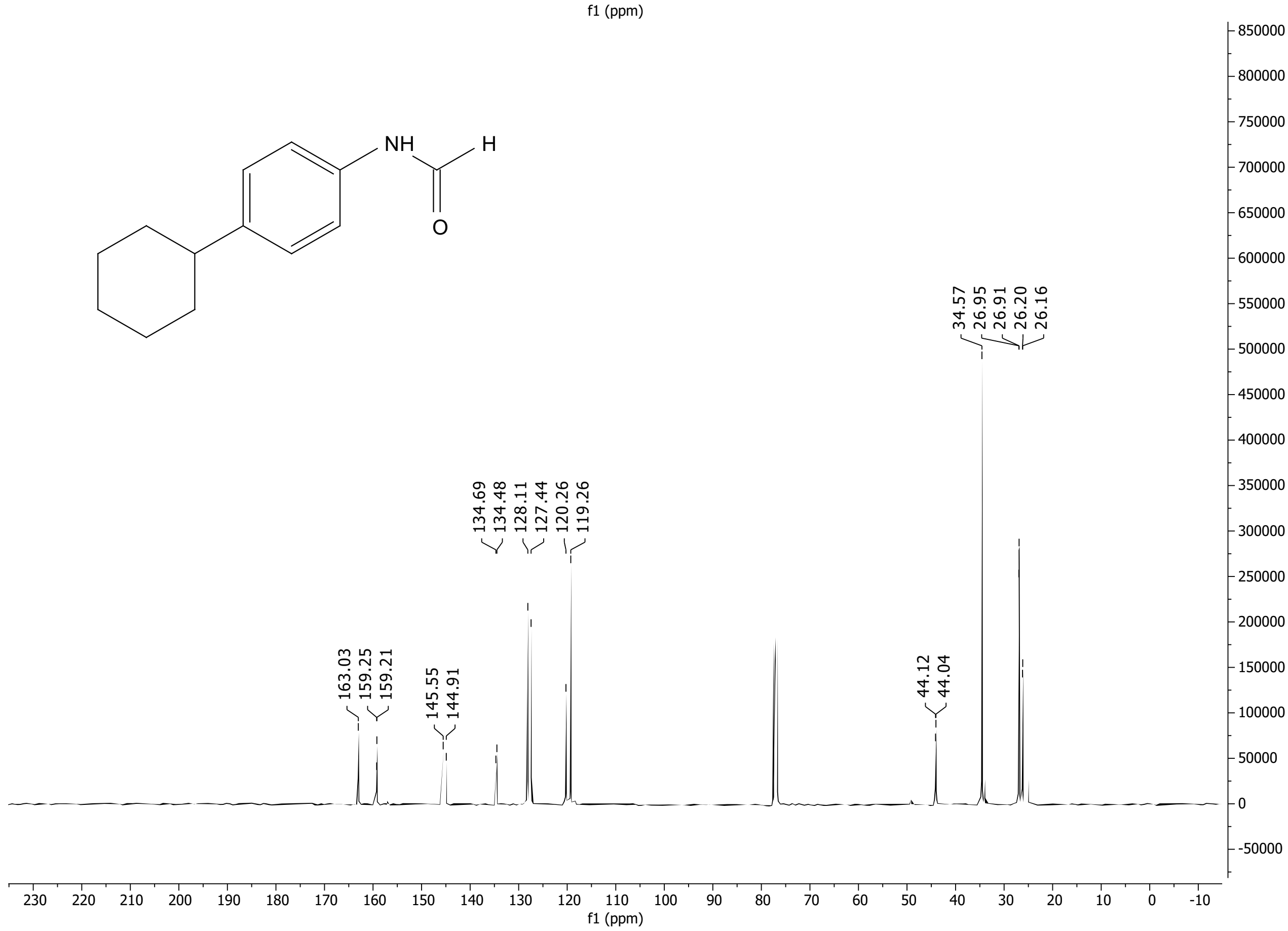
NH
H
O
34.57
26.95
26.91
26.20
26.16
134.69
134.48
128.11
127.44
120.26
119.26
163.03
159.25
159.21
145.55
144.91
44.12
44.04
f1 (ppm)

24

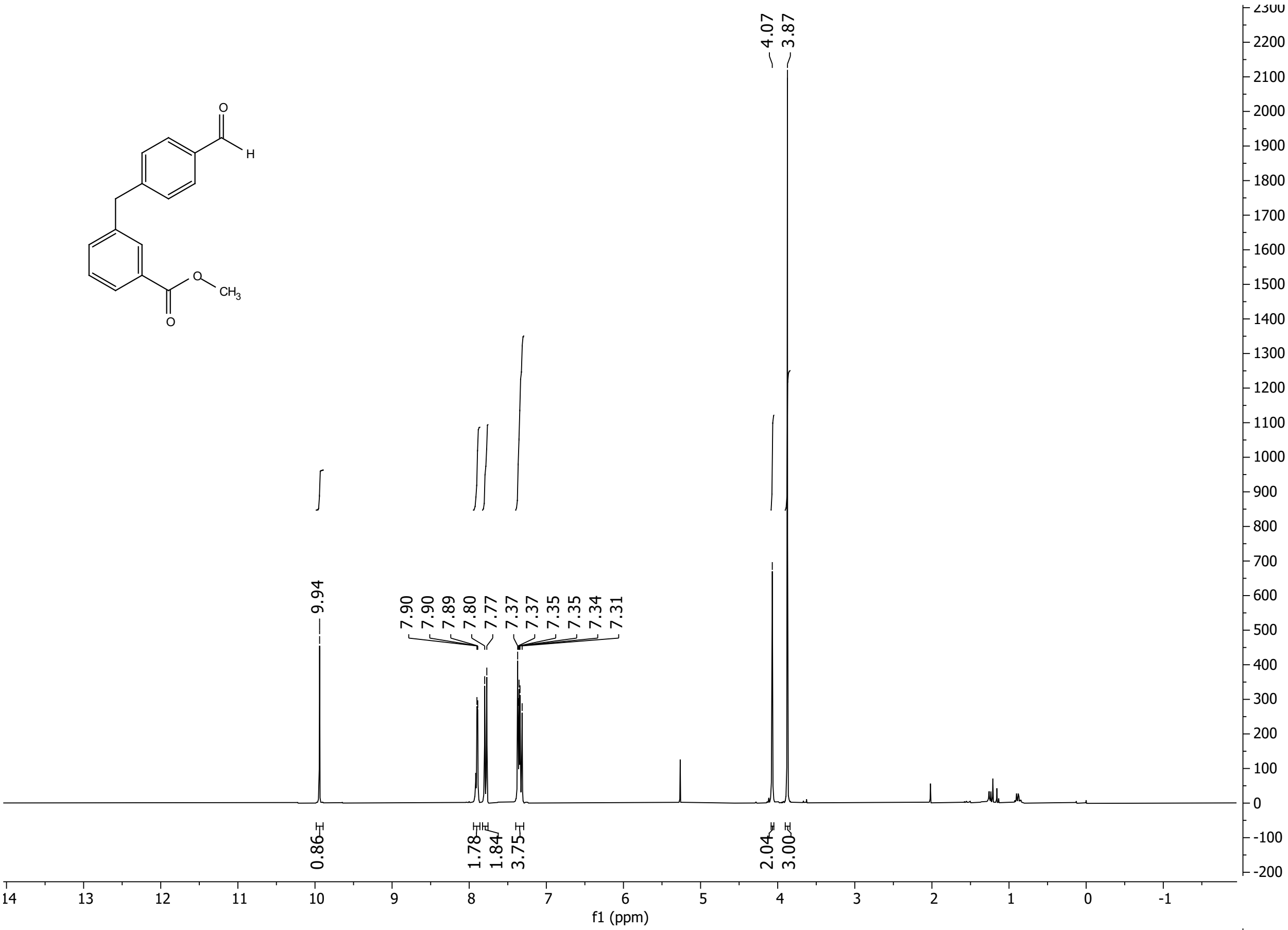
O
H
O
CH3
O
9.94
7.90
7.90
7.89
7.80
7.77
7.37
7.37
7.35
7.35
7.34
7.31
4.07
3.87
0.86
1.78
1.84
3.75
2.04
3.00
f1 (ppm)

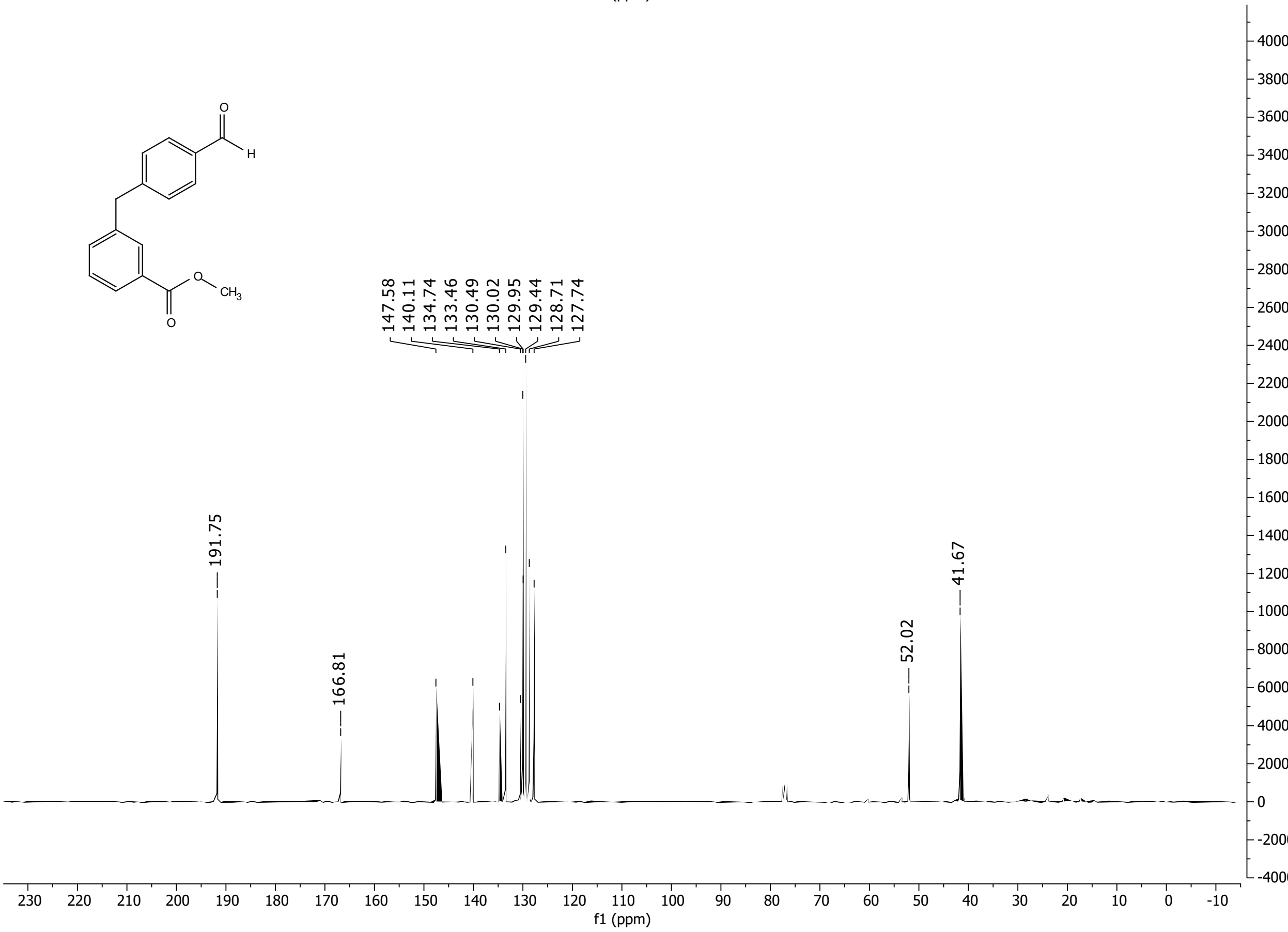
O
H
O
CH3
O
191.75
166.81
147.58
140.11
134.74
133.46
130.49
130.02
129.95
129.44
128.71
127.74
52.02
41.67
f1 (ppm)

26

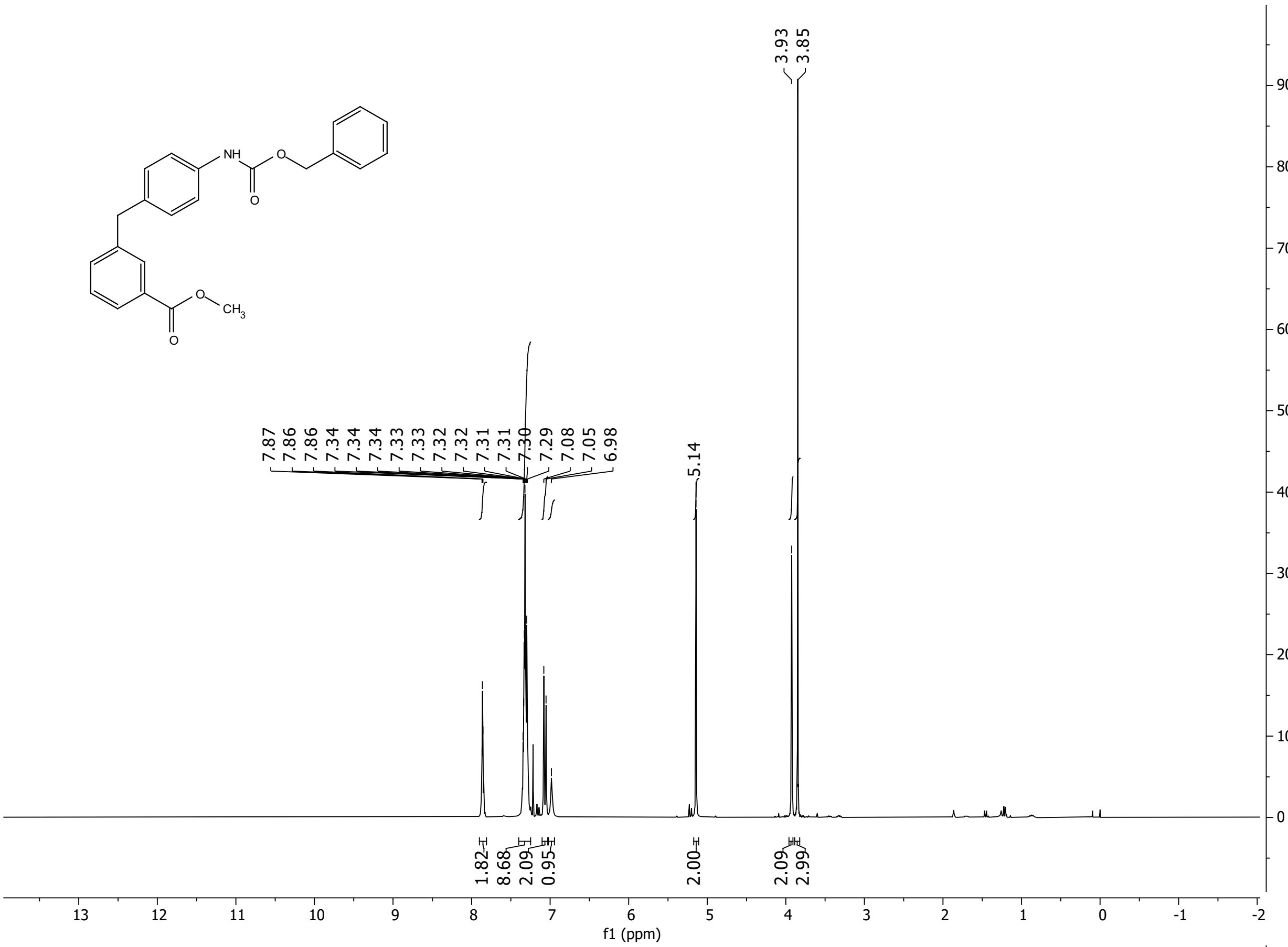

NH
O
O
O
CH3
7.87
7.86
7.86
7.34
7.34
7.34
7.33
7.33
7.32
7.32
7.31
7.31
7.30
7.29
7.08
7.05
6.98
5.14
3.93
3.85
1.82
8.68
2.09
0.95
2.00
2.09
2.99
f1 (ppm)

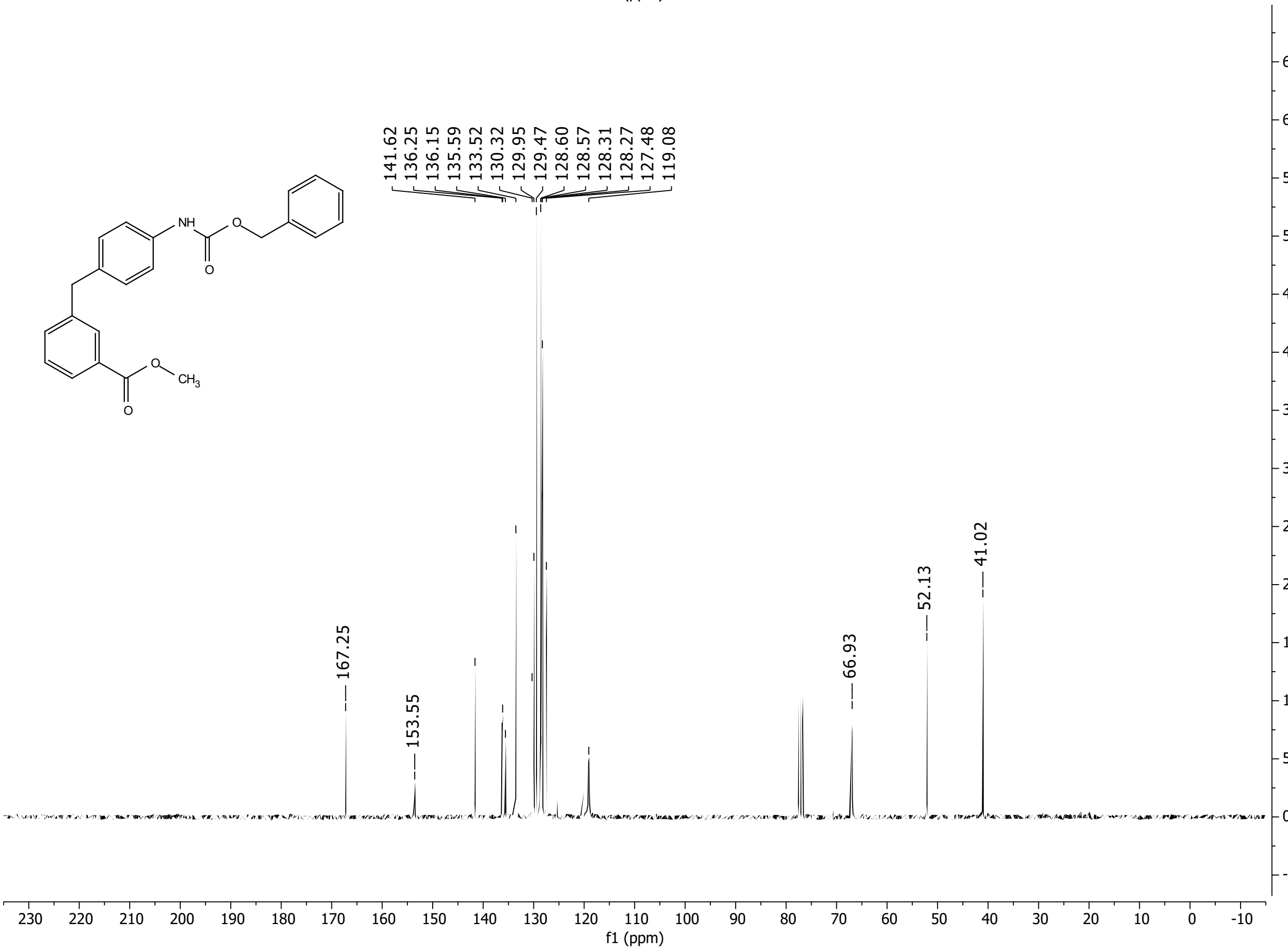

NH
O
O
O
CH3
141.62
136.25
136.15
135.59
133.52
130.32
129.95
129.47
128.60
128.57
128.31
128.27
127.48
119.08
167.25
153.55
66.93
52.13
41.02
f1 (ppm)

30

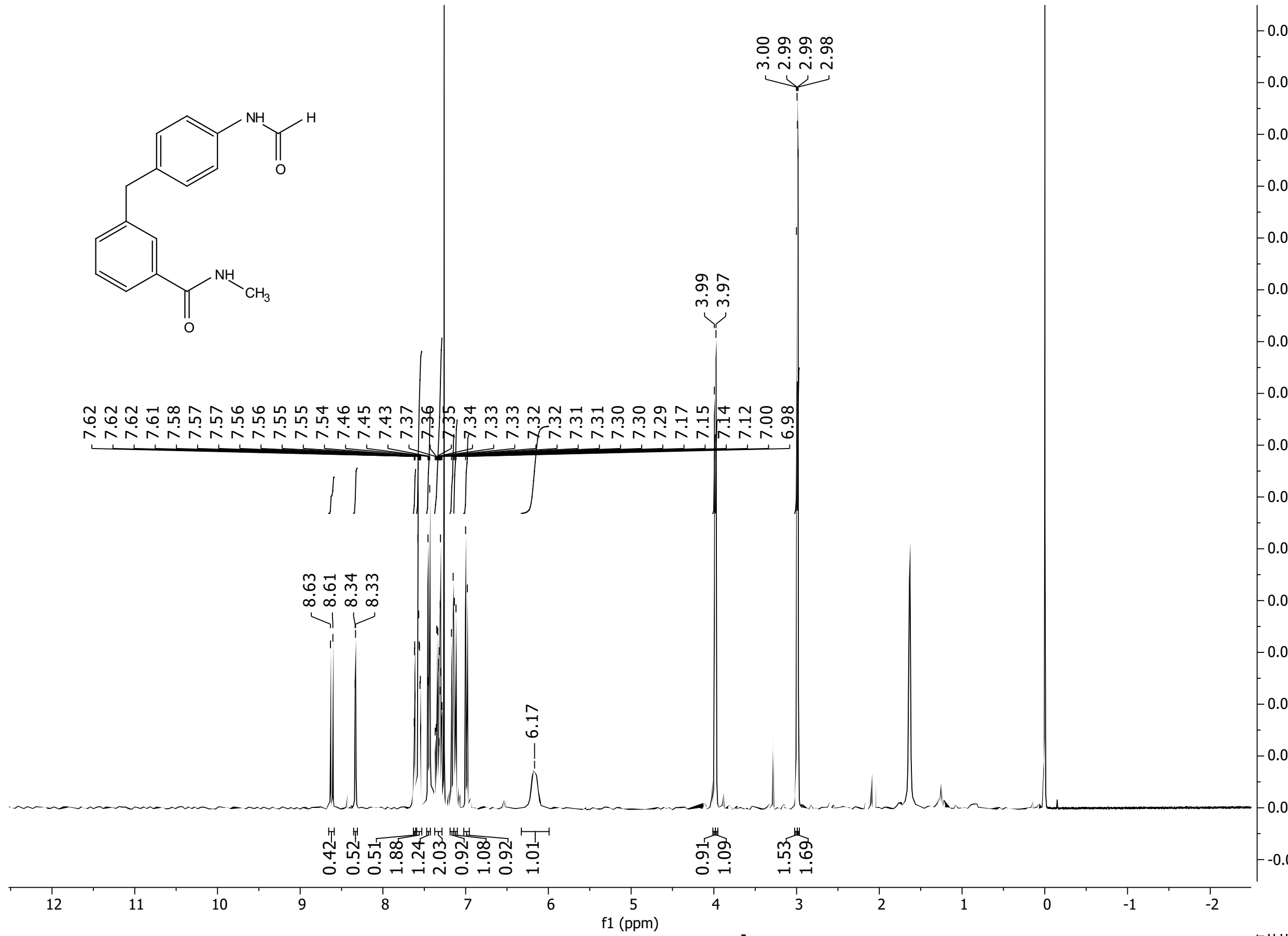

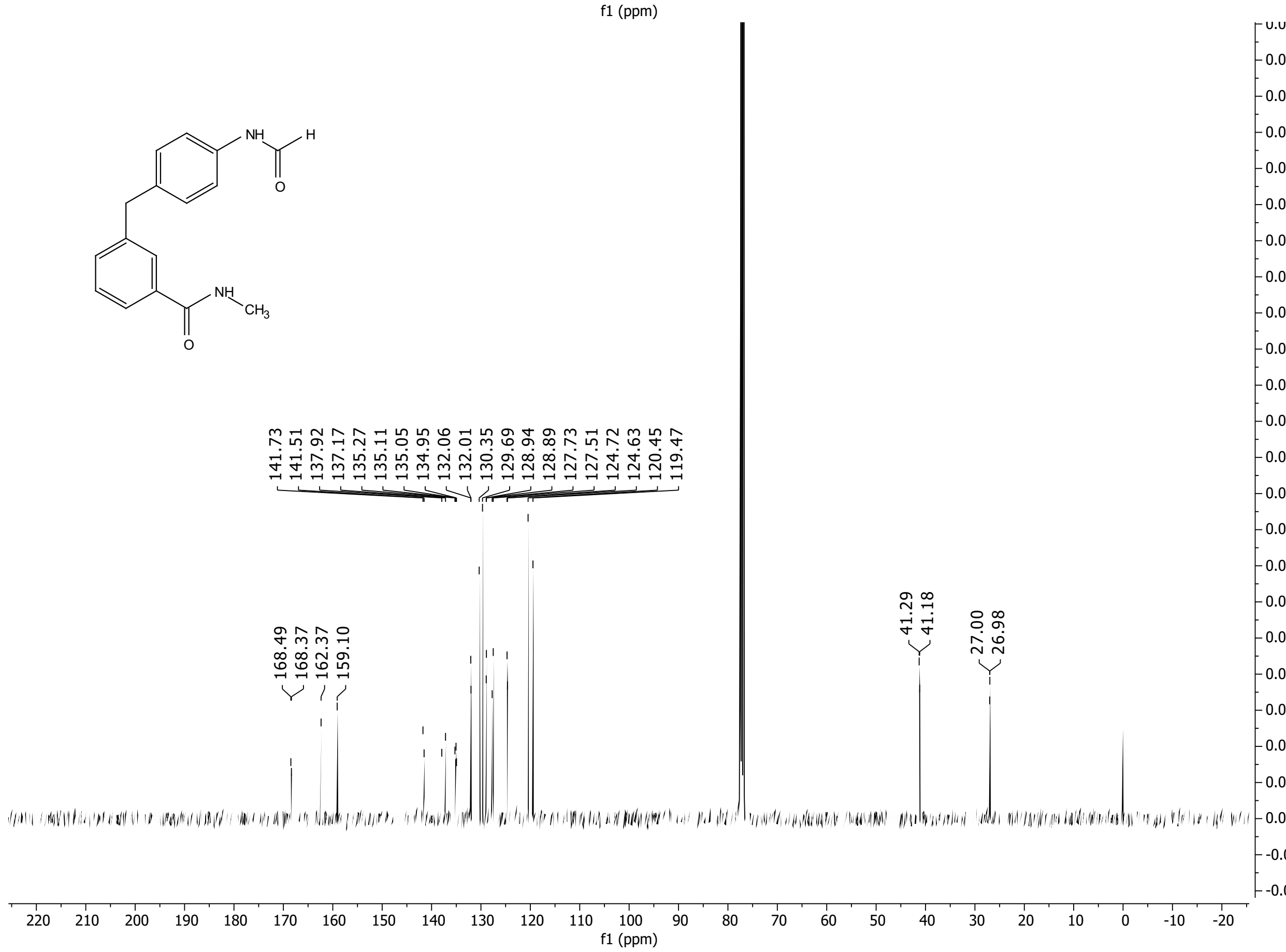

31

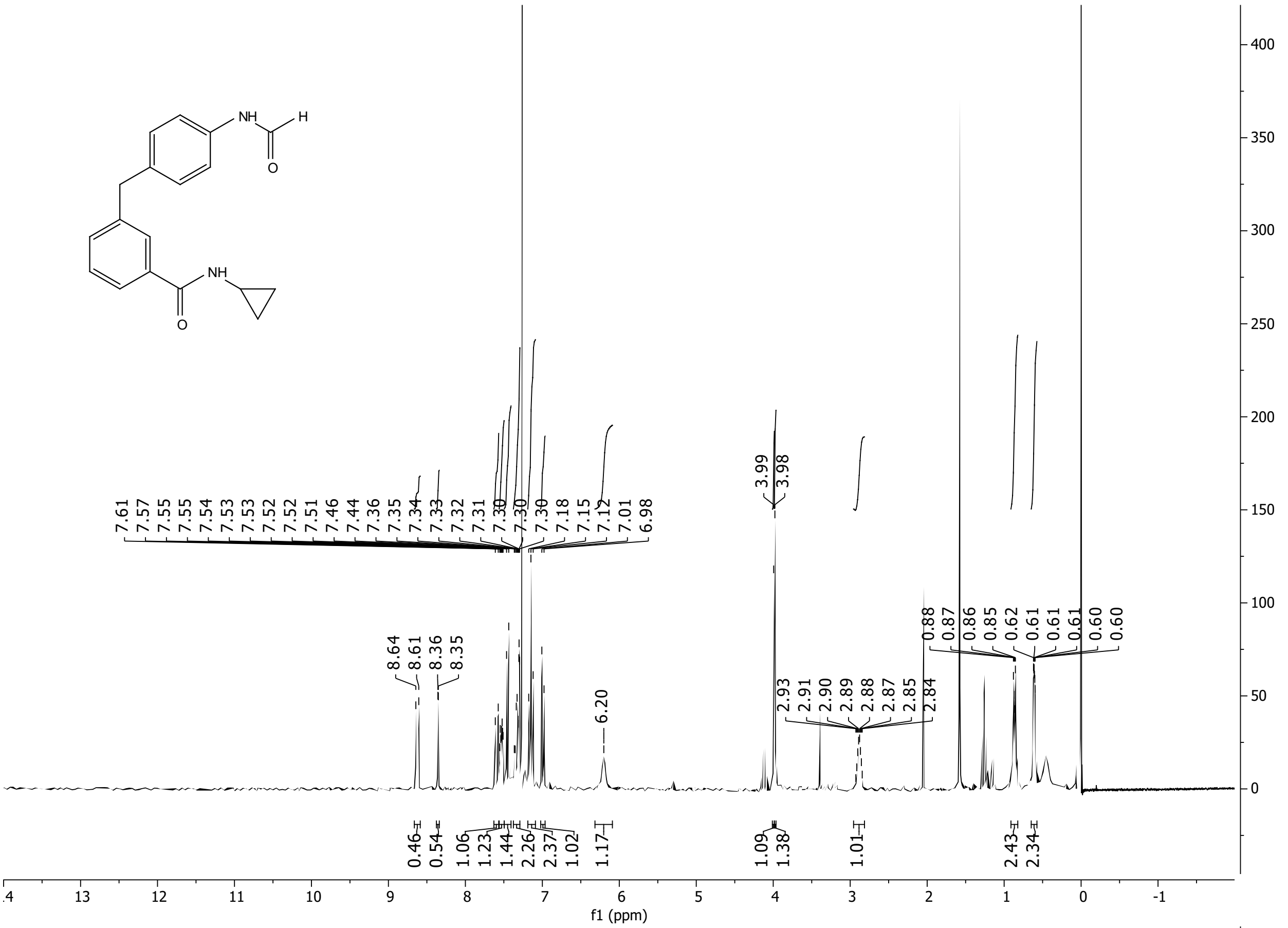
NH
H
O
NH
O
7.61 7.57 7.55 7.55 7.54 7.53 7.53 7.52 7.52 7.51 7.46 7.44 7.36 7.35 7.34 7.33 7.32 7.31 7.30 7.30 7.30 7.18 7.15 7.12 7.01 6.98
3.99 3.98
8.64 8.61 8.36 8.35
6.20
2.93 2.91 2.90 2.89 2.88 2.87 2.85 2.84
0.88 0.87 0.86 0.85 0.62 0.61 0.61 0.61 0.60 0.60
0.46 0.54 1.06 1.23 1.44 2.26 2.37 1.02 1.17 1.09 1.38 1.01 2.43 2.34
f1 (ppm)

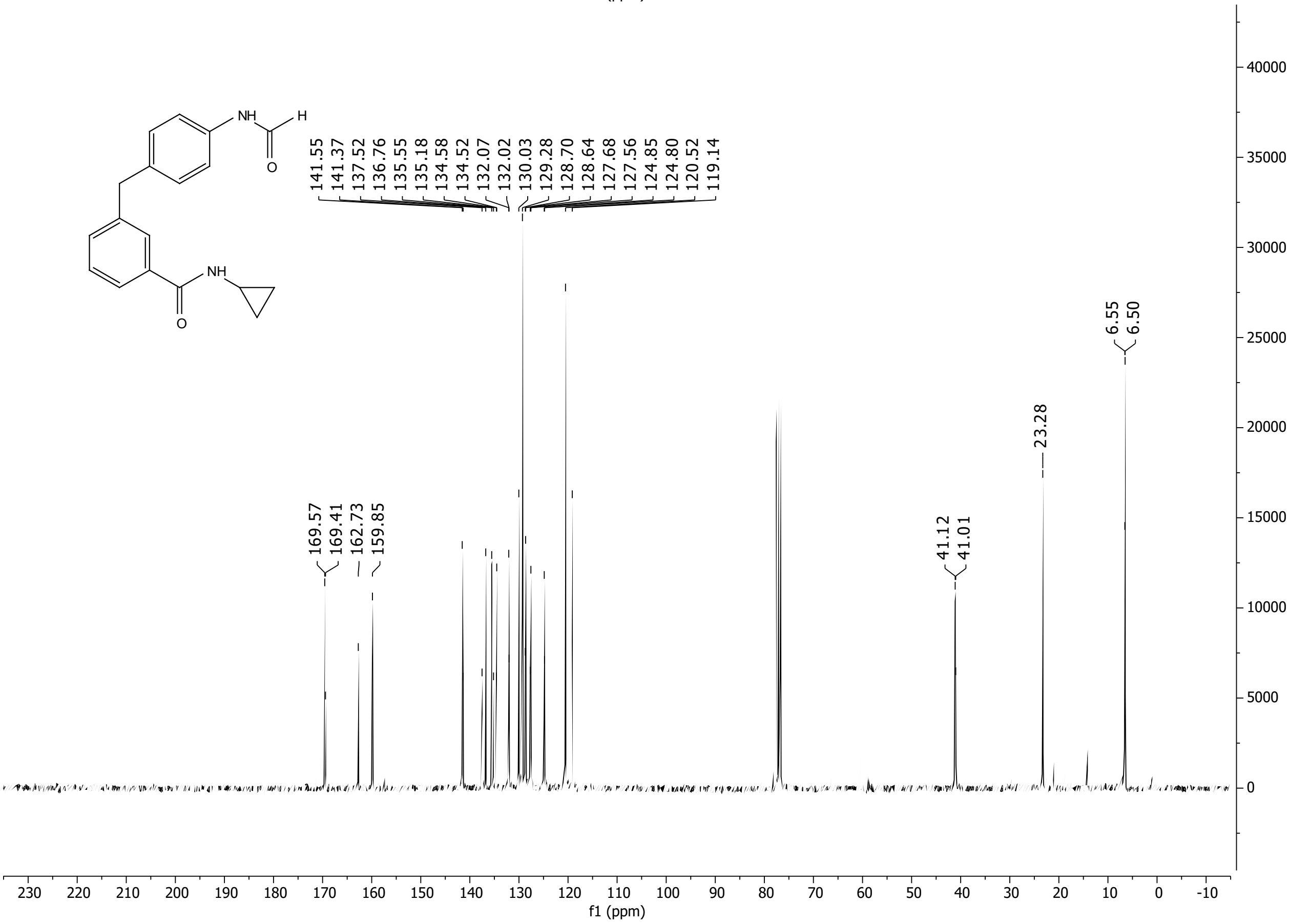
NH
H
O
NH
O
141.55 141.37 137.52 136.76 135.55 135.18 134.58 134.52 132.07 132.02 130.03 129.28 128.70 128.64 127.68 127.56 124.85 124.80 120.52 119.14
169.57 169.41 162.73 159.85
41.12 41.01
23.28
6.55 6.50
f1 (ppm)

33

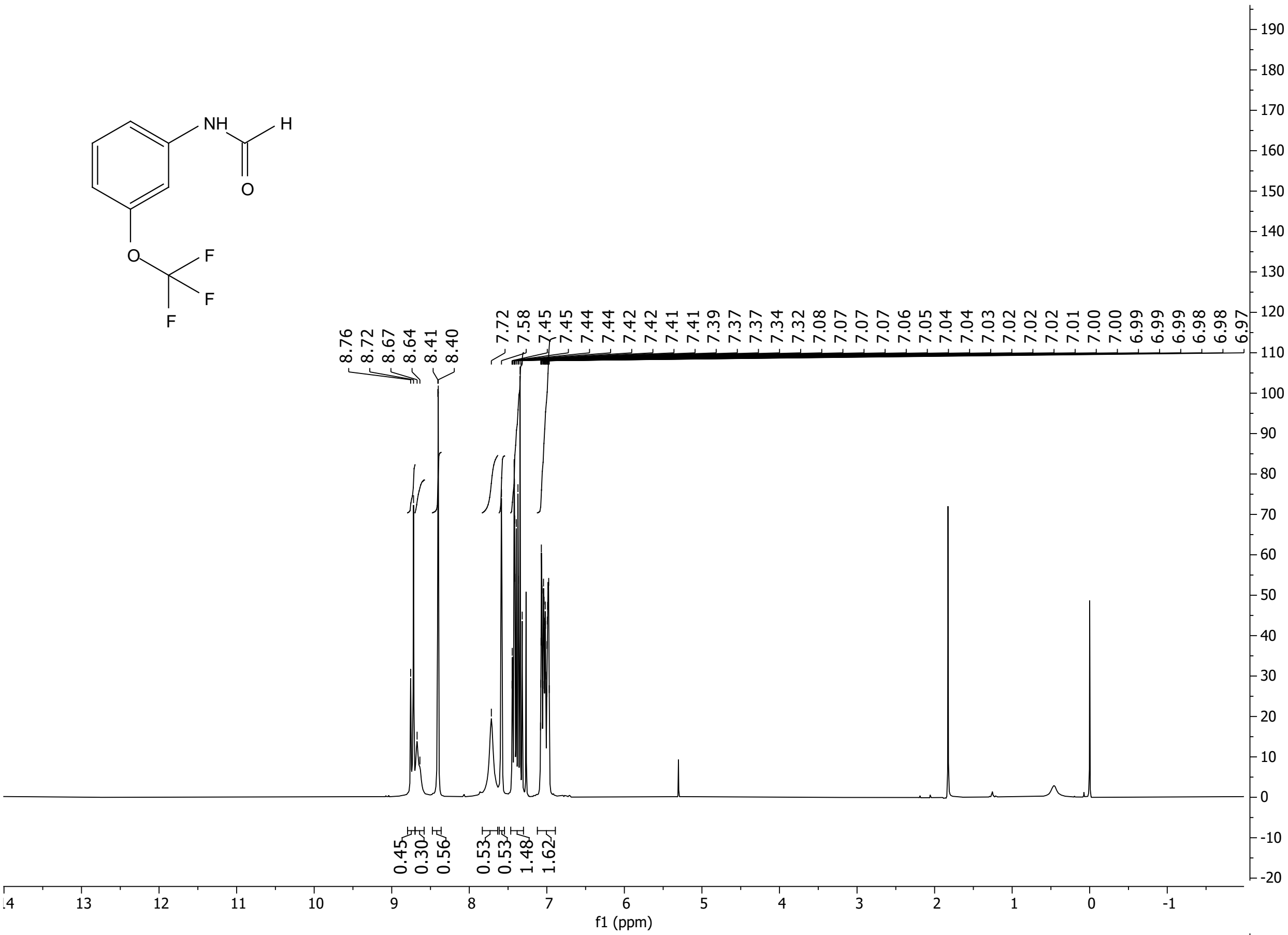

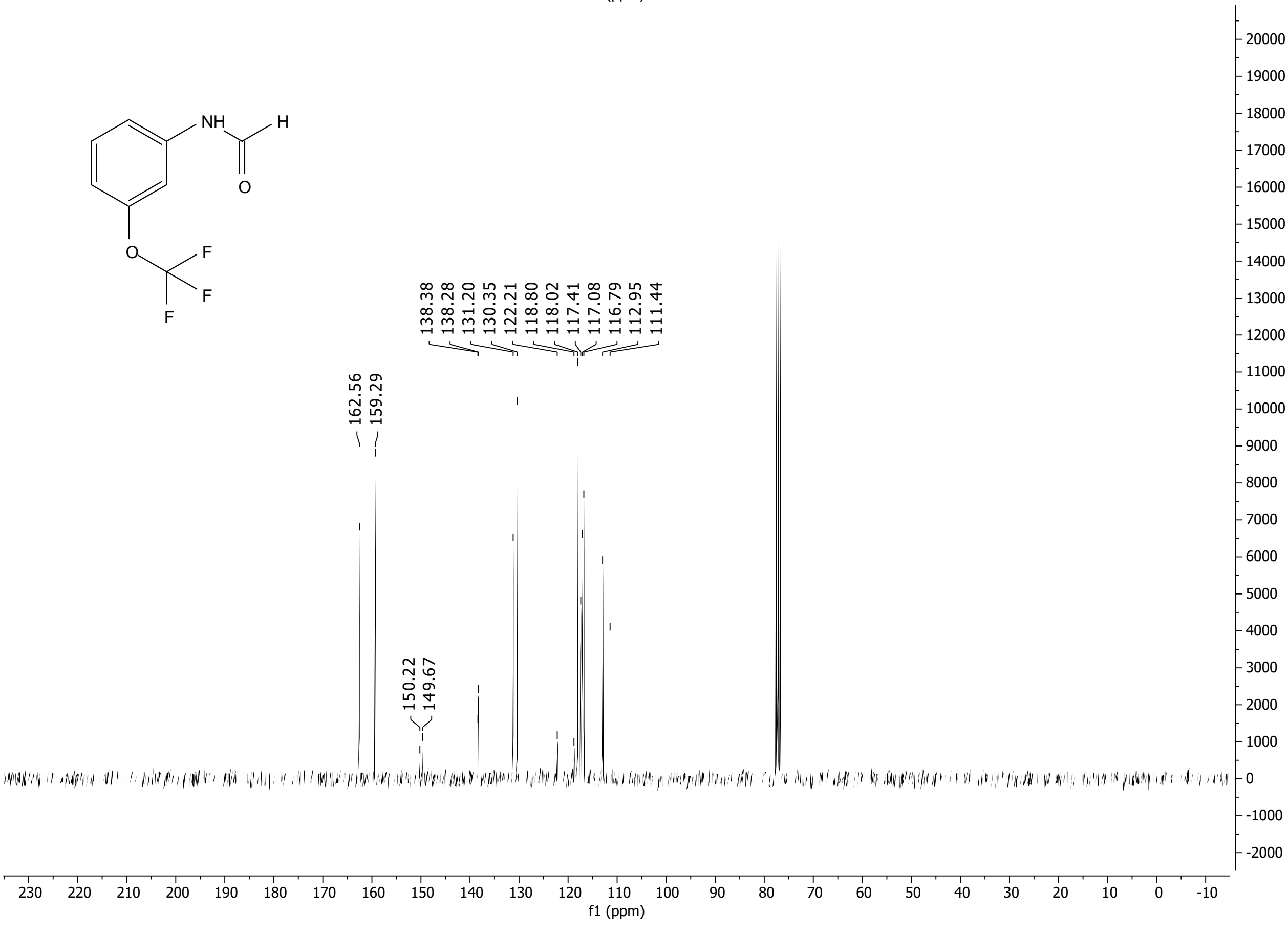

36

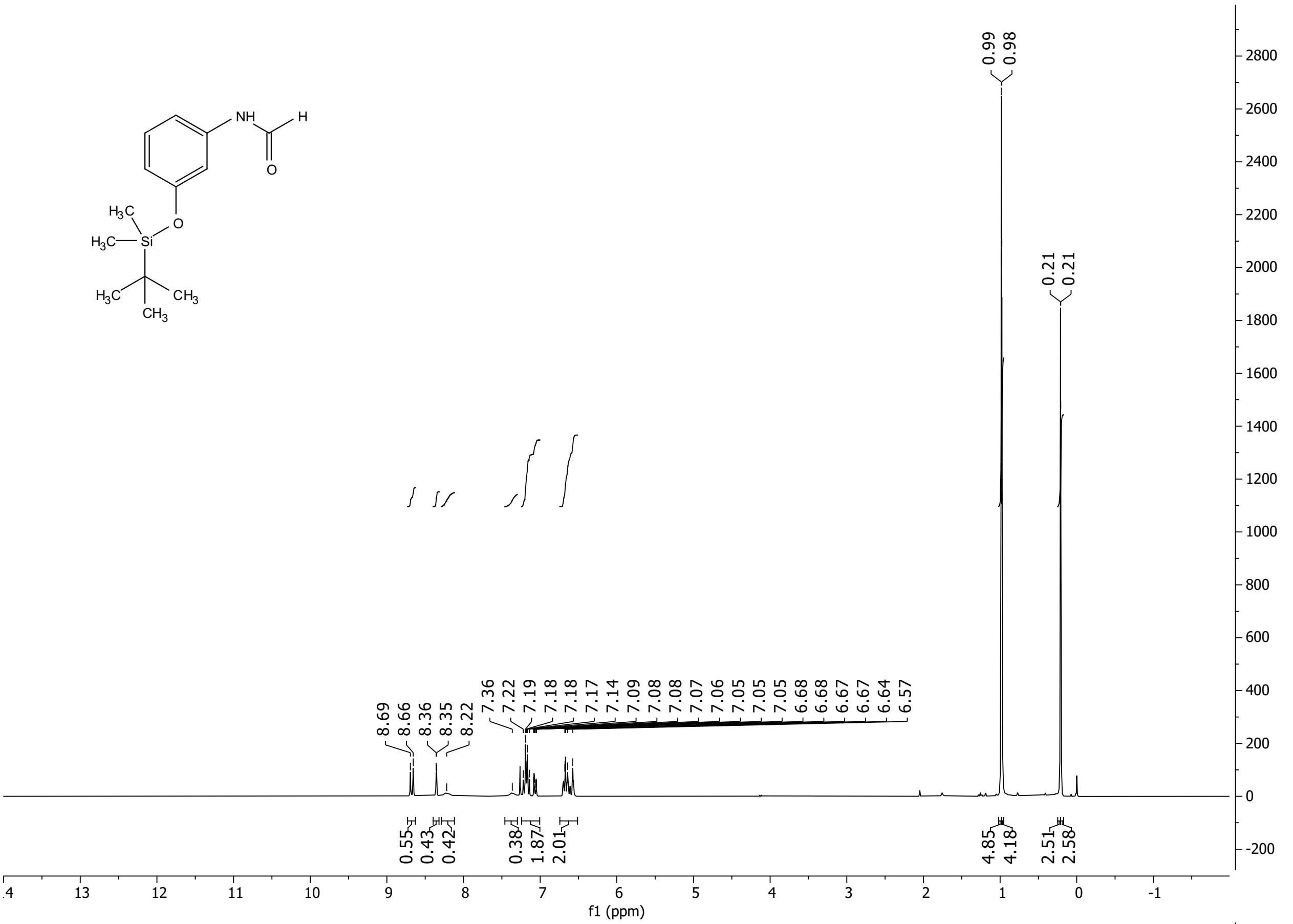
NH
H
O
O
H_3C
H_3C
Si
H_3C
CH_3
CH_3
0.99
0.98
0.21
0.21
8.69
8.66
8.36
8.35
8.22
7.36
7.22
7.19
7.18
7.18
7.17
7.14
7.09
7.08
7.07
7.06
7.05
7.05
6.68
6.68
6.67
6.67
6.64
6.57
0.55
0.43
0.42
0.38
1.87
2.01
4.85
4.18
2.51
2.58
f1 (ppm)

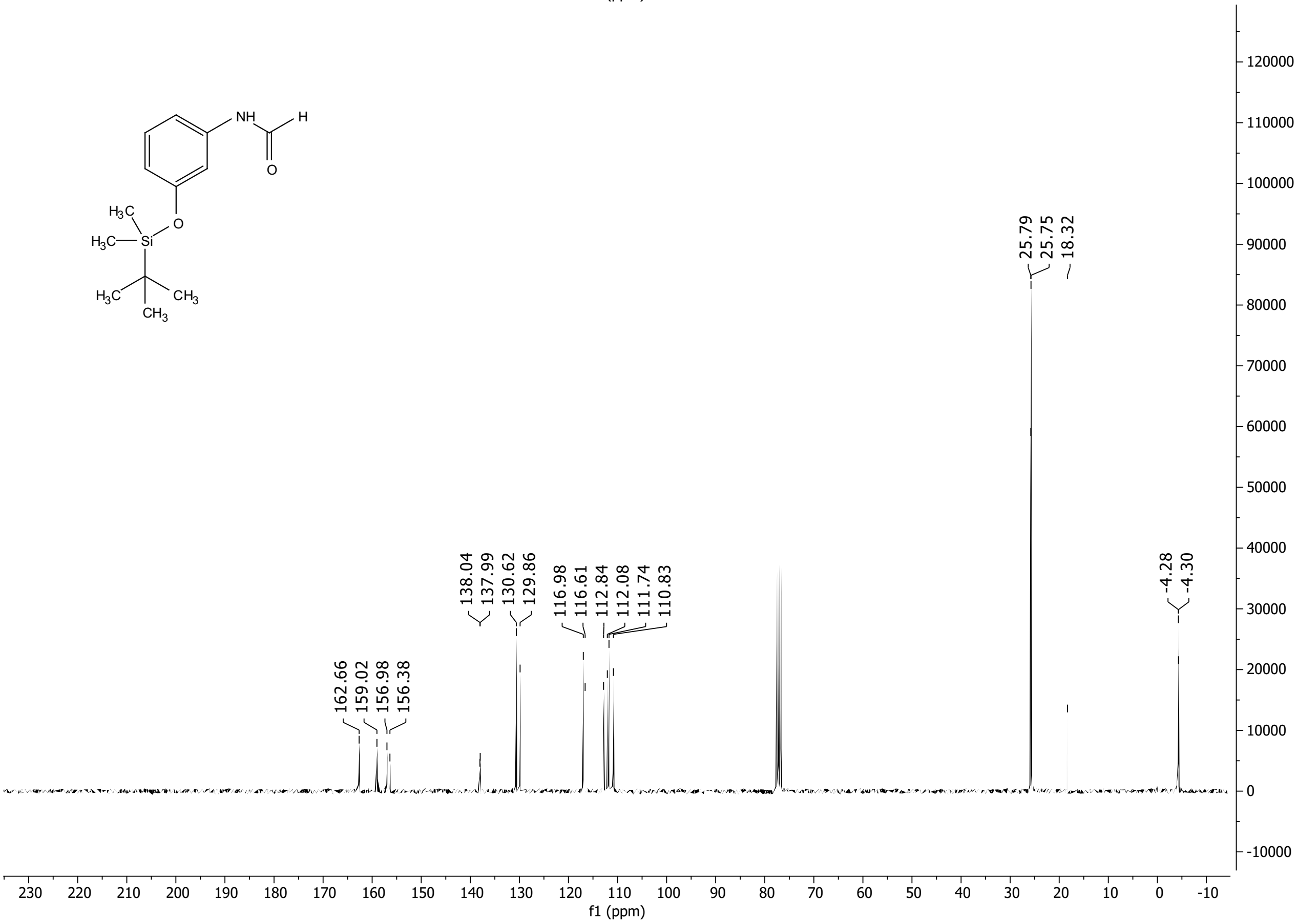
NH
H
O
O
H_3C
H_3C
Si
H_3C
CH_3
CH_3
25.79
25.75
18.32
138.04
137.99
130.62
129.86
116.98
116.61
112.84
112.08
111.74
110.83
162.66
159.02
156.98
156.38
-4.28
-4.30
f1 (ppm)

14

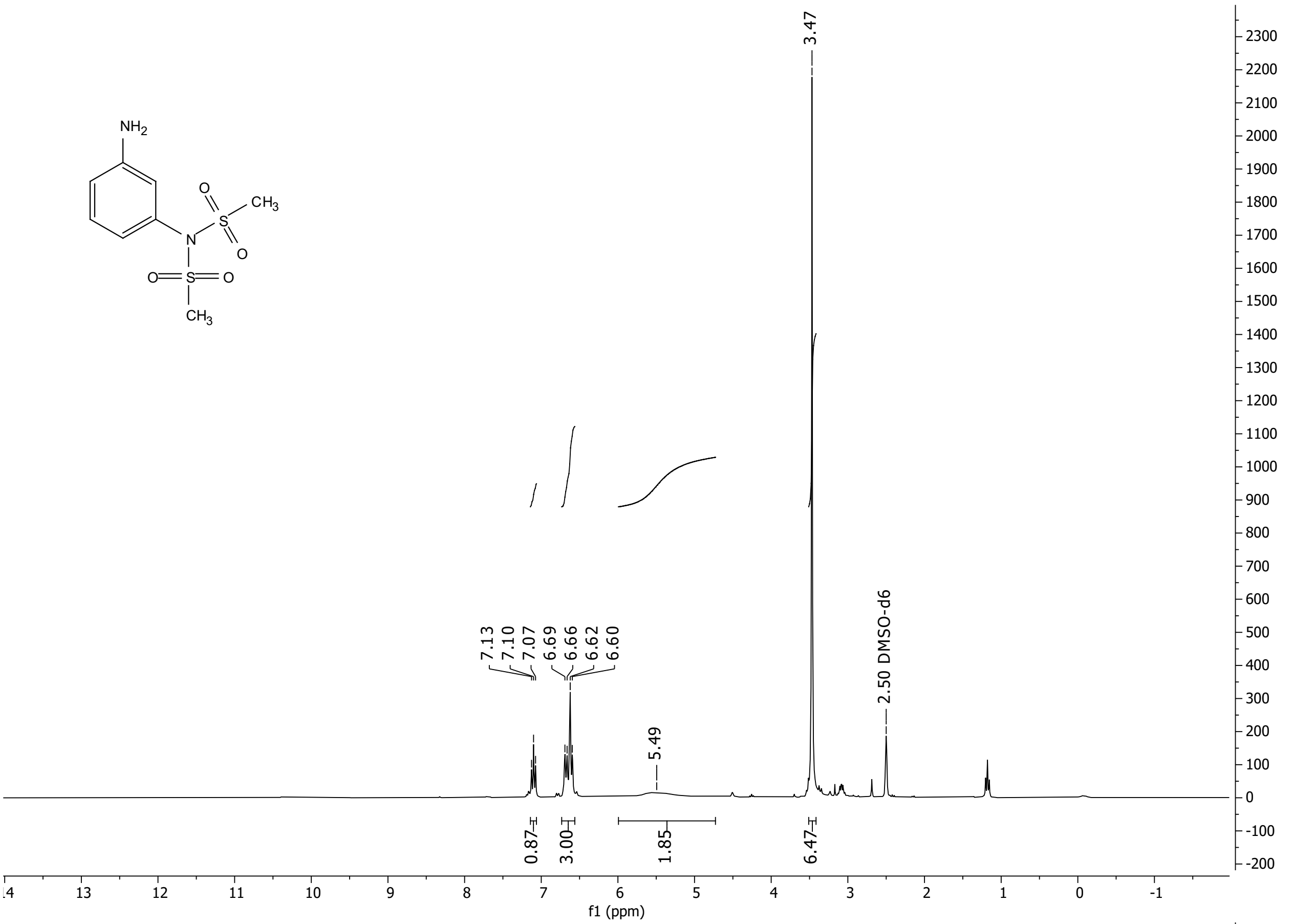
NH2
O
CH3
N
S
O
O=S=O
CH3
3.47
7.13
7.10
7.07
6.69
6.66
6.62
6.60
2.50 DMSO-d6
5.49
0.87
3.00
1.85
6.47
f1 (ppm)

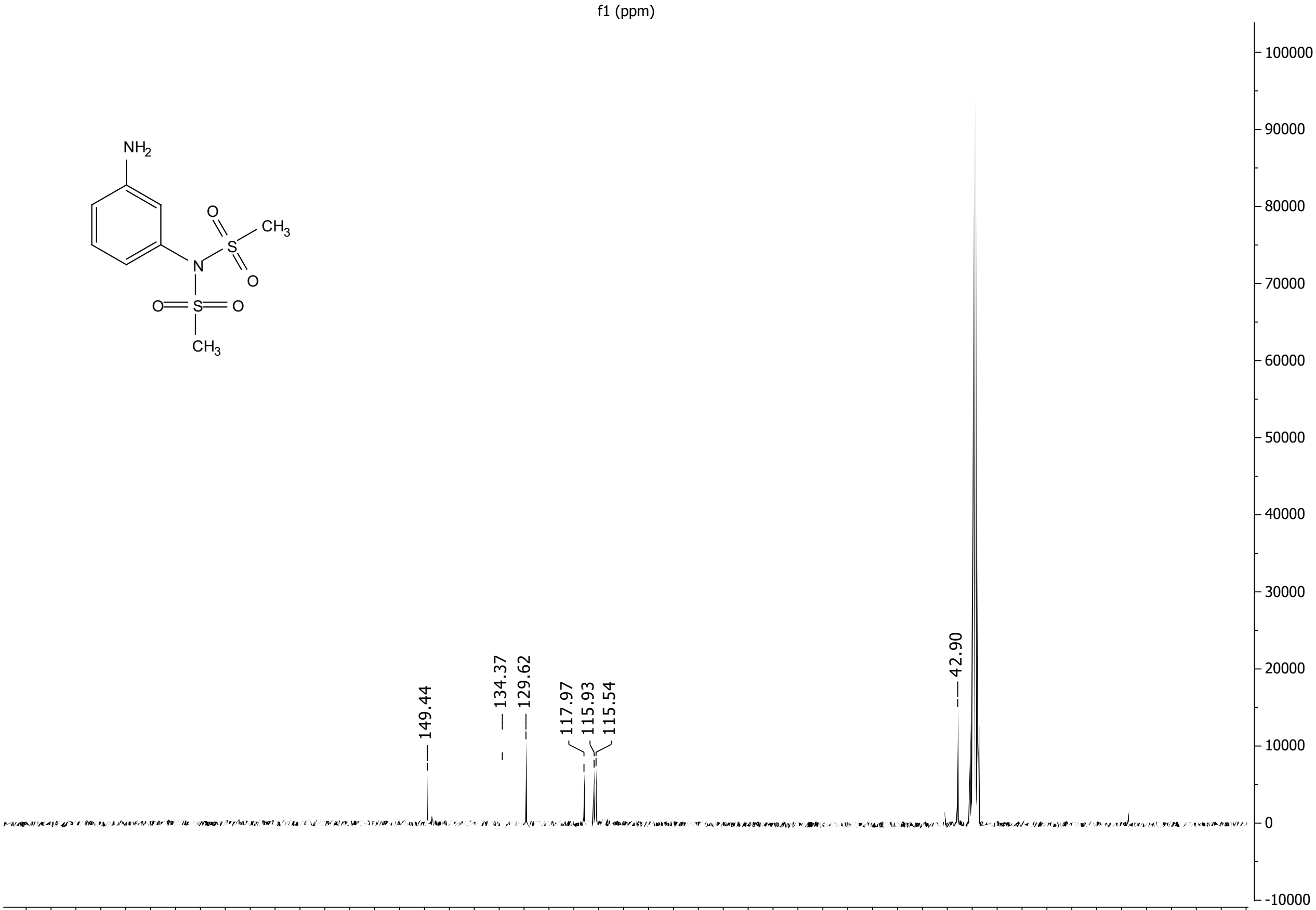
NH2
O
CH3
N
S
O
O=S=O
CH3
149.44
134.37
129.62
117.97
115.93
115.54
42.90
f1 (ppm)

41

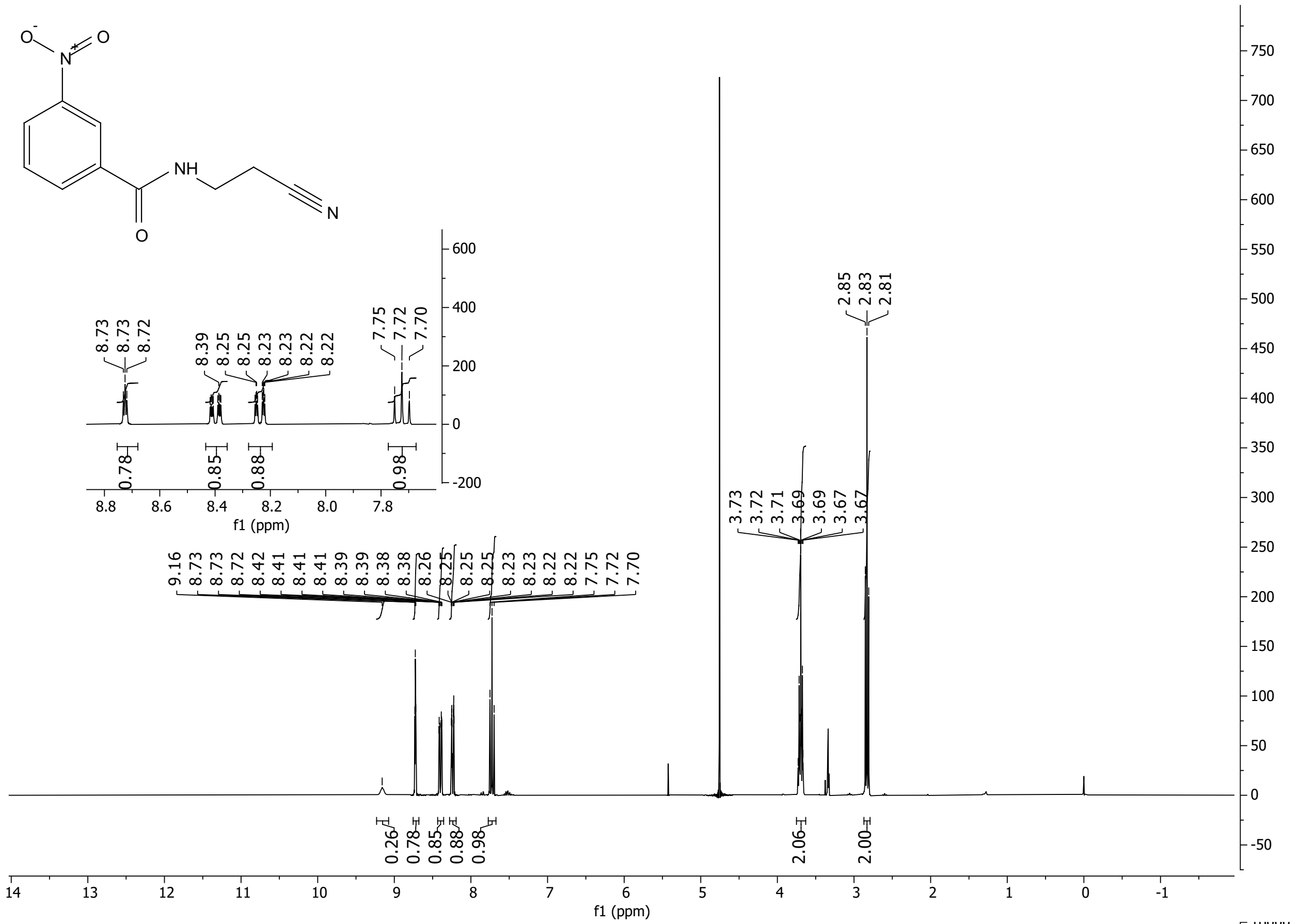

9.16 8.73 8.73 8.72 8.42 8.41 8.41 8.41 8.39 8.39 8.38 8.38 8.26 8.25 8.25 8.25 8.23 8.23 8.22 8.22 7.75 7.72 7.70
3.73 3.72 3.71 3.69 3.69 3.67 3.67
2.85 2.83 2.81
0.26 0.78 0.85 0.88 0.98 2.06 2.00
f1 (ppm)

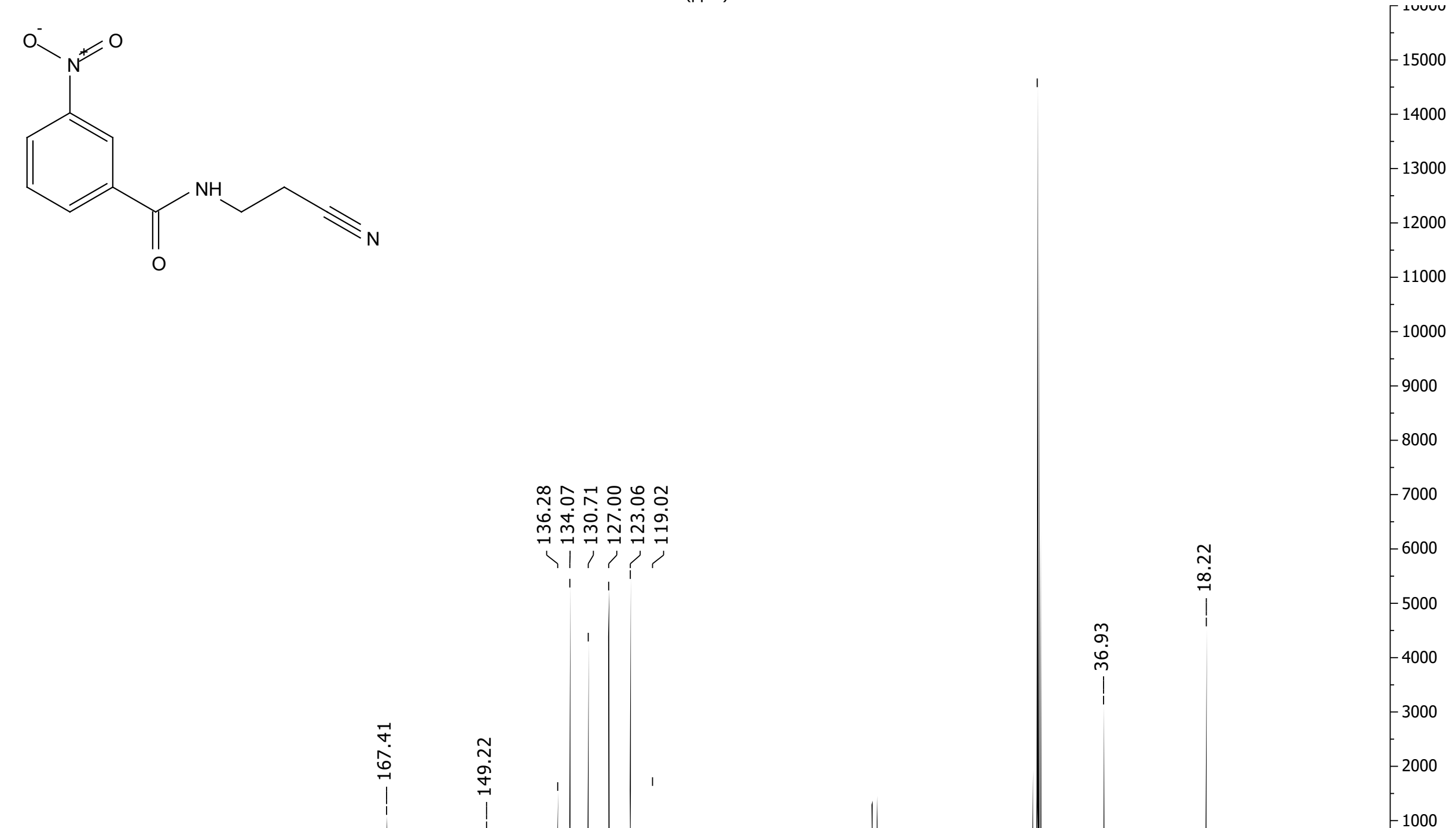

167.41
149.22
136.28 134.07 130.71 127.00 123.06 119.02
36.93
18.22
f1 (ppm)

42

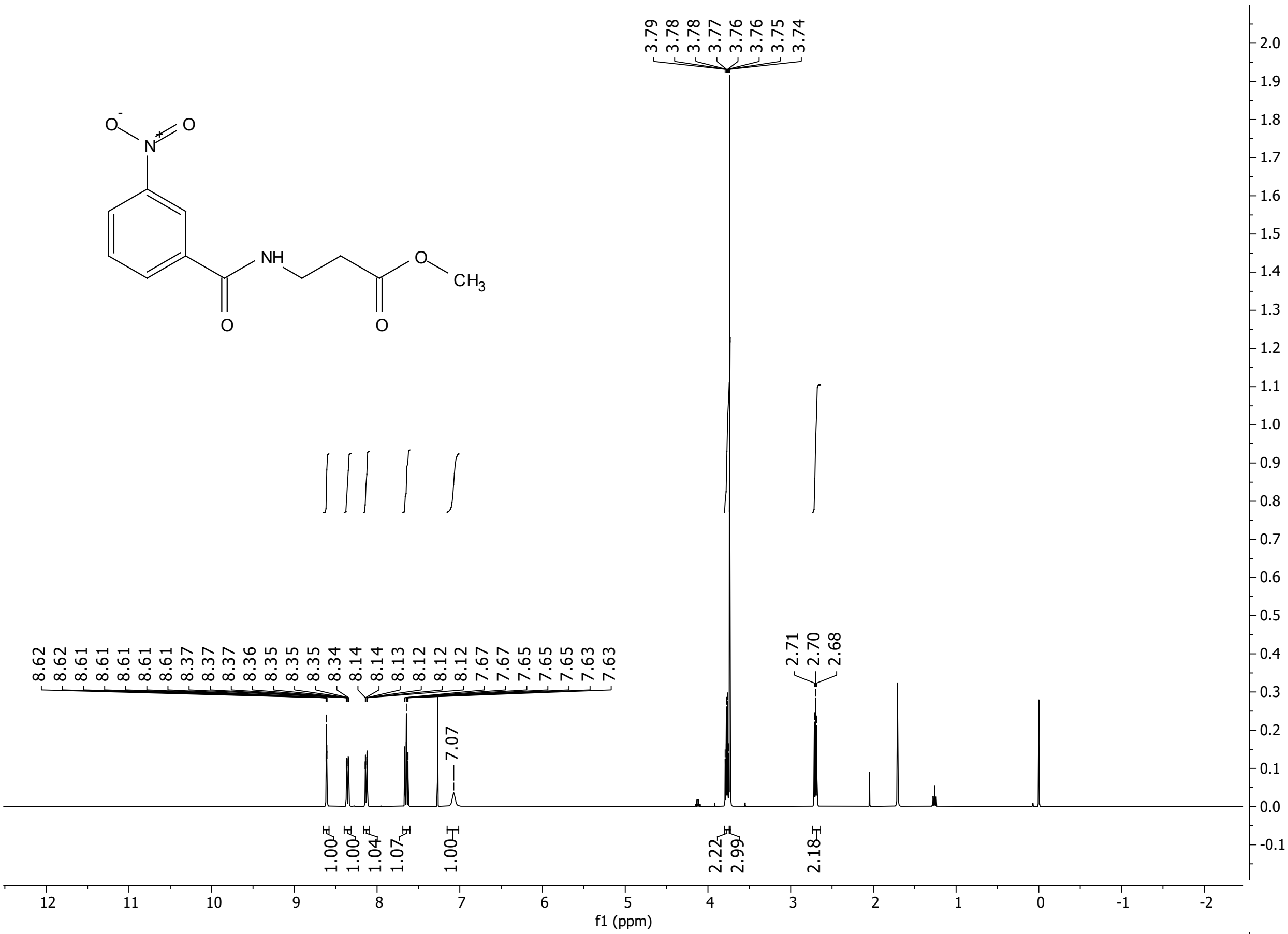

8.62 8.62 8.61 8.61 8.61 8.61 8.61 8.37 8.37 8.36 8.35 8.35 8.34 8.14 8.14 8.13 8.12 8.12 8.12 7.67 7.67 7.65 7.65 7.65 7.63 7.63
7.07
3.79 3.78 3.78 3.77 3.76 3.76 3.75 3.74
2.71 2.70 2.68
1.00 1.00 1.04 1.07 1.00 2.22 2.99 2.18
NH
O
CH3
f1 (ppm)

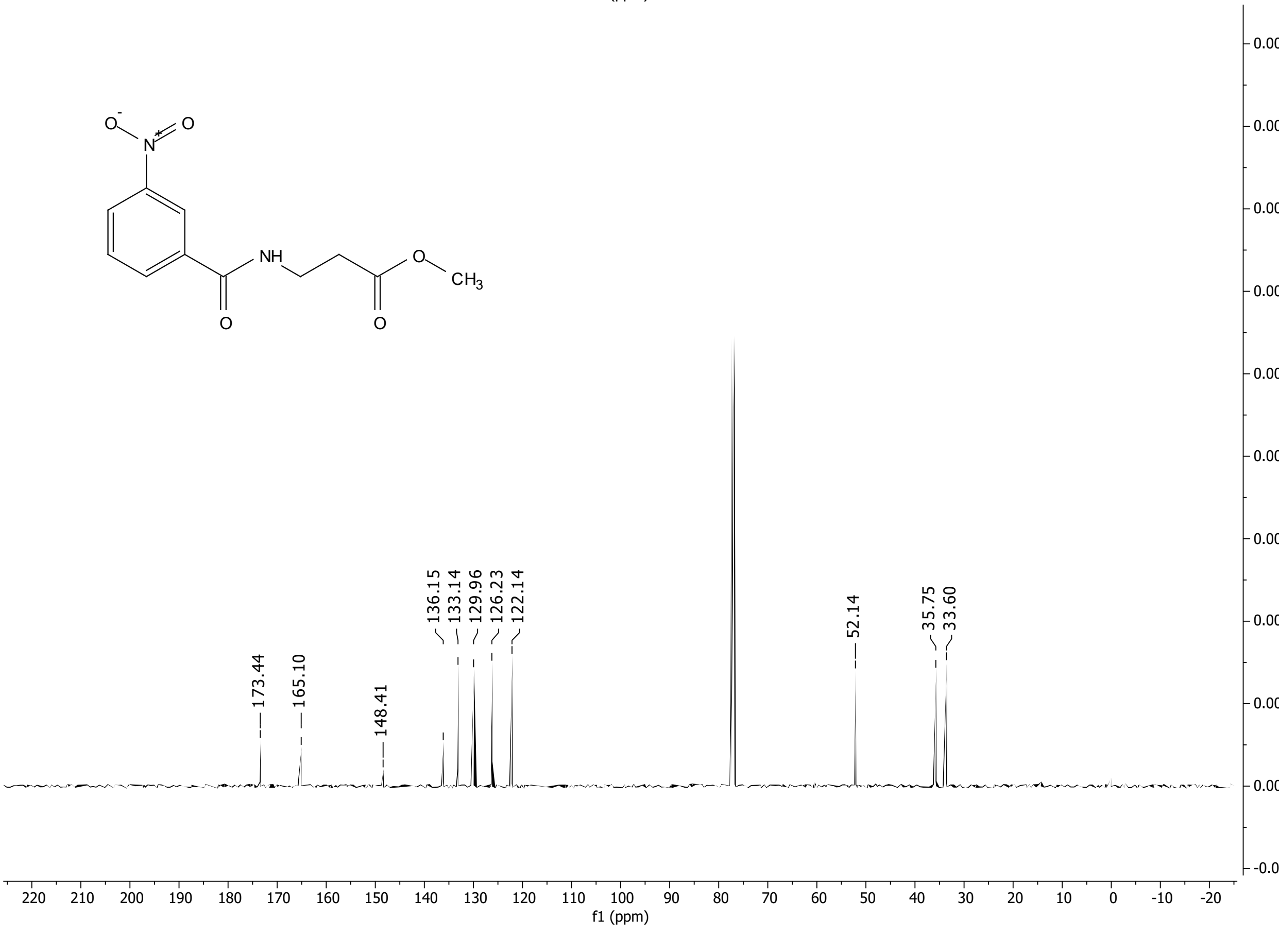

173.44
165.10
148.41
136.15 133.14 129.96 126.23 122.14
52.14
35.75 33.60
NH
O
CH3
f1 (ppm)

45

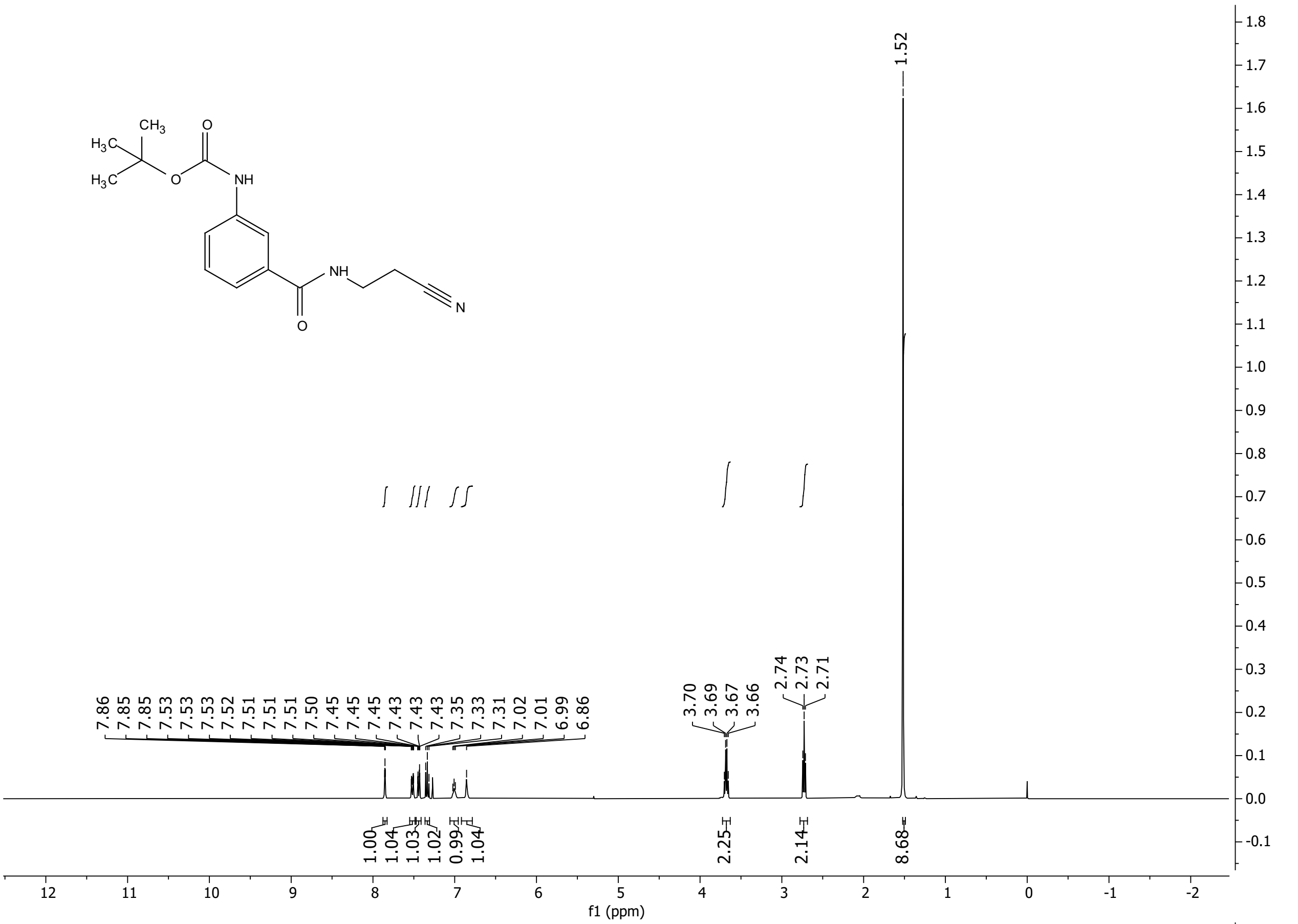
CH3
H3C
H3C
O
O
NH
NH
O
N
7.86 7.85 7.85 7.53 7.53 7.53 7.52 7.51 7.51 7.51 7.50 7.45 7.45 7.45 7.43 7.43 7.43 7.35 7.33 7.31 7.02 7.01 6.99 6.86
3.70 3.69 3.67 3.66
2.74 2.73 2.71
1.52
1.00 1.04 1.03 1.02 0.99 1.04
2.25
2.14
8.68
f1 (ppm)

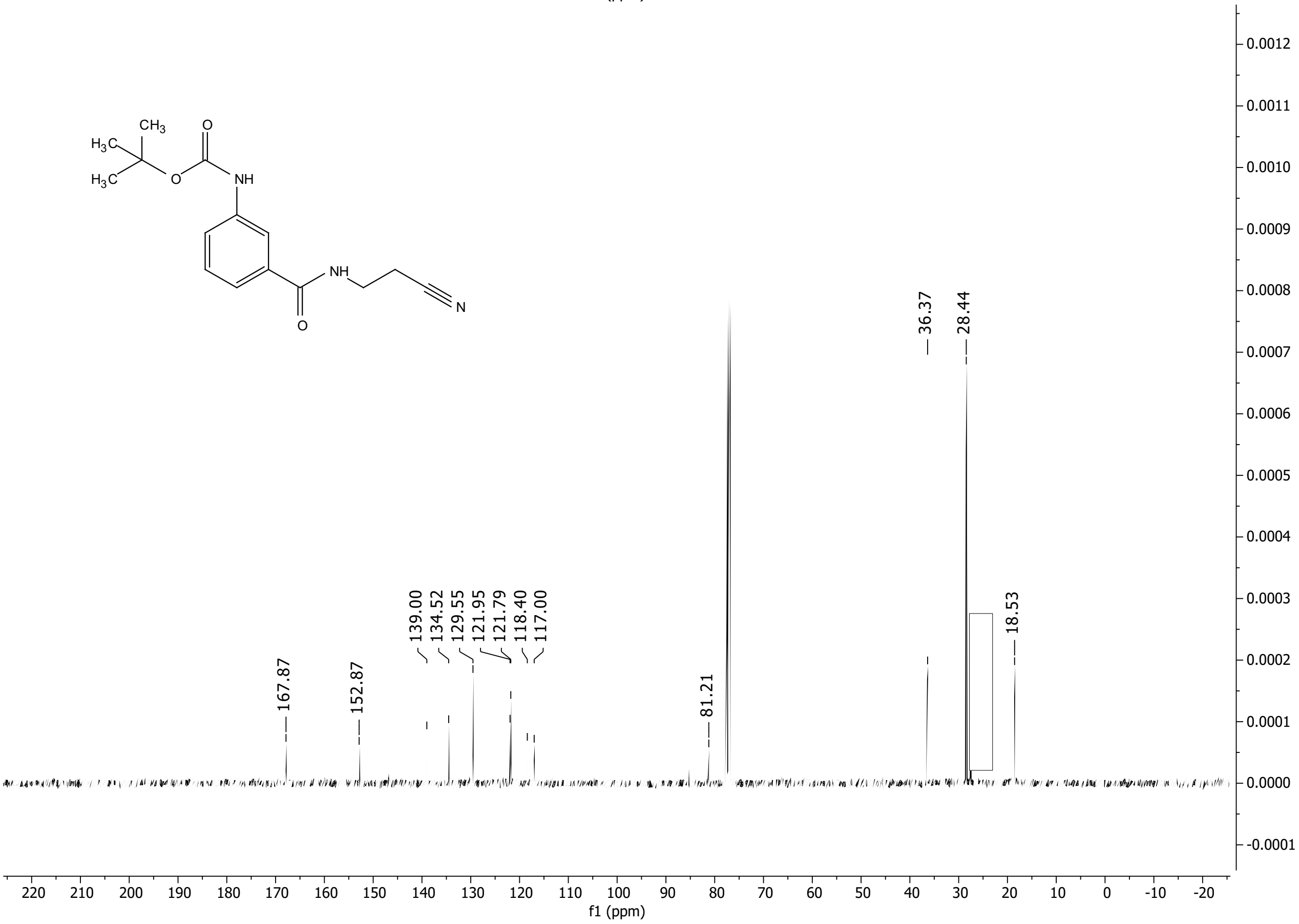
CH3
H3C
H3C
O
O
NH
NH
O
N
167.87
152.87
139.00 134.52 129.55 121.95 121.79 118.40 117.00
81.21
36.37
28.44
18.53
f1 (ppm)

46

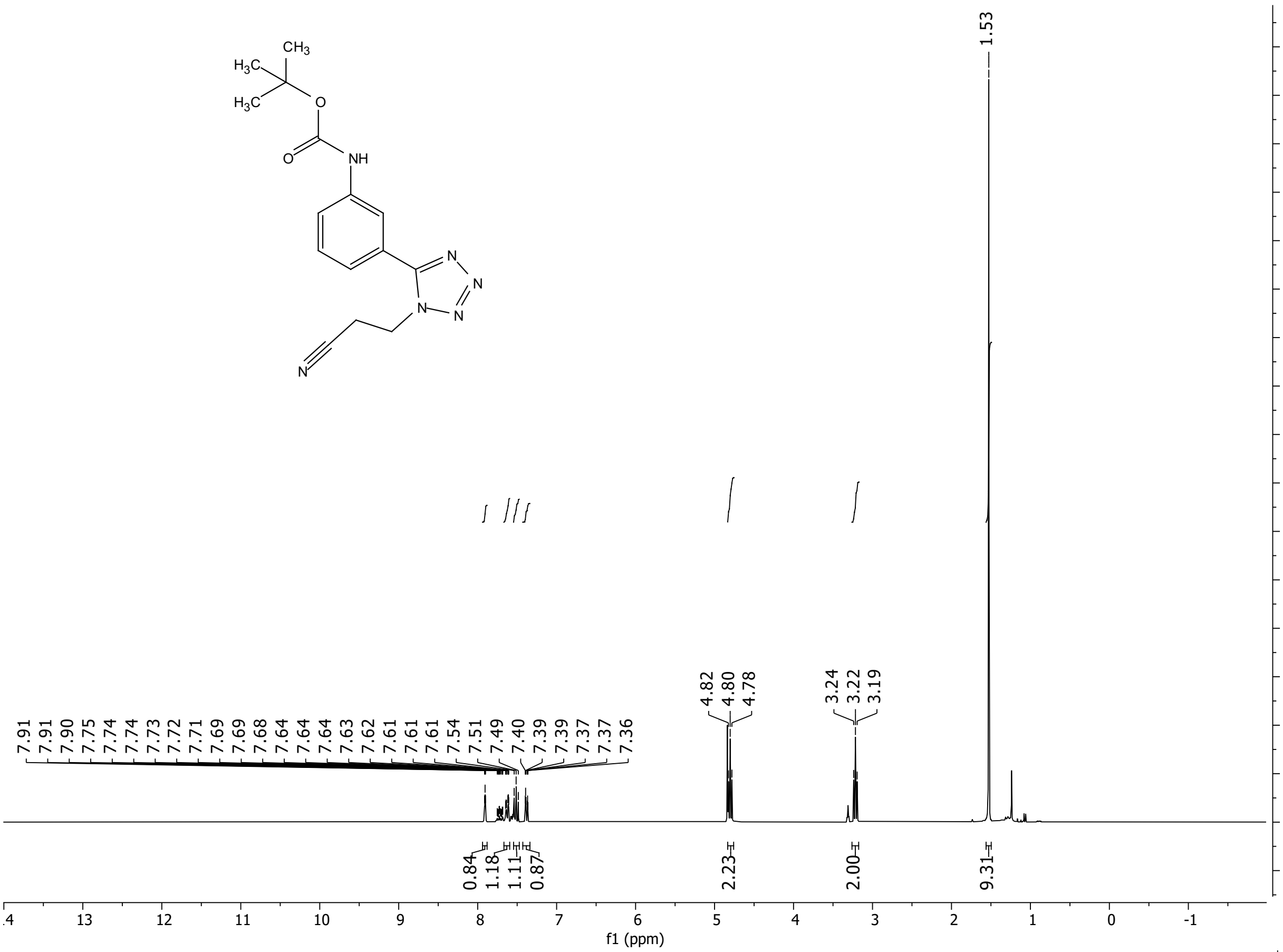
1.53
4.82
4.80
4.78
3.24
3.22
3.19
7.91 7.91 7.90 7.75 7.74 7.74 7.73 7.72 7.71 7.69 7.69 7.68 7.64 7.64 7.63 7.62 7.61 7.61 7.61 7.54 7.51 7.49 7.40 7.39 7.39 7.37 7.37 7.36
0.84
1.18
1.11
0.87
2.23
2.00
9.31
f1 (ppm)

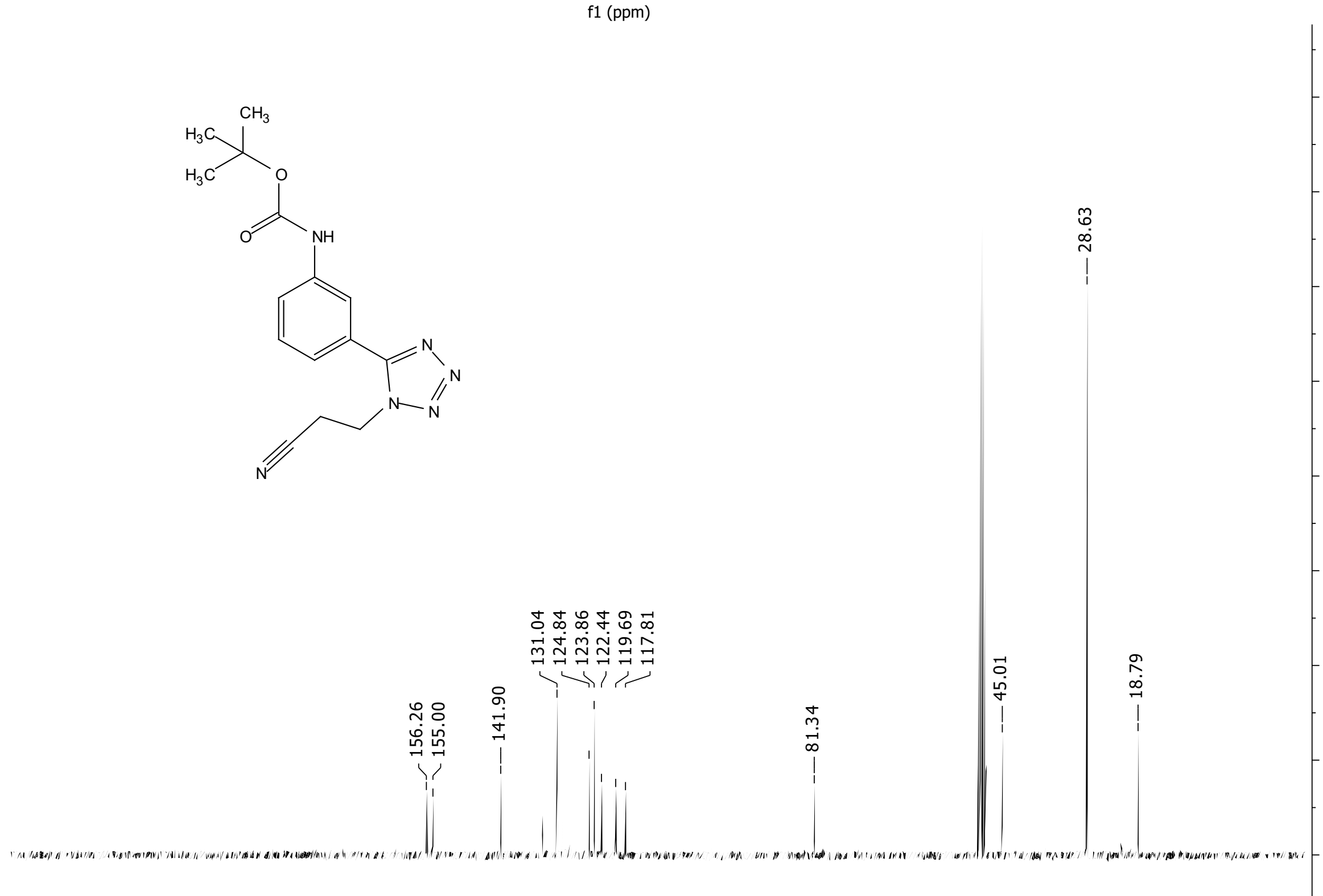
28.63
131.04
124.84
123.86
122.44
119.69
117.81
45.01
18.79
156.26
155.00
141.90
81.34
f1 (ppm)

18

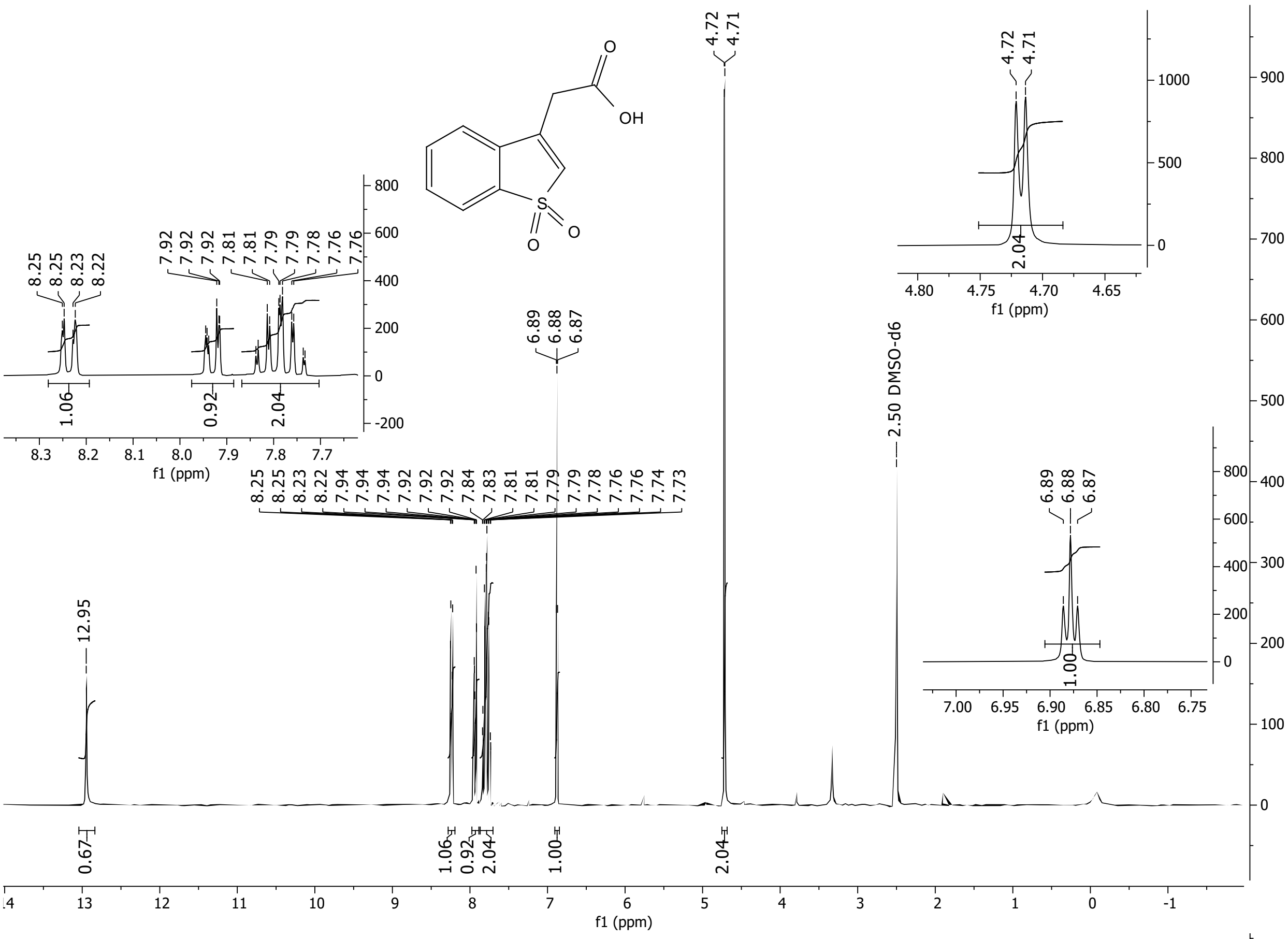

12.95
8.25 8.25 8.23 8.22 7.94 7.94 7.94 7.92 7.92 7.92 7.84 7.83 7.81 7.81 7.79 7.79 7.78 7.76 7.76 7.74 7.73
6.89 6.88 6.87
4.72 4.71
2.50 DMSO-d6
0.67 1.06 0.92 2.04 1.00 2.04
f1 (ppm)
OH
O
S

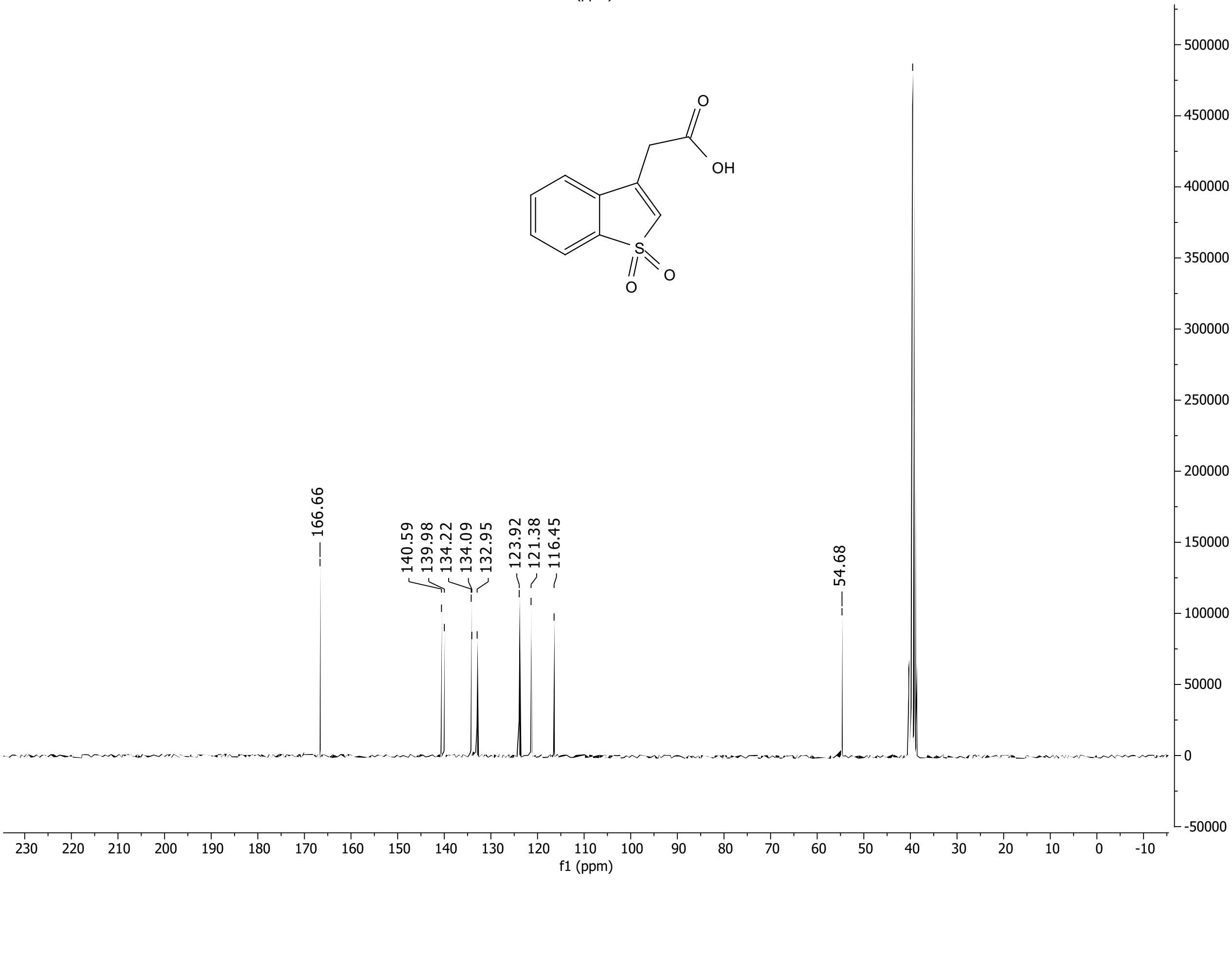

166.66
140.59 139.98 134.22 134.09 132.95
123.92 121.38 116.45
54.68
f1 (ppm)
OH
O
S

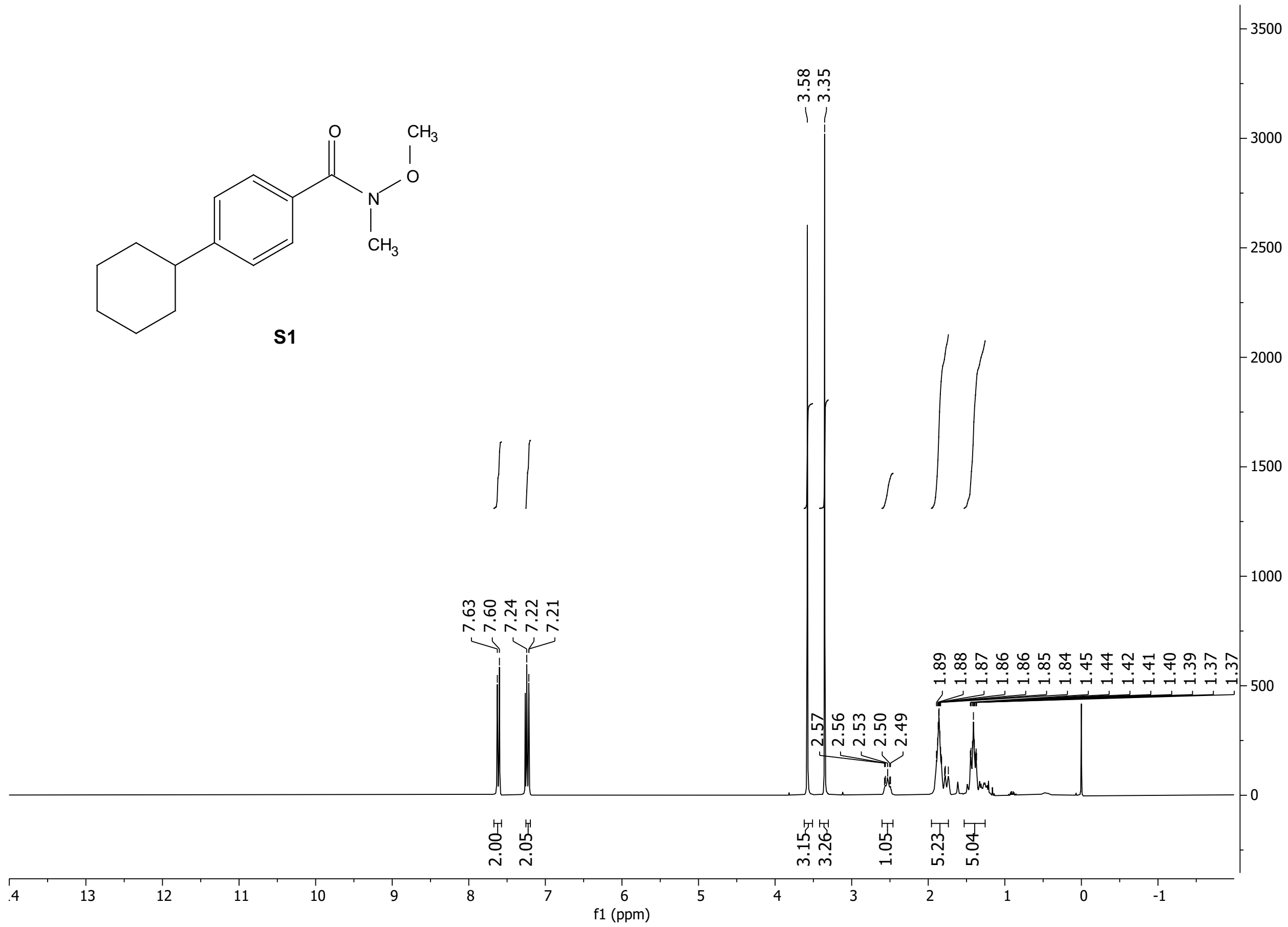

S1

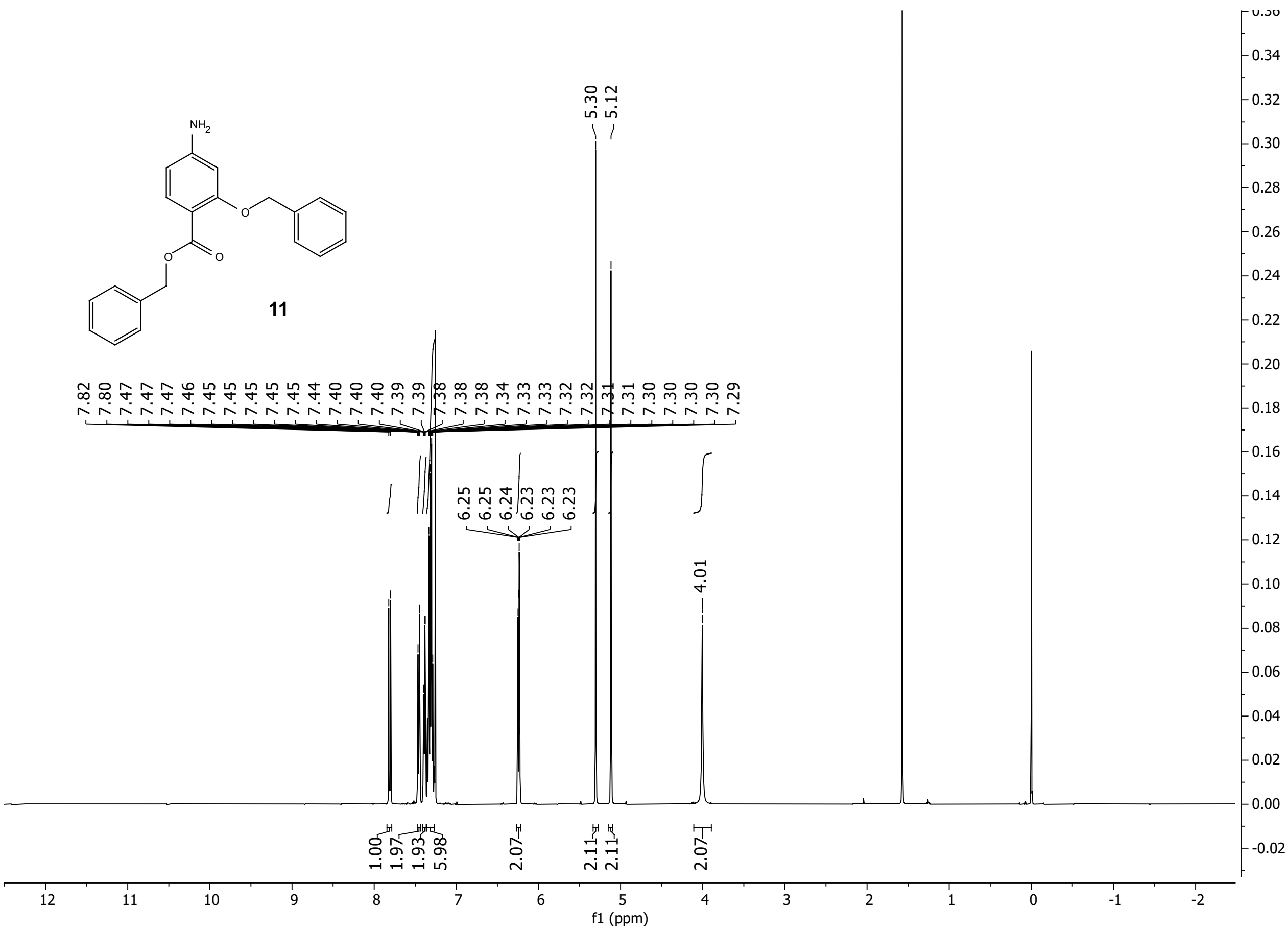

11

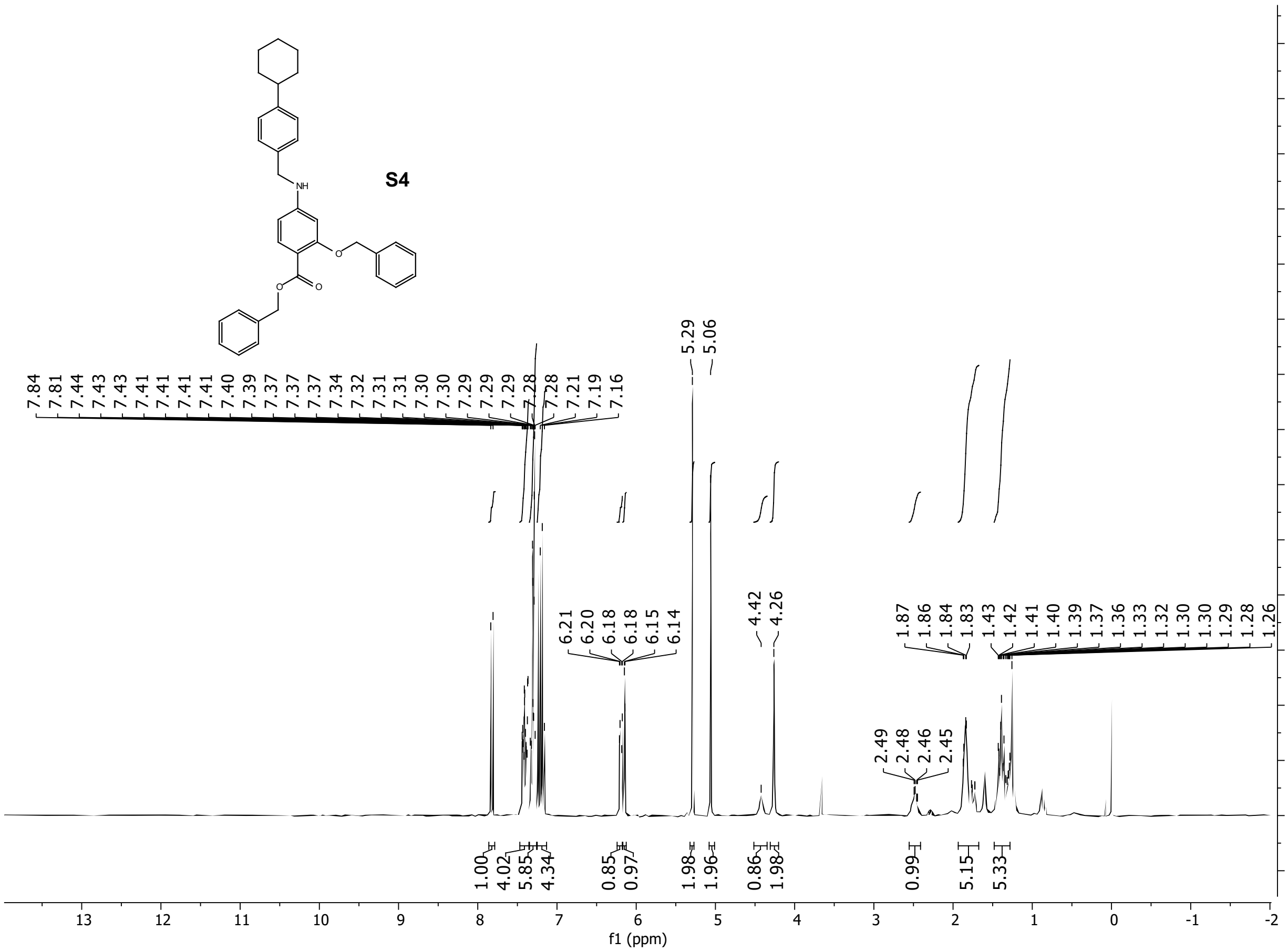
S4
f1 (ppm)

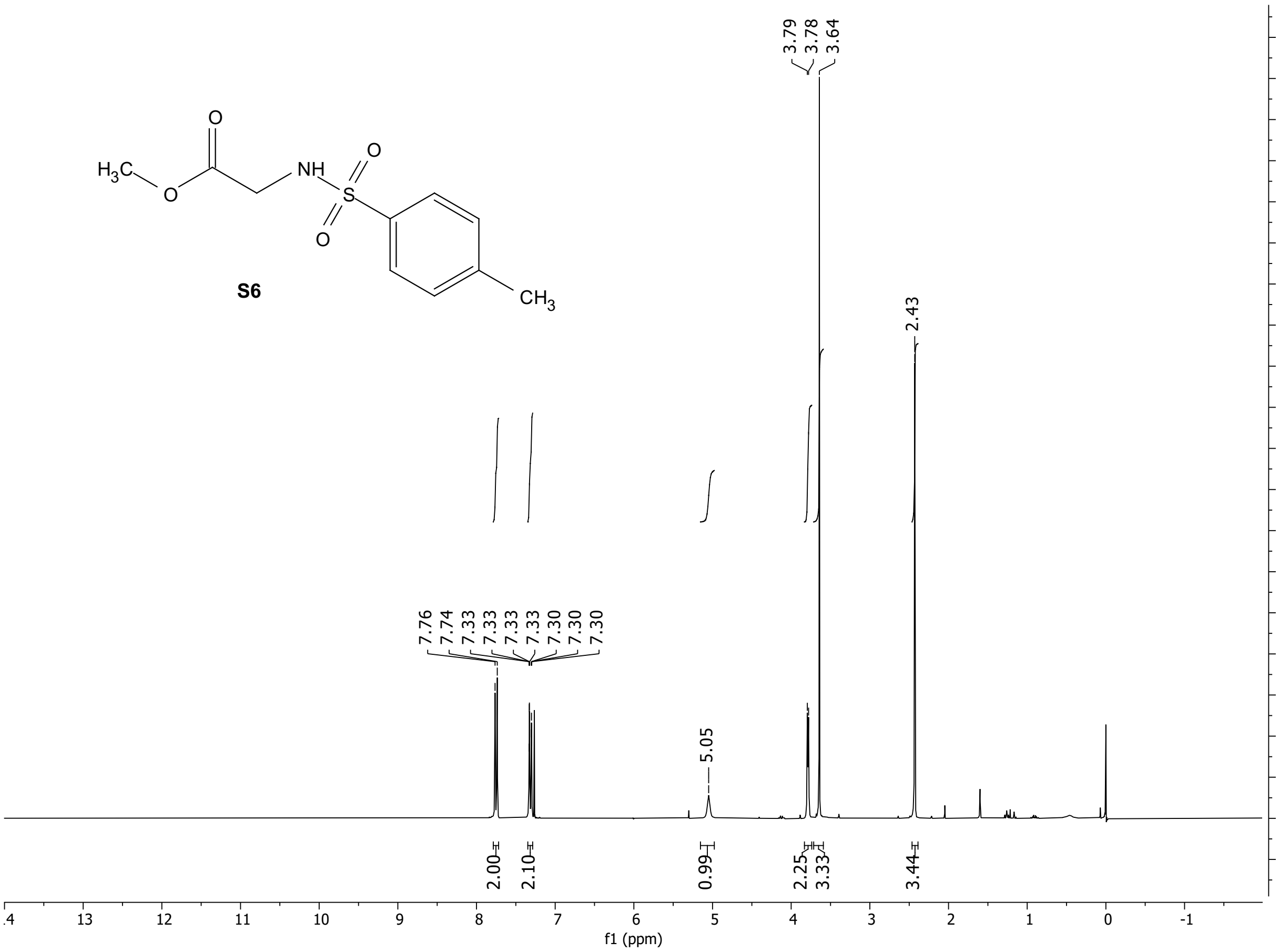
S6
f1 (ppm)

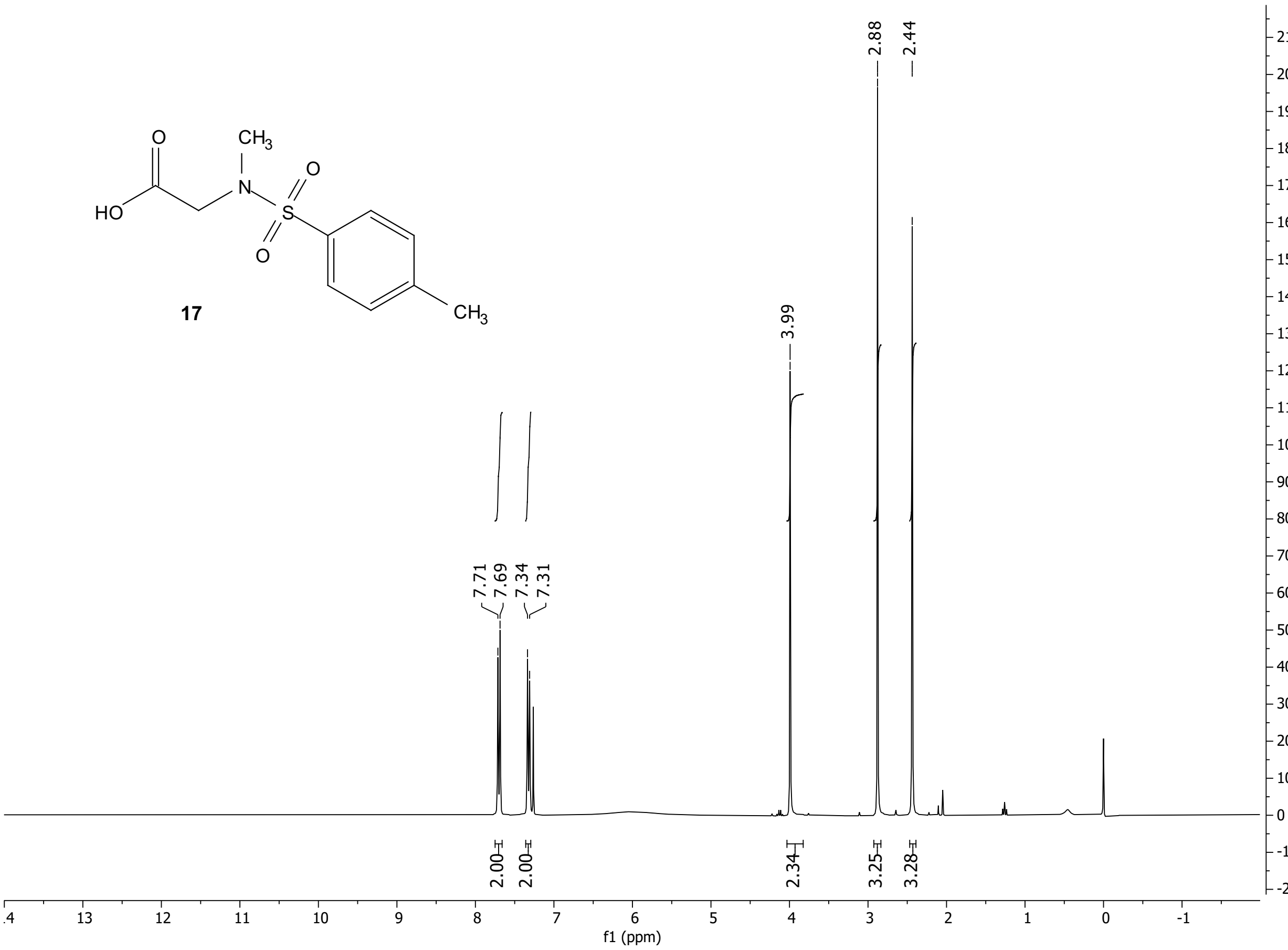

17
7.71
7.69
7.34
7.31
3.99
2.88
2.44
2.00
2.00
2.34
3.25
3.28
f1 (ppm)

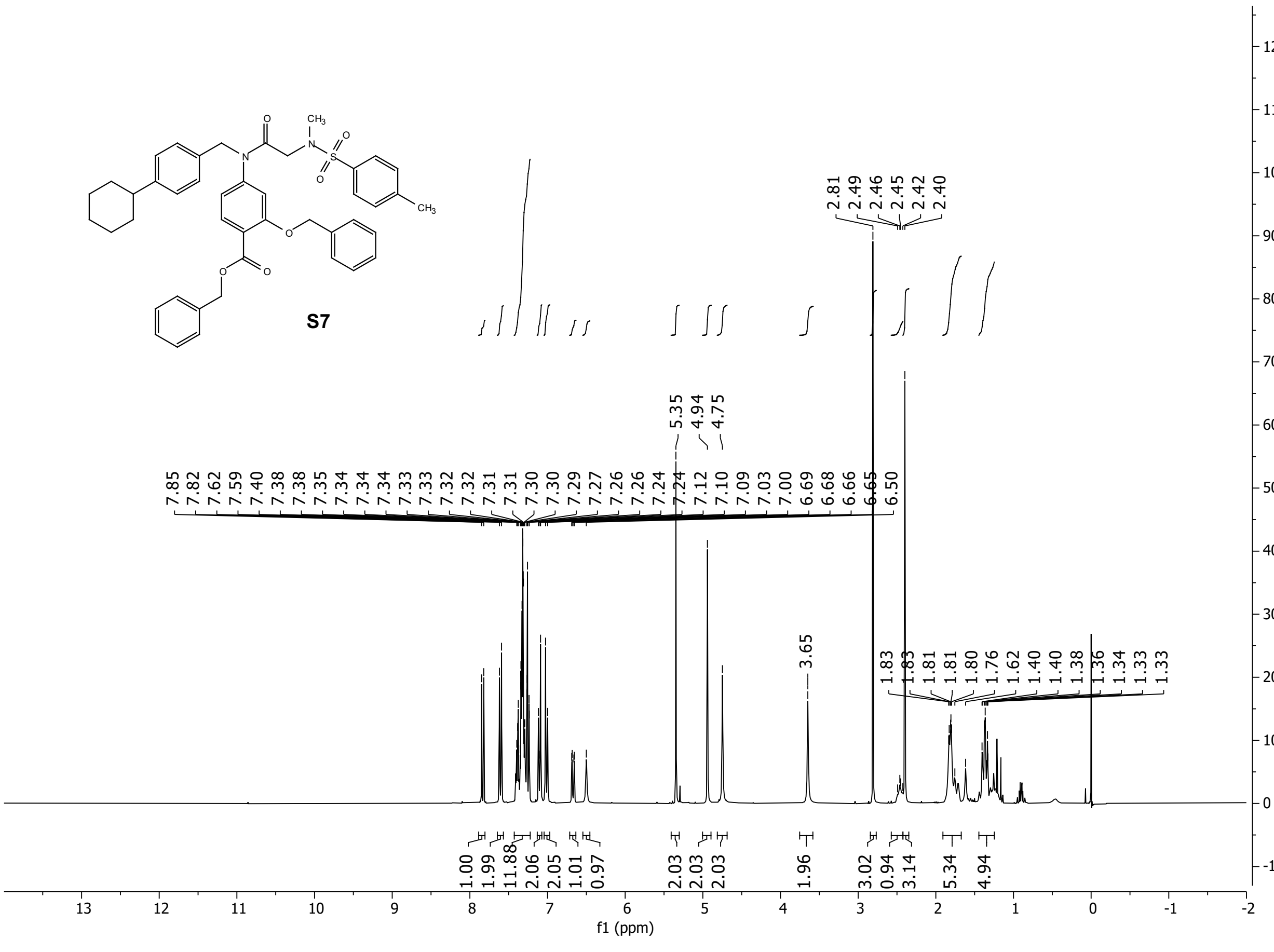

S7
7.85 7.82 7.62 7.59 7.40 7.38 7.38 7.35 7.34 7.34 7.34 7.33 7.33 7.32 7.32 7.31 7.31 7.30 7.30 7.29 7.27 7.26 7.26 7.24 7.24 7.12 7.10 7.09 7.03 7.00 6.69 6.68 6.66 6.65 6.50
5.35
4.94
4.75
3.65
2.81 2.49 2.46 2.45 2.42 2.40
1.83 1.83 1.81 1.81 1.80 1.76 1.62 1.40 1.40 1.38 1.36 1.34 1.33 1.33
1.00 1.99 11.88 2.06 2.05 1.01 0.97 2.03 2.03 2.03 1.96 3.02 0.94 3.14 5.34 4.94
f1 (ppm)

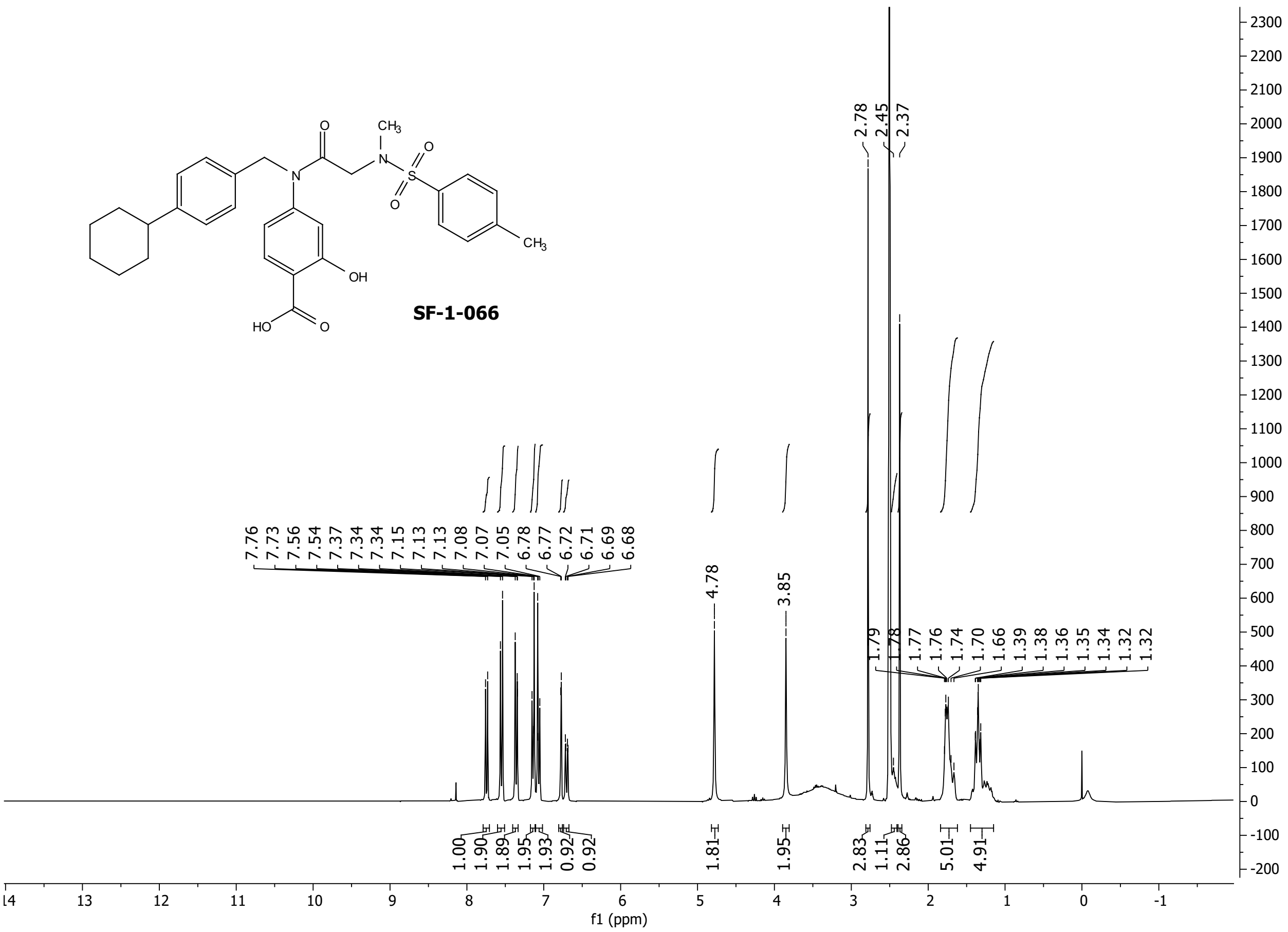
SF-1-066
f1 (ppm)

3. HPLC copies for compounds 2a-w, and SF-1-066

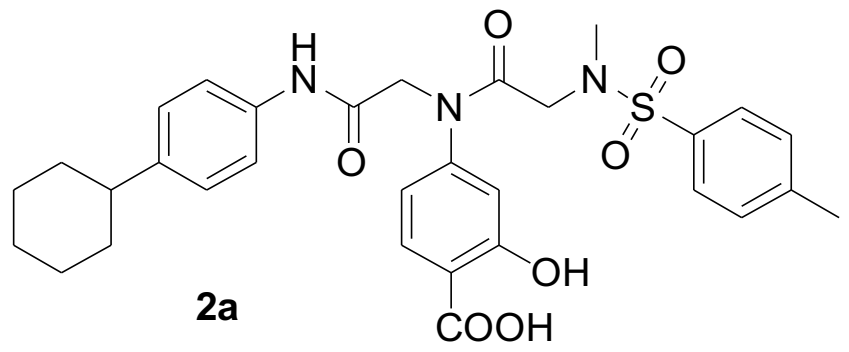

2a

Peak #	RetTime [min]	Type	Width [min]	Area mAU *s	Height [mAU]	Area %
1	8.441	MM	0.1790	66.29407	6.17334	0.7777
2	13.116	MM	0.1665	34.27523	3.43145	0.4021
3	13.633	MM	0.3289	8424.03711	426.86365	98.8202

Column Gemini C18 150x3mm 5u + precolumn + inlet filter. Conc: 300ug/mL (CH3CN/H2O+0,1%FA 1:1); flow=0.5ml/min; Vinj 5ul; Temp: 26°C Term. OFF, VWD=220nm; grad. A=H2O+0,1%FA, B=C H3CN+0,1%FA, 0min A=50%, 20min A=0%

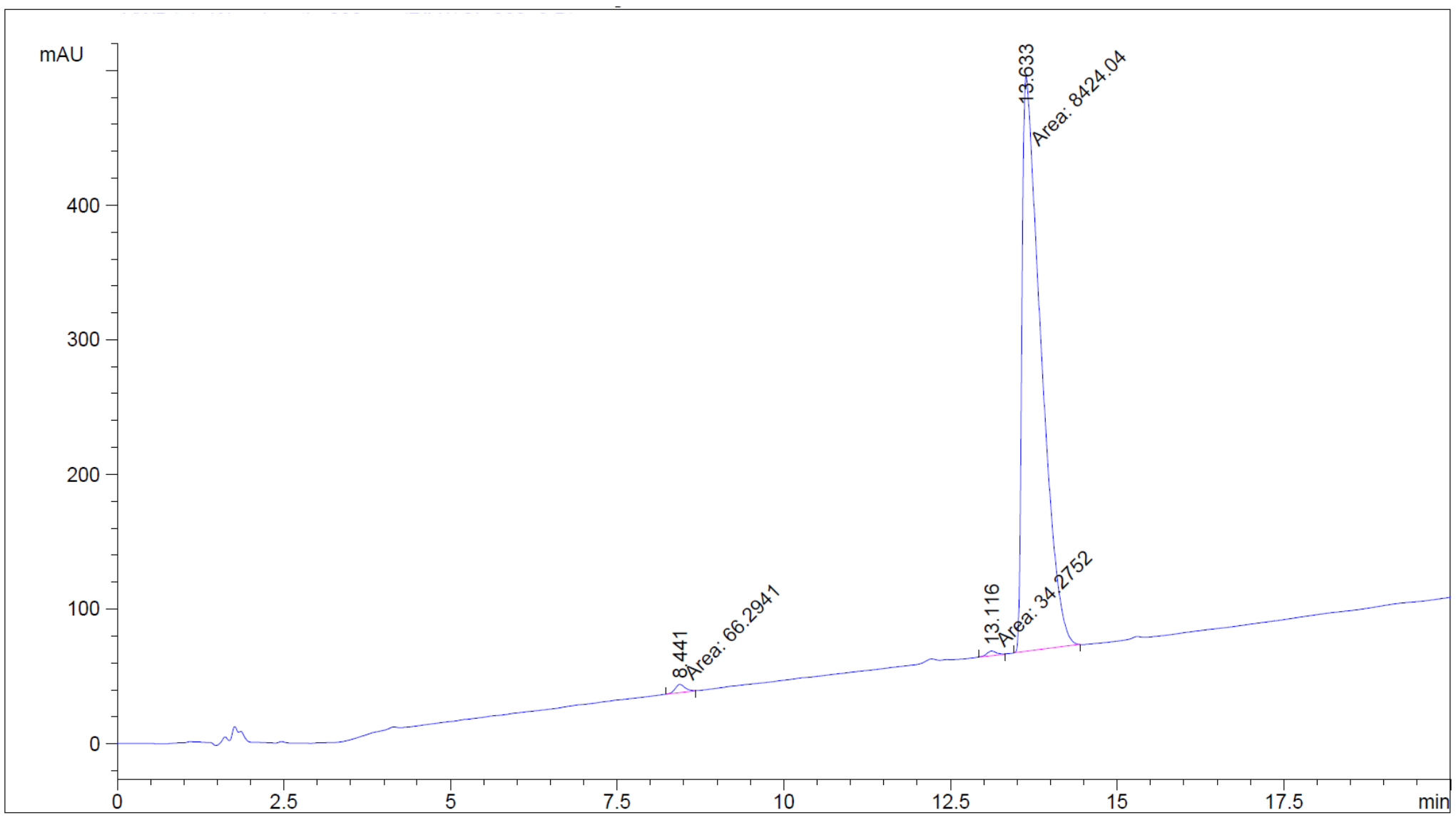

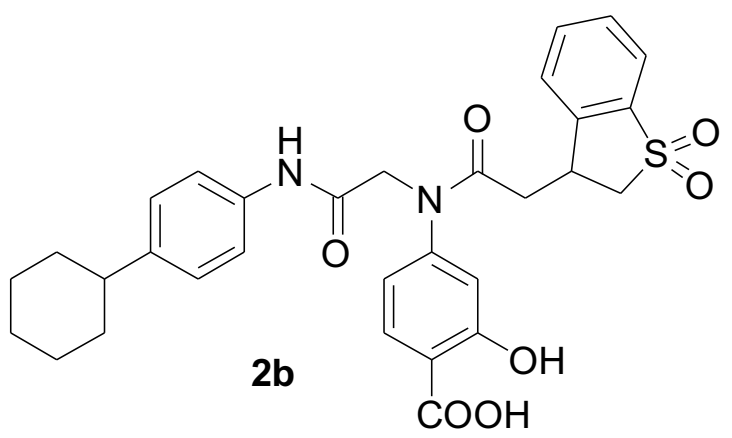

2b

Peak #	RetTime [min]	Type	Width [min]	Area mAU *s	Height [mAU]	Area %
1	10.889	MM	0.2638	6183.81201	390.73676	98.9405
2	12.409	MM	0.2429	66.21924	4.54394	1.0595

Column Gemini C18 150x3mm 5u + precolumn + inlet filter; Conc:300ug/ml (CH3CN/H2O+0,1%FA 1:1); flow=0,5ml/min;; Vinj 5ul; Temp: 26°C Term.OFF, VWD=220nm; grad. A=H2O+0,1%FA, B=CH3CN+0,1%FA, 0min A=50%, 20min A=0%

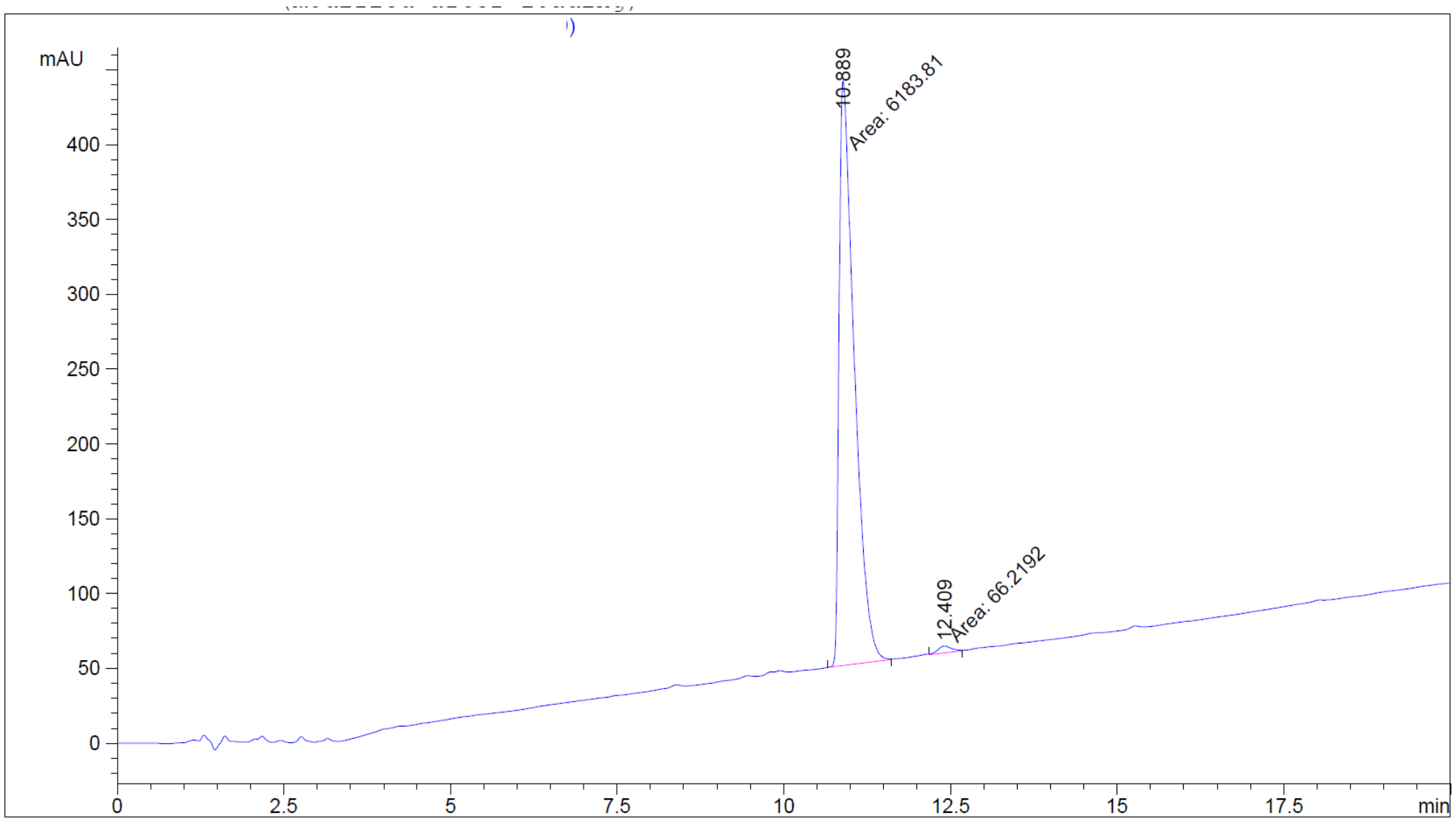

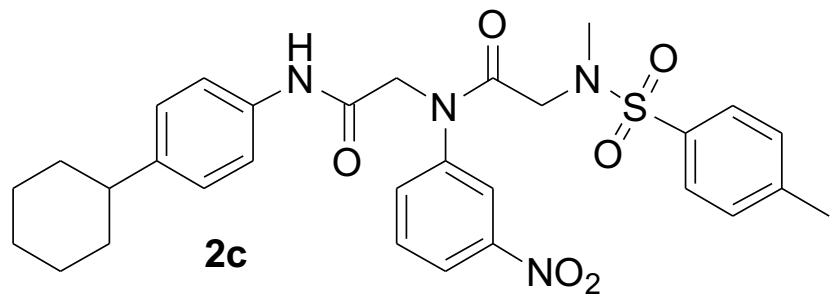

2c

Peak #	RetTime [min]	Type	Width [min]	Area mAU *s	Height [mAU]	Area %
1	14.047	MM	0.1540	3505.67700	379.51538	100.0000

Column Gemini C18 150x3mm 5u + precolumn + inlet filter; Conc: 300ug/ml (CH3CN/H2O+0,1%FA 1:1 +1%DMSO); flow=0,5ml/min;; Vinj 5ul; Temp: 24°C Term.OFF, VWD=220nm; grad. A=H2O+0,1%FA, B=CH3CN+0,1%FA, 0min A=50%, 20min A=0%

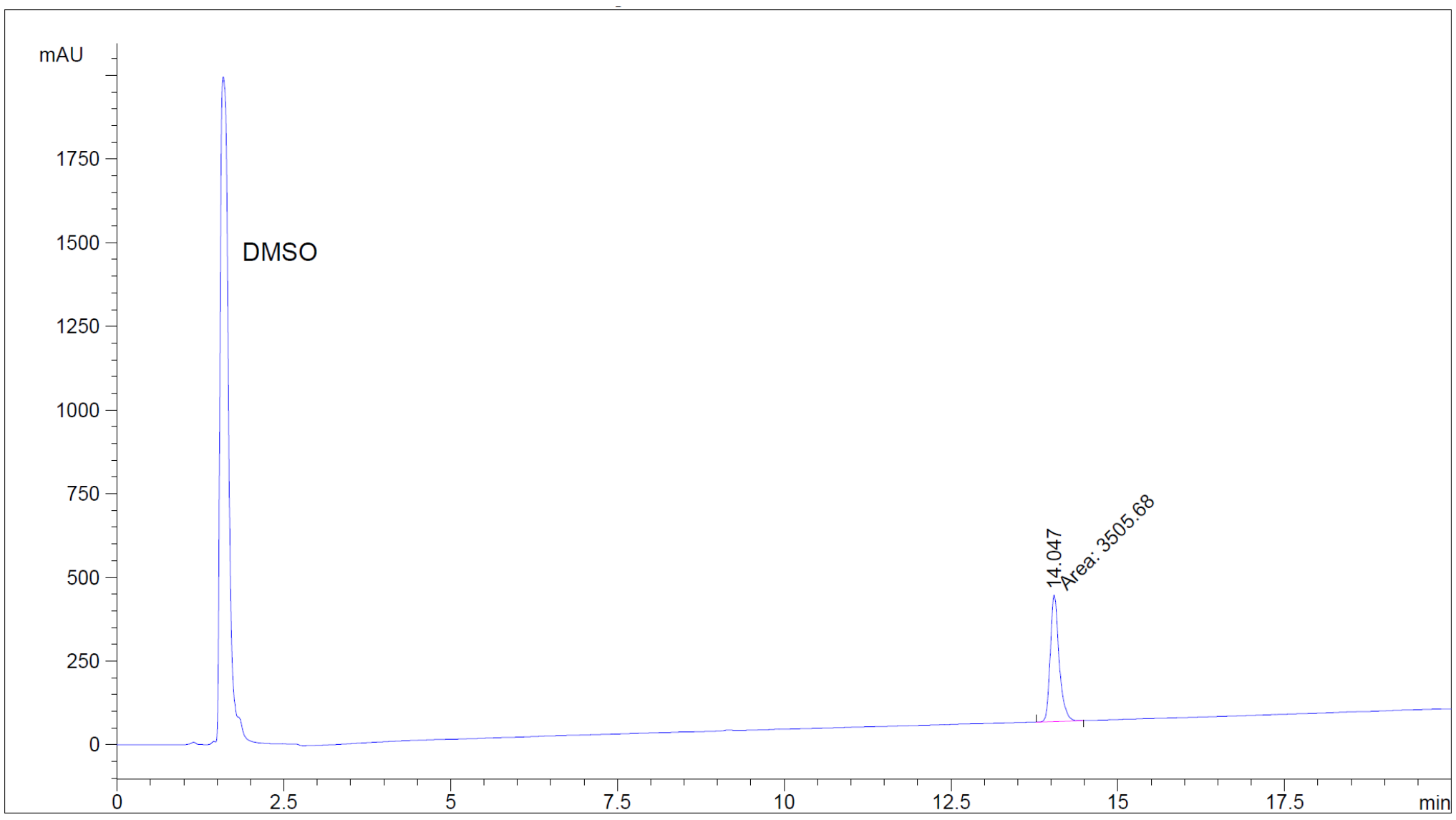

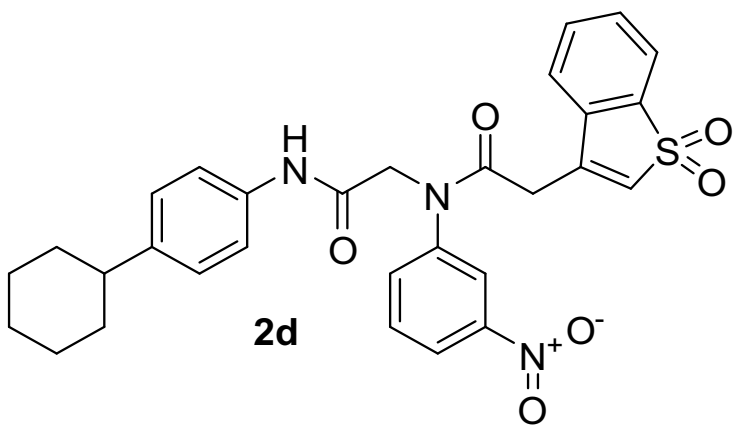

2d

Peak #	RetTime [min]	Type	Width [min]	Area mAU *s	Height [mAU]	Area %
1	18.112	MF	0.1254	3731.05615	495.74347	97.7498
2	18.673	FM	0.1286	46.81221	6.06639	1.2264
3	21.418	MM	0.1196	39.07543	5.44304	1.0237

Column Phenylic C6 150x3mm 3u + precolumn + inlet filter; Conc:100ug/ml (CH3CN + circa 1%DMSO); flow=0,34ml/min; Vinj 5ul; Temp: 25°C Term.ON, VWD=220nm; grad.A=H2O, B=CH3CN,0min A=70%, 20min A=0%

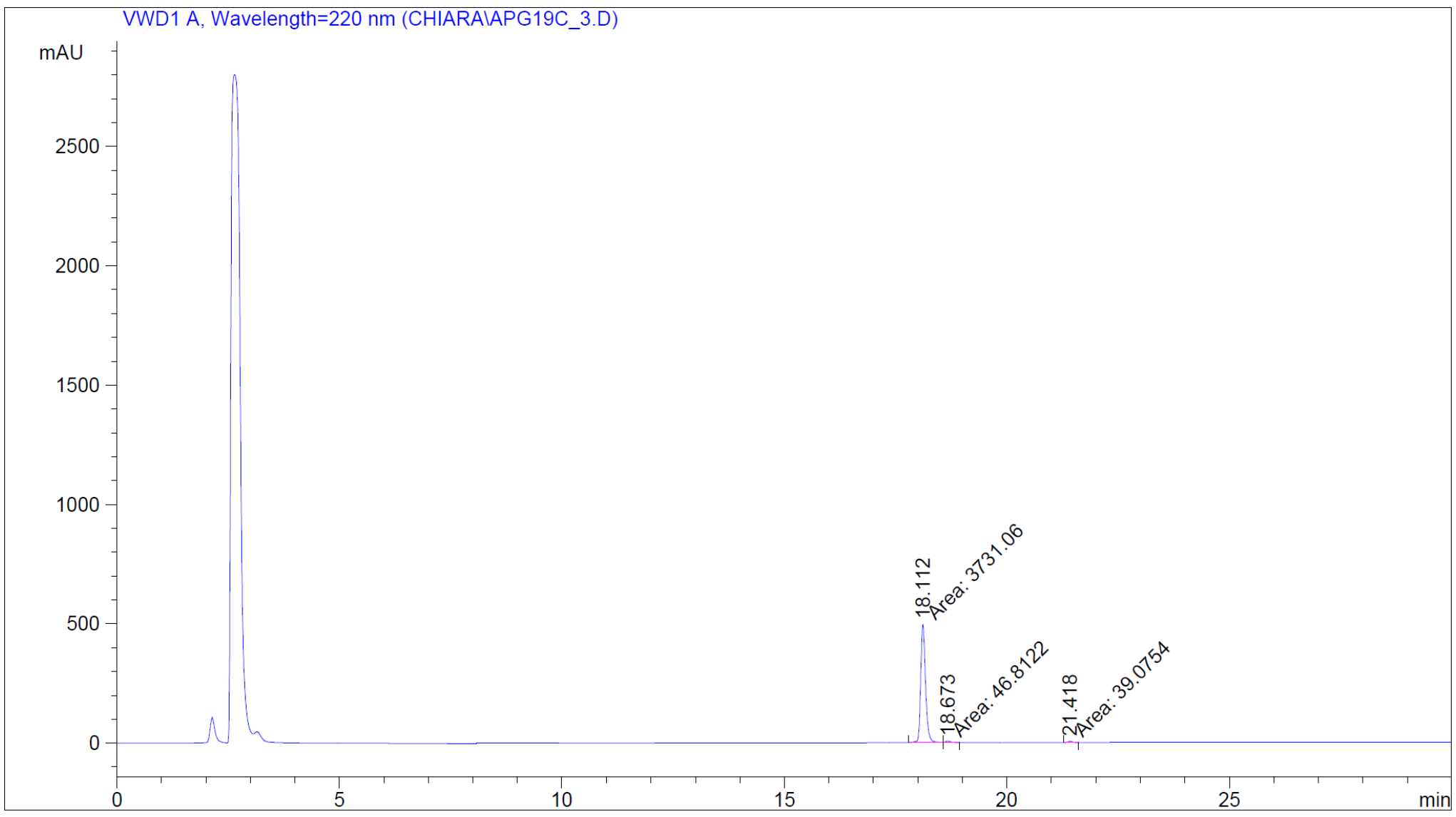

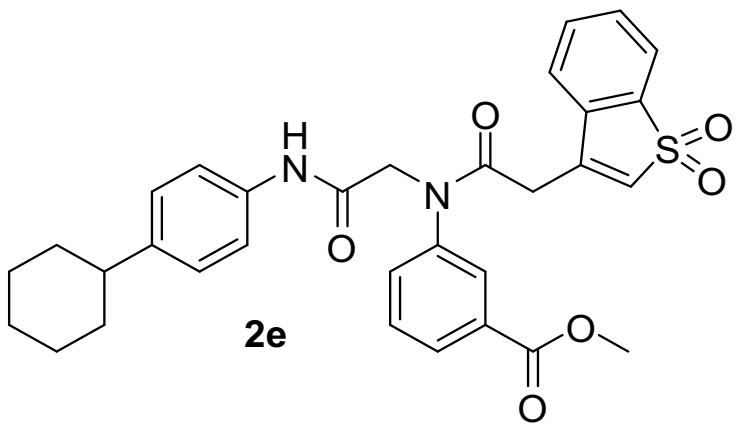

2e

Peak #	RetTime [min]	Type	Width [min]	Area mAU *s	Height [mAU]	Area %
1	11.826	MM	0.1403	81.58263	9.69099	1.1300
2	12.244	MM	0.1543	7138.10254	770.90906	98.8700

Column Gemini C18 150x3mm 5u + precolumn + inlet filter; Conc:200ug/ml (CH3CN+0,1%FA+1%DMSO); flow=0,5ml/min; Vinj 5ul; Temp: 26 °C Term.OFF, VWD=220nm; grad. A=H2O+0,1%FA, B=CH3CN+0,1%FA, 0min A=50%, 20min A=0%

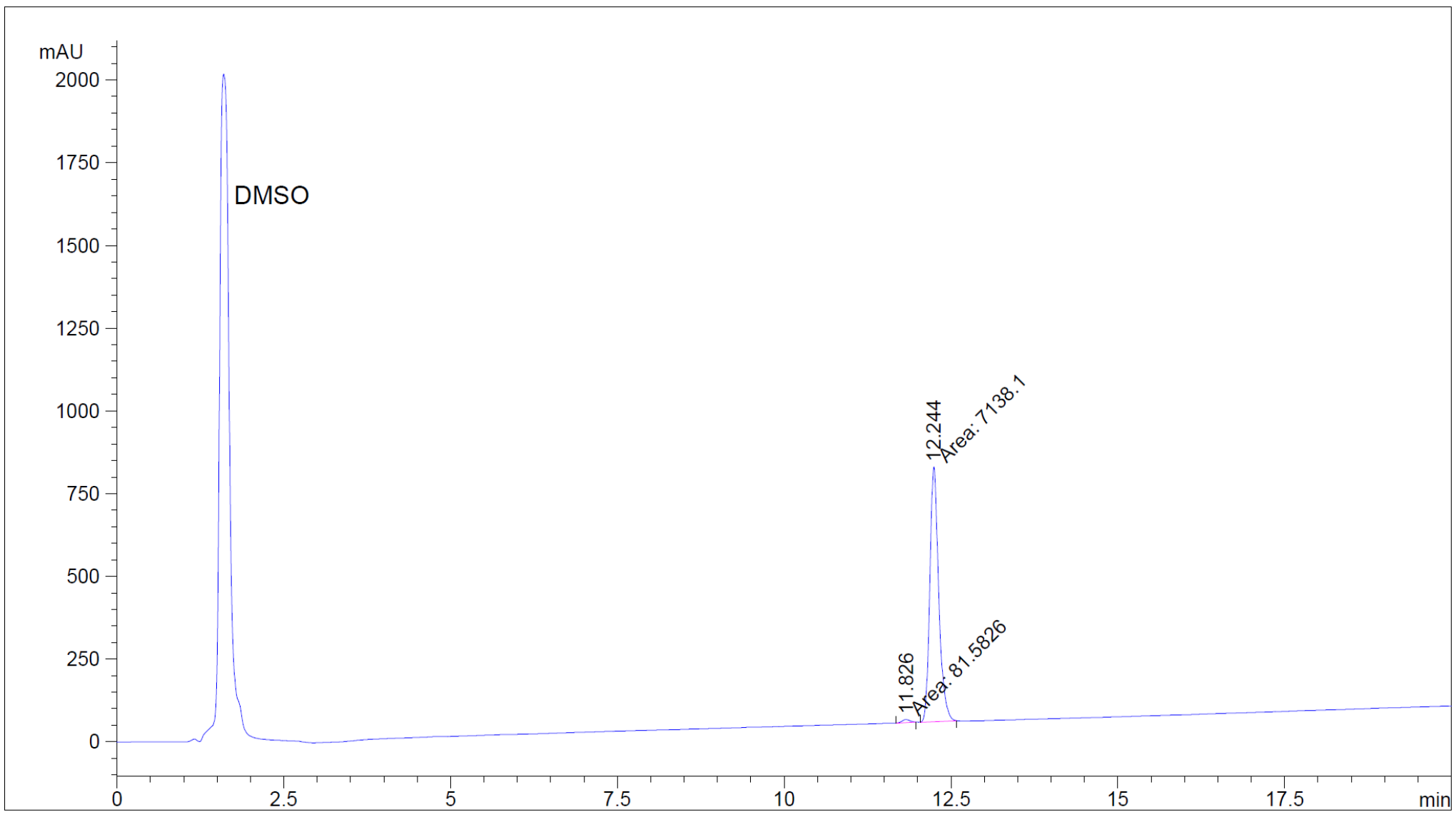

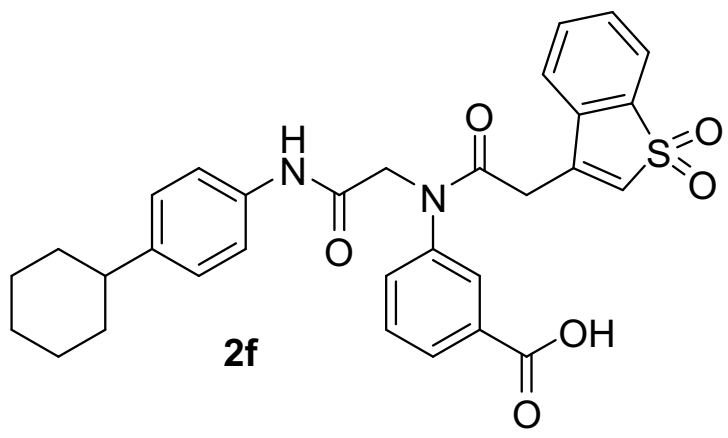

2f

Peak #	RetTime [min]	Type	Width [min]	Area mAU *s	Height [mAU]	Area %
1	8.902	MF	0.1866	55.88973	4.99162	0.7051
2	9.170	FM	0.1528	7649.52051	834.14954	96.5010
3	12.307	MM	0.1577	221.47359	23.40824	2.7940

Column Gemini C18 150x3mm 5u + precolumn + inlet filter; Conc:250ug/ml (CH3CN+0,1%FA +1% DMSO); flow=0,5ml/min; Vinj 5ul; Temp: 24°C Term.OFF, VWD=220nm; grad. A=H2O+0,1%FA, B=CH3CN+0,1%FA, 0min A=50%, 20min A=0%

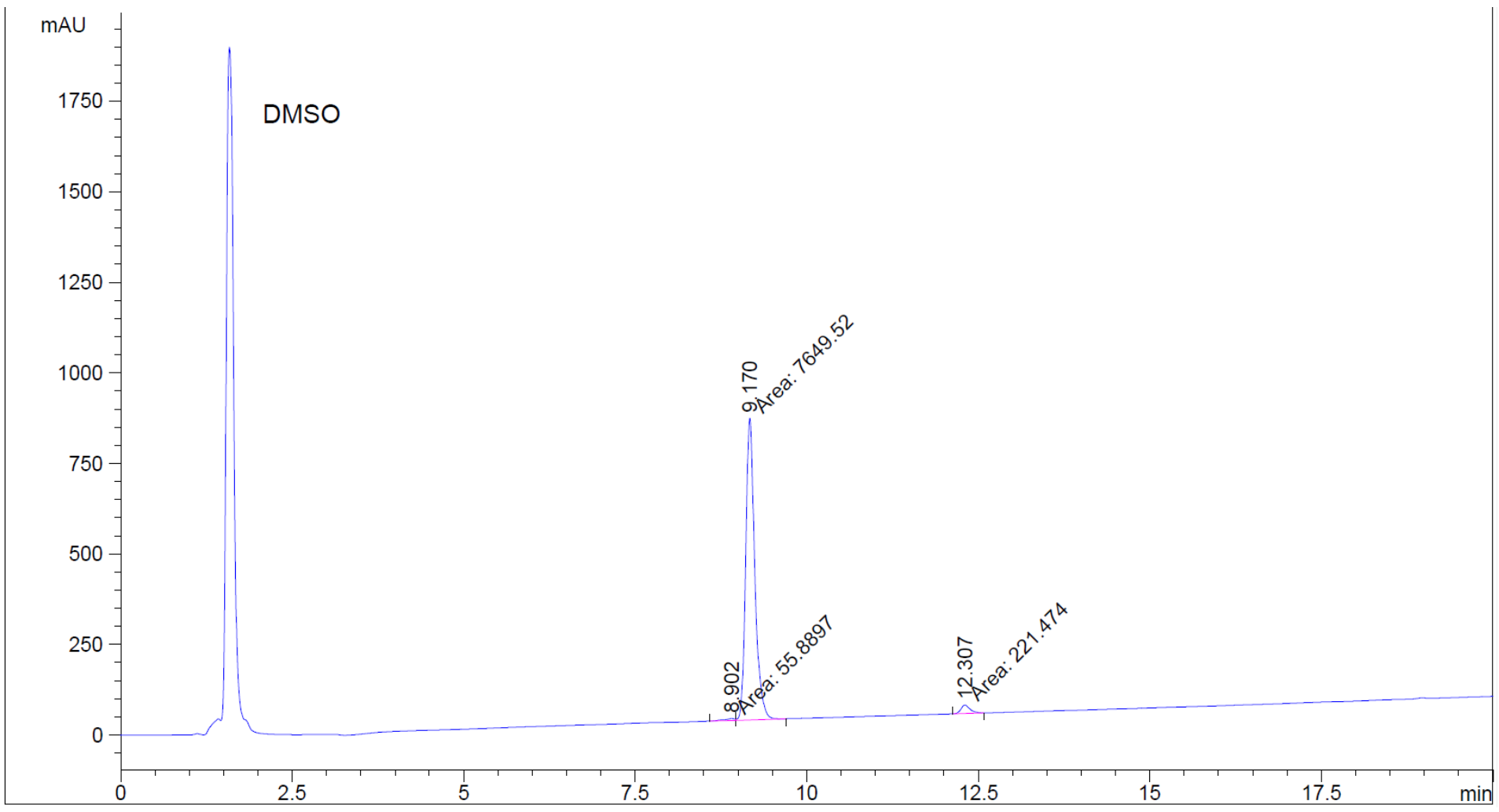

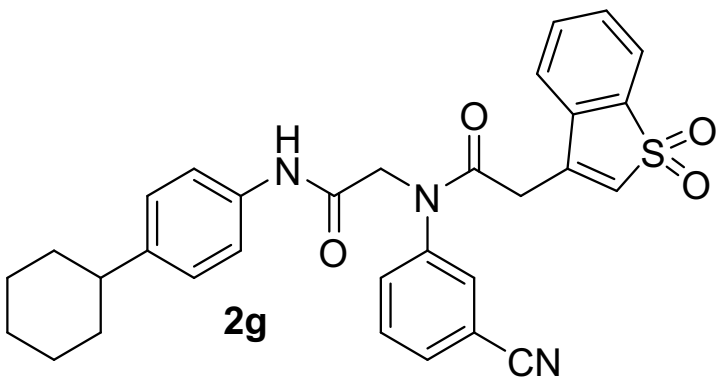

2g

Peak #	RetTime [min]	Type	Width [min]	Area mAU *s	Height [mAU]	Area %
1	17.677	MF	0.1262	4809.75195	635.24506	97.8631
2	18.284	FM	0.1287	23.84499	3.08881	0.4852
3	21.412	VB	0.1109	81.18026	11.17664	1.6518

Column Phenylic C6 150x3mm 3u + precolumn + inlet filter; Conc:100ug/ml (CH3CN + circa 0.5%DMSO); flow=0,34ml/min; Vinj 5ul; Temp: 25°C Term.ON, VWD=220nm; grad.A=H2O, B=CH3CN,0min A=70%, 20min A=0%

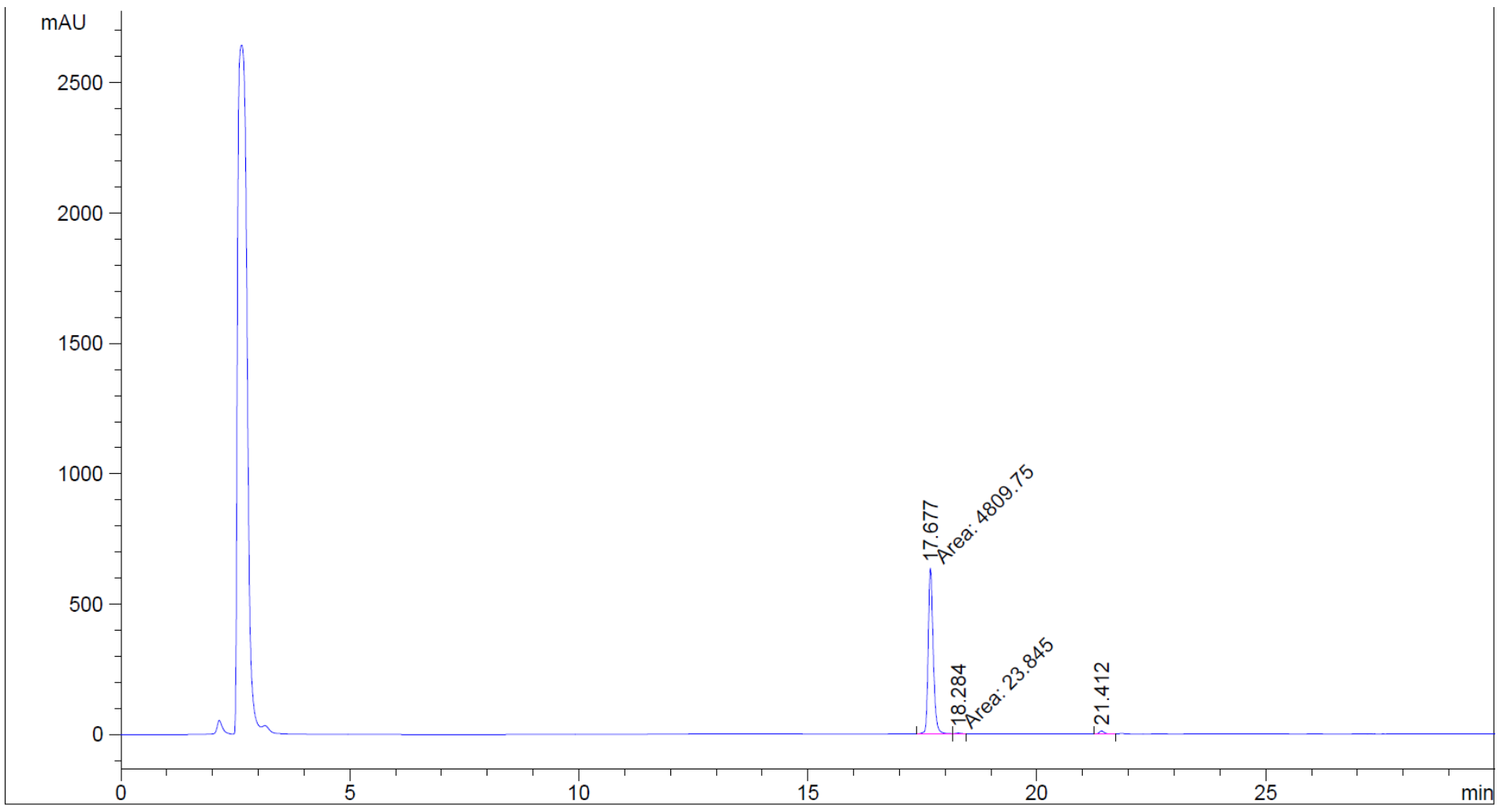

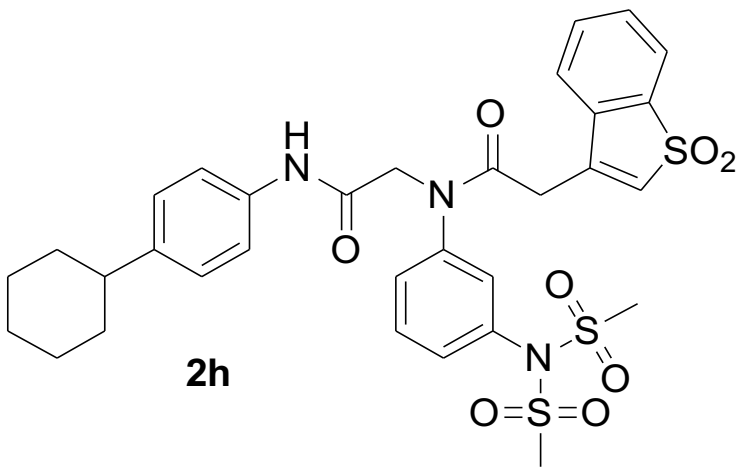

2h

Signal 1: VWD1 A, Wavelength=220 nm

Peak #	RetTime [min]	Type	Width [min]	Area mAU *s	Height [mAU]	Area %
1	15.945	MM	0.0866	33.87428	6.51667	1.1800
2	19.639	MF	0.1127	63.74095	9.42321	2.2204
3	19.902	FM	0.0950	2773.07813	486.58725	96.5996

Column ACE Excel 3 C18-AR 150 x 3 mm, 3 micron + precolumn + inline filter Conc:200ug/ml (CH3CN/H2O+0,1%FA 1:1); flow=0,38ml/min; Vinj 5ul; Temp: 30°C Term.ON, VWD=220nm ; grad.A=H2O+0,1%FA, B=CH3CN+0,1%FA,0min A=90%, 20min A=0%

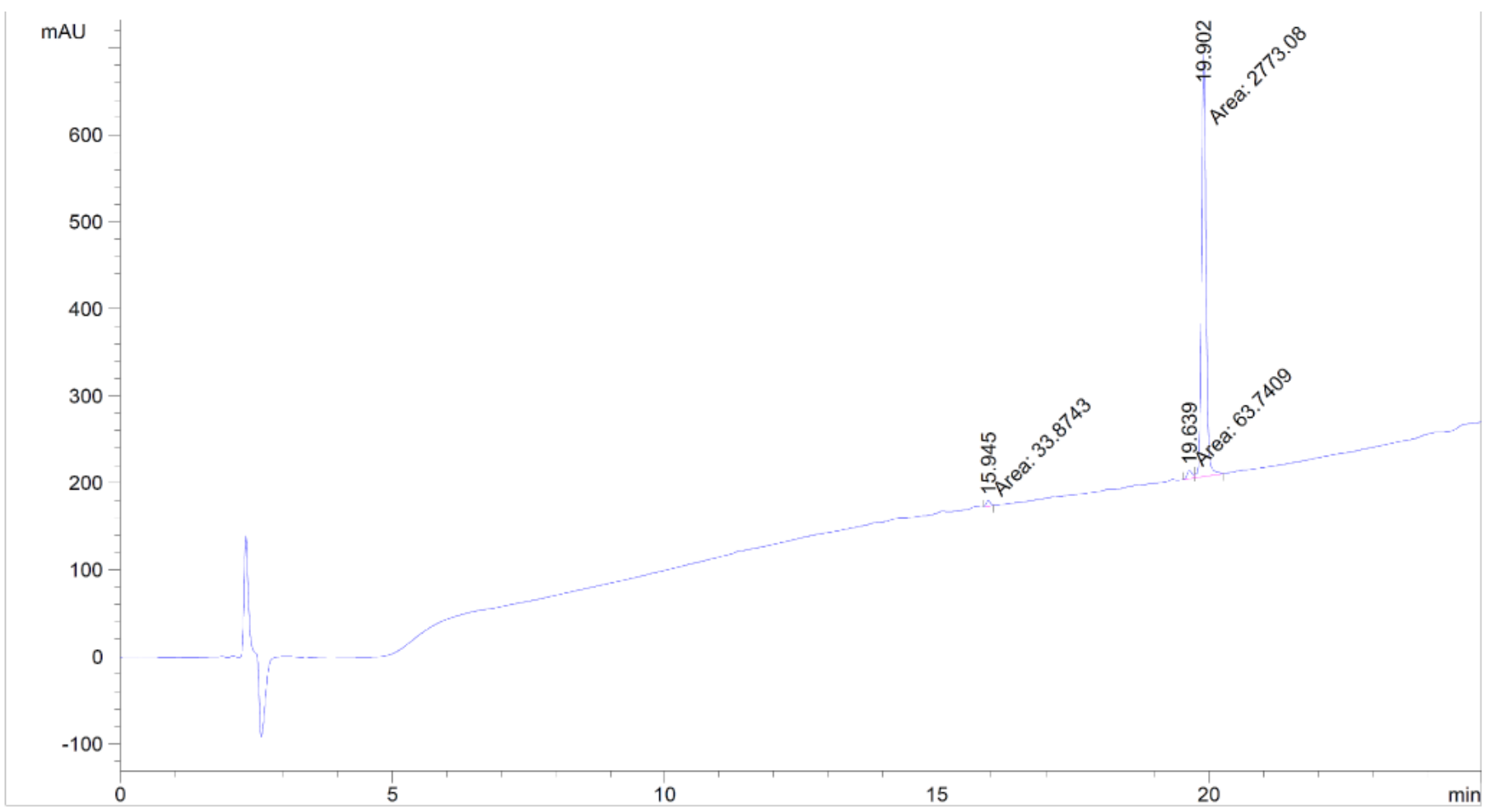

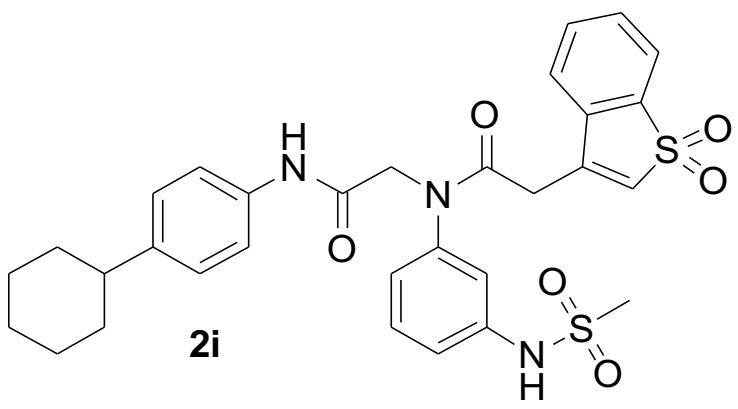

2i

Peak #	RetTime [min]	Type	Width [min]	Area mAU *s	Height [mAU]	Area %
1	9.571	MM	0.1483	9117.73828	1024.72180	97.5832
2	10.806	MM	0.1805	123.50746	11.40655	1.3218
3	11.823	MM	0.1515	25.80654	2.83897	0.2762
4	18.989	MM	0.1570	76.50516	8.12001	0.8188

Column Gemini C18 150x3mm 5u + precolumn + inlet filter; Conc:300ug/ml (CH3CN/H2O+0,1%FA 1:1 +1%DMSO); flow=0,5ml/min; Vinj 5ul; Temp: 24°C Term.OFF, VWD=220nm; grad. A=H2O+0,1%FA, B=CH3CN+0,1%FA, 0min A=50%, 20min A=0%

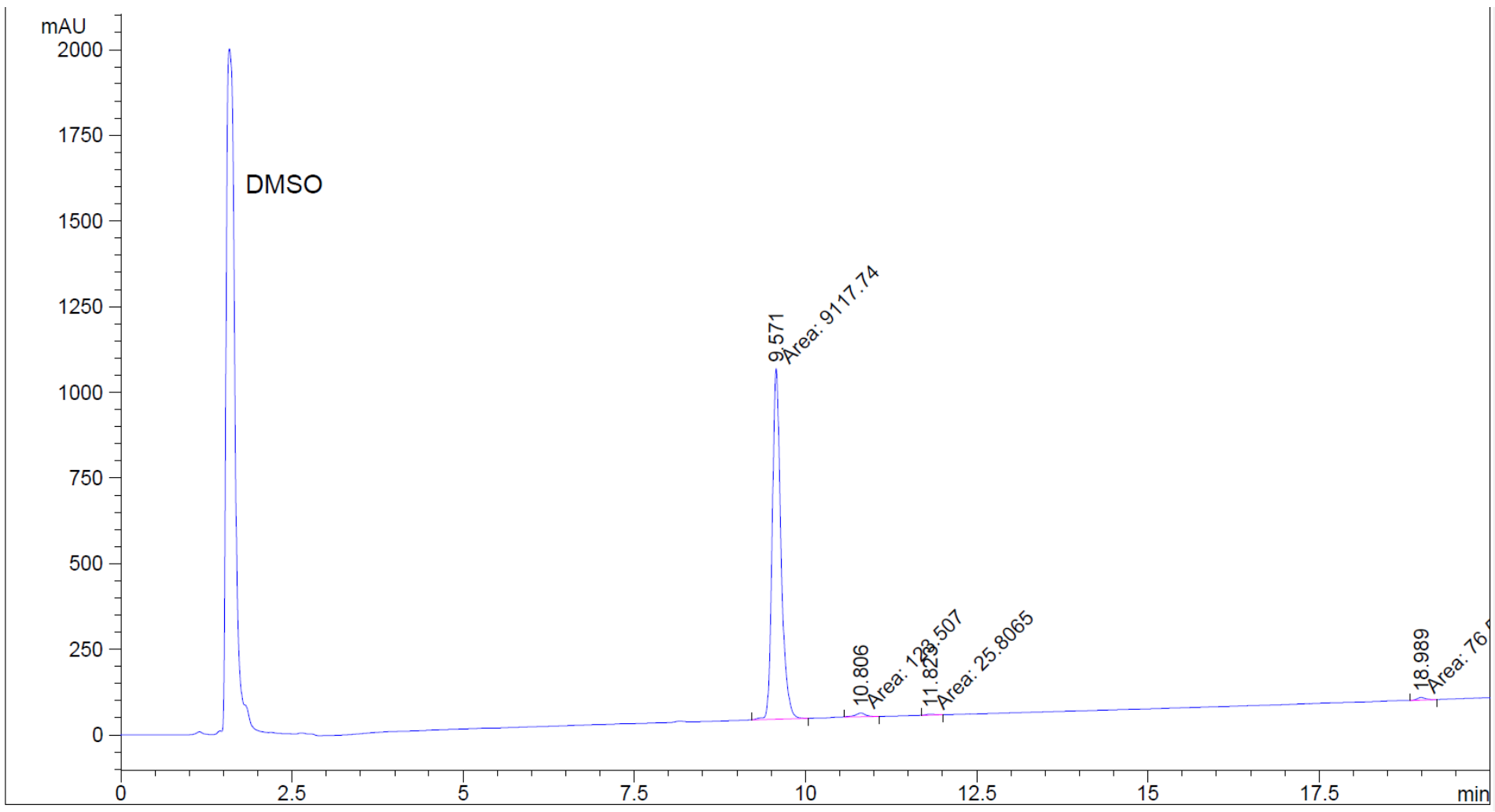

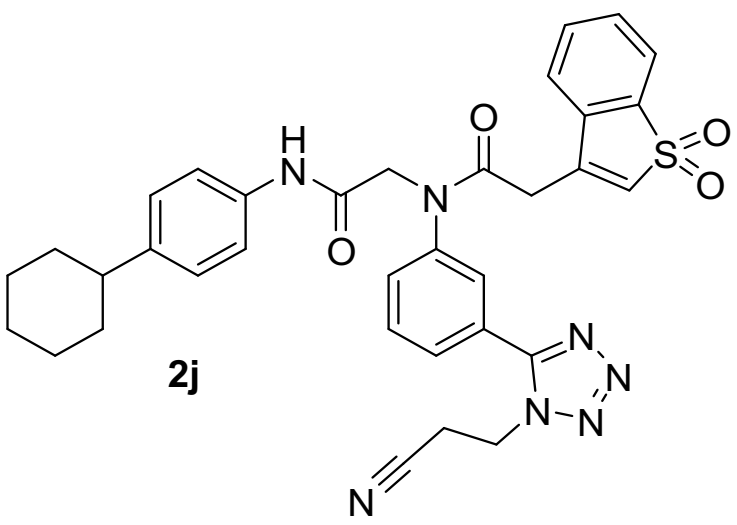

2j

Peak #	RetTime [min]	Type	Width [min]	Area mAU *s	Height [mAU]	Area %
1	3.104	MM	0.1126	122.80843	18.18335	1.5724
2	7.281	MM	0.1439	30.82735	3.56971	0.3947
3	9.835	MM	0.1476	7656.50244	864.59290	98.0329

Column Gemini C18 150x3mm 5u + precolumn + inlet filter; Conc:250ug/ml (CH3CN/H2O+0,1%FA 1:1 +1%DMSO); flow=0,5ml/min;; Vinj 5ul; Temp: 24°C Term.OFF, VWD=220nm; grad. A=H2O+0,1%FA, B=CH3CN+0,1%FA, 0min A=50%, 20min A=0%

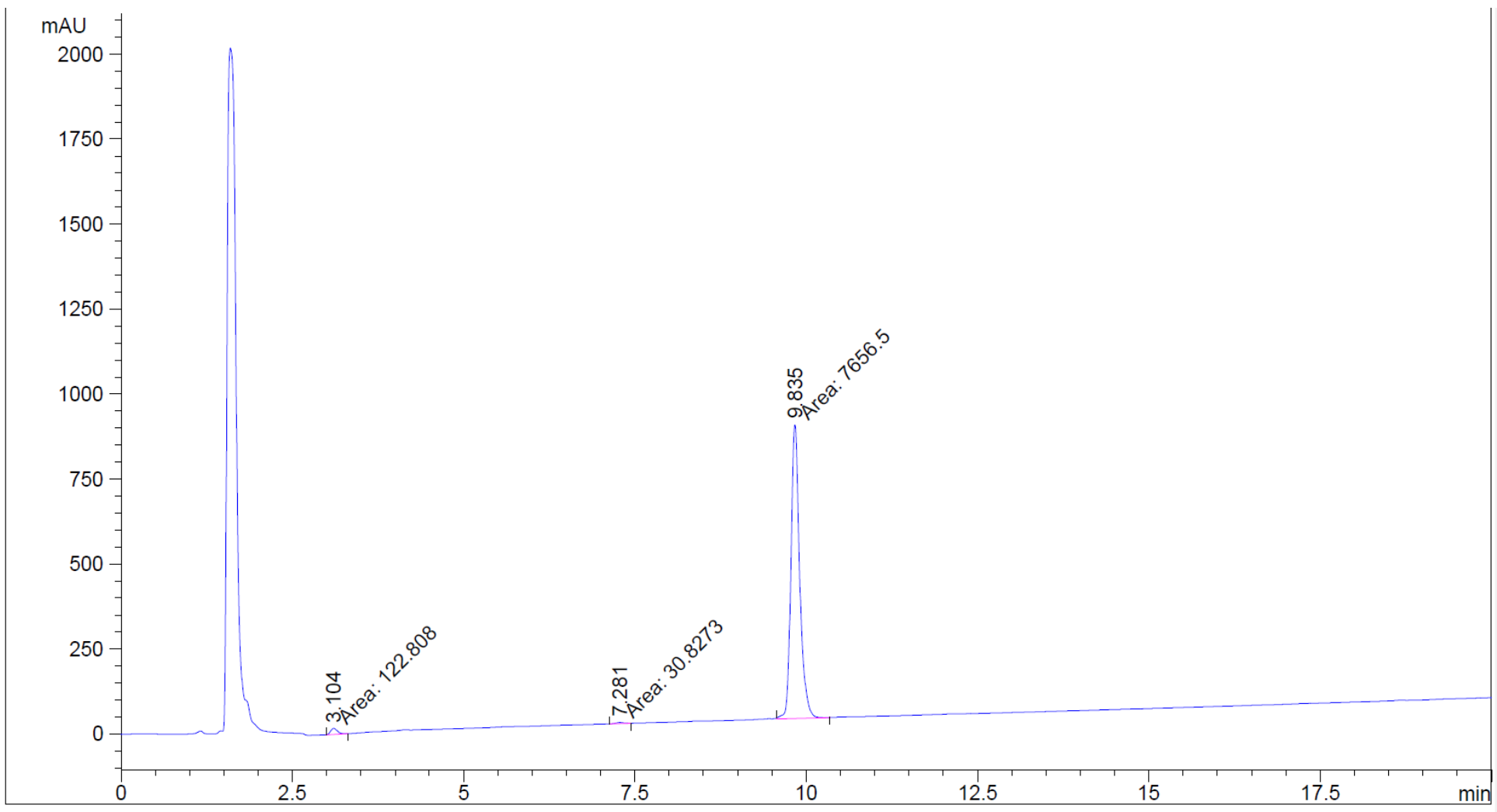

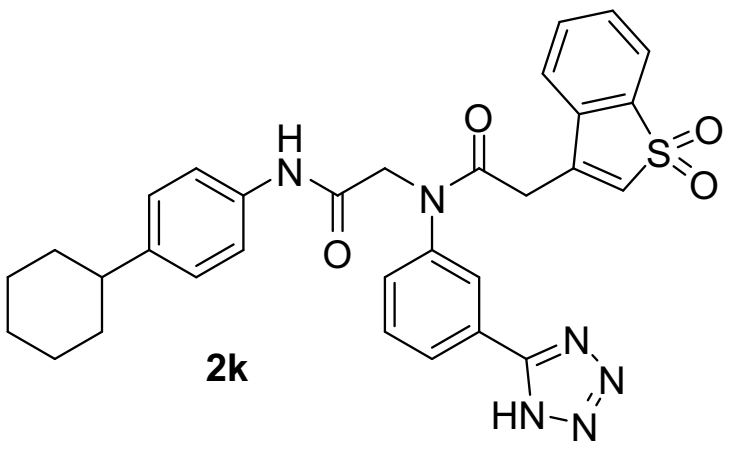

2k

Peak #	RetTime [min]	Type	Width [min]	Area mAU *s	Height [mAU]	Area %
1	9.472	MM	0.1594	3296.44409	344.67654	100.0000

Column Gemini C18 150x3mm 5u + precolumn + inlet filter; Conc: 200ug/ml (CH3CN+0,1%FA +circa1%DMSO); flow=0,5ml/min; Vinj 5ul; Temp: 24°C Term.OFF, VWD=220nm; grad. A=H2O+0,1%FA, B=CH3CN+0,1%FA, 0min A=50%, 20min A=0%

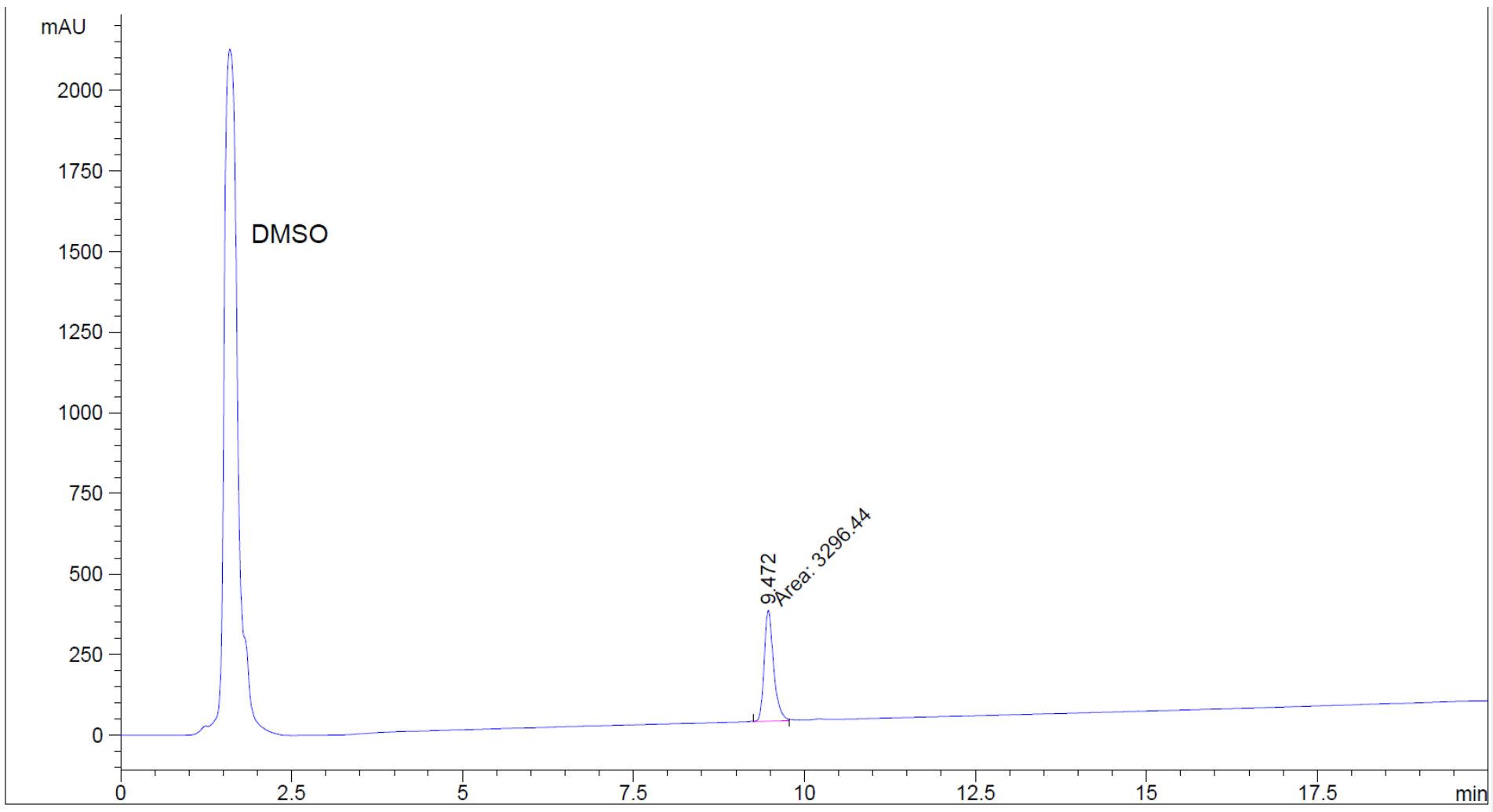

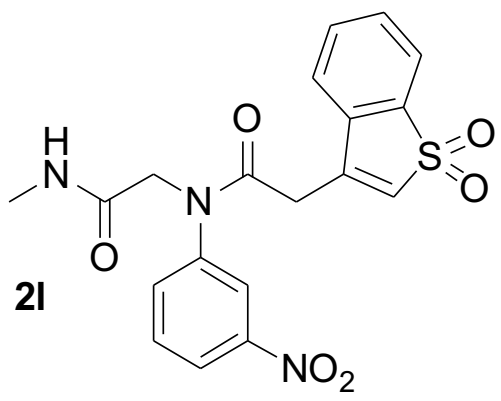

2l

Peak #	RetTime [min]	Type	Width [min]	Area mAU *s	Height [mAU]	Area %
1	9.395	MM	0.1976	6846.52148	577.55872	99.1403
2	10.133	MM	0.1821	59.37031	5.43372	0.8597

Column Phenylic C6 150x3mm 3u + precolumn + inlet filter; Conc:200ug/ml (CH3CN); flow=0,34ml/min; Vinj 5ul; Temp: 26°C Term.OFF, VWD=220nm; grad.A=H2O+0,1%FA, B=CH3CN+0,1%FA,0min A=70%, 20min A=0%

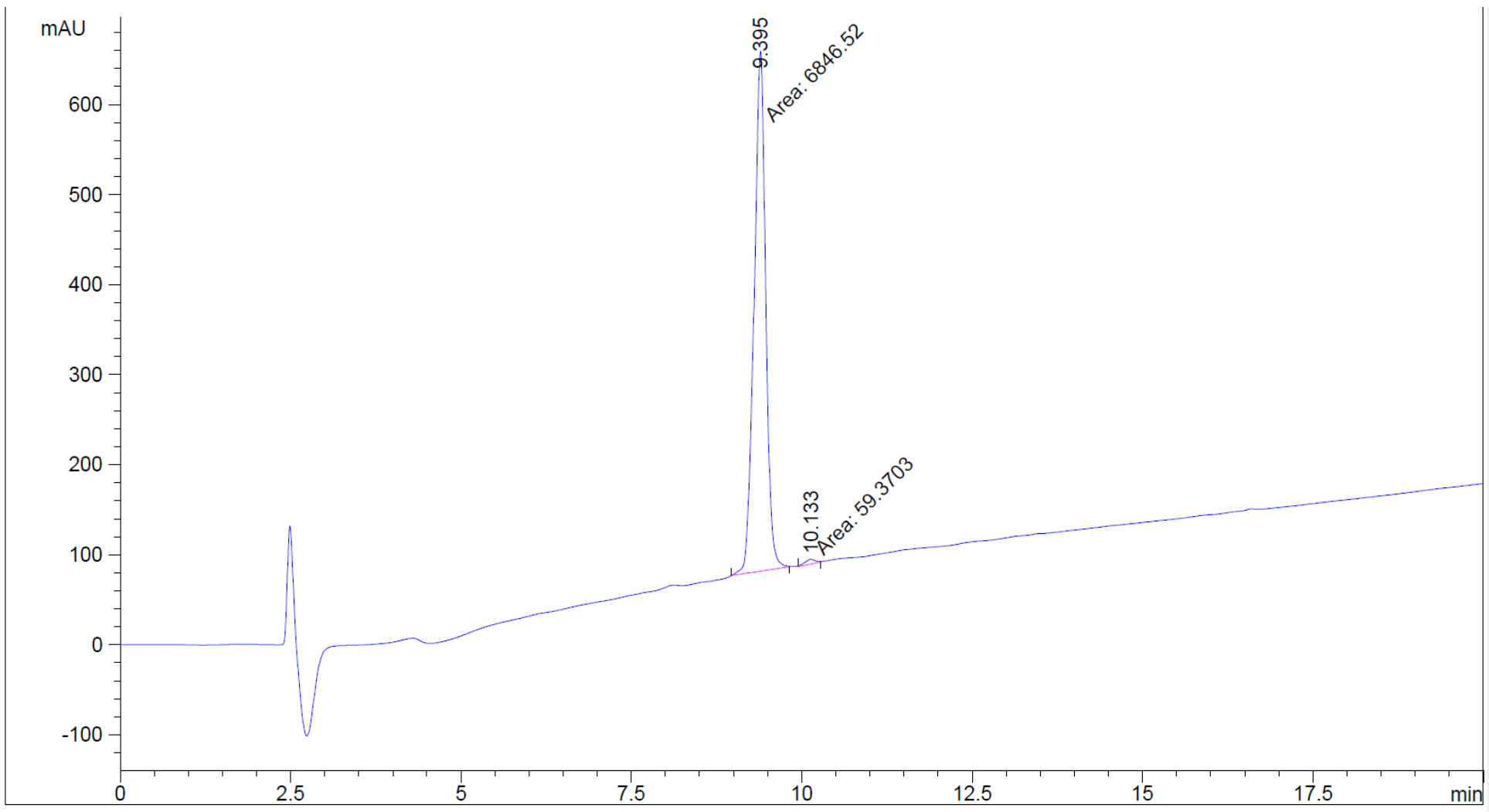

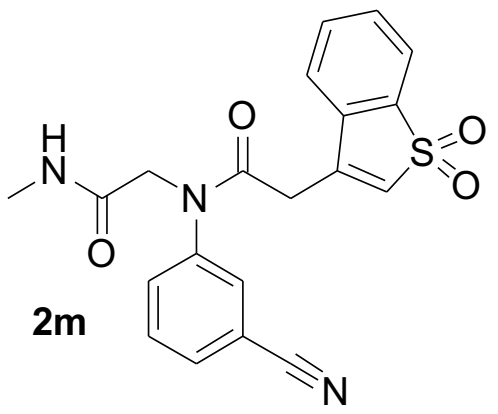

2m

Peak #	RetTime [min]	Type	Width [min]	Area mAU *s	Height [mAU]	Area %
1	8.511	MM	0.2208	1.79071e4	1351.66846	99.4991
2	9.262	MM	0.2014	90.14043	7.45807	0.5009

Column Phenylic C6 150x3mm 3u + precolumn + inlet filter; Conc:300ug/ml (CH3CN); flow=0,34ml/min; Vinj 5ul; Temp: 26°C Term.OFF, VWD=220nm; grad.A=H2O+0,1%FA, B=CH3CN+0,1%FA,0min A=70%, 20min A=0%

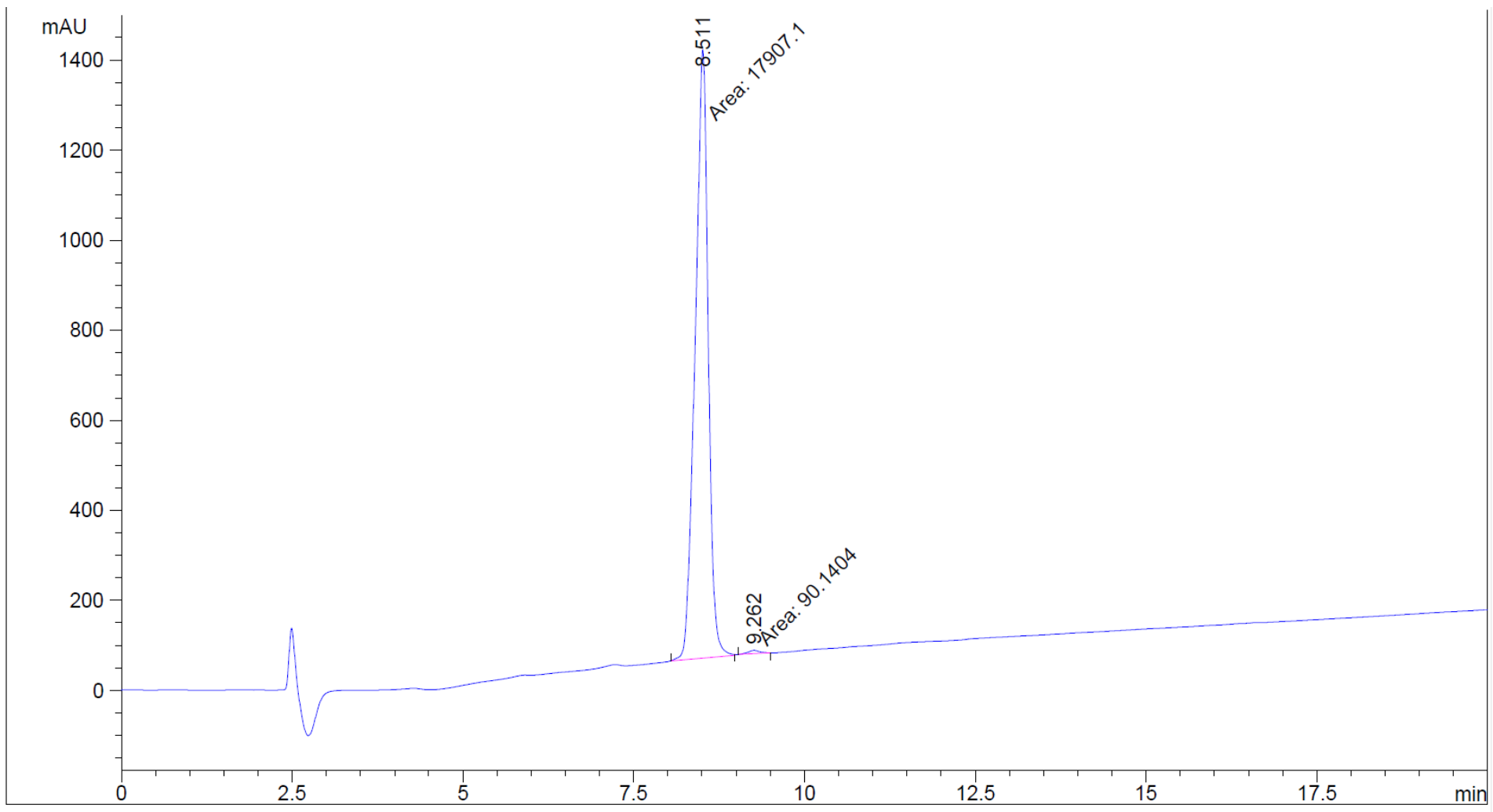

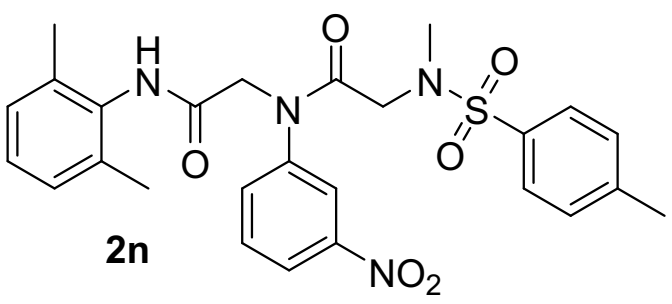

2n

Peak #	RetTime [min]	Type	Width [min]	Area mAU *s	Height [mAU]	Area %
1	4.508	MM	0.1413	78.00109	9.19854	0.8475
2	6.727	MM	0.1517	111.59973	12.26029	1.2125
3	7.785	MM	0.1430	8958.88281	1044.44800	97.3349
4	11.007	MM	0.1545	55.70100	6.00975	0.6052

Column Gemini C18 150x3mm 5u + precolumn + inlet filter; Conc:300ug/ml (CH3CN/H2O+0,1%FA 1:1); flow=0,5ml/min;; Vinj 5ul; Temp: 24°C Term.OFF, VWD=220nm; grad. A=H2O+0,1%FA, B=CH3CN+0,1%FA, 0min A=50%, 20min A=0%

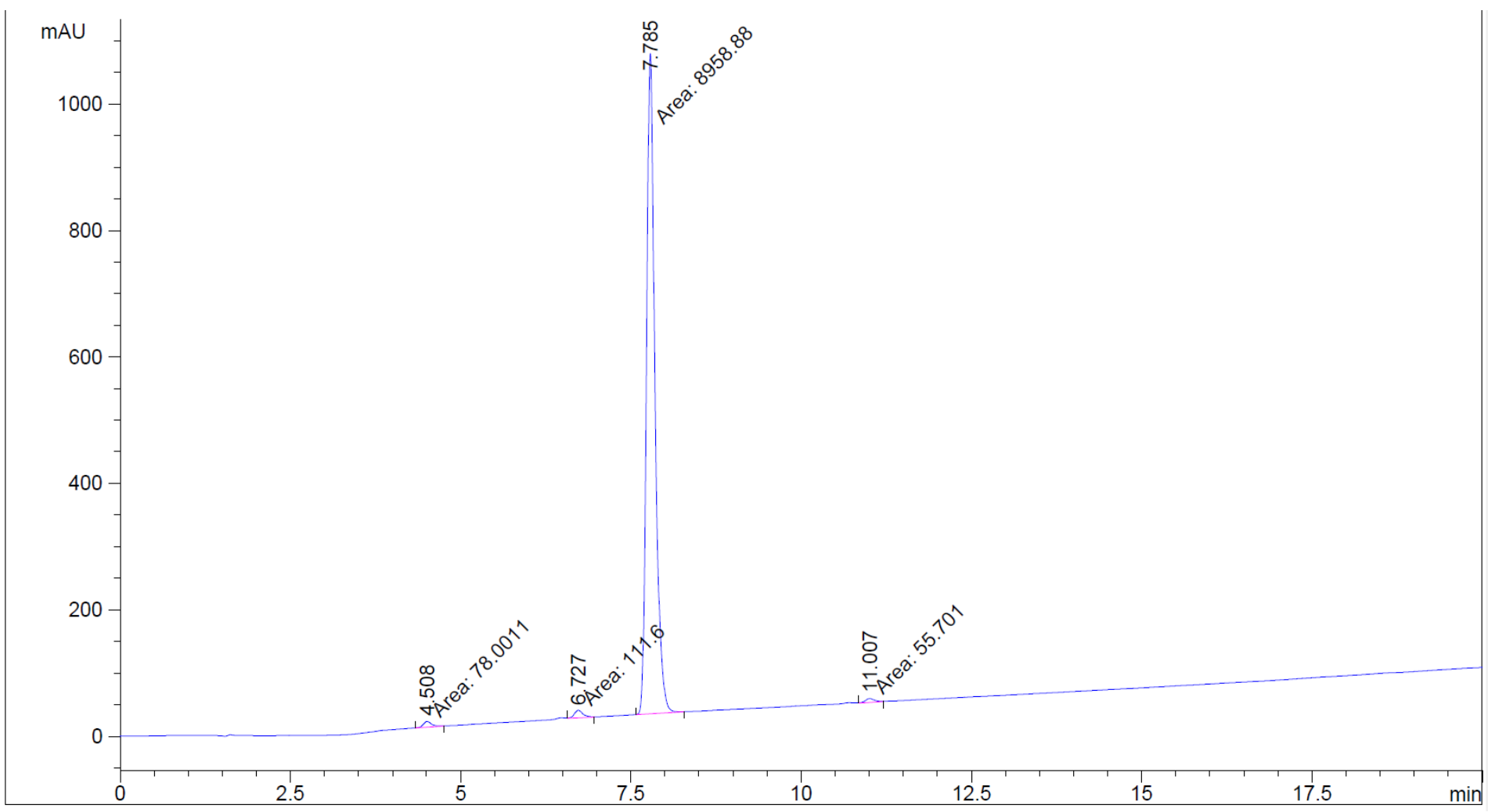

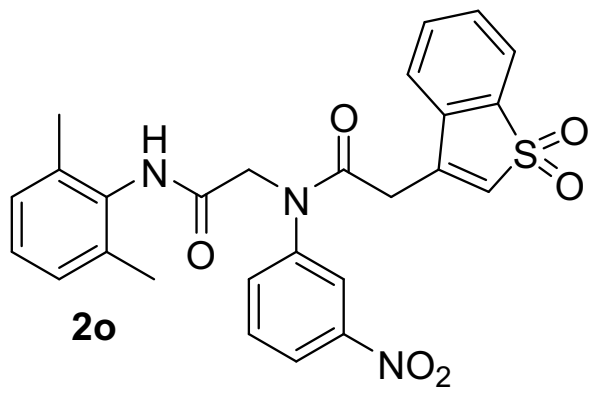

2o

Peak #	RetTime [min]	Type	Width [min]	Area mAU *s	Height [mAU]	Area %
1	13.700	MM	0.1325	19.07823	2.39930	0.3998
2	14.387	MM	0.1304	4696.85107	600.37042	98.4251
3	15.114	MM	0.1203	56.07629	7.77195	1.1751

Column Phenylic C6 150x3mm 3u + precolumn + inlet filter; Conc:100ug/ml (CH3CN + DMSO); flow=0,34ml/min; Vinj 5ul; Temp: 25˚C Term.ON, VWD=220nm; grad.A=H2O, B=CH3CN,0min A=70%, 20min A=0%

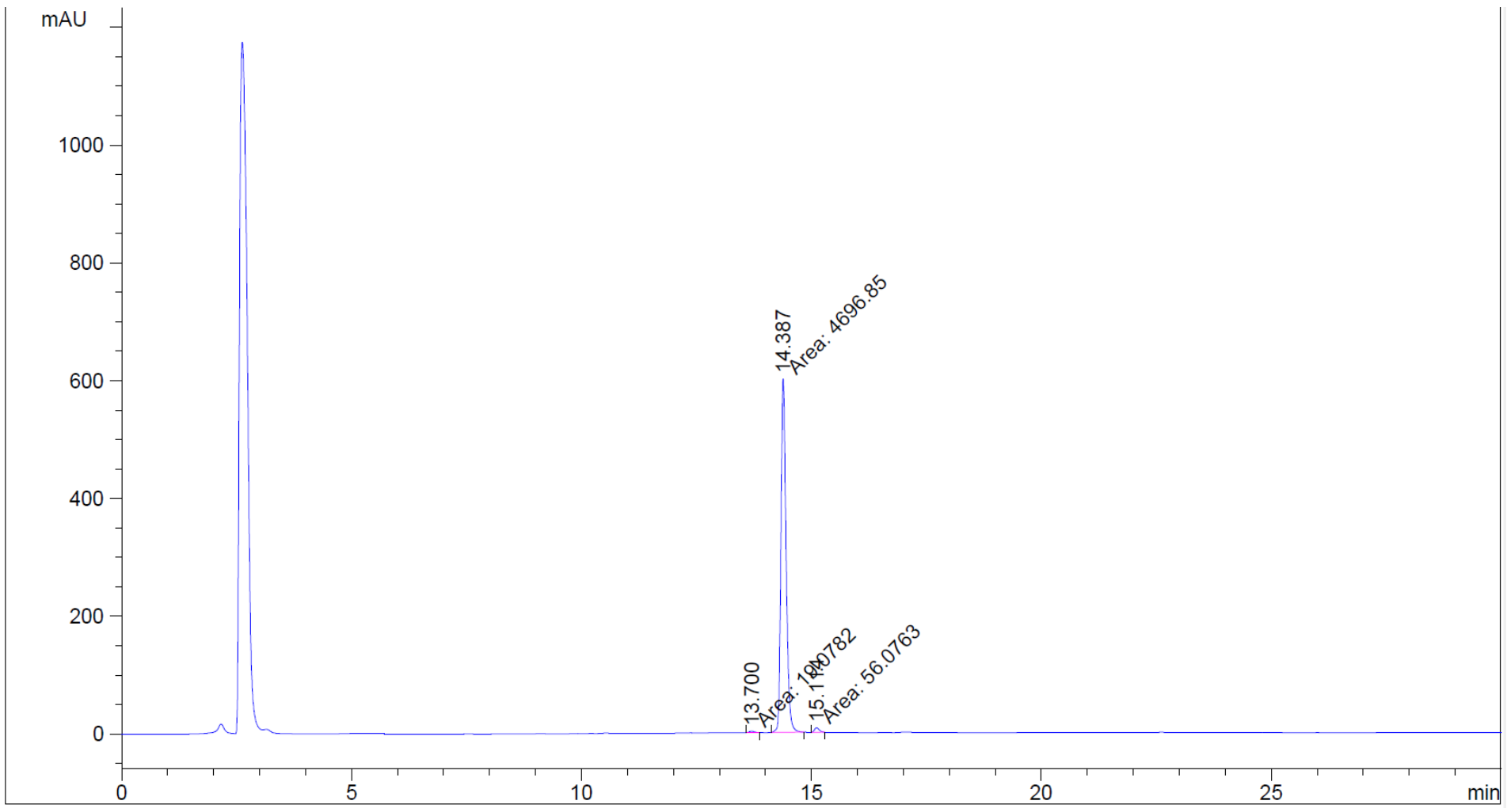

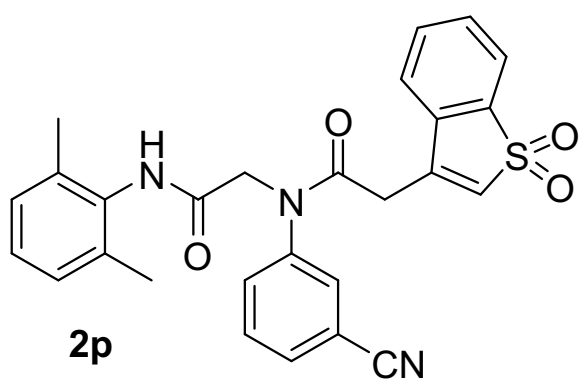

2p

Peak #	RetTime [min]	Type	Width [min]	Area mAU *s	Height [mAU]	Area %
1	5.363	MM	0.1579	9145.37012	965.39020	98.9526
2	6.398	MM	0.1539	96.80252	10.48147	1.0474

Column Gemini C18 150x3mm 5u + precolumn + inlet filter;Conc:200ug/ml (CH3CN+0,1%FA+1%DMSO); flow=0,5ml/min;; Vinj 5ul; Temp: 26˚C Term.OFF, VWD=220nm; grad. A=H2O+0,1%FA, B=CH3CN+0,1%FA, 0min A=50%, 20min A=0%

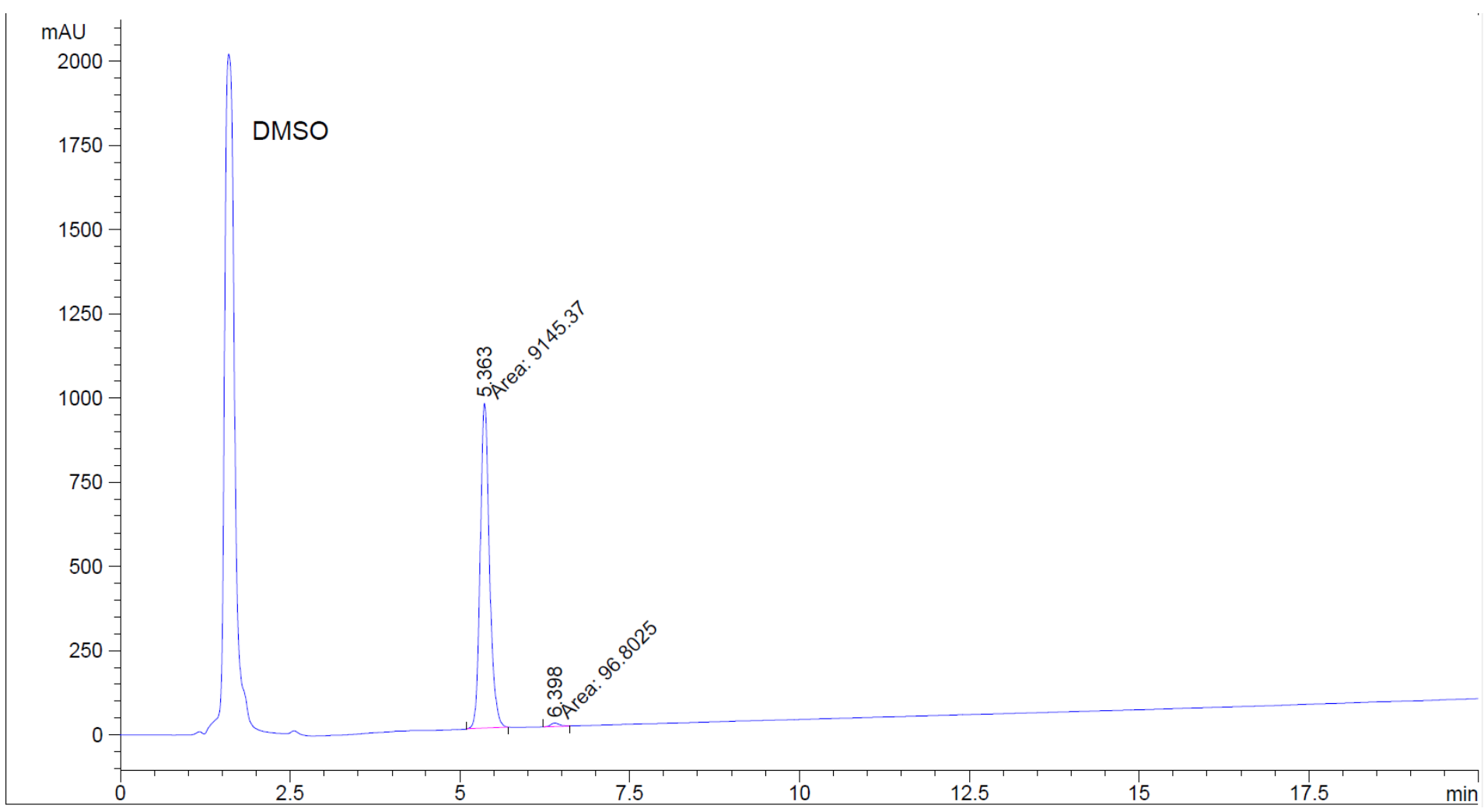

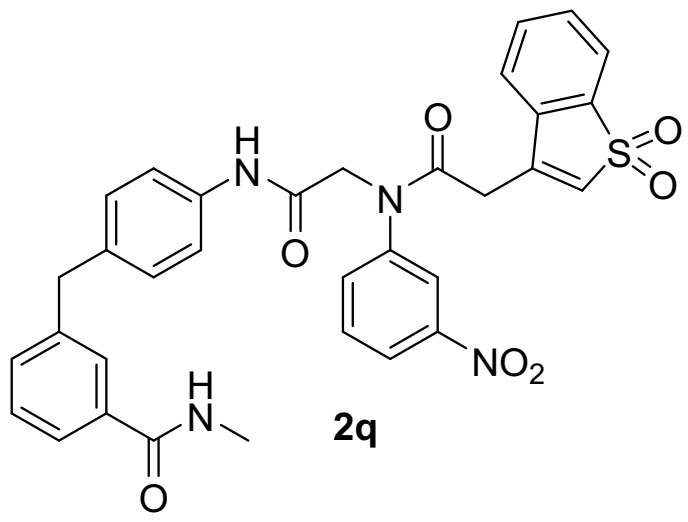

2q

Peak #	RetTime [min]	Type	Width [min]	Area mAU *s	Height [mAU]	Area %
1	5.068	MM	0.1401	225.59012	26.83553	2.0229
2	7.011	MF	0.1268	161.01917	21.15667	1.4439
3	7.330	FM	0.1213	1.07652e4	1479.26575	96.5332

Column ACE Excel 3 C18-AR 150 x 3 mm, 3 micron + precolumn + inline filter; Conc:250ug/ml (CH3CN/H2O+0,1%FA 1:1+1%DMSO); flow=0,38ml/min; Vinj 5ul; Temp: 30°C Term.ON, VWD=220nm ; grad.A=H2O+0,1%FA, B=CH3CN+0,1%FA,0min A=50%, 20min A=0%

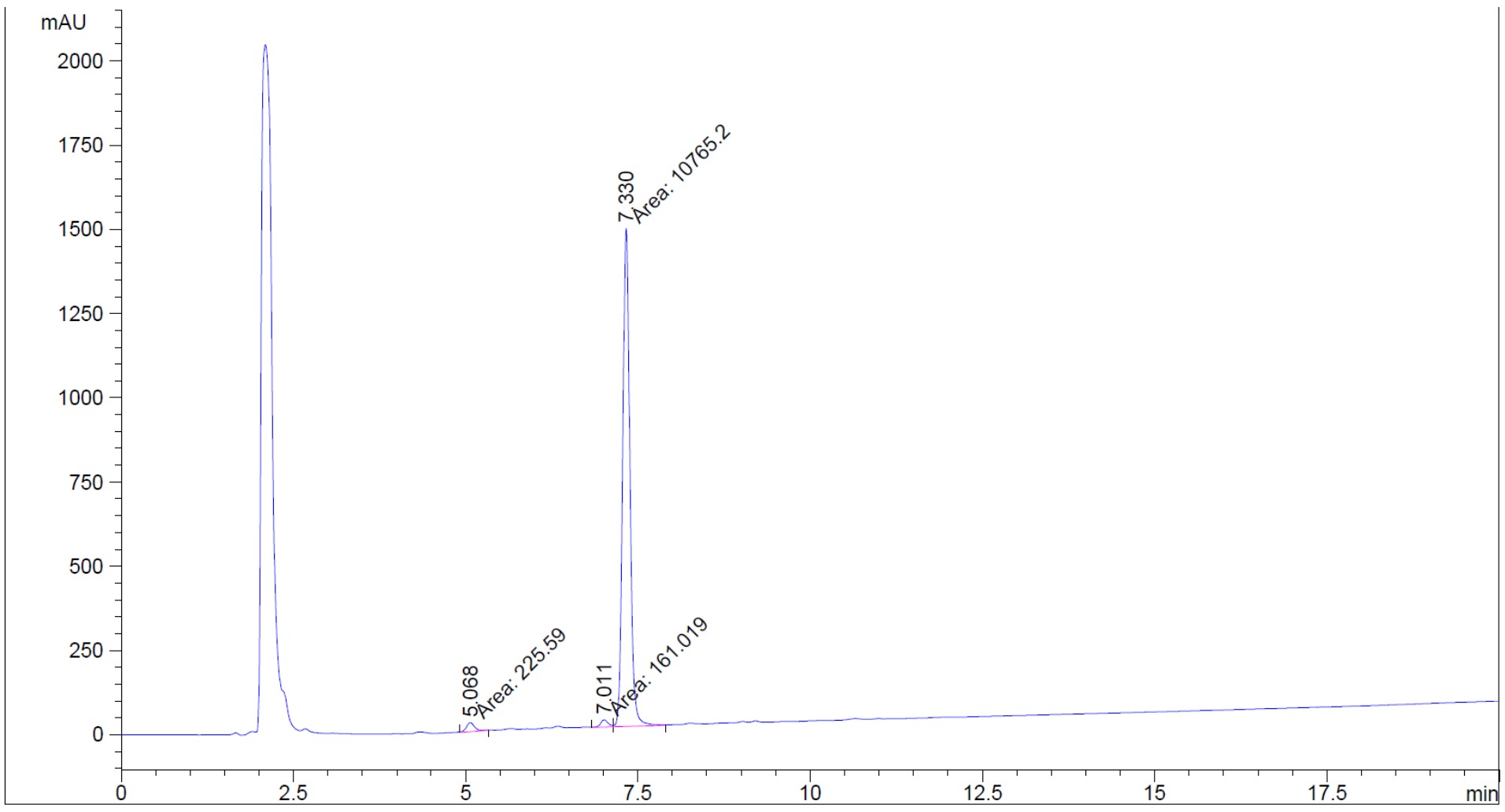

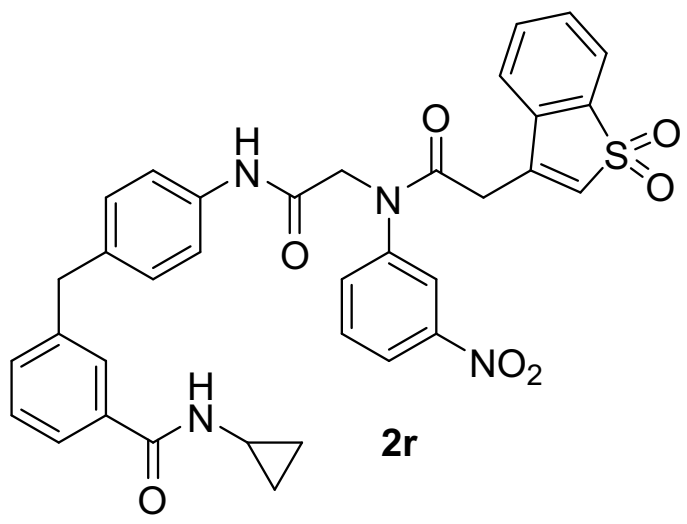

2r

Peak #	RetTime [min]	Type	Width [min]	Area mAU *s	Height [mAU]	Area %
1	2.446	MM	0.1553	61.23285	6.57206	0.7516
2	2.782	MM	0.1883	44.59555	3.94634	0.5474
3	4.734	MM	0.1353	67.45847	8.30924	0.8280
4	5.002	MM	0.1127	21.57635	3.19211	0.2648
5	5.373	MM	0.1085	10.89709	1.67447	0.1337
6	5.624	MM	0.1351	21.43795	2.64432	0.2631
7	6.144	MF	0.1302	82.59026	10.57604	1.0137
8	6.440	FM	0.1408	7785.97461	921.96088	95.5639
9	7.358	MM	0.1617	23.89272	2.46313	0.2933
10	9.699	MM	0.1849	27.74788	2.50051	0.3406

Column Gemini C18 150x3mm 5u + precolumn + inlet filter; Conc:250ug/ml (CH3CN/H2O+0,1%FA 1:1 +1% DMSO); flow=0,5ml/min;; Vinj 5ul; Temp: 24°C Term.OFF, VWD=220nm; grad. A=H2O+0,1%FA, B=CH3CN+0,1%FA, 0min A=50%, 20min A=0%

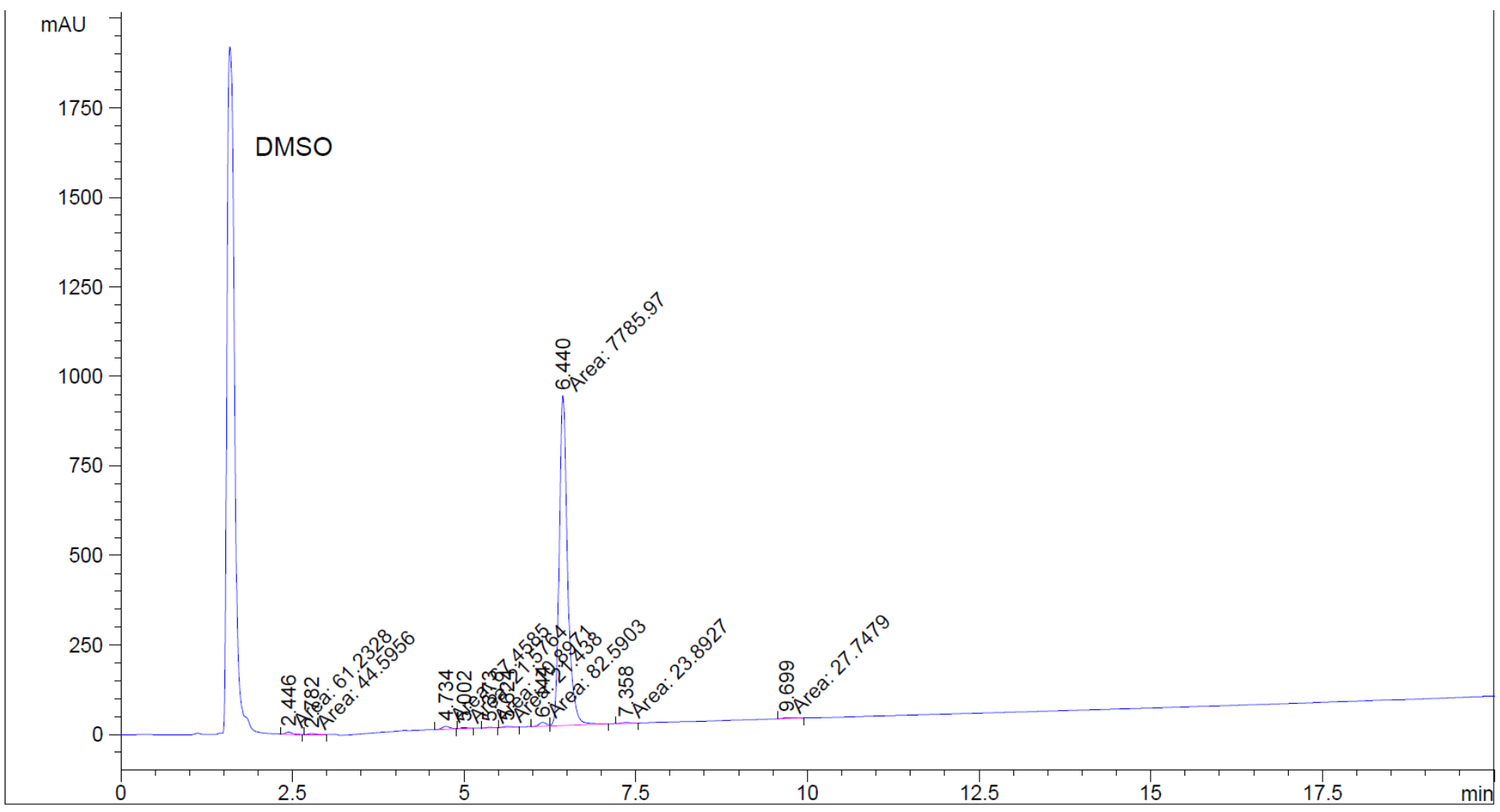

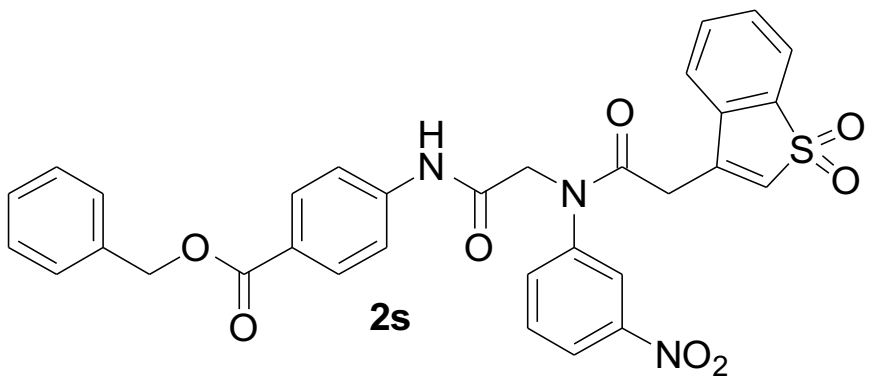

Peak #	RetTime [min]	Type	Width [min]	Area mAU *s	Height [mAU]	Area %
1	9.369	MM	0.1432	63.27843	7.36332	0.6474
2	9.760	MM	0.1411	9710.58691	1147.24561	99.3526

Column Gemini C18 150x3mm 5u + precolumn + inlet filter; Conc:300ug/ml (CH3CN/H2O+0,1%FA 1:1); flow=0,5ml/min;; Vinj 5ul; Temp: 26°C Term.OFF, VWD=220nm; grad. A=H2O+0,1%FA, B=CH3CN+0,1%FA, 0min A=50%, 20min A=0%

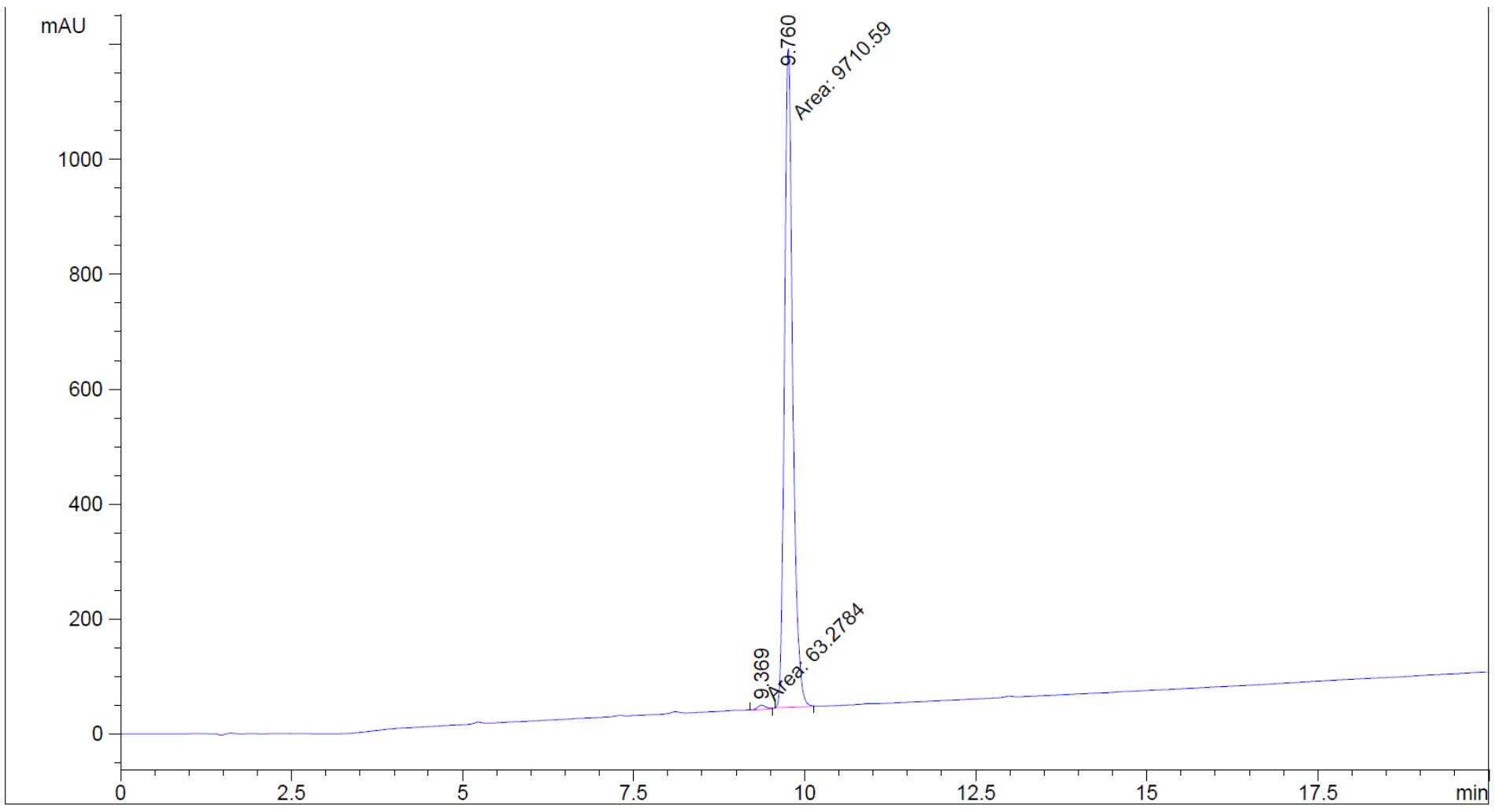

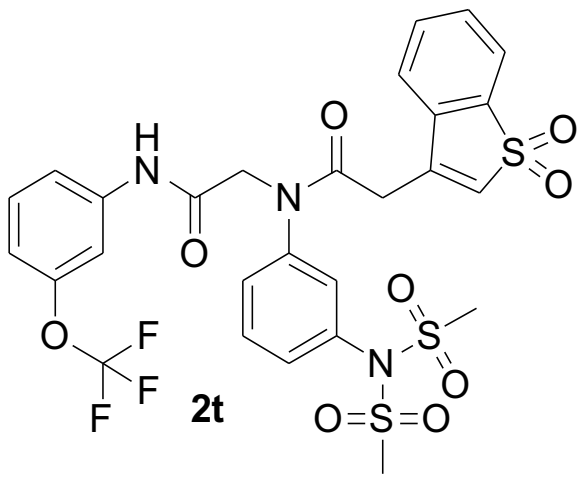

Peak #	RetTime [min]	Type	Width [min]	Area mAU *s	Height [mAU]	Area %
1	9.686	MM	0.1884	7014.05322	620.40216	95.7183
2	11.943	MM	0.1600	313.75278	32.68757	4.2817

Column Phenylic C6 150x3mm 3u + precolumn + inlet filter; Conc:200ug/ml(CH3CN); flow=0,34ml/min; Vinj 5ul; Temp: 25°C Term.ON,VWD=220nm; grad.A=H2O+0,1%FA, B=CH3CN+0,1%FA,0minA=50%, 20min A=0%

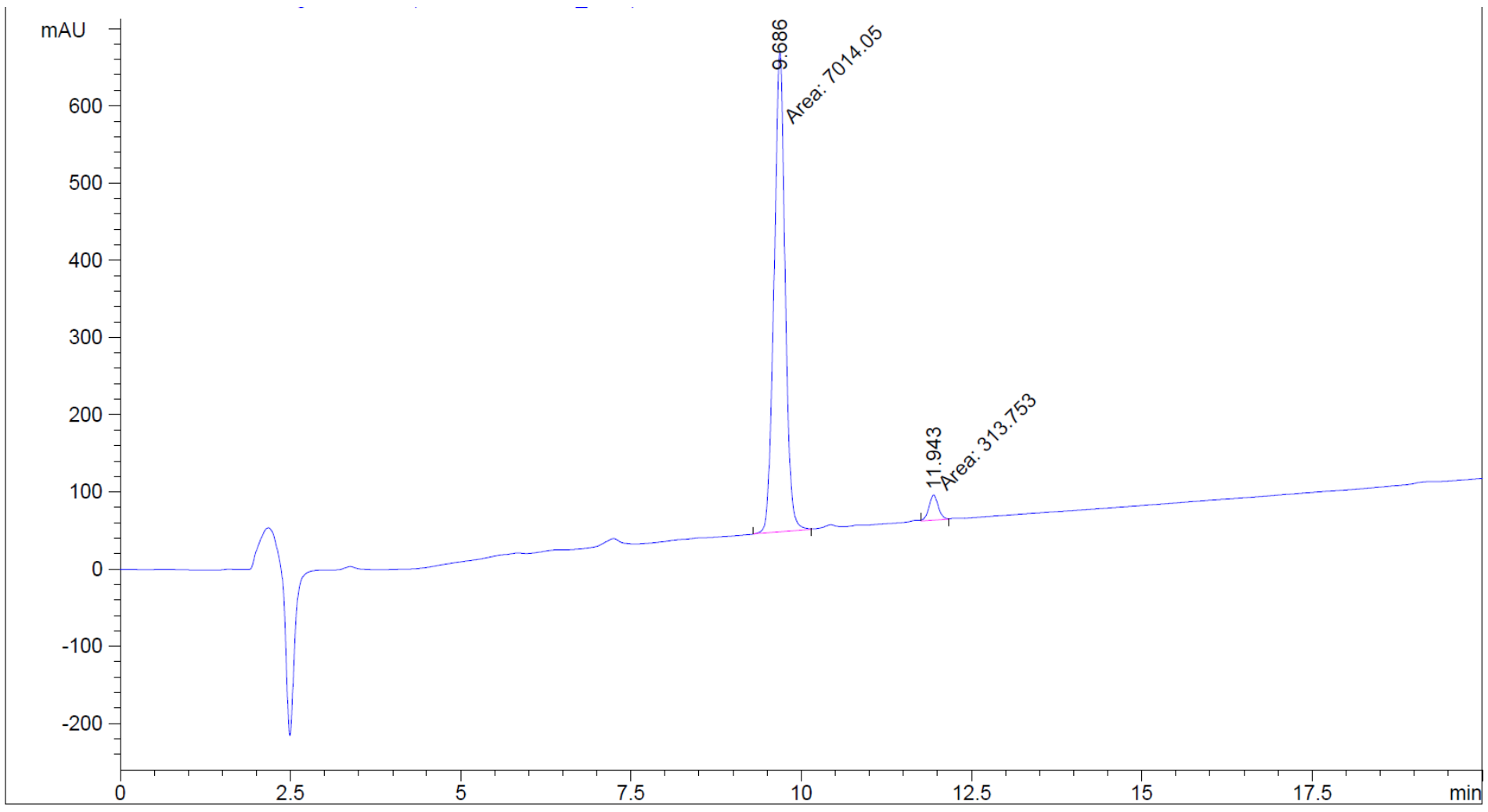

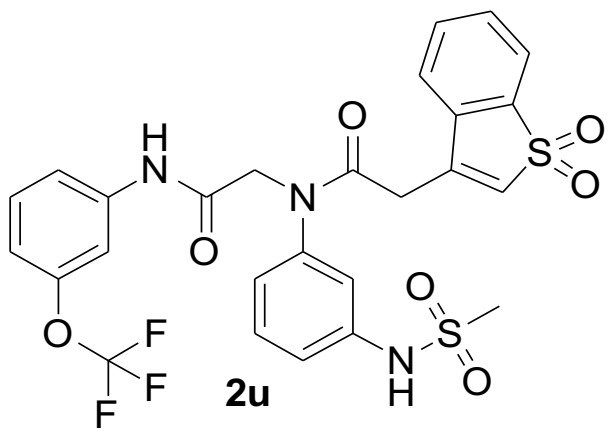

2u

Peak #	RetTime [min]	Type	Width [min]	Area mAU *s	Height [mAU]	Area %
1	8.182	MM	0.2152	9395.89453	727.64294	96.0899
2	9.217	MM	0.1860	116.34533	10.42529	1.1898
3	10.797	MF	0.1875	66.66532	5.92429	0.6818
4	11.133	FM	0.1715	199.32353	19.37337	2.0384

Column Phenylic C6 150x3mm 3u + precolumn + inlet filter; Conc:200ug/ml (CH3CN); flow=0,34ml/min; Vinj 5ul; Temp: 25°C Term.ON,VWD=220nm; grad.A=H2O+0,1%FA, B=CH3CN+0,1%FA,0minA=50%, 20min A=0%

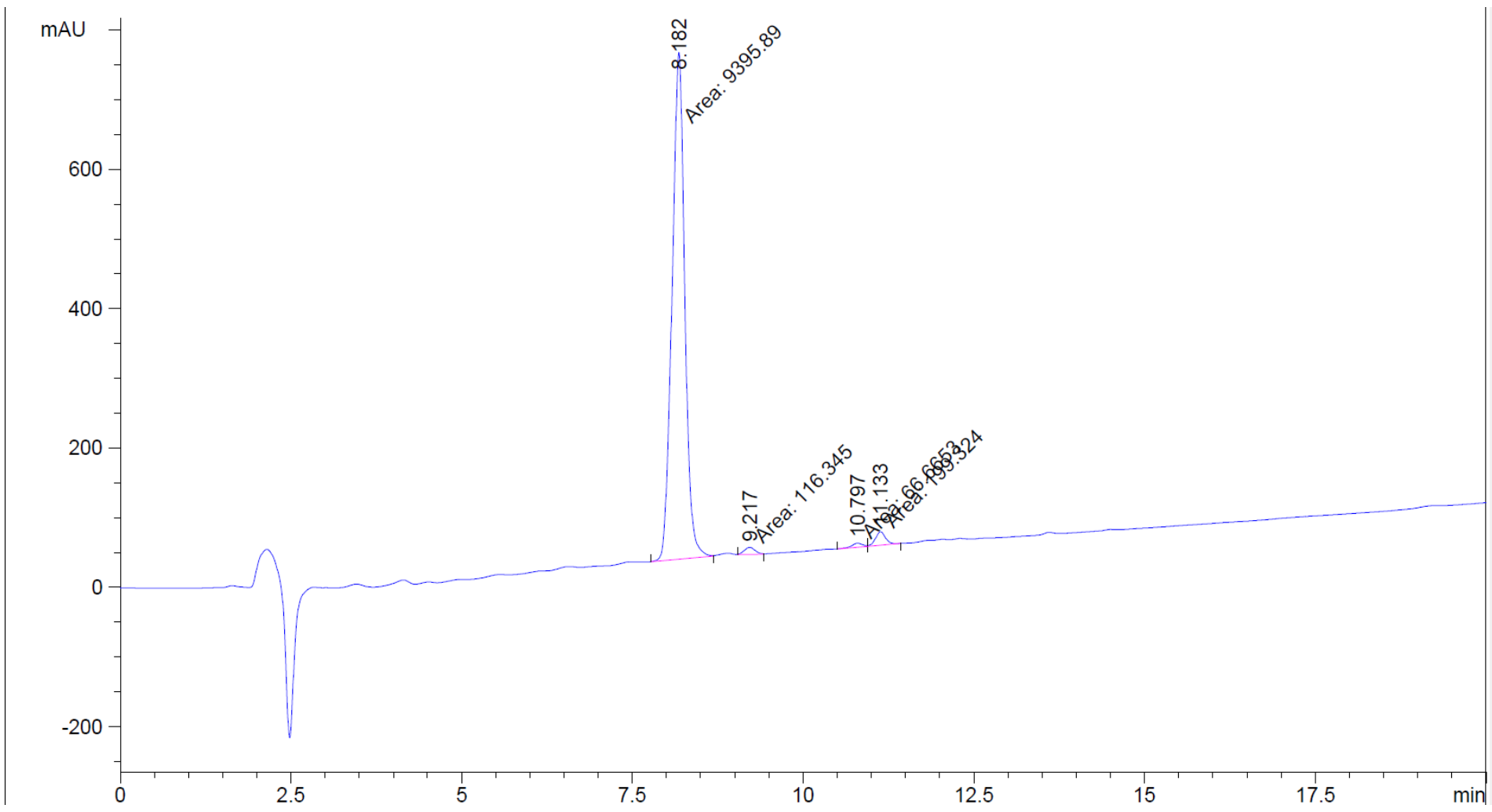

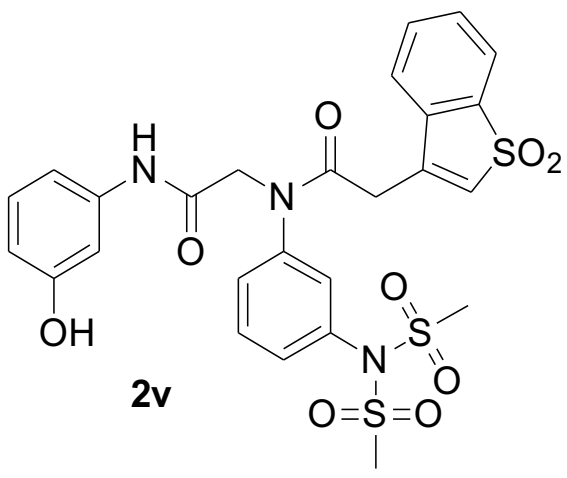

2v

Peak #	RetTime [min]	Type	Width [min]	Area mAU *s	Height [mAU]	Area %
1	13.313	MM	0.0926	121.18446	21.81840	2.4599
2	14.681	MM	0.0922	4805.25879	868.89972	97.5401

Column ACE Excel 3 C18-AR 150 x 3 mm, 3 micron + precolumn + inline filter; Conc:200ug/ml (CH3CN+0,1%FA); flow=0,38ml/min; Vinj 5ul; Temp: 30°C Term.ON, VWD=220nm ; grad.A=H2O+0,1%FA, B=CH3CN+0,1%FA,0min A=90%, 20min A=0%

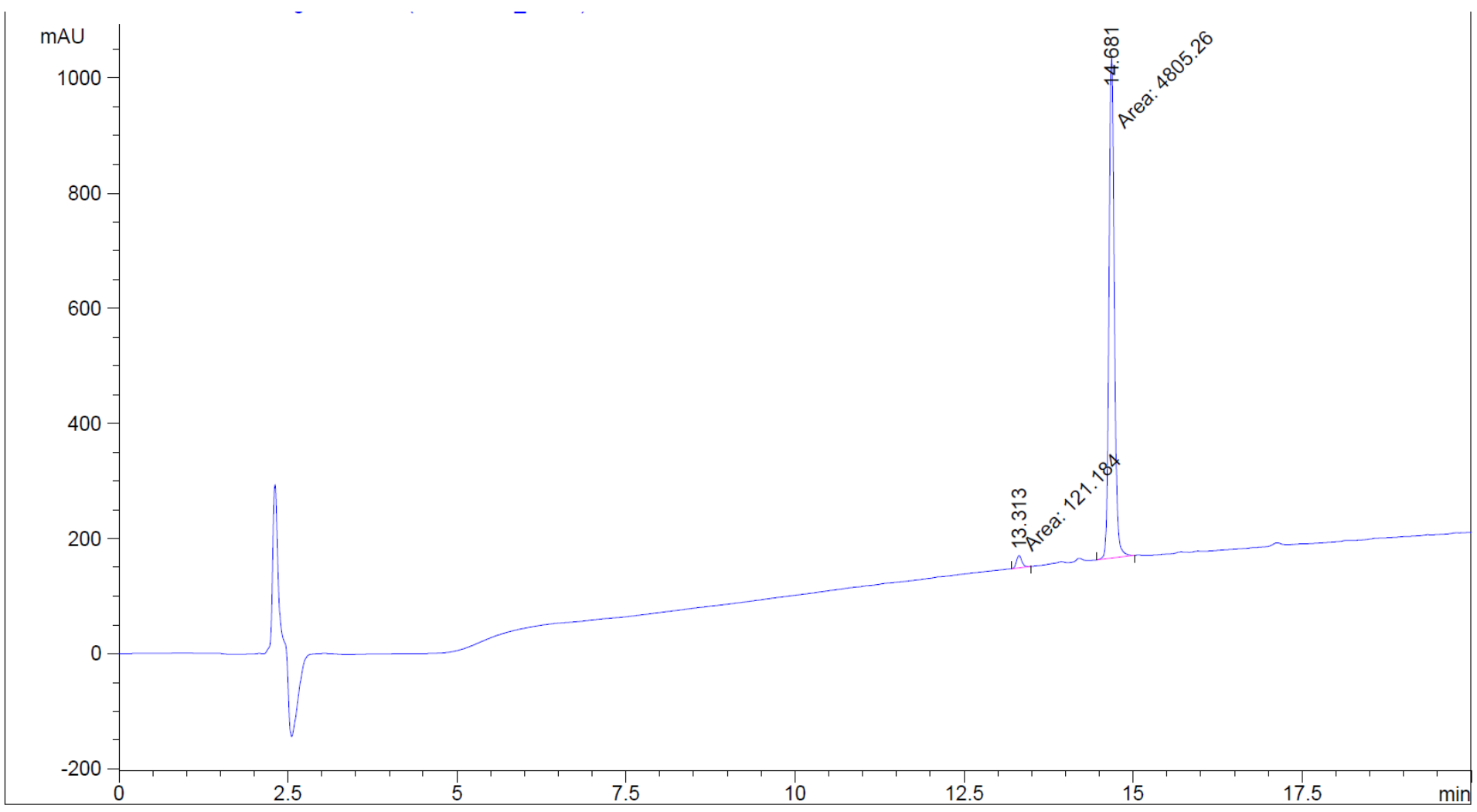

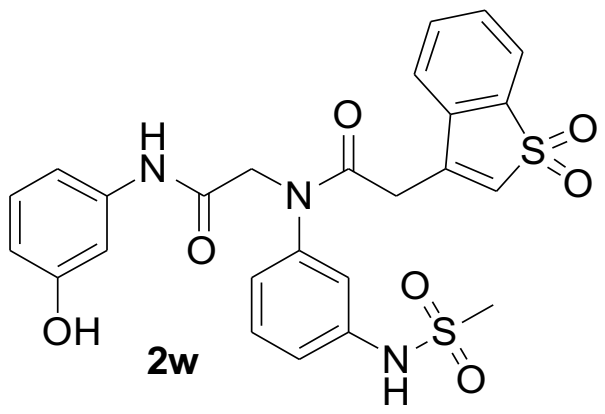

2w

Peak #	RetTime [min]	Type	Width [min]	Area mAU *s	Height [mAU]	Area %
1	10.756	MM	0.1571	354.13190	37.58041	2.3900
2	13.658	MM	0.1429	1.42452e4	1661.96741	96.1401
3	15.172	MM	0.1699	217.79245	21.36161	1.4699

Column Phenylic C6 150x3mm 3u + precolumn + inlet filter; Conc:200ug/ml (CH3CN); flow=0,34ml/min; Vinj 5ul; Temp: 25°C Term.ON,VWD=220nm; grad.A=H2O+0,1%FA, B=CH3CN+0,1%FA,0min A=90%, 20min A=0%

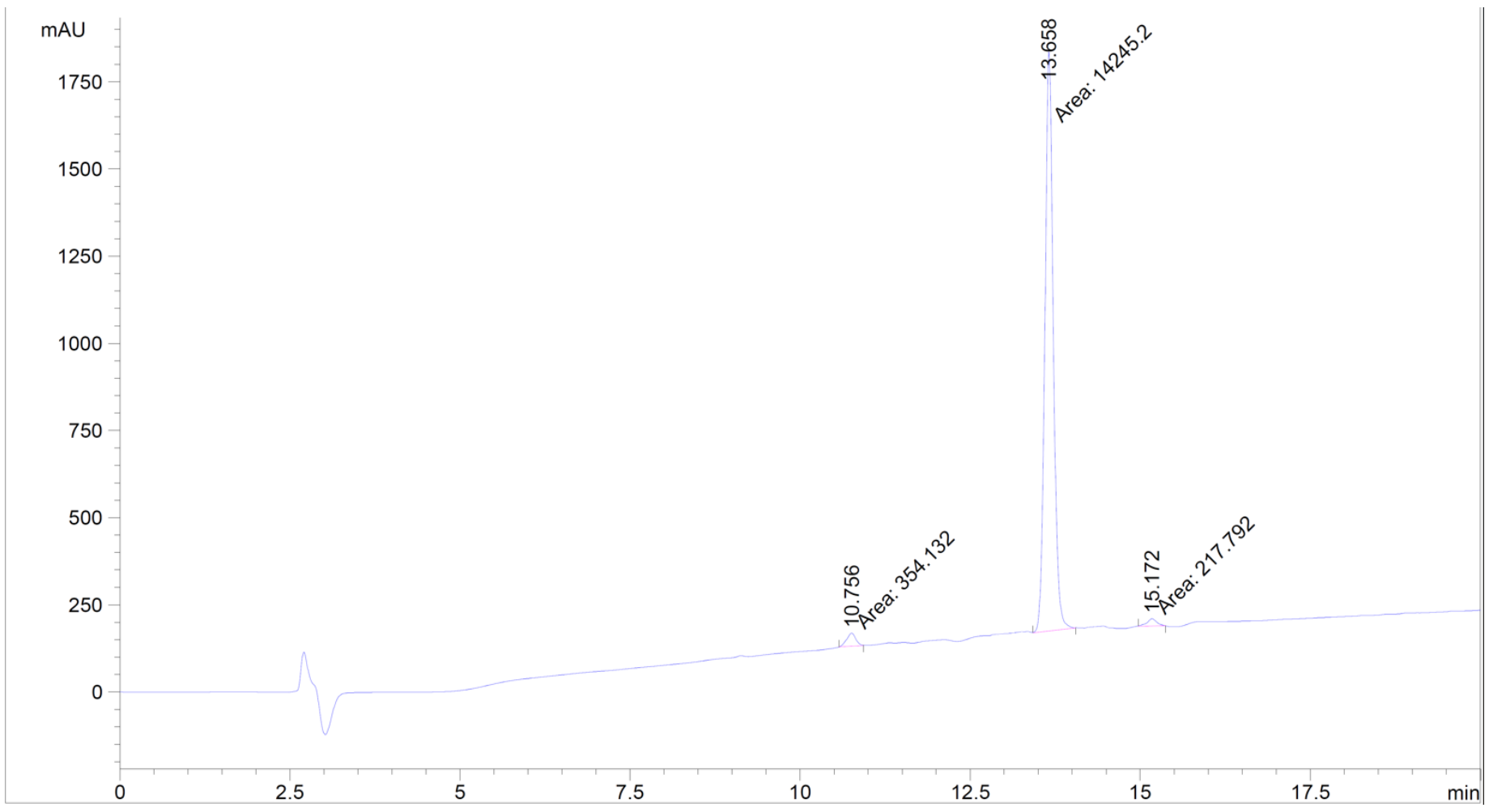

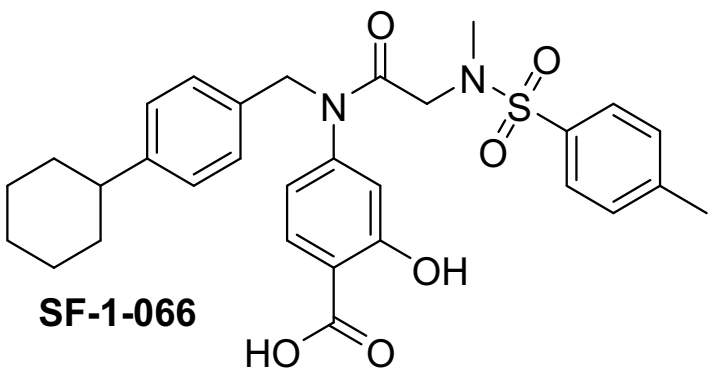

SF-1-066

Peak #	RetTime [min]	Type	Width [min]	Area mAU *s	Height [mAU]	Area %
1	12.042	MM	0.4180	9045.14551	360.67972	100.0000

Column Phenylic C6 150x3mm 3u + precolumn + inlet filter; Conc:200mg/ml (CH3CN/H2O+0,1%FA 1:1); flow=0,34ml/min; Vinj 5ul; Temp: 25°C Term.ON, VWD=220nm; grad. A=H2O+0,1%FA , B=CH3CN+0,1%FA, 0min A=30%, 20min A=0%

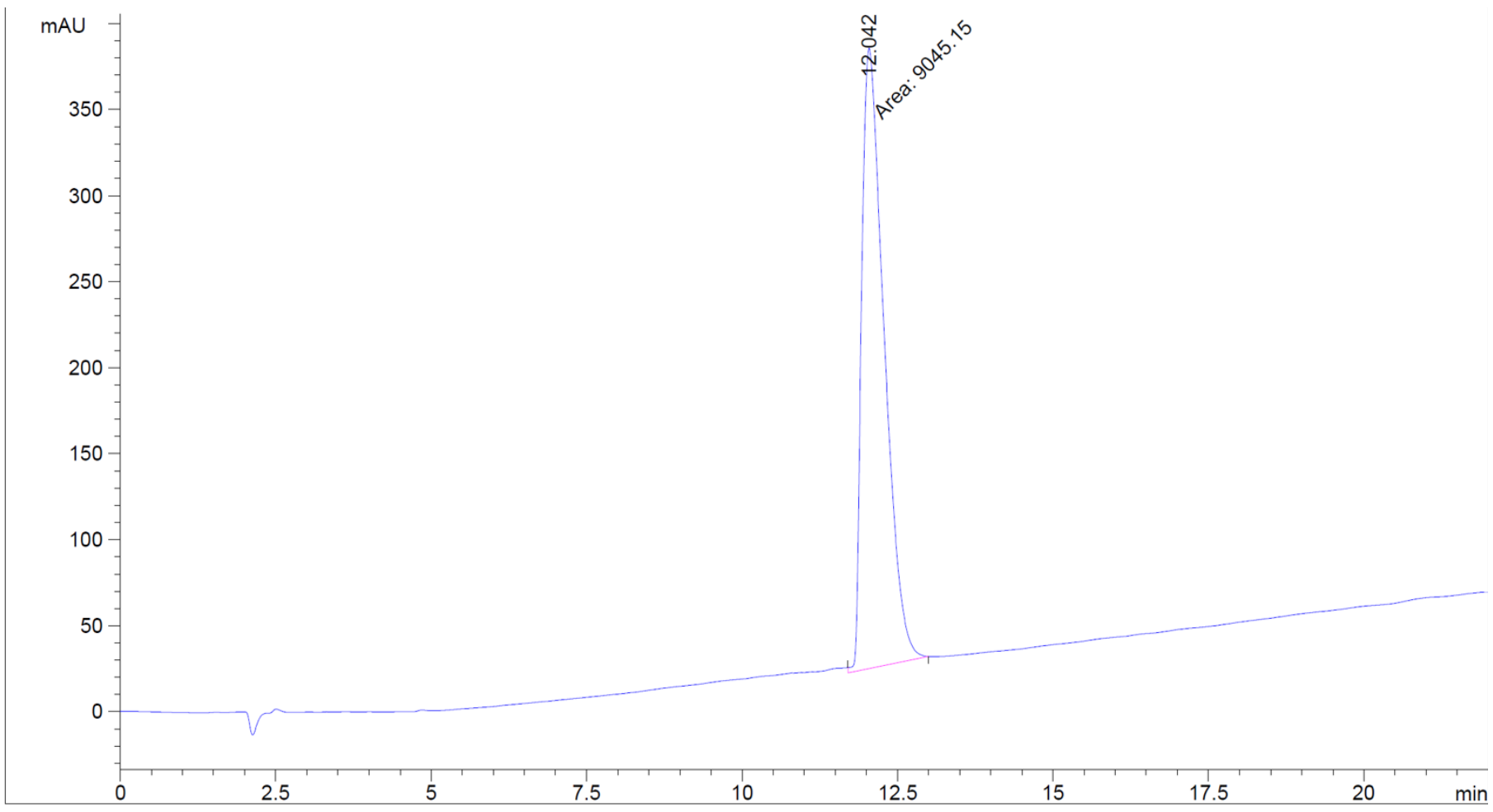

Supplement: Supplementary file 1 [file ao6c07733_si_001.pdf]
